# Gastrodin Derivatives from *Gastrodia elata*

**DOI:** 10.1007/s13659-019-00224-1

**Published:** 2019-11-16

**Authors:** Cheng-Bo Xu, Qing-Lan Guo, Ya-Nan Wang, Sheng Lin, Cheng-Gen Zhu, Jian-Gong Shi

**Affiliations:** State Key Laboratory of Bioactive Substance and Function of Natural Medicines, Institute of Materia Medica, Chinese Academy of Medical Sciences and Peking Union Medical College, Beijing, 100050 People’s Republic of China

**Keywords:** Orchidaceae, *Gastrodia elata* Blume, *p*-Hydroxybenzyl gastrodin ethers, Reaction of gastrodin with *p*-hydroxybenzyl alcohol, Component variation during decocting

## Abstract

**Abstract:**

Nine new gastrodin derivatives, including seven *p*-hydroxybenzyl-modified gastrodin ethers (**1**–**7**), 6′-*O*-acetylgastrodin (**8**), and 4-[*α*-d-glucopyranosyl-(1 →6)-*β*-d-glucopyranosyloxy]benzyl alcohol (**9**), together with seven known derivatives, were isolated from an aqueous extract of *Gastrodia elata* (“tian ma”) rhizomes. Their structures were determined by spectroscopic and chemical methods as well as single crystal X-ray diffraction. Compounds **1**–**4**, **7**, **10**, and **11** were also isolated from a reaction mixture by refluxing gastrodin and *p*-hydroxybenzyl alcohol in H_2_O. As both gastrodin and *p*-hydroxybenzyl alcohol exist in the plant, the reaction results provide evidence for the production and increase/decrease of potential effective/toxic components when “tian ma” is decocted solely or together with ingredients in Chinese traditional medicine formulations, though the isolates were inactive in the preliminarily cell-based assays at concentrations of 10 μM. Moreover, using ultra-performance liquid chromatography high-resolution electrospray ionization mass spectrometry (UPLC-HRESIMS), **4**, **7**, **10**, and **11**, as well as component variations, were detectable in the freshly prepared extracts of different types of samples, including the freeze-dried fresh *G. elata* rhizomes.

**Graphic Abstract:**

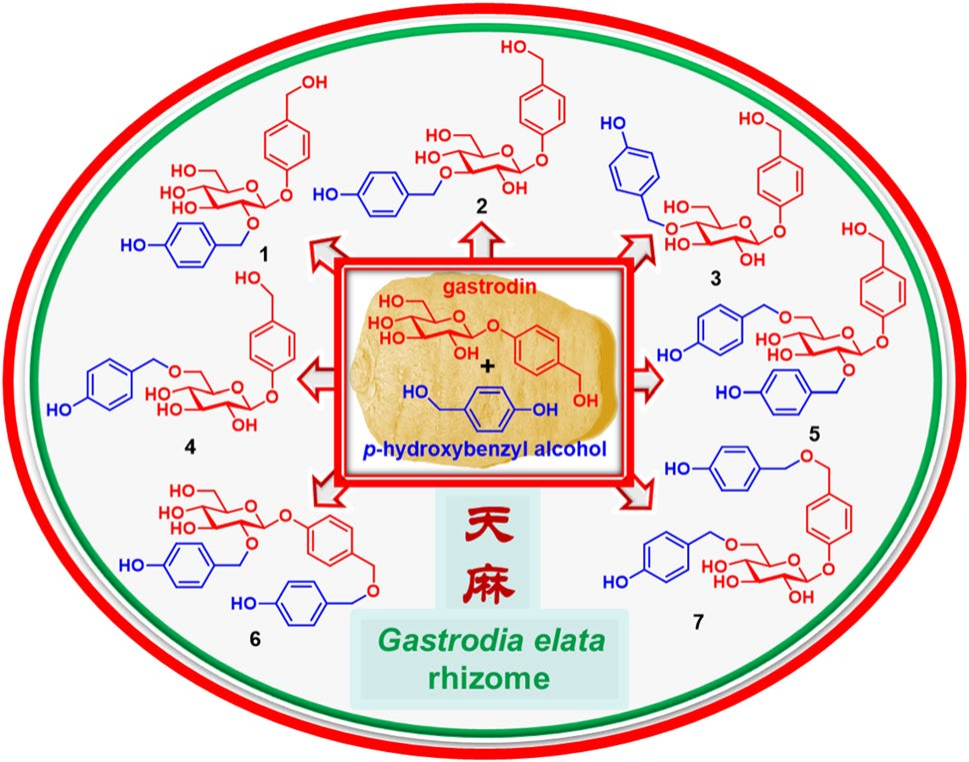

**Electronic supplementary material:**

The online version of this article (10.1007/s13659-019-00224-1) contains supplementary material, which is available to authorized users.

## Introduction

Rhizoma Gastrodiae (“tian ma”) is an important tonic herbal medicine derived from *Gastrodia elata* Blume (Orchidaceae) rhizomes. Its medicinal application for improving health conditions and treating neuralgic and nervous disorders can be traced back to the earliest Chinese pharmacopeia “Shen Nong Ben Cao Jing” [1–3]. Chemical and pharmacological studies have characterized different structural features and biological activities of the constituents of the *G. elata* material extracts, indicating enrichment in bioactive metabolites containing *p*-hydroxybenzyl [4–25]. Although EtOH or MeOH are common protocols used for extracting chemical components from plant material, in most cases herbal medicine is decocted with water, with the aqueous decoctions then applied to treat patients. Undoubtedly, chemical reactions occur during decocting, and the resulting artificial products are highly suspected of playing important roles in the theory of Chinese medicine, although concrete proof is yet to be reported in many cases. Thus, we systematically studied the chemical constituents of an aqueous extract of “tian ma” to unravel the chemical and biological diversity of certain traditional Chinese medicines [26–37]. We previously reported on 51 compounds from the aqueous extract, including seven new *p*-hydroxybenzyl-substituted amino acids, seven new *p*-hydroxybenzyl-substituted glutathione derivatives, one new *N*,*N*-bis(*p*-hydroxybenzyl)-substituted ergothioneine, and one new bis(*p*-hydroxybenzyl)-substituted 9,9′-neolignan with a novel carbon skeleton [38–41]. In addition, at a dosage of 5.0 mg kg^−1^, a fraction containing mainly *p*-glucopyranosyloxybenzyl citrates was found to improve scopolamine and cycloheximide impaired memory in mice [42], with *N*^6^-(*p*-hydroxybenzyl)adenosine isolated as the key sedative constituent of the extract [43, 44] and benzyl tetramer (20C) showing neuroprotective effects and the potential to alleviate several symptoms of Parkinson’s Disease (PD) [45–49]. Herein, we reported on the isolation and structural elucidation of nine new gastrodin derivatives modified at the glucosyl unit (**1**–**9**) and seven known derivatives, along with investigation of the potential production of “tian ma” constituents during decocting.

## Results and Discussion

**Fig. 1:**
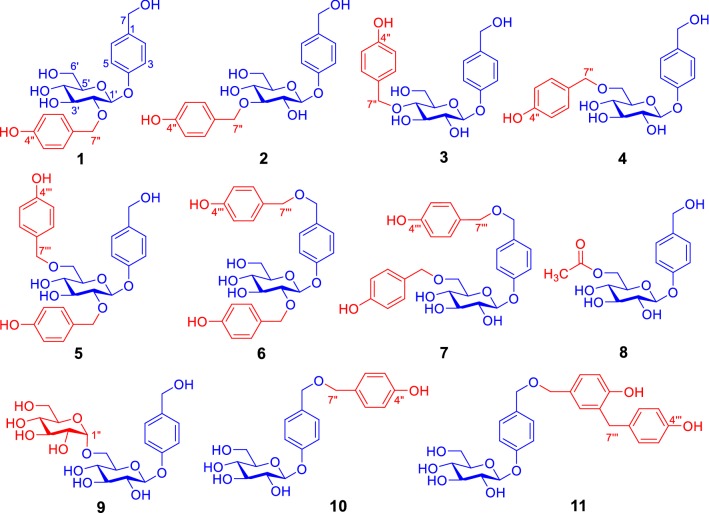
Structures of compounds **1**–**11**

**Fig. 2:**
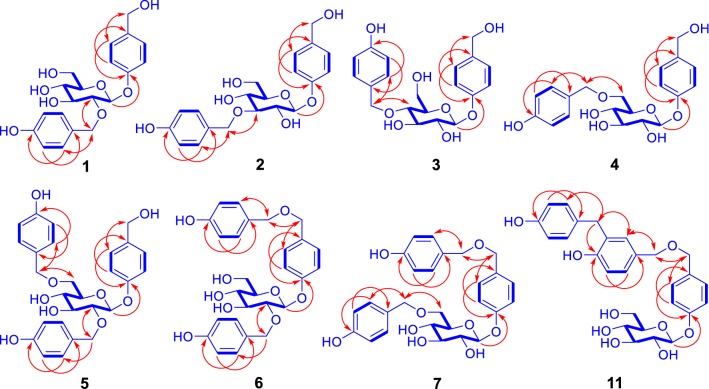
Main ^1^H-^1^H COSY (thick lines) and HMBC (arrows, from ^1^H to ^13^C) correlations of compounds **1**–**7** and **11**

Compound **1** was obtained as a white amorphous powder (Fig. 1). Its infrared (IR) spectrum showed the presence of hydroxy (3354 cm^−1^) and aromatic ring (1612 and 1513 cm^−1^) functionalities. The positive ion electrospray ionization mass spectrometry (ESIMS) of **1** exhibited quasi-molecular ion peaks at *m/z* 415 [M + Na]^+^, 431 [M + K]^+^, and high resolution ESIMS (HRESIMS) at *m/z* 415.1369 indicated the molecular formula of C_20_H_24_O_8_ (calcd for C_20_H_24_O_8_Na [M + Na]^+^, 415.1363). The ^1^H nuclear magnetic resonance (NMR) spectrum of **1** showed typical resonances (Tables 1, 2) due to two *p*-hydroxybenzyl alcohol-derived units at *δ*_H_ 7.24 (2H, d, *J* = 8.5 Hz, H-2/6), 7.15 (2H, d, *J* = 8.0 Hz, H-2″/6″), 6.99 (2H, d, *J* = 8.5 Hz, H-3/5), 6.68 (2H, d, *J* = 8.0 Hz, H-3″/5″), 4.43 (2H, d, *J* = 5.5 Hz, H_2_-7), and 4.72 and 4.65 (1H each, d, *J* = 11.0 Hz, H-7″a and H-7″b). In addition, the spectrum showed *β*-glucopyranosyl with an anomeric proton resonated at *δ*_H_ 4.97 (1H, d, *J* = 7.5 Hz, H-1′) and five carbon-bearing protons resonated between *δ*_H_ 3.19 and 3.70. The presence of the above units was verified by ^13^C NMR and distortionless enhancement by polarization transfer (DEPT) spectroscopic data (Table 2) and further confirmed by 2D NMR data analysis (Fig. 2). In the heteronuclear multiple bond correlation (HMBC) spectrum of **1**, the three-bond heteronuclear correlations of C-4 with H-1′, C-2′ with H_2_-7″, and C-7″ with H-2′ revealed that C-4 of one *p*-hydroxybenzyl alcohol-derived unit was substituted by *β*-glucopyranosyloxy, with C-2′ linked through an ether bond to C-7″ of the other *p*-hydroxybenzyl alcohol-derived unit. Furthermore, PtO_2_-catalyzed hydrogenation of **1** yielded *p*-hydroxybenzyl alcohol and gastrodin, which were identified by comparison of their ^1^H NMR spectroscopic data with those of authentic samples. The gastrodin obtained from the hydrogenation of **1** had $[\alpha ]_{\text{D}}^{20}$ −57.5 (*c* 0.25, MeOH), which was identical to that of the authentic sample $[\alpha ]_{\text{D}}^{20}$−58.0 (*c* 0.25, MeOH). Therefore, the structure of compound **1** was identified as 2′-*O*-(4″-hydroxybenzyl)gastrodin.

**Table 1:** NMR spectral data (*δ*) for compounds **1**–**9**^a^

No.	**1**	**3**	**3**	**4**	**5**	**6**	**7**	**8**	**9**
2/6	7.24 d (8.5)	7.24 d (8.0)	7.21 d (8.5)	7.20 d (8.0)	7.21 d (8.5)	7.12 d (8.5)	7.12 d (8.5)	7.21 d (8.5)	7.23 d (8.5)
3/5	6.99 d (8.5)	6.99 d (8.0)	6.96 d (8.5)	6.98 d (8.0)	7.00 d (8.5)	6.72 d (8.5)	6.72 d (8.5)	6.94 d (8.5)	7.01 d (8.5)
7	4.43 d (5.5)	4.42 d (5.5)	4.41 d (5.5)	4.33 s	4.42 d (5.5)	4.35 s	4.34 s	4.40 d (5.5)	4.40 s
1′	4.97 d (7.5)	4.87 d (7.5)	4.84 d (7.5)	4.85 d (7.5)	4.99 d (7.5)	4.97 d (8.0)	4.88 d (7.5)	4.85 d (7.5)	4.80 d (7.5)
2′	3.24 dd (7.5, 8.5)	3.21 m	3.26 dd (7.5, 8.5)	3.24 dd (7.5, 8.5)	3.24 m	3.22 (1H, m)	3.20 m	3.16 m	3.41 m
3′	3.38 m	3.35 m	3.46 m	3.25 m	3.38 m	3.24 (1H, m)	3.24 m	3.27 m	3.20 m
4′	3.19 m	3.15 m	3.24 m	3.11 m	3.15 m	3.17 (1H, m)	3.11 m	3.21 m	3.08 m
5′	3.32 m	3.30 m	3.37 m	3.52 m	3.55 m	3.31 (1H, m)	3.56 m	3.56 m	3.18 m
6′a	3.70 dd (11.5, 5.5)	3.69 dd (11.5, 5.5)	3.62 dd (11.5, 5.5)	3.71 dd (11.5, 5.5)	3.70 d (10.0)	3.68 dd (10.5, 5.0)	3.69 brd (10.5)	4.25 dd (11.5, 2.0)	3.65dd (11.5, 3.5)
6′b	3.46 dd (11.5, 6.0)	3.48 dd (11.5, 6.0)	3.47 dd (11.5, 6.0)	3.44 dd (11.5, 6.0)	3.45 dd (11.0, 7.0)	3.45 m	3.43 dd (10.5, 6.5)	4.05 dd (11.5, 6.5)	3.53 dd (11.5, 6.5)
1″									4.67 d (3.5)
2″	7.15 d (8.0)	7.21 d (8.0)	7.13 d (8.5)	7.07 d (8.0)	7.14 d (8.0)	7.13 d (8.5)	7.07 d (8.5)	1.99 s	3.41 m
3″	6.68 d (8.0)	6.70 d (8.0)	6.70 d (8.5)	6.69 d (8.0)	6.68 d (9.0)	6.66 d (8.5)	6.68 d (8.5)		3.42 m
4″									3.20 m
5″	6.68 d (8.0)	6.70 d (8.0)	6.70 d (8.5)	6.69 d (8.0)	6.68 d (9.0)	6.66 d (8.5)	6.68 d (8.5)		3.43 m
6″a	7.15 d (8.0)	7.21 d (8.0)	7.13 d (8.5)	7.07 d (8.0)	7.14 d (8.0)	7.13 d (8.5)	7.07 d (8.5)		3.65 m
6″b									3.53 m
7″a	4.72 d (11.0)	4.69 s	4.73 d (11.0)	4.42 s	4.70 d (11.0)	4.70 d (11.0)	4.32 s		
7″b	4.65 d (11.0)		4.44 d (11.0)		4.63 d (11.0)	4.63 d (11.0)			
2′′′/6′′′					7.08 d (8.5)	7.25 d (8.5)	7.22 d (8.5)		
3′′′/5′′′					6.70 d (9.0)	7.00 d (8.5)	7.01 d (8.5)		
7″a					4.34 s	4.39 s	4.39 s		
7-OH	5.07 t (5.5)	5.07 t (5.5)	5.07 t (5.5)		5.08 t (5.5)			5.07 t (5.0)	
2′-OH		5.48 d (4.5)	5.37 d (5.0)	5.31 s			5.31 d (5.0)	5.37 d (5.0)	
3′-OH	5.21 d (5.5)		5.27 d (5.5)	5.10 s	5.20 d (5.5)	5.21 d (5.0)	5.10 d (5.0)	5.27 d (5.0)	
4′-OH	5.08 d (5.0)	5.18 d (4.5)		5.10 s	5.25 d (55)	5.09 d (5.5)	5.10 d (5.0)	5.19 d (5.0)	
6′-OH	4.58 t (5.5)	4.58 t (5.5)				4.58 t (5.5)			
4″-OH	9.30 s	9.28 s	9.33 s		9.33 s	9.38 s	9.35 s		
4″′-OH					9.30 s	9.31 s	9.31 s		

^a^Data (*δ*) were measured in DMSO-*d*_6_ at 500 MHz. Coupling constants (*J*) in Hz are given in parentheses. Assignments were based on DEPT, ^1^H-^1^H COSY, HSQC, and HMBC experiments

**Table 2:** ^13^C NMR spectral data (*δ*) for compounds **1**–**9**

No.	**1**	**2**	**3**	**4**	**5**	**6**	**7**	**8**	**9**
1	136.0	135.9	135.9	135.9	136.1	129.1	128.5	136.0	136.2
2/6	127.8	127.7	127.7	127.6	127.7	129.4	129.3	127.6	128.1
3/5	115.8	116.0	116.0	116.0	115.8	115.7	115.0	115.8	116.6
4	156.1	156.2	156.3	156.2	156.0	156.6	156.8	156.0	156.7
7	62.5	62.5	62.6	62.5	62.5	71.8	72.1	62.4	62.8
1′	100.4	100.5	100.3	100.4	100.2	100.9	100.2	100.2	101.3
2′	81.0	73.1	73.6	73.2	81.0	81.7	73.2	73.2	72.8
3′	76.0	84.6	76.8	76.6	75.9	76.7	76.6	76.3	76.9
4′	70.0	69.3	77.4	70.0	70.2	70.6	69.9	69.9	70.3
5′	76.9	76.9	75.7	75.4	75.2	77.6	75.4	73.6	75.1
6′	60.7	60.5	60.4	69.2	69.2	61.3	69.2	63.4	66.7
1″	129.1	129.7	129.3	128.7	129.1	129.0	128.5	170.2	98.6
2″	129.4	129.2	129.5	129.1	129.4	129.3	129.2	20.6	72.1
3″	114.7	114.6	114.9	114.9	114.7	115.4	114.7		76.9
4″	156.7	156.5	156.8	156.7	156.7	156.8	156.7		70.2
5″	114.7	114.6	114.9	114.9	114.7	115.4	114.7		73.5
6″	129.4	129.2	129.5	129.1	129.4	129.3	129.2		61.0
7″	73.6	73.8	73.6	72.1	73.6	74.3	70.6		
1″′					128.7	132.0	131.8		
2″′/6″′					129.1	129.7	128.9		
3″′/5″′					114.9	116.6	116.0		
4″′					156.7	157.4	156.8		
7″					72.1	71.3	71.1		

Data (*δ*) were measured in DMSO-*d*_6_ at 125 MHz. Assignments were based on DEPT, ^1^H-^1^H COSY, HSQC, and HMBC experiments

**Fig. 3:**
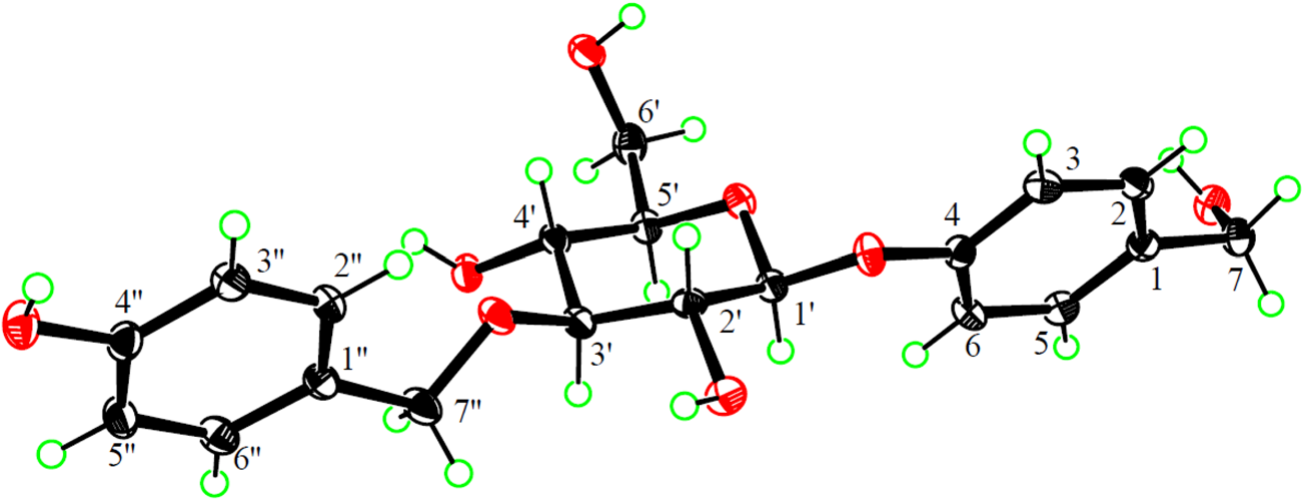
ORTEP diagram of crystal structure of **2**

Compound **2** exhibited spectroscopic data similar to **1**. When comparing the NMR spectroscopic data of the two compounds (Tables 1 and 2), H-1′-H-5′ and C-2′ and C-4′ in **2** were shielded by Δ*δ*_H_ > − 0.02 and Δ*δ*_C_ − 7.9 and − 0.7, respectively, whereas H-2″/6″ and C-3′ were deshielded by +0.06 and Δ*δ*_C_ + 8.6, respectively. In addition, the AB coupling system of H_2_-7″ in **1** was replaced by a singlet at *δ*_H_ 4.69 in **2**. These differences suggested that the 4″-hydroxybenzyl at C-2′ in **1** migrated to C-3′ in **2**, which was confirmed by 2D NMR data analysis (Fig. 2), particularly the HMBC correlations of C-3′ with H_2_-7″. Based on a hydrogenated mixture of **2** with the PtO_2_ catalyst, *p*-hydroxybenzyl alcohol and gastrodin were isolated and identified using the same protocol as described for **1**. Therefore, the structure of compound **2** was determined to be 3′-*O*-(4″-hydroxybenzyl)gastrodin, as confirmed by crystallographic analysis of a suitably crystal grown in a mixed solvent of EtOH−H_2_O (1:1), with the ORTEP diagram of the crystal structure shown in Fig. 3.

The spectroscopic data of **3** indicated that this compound was another isomer of **1** and **2**. Comparison of the NMR spectroscopic data between **3** and **2** (Tables 1 and 2) demonstrated that H-4′ and H-6′b and C-3′ and C-5′ in **3** were shielded by Δ*δ*_H_ − 0.09 and − 0.01 and Δ*δ*_C_ − 7.8 and − 1.2, respectively; in contrast H-3′ and H-5′ and C-2′ and C-4′ were deshielded by Δ*δ*_H_ + 0.11 and + 0.07 and Δ*δ*_C_ + 0.5 and + 8.1, respectively. Moreover, unlike the singlet in **2**, H_2_-7″ appeared as an AB coupling system in **3** at *δ*_H_ 4.73 and 4.44 (each d, *J* = 11.0 Hz). From these differences, in combination with the HMBC correlation from H-4′ to C-7′′ (Fig. 2), the structure of compound **3** was elucidated as 4′-*O*-(4″-hydroxybenzyl)gastrodin, as confirmed by hydrogenation using the aforementioned method.

From spectroscopic data, compound **4** was identified as another isomer of **1**–**3**. Based on the chemical shift changes of C-4′ (Δ*δ*_C_ − 7.4) and C-6′ (Δ*δ*_C_ + 8.8) in **4** as those in **3**, together with the correlations from H_2_-6′ to C-7′′ in the HMBC spectrum (Fig. 2) and production of *p*-hydroxybenzyl alcohol and gastrodin by PtO_2_-catalyzed hydrogenation, the structure of compound **4** was determined to be 6′-*O*-(4″-hydroxybenzyl)gastrodin.

Compound **5** was shown to have the molecular formula C_27_H_30_O_9_ by positive ion HRESIMS at *m/z* 521.1786 (calcd for C_27_H_30_O_9_Na [M + Na]^+^, 521.1782) in combination with NMR spectroscopic data (Tables 1, 2). The NMR spectra of **5** displayed signals ascribable to three inequivalent *p*-hydroxybenzyloxy units and a *β*-glucopyranosyl moiety. This indicated that **5** was a derivative of **1**, **2**, **3**, or **4**, with one more *p*-hydroxybenzyloxy unit. In the HMBC spectrum of **5**, the correlations of H-1′/C-4, H_2_-7′′/C-2′, and H_2_-7′′′/C-6′ (Fig. 2), together with their chemical shifts, revealed that the three *p*-hydroxybenzyloxy units were located at C-1′, C-2′, and C-6′ of *β*-glucopyranosyl, respectively. Therefore, the structure of compound **5** was identified as 2′,6′-di-*O*-(*p*-hydroxybenzyl)gastrodin.

The spectroscopic data of **6** indicated it to be an isomer of **5**. Comparison of the NMR spectroscopic data between **6** and **5** indicated that H-2/6, H-3/5, H-3′, H-5′, C-1, and C-6′ in **6** were shielded by Δ*δ*_H_ − 0.09, − 0.28, − 0.14, and − 0.24 and Δ*δ*_C_ − 7.0 and − 7.9, respectively, whereas C-5′ and C-7 were deshielded by Δ*δ*_C_ + 2.4 and + 9.3, respectively. These results indicated that *p*-hydroxybenzyloxy at C-6′ and hydroxy at C-7 in **5** exchanged their positions in **6**, as verified by the HMBC correlations of H-1′/C-4, H-2′/C-7″, H_2_-7/C-7′′′, and H_2_-7′′′/C-7. Accordingly, the structure of compound **6** was determined as 2′,7-di-*O*-(*p*-hydroxybenzyl)gastrodin.

Based on spectroscopic data, compound **7** was identified as isomer of **5** and **6**. Comparing the NMR spectroscopic data between **7** and **6**, C-6′ in **7** was deshielded by Δ*δ*_C_ +7.9, whereas C-2′ and C-5′ were shielded by Δ*δ*_C_ -8.5 and -2.2, respectively. Based on these differences, together with the correlations of H-1′/C-4, H_2_-6′/C-7″, H-7/C-7′′′, and H-7′′′/C-7 in the HMBC spectrum of **7** (Fig. 2), the structure of compound **7** was classified as 6′,7-di-*O*-(*p*-hydroxybenzyl)gastrodin.

The molecular formula of compound **8** was determined as C_15_H_20_O_8_ by (−)-HRESIMS. Comparing the NMR spectroscopic data between **8** and gastrodin demonstrated the presence of an additional acetyl unit (*δ*_H_ 1.99 and *δ*_C_ 20.6 and 170.2) and deshielded shifts of H_2_-6′ (Δ*δ*_H_ + 0.60 and + 0.62) and C-6′ (Δ*δ*_C_ + 2.8) as well as a shielded shift of C-5′ (Δ*δ*_C_ − 3.3) in **8**. The chemical shift changes accorded with the effects of acetylation on C-6′ of gastrodin, and alkali hydrolysis of **8** yielded gastrodin. Thus, the structure of compound **8** was determined to be 6′-*O*-acetylgastrodin.

The spectroscopic data of **9** were similar to those of 4-[*β*-d-glucopyranosyl-(1→6)-*β*-d-glucopyranosyloxy]benzyl alcohol [50]. However, the ^1^H NMR spectrum of **9** displayed a characteristic anomeric proton signal at *δ* 4.67 (1H, d, *J* = 3.5 Hz, H-1′′), replacing that of the outer *β*-d-glucopyranosyl in the known compound. Based on this, along with liberation of only glucose as sugar from acid hydrolysis, the structure of compound **9** was identified as 4-[*α*-d-glucopyranosyl-(1→6)-*β*-d-glucopyranosyloxy]benzyl alcohol.

By comparing the measured and reported spectroscopic data, the known compounds were identified, respectively, as 4-(*β*-d-glucopyranosyloxy)benzyl 4-hydroxybenzyl ether (**10**) [2b], 4-[*β*-d-glucopyranosyl-(1→6)-*β*-d-glucopyranosyloxy]benzyl alcohol [50], 4-[*α*-d-glucopyranosyl-(1→4)-*β*-d-glucopyranosyloxy]benzyl alcohol [50], 4-[*β*-d-glucopyranosyl-(1→3)-*β*-d-glucopyranosyloxy]benzyl alcohol [50], 4-[*β*-d-glucopyranosyl-(1→4)-*β*-d-glucopyranosyloxy]benzyl alcohol [50], bis-(4-*β*-d-glucopyranosyloxy)benzyl ester [51], and 3-methoy-4- (*β*-d-glucopyranosyloxy)benzyl alcohol [52].

All isolated compounds were evaluated using preliminary in vitro assays, including neuroprotective activity against serum deprivation-induced PC12 cell damage [53], H_2_O_2_- and L-glutamate-induced SK-N-SH cell injury [54, 55], inflammation inhibitory activity against TNF-α production in RAW264.7 cells [56], antioxidant activity against Fe^2+^/cysteine-induced liver microsomal lipid peroxidation [53], cytotoxicity against human cancer cell lines [57], and antiviral activity against HIV-1 replication [57], but inactive at a concentration of 10 μM.

The new isolates (**1**–**9**) were gastrodin derivatives, with one or two additional *p*-hydroxybenzyl groups as well as an acetyl or *α*-glucopyranosyl group at different positions of glucopyranosyl moiety. In particular, glucopyranosyl in **1**–**7** was modified by *p*-hydroxybenzyl, which is unusual. We speculate that, through a reaction of gastrodin alone or between molecules of the structural units, **1**–**7** and **9** were chemically generated during the decocting procedure or biogenetically synthesized during metabolism of the plant. To support this speculation, the following reactions were performed by refluxing water solutions of: (a) gastrodin alone; (b) d-glucose and 4-hydroxybenzyl alcohol; (c) d-glucose and gastrodin; and (d) gastrodin and *p*-hydroxybenzyl alcohol, for 12 h. Both thin layer chromatography (TLC) and UPLC-HRESIMS analyses of the reaction mixtures (see Figs. S94–S99 in Supplementary Material) indicated that reactions (a–c) did not generate **1**–**7** or **9**. Fortunately, **1**–**4**, **7**, **10**, and **11** were detectable by UPLC-HRESIMS and subsequently isolated from the reaction mixture (d). These results indicated that: (a) gastrodin was highly stable in water; (b) d-glucose alone could not react with *p*-hydroxybenzyl alcohol or gastrodin in water; and (c) gastrodin had an unusual property to react with *p*-hydroxybenzyl alcohol. This suggests that the reactivity of d-glucopyranosyl moiety to *p*-hydroxybenzyl alcohol was significantly enhanced in gastrodin compared to d-glucose, which is chemically interesting. In addition, among the reaction products, only **11** was not yet isolated from the extract in this study, with the structure determined only by UPLC/Q-TOF MS analysis in previous literature [58]. The structure of synthetic **11** was identified by comprehensive analysis of spectroscopic data, including 2D NMR experiments (Fig. 2), with the detailed physicochemical properties reported (see Experimental section).

**Fig. 4:**
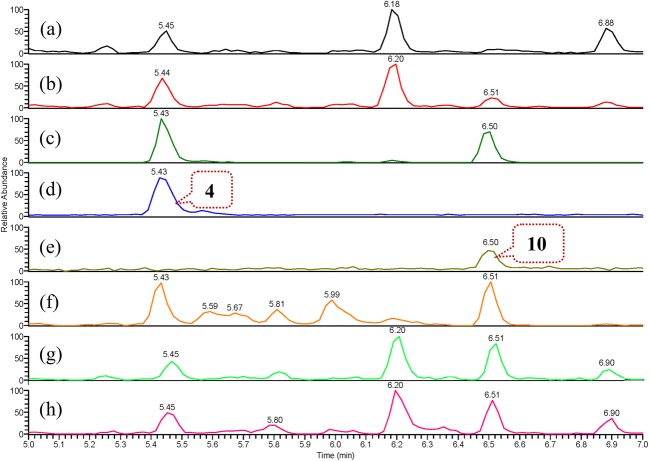
Overlaid regional UPLC-HRESIMS chromatograms of extracted positive ion at *m/z* 415 [M + Na]^+^ from (+)-TIC of: **a** aqueous extract prepared by soaking freeze-dried sample of freshly collected *G. elata* rhizomes at room temperature for 24 h; **b** aqueous extract prepared by refluxing freeze-dried sample of freshly collected *G. elata* rhizomes for 1 h; **c** aqueous extract prepared by refluxing commercially available “tian ma” sample for 1 h; **d** aqueous solution of compound **4**; **e** aqueous solution of compound **10**; **f** ethanol extract prepared by refluxing commercially available “tian ma” sample for 1 h; **g** ethanol extract prepared by refluxing freeze-dried sample of freshly collected *G. elata* rhizomes for 1 h (chromatogram was left adjusted by 0.03 min); **h** ethanol extract prepared by soaking freeze-dried sample of freshly collected *G. elata* rhizomes at room temperature for 24 h (for overlaid full UPLC-HRESIMS chromatograms, see Supplementary Material)

**Fig. 5:**
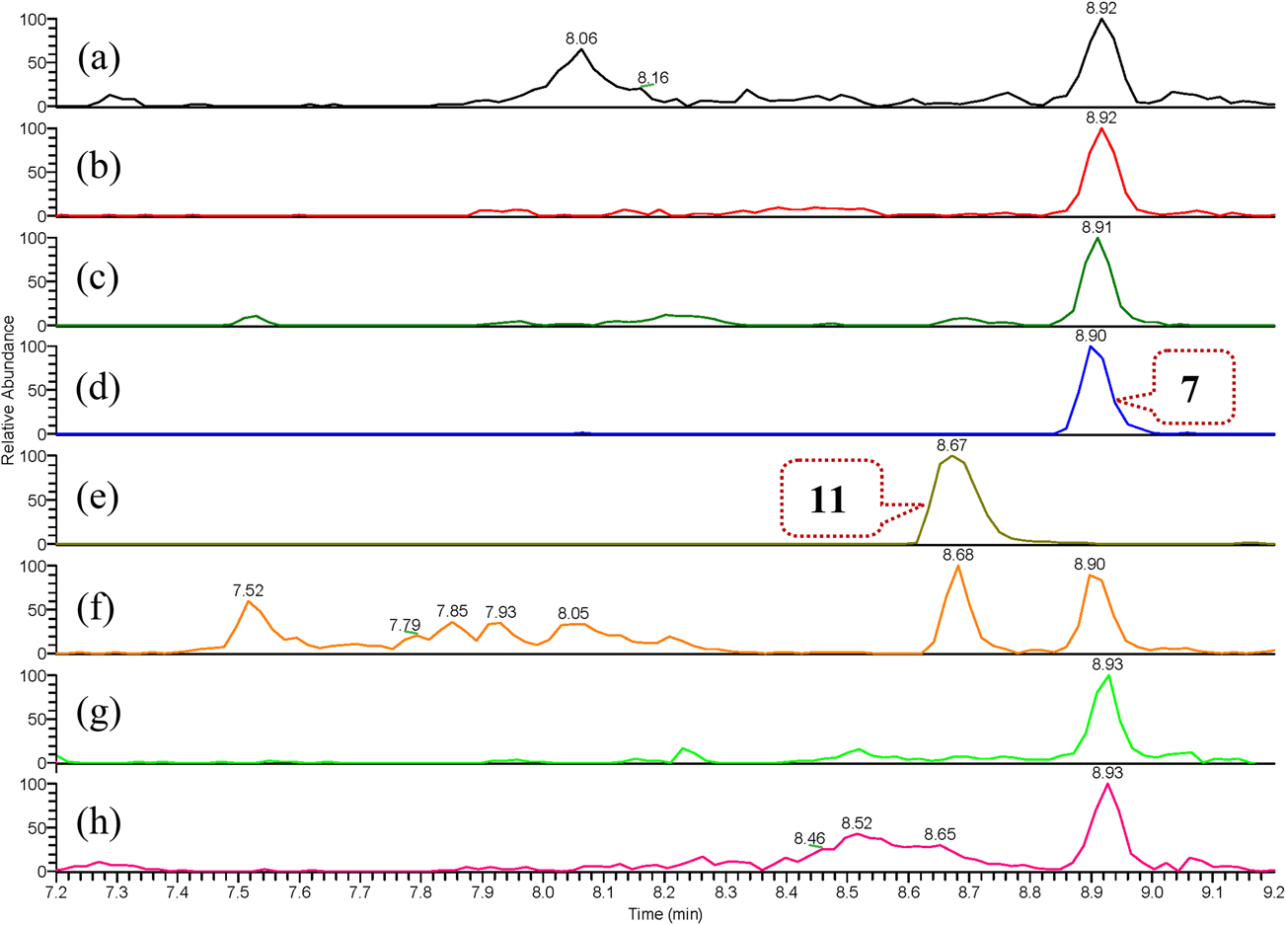
Overlaid regional UPLC-HRESIMS chromatograms of extracted positive ion at *m/z* 521 [M + Na]^+^ from (+)-TIC of: **a** aqueous extract prepared by soaking freeze-dried sample of freshly collected *G. elata* rhizomes at room temperature for 24 h; **b** aqueous extract prepared by refluxing freeze-dried sample of freshly collected *G. elata* rhizomes for 1 h; **c** aqueous extract prepared by refluxing commercially available “tian ma” sample for 1 h; **d** aqueous solution of compound **7**; **e** aqueous solution of compound **11**; **f** ethanol extract prepared by refluxing commercially available “tian ma” sample for 1 h; **g** ethanol extract prepared by refluxing freeze-dried sample of freshly collected *G. elata* rhizomes for 1 h (chromatogram was left adjusted by 0.03 min); **h** ethanol extract prepared by soaking freeze-dried sample of freshly collected *G. elata* rhizomes at room temperature for 24 h (for overlaid full UPLC-HRESIMS chromatograms, see Supplementary Material)

Furthermore, fresh *G. elata* rhizomes were collected in the same field as the initial material and fresh extracts were prepared using EtOH and H_2_O by refluxing and soaking at room temperature, respectively. Subsequent UPLC-HRESIMS analysis demonstrated the existence of **4**, **7**, **10**, and **11** in the extracts and relative higher abundances of **7** in the refluxed extracts compared to those in the extracts prepared at room temperature (see Figs. 4 and 5, and S100–S137 in Supplementary Material). Although we did not detect the presence of these compounds in living plant material without extraction, the above results confidentially indicate that these compounds occur in the decoction of “tian ma” that is ultimately utilized for the treatment of patients.

In conclusion, nine new gastrodin-derived analogues (**1**–**9**), together with seven known derivatives, were isolated from an aqueous extract of “tian ma”, with their structures unambiguously assigned. Results indicated that the *p*-hydroxybenzyl-containing metabolites dominated the chemical constituents of “tian ma”, including the major (*p*-glucopyranosyloxybenzyl citrates and gastrodin) and minor components (**1**–**9**). Due to reactivity of co-occurring *p*-hydroxybenzyl alcohol and its derivatives (e.g., gastrodin and *p*-glucopyranosyloxybenzyl citrates) in the plant, production of “artificial products”, including **1**–**7**, **10**, and **11**, is difficult to avoid during extraction and isolation procedures; however, this does not mean that the plant cannot synthesize these compounds. In most cases, herbal medicines are utilized by formulating and decocting with water, and the higher reactivity of the chemical constituents represents a higher possibility of the formation of some potential effective compounds during decocting. We believe that this may be the real medicinal chemistry behind ancient Chinese traditional medicine and other traditional herbal medicines. Although the known compounds and compounds **1**–**9** were inactive in the current cell-based assays, their potential significance for the clinical application of “tian ma” will be evaluated in other assay systems including animal models.

## Experimental

### General Experimental Procedures

The UV spectra were obtained on a Cary 300 spectrometer (JASCO, Tokyo, Japan) and the IR spectra were recorded on a Nicolet 5700 FT-IR microscope instrument (FT-IR microscope transmission, Thermo Electron Corporation, Madison, WI, USA). Both 1D and 2D NMR spectra were acquired with 500 or 600 MHz for ^1^H and 125 or 150 MHz for ^13^C, respectively, on INOVA 500 MHz or SYS 600 MHz spectrometers (Varian Associates Inc., Palo Alto, CA, USA) in DMSO-*d*_6_, with solvent peaks used as references. ESIMS data were measured with a Q-Trap LC/MS/MS (Turbo Ionspray Source) spectrometer. HRESIMS data were measured using a 6520 Accurate Mass Q-TOF LC/MS spectrometer (Agilent Technologies, Ltd., Santa Clara, CA, USA) or a Q Exactive Focus Mass Spectrometry (Thermo Fisher Scientific, MA, USA). Column chromatography (CC) was performed with macroporous adsorbent resin (HPD-100, Cangzhou Bon Absorber Technology Co. Ltd, Cangzhou, China), MCI gel (CHP 20P, Mitsubishi Chemical Corporation, Japan), silica gel (200–300 mesh, Qingdao Marine Chemical Inc. Qingdao, China), or Pharmadex Sephadex LH-20 (Amersham Biosciences, Inc., Shanghai, China). Preparative TLC separation was performed with high-performance silica gel TLC plates (HSGF_254_, glass precoated, Yantai Jiangyou Silica Gel Development Co., Ltd., Yantai, China). HPLC separation was performed on an instrument consisting of a Waters 600 controller, 600 pump, and 2487 dual λ absorbance detector (Waters Corporation, Milford, MA, USA), with an Prevail (250 × 10 mm i.d.) preparative column packed with C18 (5 μm). TLC was carried out with glass precoated silica gel GF_254_ plates. Spots were visualized under UV light or by spraying with 7% H_2_SO_4_ in 95% EtOH followed by heating. UPLC-HRESIMS analyses of the reaction mixture and freshly prepared extract were performed on an Ultimate 3000 UPLC system equipped with a Q Exactive Focus Mass Spectrometer (Thermo Fisher Scientific, MA, USA) and analyzed by an ACQUITY UPLC^®^ BEH C_18_ column (2.1 × 100 mm, 1.7 μm; Waters, USA), eluting with a gradient of increasing CH_3_CN in H_2_O from 5% to 60% in 15 min (0.4 mL/min, 25 °C).

### Plant Material

The rhizomes of *Gastrodia elata* were collected at the plantation field Xiao Cao Ba in Yunnan Province, China, in December 2009. Plant identification was verified by Mr. Lin Ma (Institute of Materia Medica, Beijing 100050, China). A voucher specimen (No. ID-S-2384) was deposited at the herbarium of the Department of Chemistry of Natural Products, Institute of Materia Medica, Beijing, China. Fresh *G. elata* rhizomes were collected from the same field in July 2018.

### Extraction and Isolation

The steamed and air-dried *G. elata* rhizomes (50 kg) were pulverized and ultrasonicated with H_2_O (150 L, 3 × 1 h). The aqueous extracts were combined and evaporated under reduced pressure to yield a concentrated solution (50 L), which was loaded on a macroporous adsorbent resin (HPD-100, 30 kg) column (20 × 200 cm), and eluted successively with H_2_O (50 L), 30% EtOH (150 L), 50% EtOH (120 L), and 95% EtOH (80 L) to yield four corresponding fractions A−D. After removing the solvent under reduced pressure, fraction C (1.9 kg) was chromatographed over MCI gel (CHP 20P, 75–150 μm, 10 L), with successive elution using H_2_O (30 L), 30% EtOH (70 L), 50% EtOH (70 L), 95% EtOH (30 L), and Me_2_CO (20 L), to obtain fractions C1−C5. Fraction C1 (66 g) was separated by CC over Sephadex LH-20, with successive elution using H_2_O, 30% EtOH, 50% EtOH, and 95% EtOH to give subfractions C1-1–C1-4. Further fractionation of C1-2 (36 g) by reverse-phase medium-pressure liquid chromatography (RP-MPLC, ODS, 50 μm, YMC, Co. LTD, Japan), with elution using a gradient of increasing MeOH (0-100%) in H_2_O, yielded subfractions C1-2-1–C1-2-4. Subfraction C1-2-2 (2.5 g) was separated by RP-HPLC (ODS, 5 μm, YMC), followed by elution with 28% MeOH to obtain **8** (*t*_R_ = 34.5 min, 15.0 mg). Subfraction C1-2-3 (9.3 g) was chromatographed over silica gel, followed by elution with a gradient of increasing MeOH (0–100%) in CHCl_3_, to yield C1-2-3-1-C1-2-3-5. Fraction C1-2-3-3 (0.61 g) was isolated by CC over Sephadex LH-20 (50% MeOH) to give C1-2-3-3-1 (0.42 g), which was further fractionated by preparative TLC (CHCl_3_-MeOH, 15:1) to obtain C1-2-3-3-1-1. Separation of C1-2-3-3-1-1 (0.34 g) by RP-HPLC (45% MeOH) yielded **4** (*t*_R_ = 19.8 min, 18.3 mg), **3** (*t*_R_ = 21.7 min, 18.9 mg), **2** (*t*_R_ = 25.2 min, 35.1 mg), and **1** (*t*_R_ = 27.5 min, 59.3 mg). Further fractionation of C1-3 (36 g) by CC over Sephadex LH-20 (50% MeOH) gave C1-3-1–C1-3-4, of which C1-3-3 (2.3 g) was isolated by CC over Sephadex LH-20 (MeOH) to obtain C1-3-3-1–C1-3-3-3. Isolation of C1-3-3-2 (0.96 g) by RP-HPLC (ODS, 5 μm, YMC, 35% MeOH) yielded **7** (*t*_R_ = 40.5 min, 22.8 mg), **6** (*t*_R_ = 41.8 min, 38.6 mg) and **5** (*t*_R_ = 43.6 min, 52.9 mg). Subfraction C3 (120 g) was chromatographed over silica gel, followed by elution with EtOAc-MeOH (1:0-0:1), to give C3-1−C3-7, of which C3-3 (6 g) was separated by CC over Sephadex LH-20 (50% MeOH in H_2_O) to yield subfractions C3-3-1−C3-3-4. Isolation of C3-3-3 (280 mg) by RP-HPLC (ODS, 5 μm, YMC, 22% MeOH) yielded **9** (*t*_R_ = 21.5 min, 25.6 mg).

2′-*O*-(4″-Hydroxybenzyl)gastrodin (**1**): white amorphous powder (MeOH); $[\alpha ]_{\text{D}}^{20}$ – 42.8 (*c* 0.50, MeOH); UV (MeOH) *λ*_max_ (log *ε*) 203 (3.83), 222 (3.52) nm, 274 (2.37) nm; IR *ν*_max_ 3354, 2919, 2881, 1612, 1513, 1452, 1389, 1364, 1305, 1234, 1172, 1072, 1056, 898, 829, 779, 659, 628, 582, 567, 505 cm^−1^; ^1^H NMR (DMSO-*d*_6_, 500 MHz) and ^13^C NMR (DMSO-*d*_6_, 125 MHz) data, see Tables 1 and 2; (+)-ESIMS *m/z* 415 [M + Na]^+^, 431 [M + K]^+^; (+)-HRESIMS *m/z* 415.1369 (calcd for C_20_H_24_O_8_Na [M + Na]^+^, 415.1363).

3′-*O*-(4″-Hydroxybenzyl)gastrodin (**2**): colorless prism; m.p. 176-178 °C; $[\alpha ]_{\text{D}}^{20}$-44.3 (*c* 0.50, MeOH); UV (MeOH) *λ*_max_ (log *ε*) 208 (3.79), 270 (2.35) nm; IR *ν*_max_ 3440, 3401, 3170, 3031, 2937, 2890, 2849, 1893, 1683, 1612, 1512, 1467, 1414, 1365, 1307, 1256, 1229, 1176, 1130, 1114, 1082, 1043, 1024, 995, 952, 896, 851, 825, 776, 724, 694, 663, 632, 616, 591, 573, 524 cm^−1^; ^1^H NMR (DMSO-*d*_6_, 500 MHz) and ^13^C NMR (DMSO-*d*_6_, 125 MHz) data, see Tables 1 and 2; (−)-ESIMS *m/z* 391 [M - H]^-^, 427 [M + Cl]^-^; (+)-HRESIMS *m/z* 415.1360 (calcd for C_20_H_24_O_8_Na [M + Na]^+^, 415.1363).

X-ray crystallography of **2**: Crystal data of **2** was collected on a Xcalibur, Atlas, Gemini ultra-diffractometer (Agilent Technologies, Ltd., Santa Clara, CA, USA) with Cu Kα radiation using the *ω* scan technique. Using Olex2 [59], the structure was solved with the ShelXS [60] structure solution program using Direct Methods and refined with the ShelXL [61] refinement package using Least Squares minimization. For crystal data and structure refinement parameters of **2**, see Table S1.

Crystallographic data for the structure of **2** (CCDC 1955120) were deposited at the Cambridge Crystallographic Data Centre as a supplementary publication. These data can be obtained free of charge via www.ccdc.cam.ac.uk/conts/retrieving.html (or from the Cambridge Crystallographic Data Centre, 12 Union Road, Cambridge CB21EZ, UK; fax: (+44) 1223-336-033; or deposit@ccdc.cam.ac.uk).

4′-*O*-(4″-Hydroxybenzyl)gastrodin (**3**): white amorphous powder (MeOH); -48.2 (*c* 0.50, MeOH); UV (MeOH) *λ*_max_ (log *ε*) 205 (3.71), 219 (3.58) nm, 273 (2.31); IR *ν*_max_ 3357, 2919, 2883, 1678, 1612, 1513, 1450, 1399, 1366, 1232, 1137, 1099, 1077, 1048, 935, 896, 832, 779, 725, 655, 628, 547 cm^−1^; ^1^H NMR (DMSO-*d*_6_, 500 MHz) and ^13^C NMR (DMSO-*d*_6_, 125 MHz) data, see Tables 1 and 2; (-)-ESIMS *m/z* 391 [M – H]^-^, 427 [M + Cl]^-^; (+)-HRESIMS *m/z* 415.1362 (calcd for C_20_H_24_O_8_Na [M + Na]^+^, 415.1363).

6′-*O*-(4″-Hydroxybenzyl)gastrodin (**4**): white amorphous powder (MeOH); $[\alpha ]_{\text{D}}^{20}$ − 46.6 (*c* 0.50, MeOH); UV (MeOH) *λ*_max_ (log *ε*) 203 (3.87), 222 (3.66), 274 (2.32) nm; IR *ν*_max_ 3354, 2915, 2875, 1892, 1612, 1513, 1448, 1423, 1367, 1316, 1231, 1173, 1065, 1009, 950, 875, 829, 775, 727, 710, 693, 659, 629, 601, 562 cm^−1^; ^1^H NMR (DMSO-*d*_6_, 500 MHz) and ^13^C NMR (DMSO-*d*_6_, 125 MHz) data, see Tables 1 and 2; (+)-ESIMS *m/z* 415 [M + Na]^+^; (+)-HRESIMS *m/z* 415.1375 (calcd for C_20_H_24_O_8_Na [M + Na]^+^, 415.1363).

2′,6′-Di-*O*-(*p*-hydroxybenzyl)gastrodin (**5**): white amorphous powder (MeOH); $[\alpha ]_{\text{D}}^{20}$ – 62.1 (*c* 0.50, MeOH); UV (MeOH) *λ*_max_ (log *ε*) 222 (4.17), 274 (3.19) nm; IR *ν*_max_ 3354, 2923, 2877, 1677, 1613, 1514, 1447, 1366, 1315, 1229, 1209, 1141, 1060, 830, 802, 778, 723, 662, 631, 573, 510 cm^−1^; ^1^H NMR (DMSO-*d*_6_, 500 MHz) and ^13^C NMR (DMSO-*d*_6_, 125 MHz) data, see Tables 1 and 2; (−)-ESIMS *m/z* 533 [M + Cl]^-^; (+)-HRESIMS *m/z* 521.1786 (calcd for C_27_H_30_O_9_Na [M + Na]^+^, 521.1782).

2′,7-Di-*O*-(*p*-hydroxybenzyl)gastrodin (**6**): white amorphous powder (MeOH); $[\alpha ]_{\text{D}}^{20}$ − 63.5 (*c* 0.55, MeOH); UV (MeOH) *λ*_max_ (log *ε*) 202 (4.09), 226 (3.76), 275 (2.35) nm; IR *ν*_max_ 3351, 2915, 2875, 1890, 1678, 1613, 1514, 1446, 1365, 1206, 1139, 1060, 830, 801, 777, 722, 661, 630, 600, 515 cm^−1^; ^1^H NMR (DMSO-*d*_6_, 600 MHz) and ^13^C NMR (DMSO-*d*_6_, 125 MHz) data, see Tables 1 and 2; (−)-ESIMS *m/z* 497 [M – H]^-^; (+)-HRESIMS *m/z* 521.1785 (calcd for C_27_H_30_O_9_Na [M + Na]^+^, 521.1782).

6′,7-Di-*O*-(*p*-hydroxybenzyl)gastrodin (**7**): white amorphous powder (MeOH); $[\alpha ]_{\text{D}}^{20}$ − 58.8 (*c* 0.50, MeOH); UV (MeOH) *λ*_max_ (log *ε*) 202 (4.02), 226 (3.87), 275 (2.79) nm; IR *ν*_max_ 3560, 3356, 2927, 2851, 2730, 1893, 1613, 1595, 1514, 1445, 1404, 1367, 1312, 1241, 1173, 1074, 1018, 955, 925, 897, 855, 834, 793, 768, 698, 657, 632, 586, 534, 500, 470 cm^−1^; ^1^H NMR (DMSO-*d*_6_, 500 MHz) and ^13^C NMR (DMSO-*d*_6_, 125 MHz) data, see Tables 1 and 2; (+)-ESIMS *m/z* 521 [M + Na]^+^; (−)-ESIMS *m/z* 497 [M − H]^-^, 533 [M + Cl]^-^, (+)-HRESIMS *m/z* 521.1797 (calcd for C_27_H_30_O_9_Na [M + Na]^+^, 521.1782).

6′-*O*-Acetylgastrodin (**8**): white amorphous powder (MeOH), $[\alpha ]_{\text{D}}^{20}$ − 38.3 (*c* 0.50, MeOH); UV (MeOH) *λ*_max_ (log *ε*) 220 (3.59), 272 (2.41) nm; IR *ν*_max_ 3447, 2923, 2886, 1895, 1734, 1650, 1611, 1590, 1512, 1411, 1362, 1272, 1243, 1180, 1102, 1074, 1041, 1018, 941, 925, 825, 780, 653, 618, 571, 538, 512, 475 cm^−1^; ^1^H NMR (DMSO-*d*_6_, 500 MHz) and ^13^C NMR (DMSO-*d*_6_, 125 MHz) data, see Tables 1 and 2; (+)-ESIMS *m/z* 351 [M + Na]^+^; (−)-ESIMS *m/z* 363 [M + Cl]^-^, 655 [2M − H]^−^; (+)-HRESIMS *m/z* 351.1056 (calcd for C_15_H_20_O_8_Na [M + Na]^+^, 351.1050).

4-*α*-d-Glucopyranosyl-(1→6)-*β*-d-glucopyranosyloxybenzyl alcohol (**9**): white amorphous powder (MeOH), $[\alpha ]_{\text{D}}^{20}$+21.4 (*c* 0.59, MeOH); UV (MeOH) *λ*_max_ (log *ε*) 221 (4.08), 270 (3.08) nm; ^1^H NMR (DMSO-*d*_6_, 500 MHz) and ^13^C NMR (DMSO-*d*_6_, 125 MHz) data, see Tables 1 and 2; (+)-ESIMS *m/z* 471 [M + Na]^+^, 487 [M + K]^+^; (−)-ESIMS *m/z* 447 [M – H]^-^, 483 [M + Cl]^−^; (+)-HRESIMS *m/z* 471.1486 (calcd for C_19_H_28_O_12_Na [M + Na]^+^, 471.1473); 487.1218 (calcd for C_19_H_28_O_12_K [M + K]^+^, 487.1212).

### Hydrogenation of **1–5**

Compounds **1**–**5** (3 mg each) and Pd/C (6 mg) were separately dissolved in MeOH (5 mL) and hydrogenated for 12 h, then filtered and evaporated under reduced pressure. The residue was isolated by preparative thin layer chromatography (PTLC, CH_2_Cl_2_-CH_3_OH, 3:1) to yield a product (1.4–2.0 mg) retention factor (*R*_f_ ~ 0.6) (TLC, CH_2_Cl_2_–MeOH, 3:1), retention time (*t*_R_ ~6.1 min) (HPLC, Grace C_18_ column, 5 μm, 38% MeOH in H_2_O containing 1% HOAc, 220 nm, 2.0 mL/min), and $[\alpha ]_{\text{D}}^{20}$ − 53.5–59.4 (*c* 0.21–0.34, CH_3_OH), which were consistent with those of authentic gastrodin.

### Hydrogenation and Enzymatic Hydrolysis of 6 and 7

Compounds **5** or **6** (5 mg each) and Pd/C (10 mg) were separately dissolved in MeOH (5 mL) and hydrogenated for 12 h, then filtered and evaporated under reduced pressure. The residues were separately hydrolyzed in H_2_O (5 mL) with *β*-glucosidase (5.0 mg) at 37 °C for 36 h, then extracted with EtOAc (3 × 5 mL). The aqueous phase was dried by N_2_ and chromatographed over silica gel, followed by elution with CH_3_CN–H_2_O (8:1) to yield a sugar (1.1 and 1.3 mg from **5** and **6**, respectively) with retention factor (*R*_f_ ~0.2) (TLC, CHCl_3_-MeOH-HOAc-H_2_O 7:3:2:1), $[\alpha ]_{\text{D}}^{20}$ +35.8 (*c* 0.11, H_2_O) and +38.6 (*c* 0.13, H_2_O), which were consistent with those of authentic d-glucose.

### Alkali Hydrolysis of 8

Compound **8** (5 mg) was dissolved in 3% NaOH (5 mL) and stirred for 6 h, then neutralized with 2 N HCl and evaporated under reduced pressure. The residue was isolated by PTLC (CH_2_Cl_2_-CH_3_OH 3:1) to yield a product (3.9 mg) with retention factor (*R*_f_ ~ 0.6) (TLC, CH_2_Cl_2_–MeOH, 3:1), retention time (*t*_R_ ~6.1 min) (HPLC, Grace C_18_ column, 5 μm, 38% MeOH in H_2_O containing 1% HOAc, 220 nm, 2.0 mL/min), and $[\alpha ]_{\text{D}}^{20}$ − 57.6 (*c* 0.26, CH_3_OH) consistent with those of authentic gastrodin.

### Acid hydrolysis of 9

Compound **9** (2.5 mg) was dissolved in 2 N HCl (3 mL) and stirred for 6 h, then neutralized with 2 N NaOH and extracted with EtOAc (3 × 5 mL). The aqueous phase was evaporated under reduced pressure, and chromatographed over silica gel, followed by elution with CH_3_CN–H_2_O (8:1) to yield a sugar (0.8 mg) with retention factor (*R*_f_ ~0.2) (TLC, CHCl_3_-MeOH-HOAc-H_2_O 7:3:2:1) and $[\alpha ]_{\text{D}}^{20}$ + 37.2 (*c* 0.08, H_2_O), consistent with those of authentic d-glucose. 

### Reaction of Gastrodin with 4-Hydroxybenzyl Alcohol

**Scheme 1:**
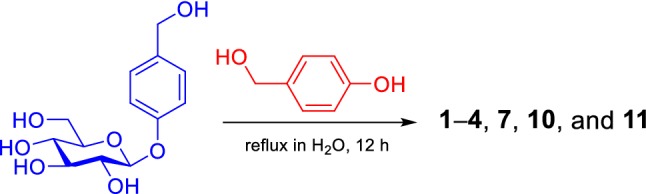
Reaction of gastrodin with *p*-hydroxybenzyl alcohol

Gastrodin (8.58 g) and 4-hydroxybenzyl alcohol (3.72 g) were refluxed in water (500 mL) for 5 days, and the mixture was concentrated under reduced pressure. The residue was chromatographed over reversed phase silica gel (C_18_, 300 g), with gradient elution increasing MeCN in H_2_O (0−100%, *V*/*V*) to obtain subfractions Fr.1-1−Fr.1-8 based on TLC analysis. Fraction Fr.1-3 (2.1 g) was further separated by CC over reversed phase silica gel (C_18_, 30 g) with 10% MeCN in H_2_O to yield Fr.1-3-1−Fr.1-3-3. Isolation of Fr.1-3-1 (350 mg) by PTLC (CH_2_Cl_2_-MeOH 5:1) gave Fr.1-3-1-1−Fr.1-3-1-4, with Fr.1-3-1-2 (50 mg) and Fr.1-3-1-3 (20 mg) separately purified by RP-HPLC using a Grace C_18_ column (28% MeOH in H_2_O, 2.0 mL/min) to yield **2** (7.1 mg) and **3** (8.3 mg). PTLC isolation (CH_2_Cl_2_-MeOH 5:1) of Fr.1-3-2 (15 mg) and Fr.1-3-3 (210 mg) yielded **1** (5.5 mg) and **4** (91.1 mg), respectively. Separation of Fr.1-4 (360 mg) gave subfractions Fr.1-4-1-Fr.1-4-3, with Fr.1-4-2 (270 mg) further isolated by PTLC (CH_2_Cl_2_–MeOH 5:1) to obtain **10** (140.2 mg) and **11** (13.4 mg). PTLC purification (CH_2_Cl_2_–MeOH 5:1) of Fr.1-4-3 (4 mg) yielded **7** (2.0 mg). The spectroscopic data of synthetic **1**–**4**, **7**, and **10** were identical to those of the natural products. The structure of **11** was elucidated only from HRESIMS analysis in previous literature [58], with detailed physicochemical properties presented herein: white amorphous powder (MeOH), $[\alpha ]_{\text{D}}^{20}$ − 24.5 (*c* 0.10, MeOH); UV (MeOH) *λ*_max_ (log *ε*) 225 (3.24), 278 (2.56) nm; ^1^H NMR (DMSO-*d*_6_, 600 MHz): *δ* 9.47 (1H, s, 4′′-O*H*), 9.21 (1H, s, 4′′′-O*H*), 7.18 (2H, d, *J* = 9.0 Hz, H-2/6), 6.99 (2H, d, *J* = 9.0 Hz, H-2′′′/6′′′), 6.98 (2H, d, *J* = 9.0 Hz, H-3/5), 6.94 (1H, dd, *J* = 8.4, 1.8 Hz, H-6′′), 6.93 (1H, d, *J* = 1.8 Hz, H-2′′), 6.80 (1H, d, *J* = 8.4 Hz, H-5′′), 6.66 (2H, d, *J* = 9.0 Hz, H-3′′′/5′′′), 5.31 (1H, d, *J* = 4.8 Hz, 2′-O*H*), 5.15 (1H, d, *J* = 4.2 Hz, 3′-O*H*), 5.10 (1H, d, *J* = 5.4 Hz, H-1′), 4.85 (1H, d, *J* = 4.2 Hz, 4′-O*H*), 4.61 (1H, t, *J* = 4.2 Hz, 6′-O*H*), 4.34 (2H, s, H-7), 4.28 (2H, s, H-7′′), 3.72 (2H, s, H-7′′′), 3.67 (1H, m, H-6′a), 3.47 (1H, m, H-6′b), 3.31 (1H, m, H-4′), 3.28 (1H, m, H-3′), 3.23 (1H, m, H-2′), 3.17 (1H, m, H-5′);^13^C NMR (150 MHz, DMSO-*d*_6_) *δ* 156.8 (C-4), 155.3 (C-4′′′), 154.4 (C-4′′), 131.7 (C-1), 131.2 (C-1′′′), 129.9 (C-2′′), 129.6 (C-2′′′/6′′′), 129.0 (C-2/6), 128.4 (C-1′′), 128.0 (C-3′′), 126.7 (C-6′′), 116.0 (C-3/5), 115.0 (C-3′′′/5′′′), 114.7 (C-5′), 100.4 (C-1′), 77.0 (C-4′), 76.6 (C-3′), 73.3 (C-2′), 71.1 (C-7′′), 70.4 (C-7), 69.7 (C-5′), 60.6 (C-6′), 34.3 (C-7′′′); (+)-HRESIMS *m/z* 521.17822 (calcd for C_27_H_30_O_9_Na [M + Na]^+^, 521.17820) (Scheme 1).

## Electronic supplementary material

Below is the link to the electronic supplementary material.

Supplementary material 1 (PDF 15,350 kb)
