# Supplementary material for: Gastrodin Derivatives from Gastrodia elata
Source: Nat Prod Bioprospect. 2019 Nov 16;9(6):393–404. doi: 10.1007/s13659-019-00224-1 (PMC6872707; doi:10.1007/s13659-019-00224-1)
Supplement: Supplementary file 1 — Supplementary material 1 (PDF 15,350 kb) [file 13659_2019_224_MOESM1_ESM.pdf]

Gastrodin derivatives from *Gastrodia elata*

Cheng-Bo Xu, Qing-Lan Guo, Ya-Nan Wang, Sheng Lin, Cheng-Gen Zhu, and Jian-Gong Shi*

State Key Laboratory of Bioactive Substance and Function of Natural Medicines, Institute of Materia Medica, Chinese Academy of Medical Sciences and Peking Union Medical College, Beijing 100050, People's Republic of China

Supplementary Material

* Corresponding author.

Tel.: 86-10-63025166

Fax: 86-10-63017757

E-mail: shijg@imm.ac.cn (Jian-Gong Shi).

We dedicate this paper to Prof. Sun Han-Dong on the occasion chromatogram of his 80th birthday.

List of Content

no.	Content	Page
1	**Table S1**. Crystal data and structure refinement for **2**	S10
2	**Fig. S1.** Crystal cell diagram for compound **2**	S10
3	**Fig. S2**. The UV spectrum of compound **1**	S11
4	**Fig. S3**. The IR spectrum of compound **1**	S12
5	**Fig. S4**. The ESIMS of compound **1**	S13
6	**Fig. S5**. The (+)-HRESIMS report of compound **1**, page 1	S14
7	**Fig. S6**. The (+)-HRESIMS report of compound **1**, page 2	S15
8	**Fig. S7**. The ^{1}H NMR spectrum of compound **1** in DMSO-d_6 at 500 MHz	S16
9	**Fig. S8**. The ^{13}C NMR spectrum of compound **1** in DMSO-d_6 at 125 MHz	S17
10	**Fig. S9**. The DEPT spectrum of compound **1** in DMSO-d_6 at 125 MHz	S18
11	**Fig. S10**. The ^{1}H-^{1}H COSY spectrum of compound **1** in DMSO-d_6 at 500 MHz	S19
12	**Fig. S11**. The HSQC spectrum of compound **1** in DMSO-d_6 (500 MHz for ^{1}H)	S20
13	**Fig. S12**. The HMBC spectrum of compound **1** in DMSO-d_6 (500 MHz for ^{1}H)	S21
14	**Fig. S13**. The UV spectrum of compound **2**	S22
15	**Fig. S14**. The IR spectrum of compound **2**	S23
16	**Fig. S15**. The ESIMS of compound **2**	S24
17	**Fig. S16**. The (+)-HRESIMS report of compound **2**, page 1	S25
18	**Fig. S17**. The (+)-HRESIMS report of compound **2**, page 2	S26
19	**Fig. S18**. The ^{1}H NMR spectrum of compound **2** in DMSO-d_6 at 500 MHz	S27
20	**Fig. S19**. The ^{13}C NMR spectrum of compound **2** in DMSO-d_6 at 125 MHz	S28
21	**Fig. S20**. The DEPT spectrum of compound **2** in DMSO-d_6 at 125 MHz	S29
22	**Fig. S21**. The ^{1}H-^{1}H COSY spectrum of compound **2** in DMSO-d_6 at 500 MHz	S30
23	**Fig. S22**. The HMQC spectrum of compound **2** in DMSO-d_6 (500 MHz for ^{1}H)	S31
24	**Fig. S23**. The HMBC spectrum of compound **2** in DMSO-d_6 (500 MHz for ^{1}H)	S32
25	**Fig. S24**. The UV spectrum of compound **3**	S33
26	**Fig. S25**. The IR spectrum of compound **3**	S34
27	**Fig. S26**. The ESIMS of compound **3**	S35
28	**Fig. S27**. The (+)-HRESIMS report of compound **3**, page 1	S36
29	**Fig. S28**. The (+)-HRESIMS report of compound **3**, page 2	S37
30	**Fig. S29**. The ^{1}H NMR spectrum of compound **3** in DMSO-d_6 at 500 MHz	S38
31	**Fig. S30**. The ^{13}C NMR spectrum of compound **3** in DMSO-d_6 at 125 MHz	S39
32	**Fig. S31**. The DEPT spectrum of compound **3** in DMSO-d_6 at 125 MHz	S40
33	**Fig. S32**. The ^{1}H-^{1}H COSY spectrum of compound **3** in DMSO-d_6 at 500 MHz	S41
34	**Fig. S33**. The HSQC spectrum of compound **3** in DMSO-d_6 (500 MHz for ^{1}H)	S42
35	**Fig. S34**. The HMBC spectrum of compound **3** in DMSO-d_6 (500 MHz for ^{1}H)	S43
36	**Fig. S35**. The UV spectrum of compound **4**	S44
37	**Fig. S36**. The IR spectrum of compound **4**	S45
38	**Fig. S37**. The ESIMS of compound **4**	S46
39	**Fig. S38**. The (+)-HRESIMS report of compound **4**, page 1	S47
40	**Fig. S39**. The (+)-HRESIMS report of compound **4**, page 2	S48
41	**Fig. S40**. The (+)-HRESIMS report of compound **4**, page 3	S49
42	**Fig. S41**. The ^{1}H NMR spectrum of compound **4** in DMSO-d_6 at 500 MHz	S50

43	**Fig. S42**. The ^{13}C NMR spectrum of compound **4** in DMSO-d_6 at 125 MHz	S51
44	**Fig. S43**. The DEPT spectrum of compound **4** in DMSO-d_6 at 125 MHz	S52
45	**Fig. S44**. The ^{1}H-^{1}H COSY spectrum of compound **4** in DMSO-d_6 at 500 MHz	S53
46	**Fig. S45**. The HSQC spectrum of compound **4** in DMSO-d_6 (500 MHz for ^{1}H)	S54
47	**Fig. S46**. The HMBC spectrum of compound **4** in DMSO-d_6 (500 MHz for ^{1}H)	S55
48	**Fig. S47**. The UV spectrum of compound **5**	S56
49	**Fig. S48**. The IR spectrum of compound **5**	S57
50	**Fig. S49**. The ESIMS of compound **5**	S58
51	**Fig. S50**. The (+)-HRESIMS report of compound **5**, page 1	S59
52	**Fig. S51**. The (+)-HRESIMS report of compound **5**, page 2	S60
53	**Fig. S52**. The ^{1}H NMR spectrum of compound **5** in DMSO-d_6 at 500 MHz	S61
54	**Fig. S53**. The ^{13}C NMR spectrum of compound **5** in DMSO-d_6 at 125 MHz	S62
55	**Fig. S54**. The DEPT spectrum of compound **5** in DMSO-d_6 at 125 MHz	S63
56	**Fig. S55**. The ^{1}H-^{1}H COSY spectrum of compound **5** in DMSO-d_6 at 500 MHz	S64
57	**Fig. S56**. The HSQC spectrum of compound **5** in DMSO-d_6 (500 MHz for ^{1}H)	S65
58	**Fig. S57**. The HMBC spectrum of compound **5** in DMSO-d_6 (500 MHz for ^{1}H)	S66
59	**Fig. S58**. The UV spectrum of compound **6**	S67
60	**Fig. S59**. The IR spectrum of compound **6**	S68
61	**Fig. S60**. The ESIMS of compound **6**	S69
62	**Fig. S61**. The (+)-HRESIMS report of compound **6**, page 1	S70
63	**Fig. S62**. The (+)-HRESIMS report of compound **6**, page 2	S71
64	**Fig. S63**. The ^{1}H NMR spectrum of compound **6** in DMSO-d_6 at 500 MHz	S72
65	**Fig. S64**. The ^{13}C NMR spectrum of compound **6** in DMSO-d_6 at 125 MHz	S73
66	**Fig. S65**. The DEPT spectrum of compound **6** in DMSO-d_6 at 125 MHz	S74
67	**Fig. S66**. The ^{1}H-^{1}H COSY spectrum of compound **6** in DMSO-d_6 at 500 MHz	S75
68	**Fig. S67**. The HSQC spectrum of compound **6** in DMSO-d_6 (500 MHz for ^{1}H)	S76
69	**Fig. S68**. The HMBC spectrum of compound **6** in DMSO-d_6 (500 MHz for ^{1}H)	S77
70	**Fig. S69**. The UV spectrum of compound **7**	S78
71	**Fig. S70**. The IR spectrum of compound **7**	S79
72	**Fig. S71**. The ESIMS of compound **7**	S80
73	**Fig. S72**. The (+)-HRESIMS report of compound **7**, page 1	S81
74	**Fig. S73**. The (+)-HRESIMS report of compound **7**, page 2	S82
75	**Fig. S74**. The ^{1}H NMR spectrum of compound **7** in DMSO-d_6 at 500 MHz	S83
76	**Fig. S75**. The ^{13}C NMR spectrum of compound **7** in DMSO-d_6 at 125 MHz	S84
77	**Fig. S76**. The DEPT spectrum of compound **7** in DMSO-d_6 at 125 MHz	S85
78	**Fig. S77**. The ^{1}H-^{1}H COSY spectrum of compound **7** in DMSO-d_6 at 500 MHz	S86
79	**Fig. S78**. The HMQC spectrum of compound **7** in DMSO-d_6 (500 MHz for ^{1}H)	S87
80	**Fig. S79**. The HMBC spectrum of compound **7** in DMSO-d_6 (500 MHz for ^{1}H)	S88
81	**Fig. S80**. The UV spectrum of compound **8**	S89
82	**Fig. S81**. The IR spectrum of compound **8**	S90
83	**Fig. S82**. The ESIMS of compound **8**	S91
84	**Fig. S83**. The (+)-HRESIMS report of compound **8**, page 1	S92
85	**Fig. S84**. The (+)-HRESIMS report of compound **8**, page 2	S93
86	**Fig. S85**. The ^{1}H NMR spectrum of compound **8** in DMSO-d_6 at 500 MHz	S94
87	**Fig. S86**. The ^{13}C NMR spectrum of compound **8** in DMSO-d_6 at 125 MHz	S95

88	**Fig. S87**. The UV spectrum of compound **9**	S96
89	**Fig. S88**. The ESIMS of compound **9**	S97
90	**Fig. S89**. The (+)-HRESIMS report of compound **9**, Page 1	S98
91	**Fig. S90**. The (+)-HRESIMS report of compound **9**, Page 2	S99
92	**Fig. S91**. The (+)-HRESIMS report of compound **9**, Page 3	S100
93	**Fig. S92**. The ^{1}H NMR spectrum of compound **9** in DMSO-d_6 at 500 MHz	S101
94	**Fig. S93**. The ^{13}C NMR spectrum of compound **9** in DMSO-d_6 at 125 MHz	S102
95	**Fig. S94** Overlaid HPLC-UV chromatograms of the reaction mixtures of: (a) gastrodin only in H_2O refluxed for 12 h; (b) D-glucose and 4-hydroxybenzyl alcohol (molar ratio,1:1) in H_2O refluxed for 12 h; (c) gastrodin and 4-hydroxybenzyl alcohol (molar ratio, 1:1) in H_2O refluxed for 12 h; (d) D-glucose and gastrodin (molar ratio, 1:1) in H_2O refluxed for 12 h; (e) 4-hydroxybenzyl alcohol in H_2O refluxed for 12 h	S103
96	**Fig. S95** Overlaid UPLC-HRESIMS (+)-TIC of the reaction mixtures of: (a) gastrodin only in H_2O refluxed for 12 h; (b) D-glucose and 4-hydroxybenzyl alcohol (molar ratio,1:1) in H_2O refluxed for 12 h; (c) gastrodin and 4-hydroxybenzyl alcohol (molar ratio, 1:1) in H_2O refluxed for 12 h; (d) D-glucose and gastrodin (molar ratio, 1:1) in H_2O refluxed for 12 h; (e) 4-hydroxybenzyl alcohol in H_2O refluxed for 12 h	S104
97	**Fig. S96** Overlaid UPLC-HRESIMS (–)-TIC of the reaction mixtures of: (a) gastrodin only in H_2O refluxed for 12 h; (b) D-glucose and 4-hydroxybenzyl alcohol (molar ratio,1:1) in H_2O refluxed for 12 h; (c) gastrodin and 4-hydroxybenzyl alcohol (molar ratio, 1:1) in H_2O refluxed for 12 h; (d) D-glucose and gastrodin (molar ratio, 1:1) in H_2O refluxed for 12 h; (e) 4-hydroxybenzyl alcohol in H_2O refluxed for 12 h	S105
98	**Fig. S97** Overlaid (a) (+)-TIC of the reaction mixture of gastrodin and 4-hydroxybenzyl alcohol (molar ratio, 1:1) in H_2O refluxed for 12 h; (b) the chromatogram of the extracted positive ion at *m/z* 415 $[M + Na]^+$ from (a); (c)–(g) (+)-TIC of aqueous solutions of compounds **1**–**4** and **10**	S106
99	**Fig. S98** Overlaid (a) (+)-TIC of the reaction mixture of gastrodin and 4-hydroxybenzyl alcohol (molar ratio, 1:1) in H_2O refluxed for 12 h; (b) the chromatogram of the extracted negative ion at *m/z* 391 $[M - H]^-$ from (a); (c)–(g) (–)-TIC of aqueous solutions of compounds **1**–**4** and **10**	S107
100	**Fig. S99** Overlaid (a) (+)-TIC of the reaction mixture of gastrodin and 4-hydroxybenzyl alcohol (molar ratio, 1:1) in H_2O refluxed for 12 h; (b) the chromatogram of the extracted positive ion at *m/z* 521 $[M + Na]^+$ from (a); (c) and (d) (+)-TIC of aqueous solutions of compounds **7** and **11**; (e) (–)-TIC of the reaction mixture of gastrodin and 4-hydroxybenzyl alcohol (molar ratio, 1:1) in H_2O refluxed for 12 h; (f) the chromatogram of the extracted negative ion at *m/z* 497 $[M - H]^-$ from (e); (g) and (h) (–)-TIC of aqueous solutions of compounds **7** and **11**	S108
101	**Fig. S100** The overlaid UPLC-HRESIMS (+)-TIC of: (a) an aqueous extract prepared by soaking of the freeze-dried sample of the freshly collected *G. elata* rhizomes at room temperature for 24 h; (b) an aqueous extract prepared by refluxing of the freeze-dried sample of the freshly collected *G. elata* rhizomes for 1 h; (c) an aqueous extract prepared by refluxing of the commercially available "tian ma" sample for 1 h; (d) an ethanol extract prepared by soaking of the freeze-dried sample of the freshly collected *G. elata* rhizomes at room temperature for 24 h; (e) an ethanol extract prepared by refluxing of the freeze-dried sample of the freshly collected *G. elata* rhizomes at room temperature for 1 h; (f) ethanol extract by refluxing of the commercially available "tian ma" sample for 1 h	S109
102	**Fig. S101** The overlaid UPLC-HRESIMS (–)-TIC of: (a) an aqueous extract prepared by soaking of the freeze-dried sample of the freshly collected *G. elata* rhizomes at room temperature for 24 h;	S110

(b) an aqueous extract prepared by refluxing of the freeze-dried sample of the freshly collected *G. elata* rhizomes for 1 h; (c) an aqueous extract prepared by refluxing of the commercially available "tian ma" sample for 1 h; (d) an ethanol extract prepared by soaking of the freeze-dried sample of the freshly collected *G. elata* rhizomes at room temperature for 24 h; (e) an ethanol extract prepared by refluxing of the freeze-dried sample of the freshly collected *G. elata* rhizomes at room temperature for 1 h; (f) ethanol extract by refluxing of the commercially available "tian ma" sample for 1 h

103 **Fig. S102** Overlaid UPLC-HRESIMS chromatograms of the extracted positive ion at *m/z* 415 [M + Na]$^+$ from (+)-TIC of: (a) an aqueous extract prepared by soaking of the freeze-dried sample of the freshly collected *G. elata* rhizomes at room temperature for 24 h; (b) an aqueous extract prepared by refluxing of the freeze-dried sample of the freshly collected *G. elata* rhizomes for 1 h; (c) an aqueous extract prepared by refluxing of the commercially available "tian ma" sample for 1 h; and (d)–(f) for the extracted negative ion at *m/z* 391 [M – H]$^-$ from (–)-TIC of (a)–(c), respectively S111

104 **Fig. S103** Overlaid UPLC-HRESIMS chromatograms of the extracted positive ion at *m/z* 415 [M + Na]$^+$ from (+)-TIC of: (a) an ethanol extract prepared by soaking of the freeze-dried sample of the freshly collected *G. elata* rhizomes at room temperature for 24 h; (b) an ethanol extract prepared by refluxing of the freeze-dried sample of the freshly collected *G. elata* rhizomes for 1 h; (c) an ethanol extract prepared by refluxing of the commercially available "tian ma" sample for 1 h; and (d)–(f) for the extracted negative ion at *m/z* 391 [M – H]$^-$ from (–)-TIC of (a)–(c), respectively S112

105 **Fig. S104** Overlaid (a) (+)-TIC of an aqueous extract prepared by soaking of the freeze-dried sample of the freshly collected *G. elata* rhizomes at room temperature for 24 h; (b) the chromatogram of the extracted positive ion at *m/z* 415 [M + Na]$^+$ from (a); (c)–(g) (+)-TIC of aqueous solutions of compounds **1**–**4** and **10**, respectively S113

106 **Fig. S105** Overlaid (a) (–)-TIC of an aqueous extract prepared by soaking of the freeze-dried sample of the freshly collected *G. elata* rhizomes at room temperature for 24 h; (b) the chromatogram of the extracted negative ion at *m/z* 391 [M – H]$^-$ from (a); (c)–(g) (–)-TIC of aqueous solutions of compounds **1**–**4** and **10**, respectively S114

107 **Fig. S106** Overlaid (a) (+)-TIC of an aqueous extract prepared by refluxing of the freeze-dried sample of the freshly collected *G. elata* rhizomes for 1 h; (b) the chromatogram of the extracted positive ion at *m/z* 415 [M + Na]$^+$ from (a); (c)–(g) (+)-TIC of aqueous solutions of compounds **1**–**4** and **10**, respectively S115

108 **Fig. S107** Overlaid (a) (–)-TIC of an aqueous extract prepared by refluxing of the freeze-dried sample of the freshly collected *G. elata* rhizomes for 1 h; (b) the chromatogram of the extracted negative ion at *m/z* 391 [M – H]$^-$ from (a); (c)–(g) (–)-TIC of aqueous solutions of compounds **1**–**4** and **10**, respectively S116

109 **Fig. S108** Overlaid (a) (+)-TIC of an aqueous extract prepared by refluxing of the commercially available "tian ma" sample for 1 h; (b) the chromatogram of the extracted positive ion at *m/z* 415 [M + Na]$^+$ from (a); (c)–(g) (+)-TIC of aqueous solutions of compounds **1**–**4** and **10**, respectively S117

110 **Fig. S109** Overlaid (a) (–)-TIC of an aqueous extract prepared by refluxing of the commercially available "tian ma" sample for 1 h; (b) the chromatogram of the extracted negative ion at *m/z* 391 [M – H]$^-$ from (a); (c)–(g) (–)-TIC of aqueous solutions of compounds **1**–**4** and **10**, respectively S118

111 **Fig. S110** Overlaid (a) (+)-TIC of an ethanol extract prepared by soaking of the freeze-dried sample of the freshly collected *G. elata* rhizomes at room temperature for 24 h; (b) the chromatogram of the extracted positive ion at *m/z* 415 [M + Na]$^+$ from (a); (c)–(g) (+)-TIC of S119

aqueous solutions of compounds **1–4** and **10**, respectively
112 **Fig. S111** Overlaid (a) (–)-TIC of an ethanol extract prepared by soaking of the freeze-dried sample of the freshly collected *G. elata* rhizomes at room temperature for 24 h; (b) the chromatogram of the extracted negative ion at *m/z* 391 $[M - H]^-$ from (a); (c)–(g) (–)-TIC of aqueous solutions of compounds **1–4** and **10**, respectively S120
113 **Fig. S112** Overlaid (a) (+)-TIC of an ethanol extract prepared by refluxing of the freeze-dried sample of the freshly collected *G. elata* rhizomes for 1 h; (b) the chromatogram of the extracted positive ion at *m/z* 415 $[M + Na]^+$ from (a); (c)–(g) (+)-TIC of aqueous solutions of compounds **1**–**4** and **10**, respectively S121
114 **Fig. S113** Overlaid (a) (–)-TIC of an ethanol extract prepared by refluxing of the freeze-dried sample of the freshly collected *G. elata* rhizomes for 1 h; (b) the chromatogram of the extracted negative ion at *m/z* 391 $[M - H]^-$ from (a); (c)–(g) (–)-TIC of aqueous solutions of compounds **1**–**4** and **10**, respectively S122
115 **Fig. S114** Overlaid (a) (+)-TIC of an ethanol extract prepared by refluxing of the commercially available "tian ma" sample for 1 h; (b) the chromatogram of the extracted positive ion at *m/z* 415 $[M + Na]^+$ from (a); (c)–(g) (+)-TIC of aqueous solutions of compounds **1**–**4** and **10**, respectively S123
116 **Fig. S115** Overlaid (a) (–)-TIC of an ethanol extract prepared by refluxing of the commercially available "tian ma" sample for 1 h; (b) the chromatogram of the extracted negative ion at *m/z* 391 $[M - H]^-$ from (a); (c)–(g) (–)-TIC of aqueous solutions of compounds **1–4** and **10**, respectively S124
117 **Fig. S116** Overlaid UPLC-HRESIMS chromatograms of the extracted positive ion at *m/z* 521 $[M + Na]^+$ from (+)-TIC of: (a) an aqueous extract prepared by soaking of the freeze-dried sample of the freshly collected *G. elata* rhizomes at room temperature for 24 h; (b) an aqueous extract prepared by refluxing of the freeze-dried sample of the freshly collected *G. elata* rhizomes for 1 h; (c) an aqueous extract prepared by refluxing of the commercially available "tian ma" sample for 1 h; and (d)–(f) for the extracted negative ion at *m/z* 497 $[M - H]^-$ from (–)-TIC of (a)–(c), respectively S125
118 **Fig. S117** Overlaid UPLC-HRESIMS chromatograms of the extracted positive ion at *m/z* 521 $[M + Na]^+$ from (+)-TIC of: (a) an ethanol extract prepared by soaking of the freeze-dried sample of the freshly collected *G. elata* rhizomes at room temperature for 24 h; (b) an ethanol extract prepared by refluxing of the freeze-dried sample of the freshly collected *G. elata* rhizomes for 1 h; (c) an ethanol extract prepared by refluxing of the commercially available "tian ma" sample for 1 h; and (d)–(f) for the extracted negative ion at *m/z* 497 $[M - H]^-$ from (–)-TIC of (a)–(c), respectively S126
119 **Fig. S118** Overlaid (a) (+)-TIC of an aqueous extract prepared by soaking of the freeze-dried sample of the freshly collected *G. elata* rhizomes at room temperature for 24 h; (b) the chromatogram of the extracted positive ion at *m/z* 521 $[M + Na]^+$ from (a); (c) and (d) (+)-TIC of aqueous solutions of compounds **7** and **10**; (e) (–)-TIC of an aqueous extract prepared by soaking of the freeze-dried sample of the freshly collected *G. elata* rhizomes at room temperature for 24 h; (f) the chromatogram of the extracted negative ion at *m/z* 497 $[M - H]^-$ from (e); (g) and (h) (–)-TIC of aqueous solutions of compounds **7** and **10** S127
120 **Fig. S119** Overlaid (a) (+)-TIC of an aqueous extract prepared by refluxing of the freeze-dried sample of the freshly collected *G. elata* rhizomes for 1 h; (b) the chromatogram of the extracted positive ion at *m/z* 521 $[M + Na]^+$ from (a); (c) and (d) (+)-TIC of aqueous solutions of compounds **7** and **10**; (e) (–)-TIC of an aqueous extract prepared by refluxing of the freeze-dried sample of the freshly collected *G. elata* rhizomes for 1 h; (f) the chromatogram of the extracted negative ion at *m/z* 497 $[M - H]^-$ from (e); (g) and (h) (–)-TIC of aqueous solutions of compounds **7** and **10** S128

121	**Fig. S120** Overlaid (a) (+)-TIC of an aqueous extract prepared by refluxing of the commercially available “tian ma” sample for 1 h; (b) the chromatogram of the extracted positive ion at *m/z* 521 $[M + Na]^+$ from (a); (c) and (d) (+)-TIC of aqueous solutions of compounds **7** and **10**; (e) (–)-TIC of an aqueous extract prepared by refluxing of the commercially available “tian ma” sample for 1 h; (f) the chromatogram of the extracted negative ion at *m/z* 497 $[M - H]^-$ from (e); (g) and (h) (−)-TIC of aqueous solutions of compounds **7** and **10**	S129
122	**Fig. S121** Overlaid (a) (+)-TIC of an ethanol extract prepared by soaking of the freeze-dried sample of the freshly collected *G. elata* rhizomes at room temperature for 24 h; (b) the chromatogram of the extracted positive ion at *m/z* 521 $[M + Na]^+$ from (a); (c) and (d) (+)-TIC of aqueous solutions of compounds **7** and **10**; (e) (–)-TIC of an ethanol extract prepared by soaking of the freeze-dried sample of the freshly collected *G. elata* rhizomes at room temperature for 24 h; (f) the chromatogram of the extracted negative ion at *m/z* 497 $[M - H]^-$ from (e); (g) and (h) (–)-TIC of aqueous solutions of compounds **7** and **10**	S130
123	**Fig. S122** Overlaid (a) (+)-TIC of an ethanol extract prepared by refluxing of the freeze-dried sample of the freshly collected *G. elata* rhizomes for 1 h; (b) the chromatogram of the extracted positive ion at *m/z* 521 $[M + Na]^+$ from (a); (c) and (d) (+)-TIC of aqueous solutions of compounds **7** and **10**; (e) (–)-TIC of an ethanol extract prepared by refluxing of the freeze-dried sample of the freshly collected *G. elata* rhizomes for 1 h; (f) the chromatogram of the extracted negative ion at *m/z* 497 $[M - H]^-$ from (e); (g) and (h) (–)-TIC of aqueous solutions of compounds **7** and **10**	S131
124	**Fig. S123** Overlaid (a) (+)-TIC of an ethanol extract prepared by refluxing of the commercially available “tian ma” sample for 1 h; (b) the chromatogram of the extracted positive ion at *m/z* 521 $[M + Na]^+$ from (a); (c) and (d) (+)-TIC of aqueous solutions of compounds **7** and **10**; (e) (–)-TIC of an ethanol extract prepared by refluxing of the commercially available “tian ma” sample for 1 h; (f) the chromatogram of the extracted negative ion at *m/z* 497 $[M - H]^-$ from (e); (g) and (h) (−)-TIC of aqueous solutions of compounds **7** and **10**	S132
125	**Fig. S124** Overlaid UPLC-HRESIMS chromatograms of the extracted positive ion at *m/z* 471 $[M + Na]^+$ from (+)-TIC of: (a) an aqueous extract prepared by soaking of the freeze-dried sample of the freshly collected *G. elata* rhizomes at room temperature for 24 h; (b) an aqueous extract prepared by refluxing of the freeze-dried sample of the freshly collected *G. elata* rhizomes for 1 h; (c) an aqueous extract prepared by refluxing of the commercially available “tian ma” sample for 1 h; and (d)–(f) for the extracted negative ion at *m/z* 447 $[M - H]^-$ from (–)-TIC of (a)–(c), respectively	S133
126	**Fig. S125** Overlaid UPLC-HRESIMS chromatograms of the extracted positive ion at *m/z* 471 $[M + Na]^+$ from (+)-TIC of: (a) an ethanol extract prepared by soaking of the freeze-dried sample of the freshly collected *G. elata* rhizomes at room temperature for 24 h; (b) an ethanol extract prepared by refluxing of the freeze-dried sample of the freshly collected *G. elata* rhizomes for 1 h; (c) an ethanol extract prepared by refluxing of the commercially available “tian ma” sample for 1 h; and (d)–(f) for the extracted negative ion at *m/z* 447 $[M - H]^-$ from (–)-TIC of (a)–(c), respectively	S134
127	**Fig. S126** Overlaid (a) (+)-TIC of an aqueous extract prepared by soaking of the freeze-dried sample of the freshly collected *G. elata* rhizomes at room temperature for 24 h; (b) the chromatogram of the extracted positive ion at *m/z* 471 $[M + Na]^+$ from (a); (c)–(g) (+)-TIC of aqueous solutions of compounds **9**, 4-[*β*-D-glucopyranosyl-(1→6)-*β*-D-glucopyranosyloxy]benzyl alcohol, 4-[*α*-D-glucopyranosyl-(1→4)-*β*-D-glucopyranosyloxy]benzyl alcohol, 4-[*β*-D-glucopyra-nosyl-(1→3)-*β*-D-glucopyranosyloxy]benzyl alcohol, and 4-[*β*-D-glucopyranosyl-(1→4)-*β*-D-glucopyranosyloxy]benzyl alcohol	S135

128 **Fig. S127** Overlaid (a) (–)-TIC of an aqueous extract prepared by soaking of the freeze-dried sample of the freshly collected *G. elata* rhizomes at room temperature for 24 h; (b) the chromatogram of the extracted negative ion at *m/z* 447 [M – H]$^-$ from (a); (c)–(g) (–)-TIC of aqueous solutions of compounds **9**, 4-[*β*-D-glucopyranosyl-(1→6)-*β*-D-glucopyranosyloxy]benzyl alcohol, 4-[*a*-D-glucopyranosyl-(1→4)-*β*-D-glucopyranosyloxy]benzyl alcohol, 4-[*β*-D-glucopyra-nosyl-(1→3)-*β*-D-glucopyranosyloxy]benzyl alcohol, and 4-[*β*-D-glucopyranosyl-(1→4)-*β*-D-glucopyranosyloxy]benzyl alcohol S136

129 **Fig. S128** Overlaid (a) (+)-TIC of an aqueous extract prepared by refluxing of the freeze-dried sample of the freshly collected *G. elata* rhizomes for 1 h; (b) the chromatogram of the extracted positive ion at *m/z* 471 [M + Na]$^+$ from (a); (c)–(g) (+)-TIC of aqueous solutions of compounds **9**, 4-[*β*-D-glucopyra-nosyl-(1→6)-*β*-D-glucopyranosyloxy]benzyl alcohol, 4-[*a*-D-glucopyra-nosyl-(1→4)-*β*-D-glucopyranosyloxy]benzyl alcohol, 4-[*β*-D-glucopyranosyl-(1→3)-*β*-D-glucopyrano-syloxy]benzyl alcohol, and 4-[*β*-D-glucopyranosyl-(1→4)-*β*-D-glucopyranosyloxy]benzyl alcohol S137

130 **Fig. S129** Overlaid (a) (–)-TIC of an aqueous extract prepared by refluxing of the freeze-dried sample of the freshly collected *G. elata* rhizomes for 1 h; (b) the chromatogram of the extracted negative ion at *m/z* 447 [M – H]$^-$ from (a); (c)–(g) (–)-TIC of aqueous solutions of compounds **9**, 4-[*β*-D-glucopyranosyl-(1→6)-*β*-D-glucopyranosyloxy]benzyl alcohol, 4-[*a*-D-glucopyranosyl-(1→4)-*β*-D-glucopyranosyloxy]benzyl alcohol, 4-[*β*-D-glucopyranosyl-(1→3)-*β*-D-glucopyrano-syloxy]benzyl alcohol, and 4-[*β*-D-glucopyranosyl-(1→4)-*β*-D-glucopyranosyloxy]benzyl alcohol S138

131 **Fig. S130** Overlaid (a) (+)-TIC of an aqueous extract prepared by refluxing of the commercially available "tian ma" sample for 1 h; (b) the chromatogram of the extracted positive ion at *m/z* 471 [M + Na]$^+$ from (a); (c)–(g) (+)-TIC of aqueous solutions of compounds **9**, 4-[*β*-D-glucopyranosyl-(1→6)-*β*-D-glucopyranosyloxy]benzyl alcohol, 4-[*a*-D-glucopyranosyl-(1→4)-*β*-D-glucopyranosyloxy]benzyl alcohol, 4-[*β*-D-glucopyranosyl-(1→3)-*β*-D-glucopyrano-syloxy]benzyl alcohol, and 4-[*β*-D-glucopyranosyl-(1→4)-*β*-D-glucopyranosyloxy]benzyl alcohol S139

132 **Fig. S131** Overlaid (a) (–)-TIC of an aqueous extract prepared by refluxing of the commercially available "tian ma" sample for 1 h; (b) the chromatogram of the extracted negative ion at *m/z* 447 [M – H]$^-$ from (a); (c)–(g) (–)-TIC of aqueous solutions of compounds **9**, 4-[*β*-D-glucopyranosyl-(1→6)-*β*-D-glucopyranosyloxy]benzyl alcohol, 4-[*a*-D-glucopyranosyl-(1→4)-*β*-D-glucopyranosyloxy]benzyl alcohol, 4-[*β*-D-glucopyranosyl-(1→3)-*β*-D-glucopyrano-syloxy]benzyl alcohol, and 4-[*β*-D-glucopyranosyl-(1→4)-*β*-D-glucopyranosyloxy]benzyl alcohol S140

133 **Fig. S132** Overlaid (a) (+)-TIC of an ethanol extract prepared by soaking of the freeze-dried sample of the freshly collected *G. elata* rhizomes at room temperature for 1 h; (b) the chromatogram of the extracted positive ion at *m/z* 471 [M + Na]$^+$ from (a); (c)–(g) (+)-TIC of aqueous solutions of compounds **9**, 4-[*β*-D-glucopyranosyl-(1→6)-*β*-D-glucopyranosyloxy]benzyl alcohol, 4-[*a*-D-glucopyranosyl-(1→4)-*β*-D-glucopyranosyloxy]benzyl alcohol, 4-[*β*-D-glucopyra-nosyl-(1→3)-*β*-D-glucopyranosyloxy]benzyl alcohol, and 4-[*β*-D-glucopyranosyl-(1→4)-*β*-D-glucopyranosyloxy]benzyl alcohol S141

134 **Fig. S133** Overlaid (a) (–)-TIC of an ethanol extract prepared by soaking of the freeze-dried sample of the freshly collected *G. elata* rhizomes at room temperature for 1 h; (b) the chromatogram of the extracted negative ion at *m/z* 447 [M – H]$^-$ from (a); (c)–(g) (–)-TIC of aqueous solutions of compounds **9**, 4-[*β*-D-glucopyranosyl-(1→6)-*β*-D-glucopyranosyloxy]benzyl alcohol, 4-[*a*-D-glucopyranosyl-(1→4)-*β*-D-glucopyranosyloxy]benzyl alcohol, 4-[*β*-D-glucopy-ranosyl-(1→3)-*β*-D-glucopyranosyloxy]benzyl alcohol, and 4-[*β*-D-glucopyranosyl-(1→4)-*β*-D-glucopyranosyloxy]benzyl alcohol S142

135 **Fig. S134** Overlaid (a) (+)-TIC of an ethanol extract prepared by refluxing of the freeze-dried sample of the freshly collected *G. elata* rhizomes for 1 h; (b) the chromatogram of the extracted positive ion at *m/z* 471 [M + Na]$^+$ from (a); (c)–(g) (+)-TIC of aqueous solutions of compounds **9**, 4-[*β*-D-glucopyranosyl-(1→6)-*β*-D-glucopyranosyloxy]benzyl alcohol, 4-[*a*-D-glucopyranosyl-(1→4)-*β*-D-glucopyranosyloxy]benzyl alcohol, 4-[*β*-D-glucopyranosyl-(1→3)-*β*-D-glucopyranosyloxy]benzyl alcohol, and 4-[*β*-D-glucopyranosyl-(1→4)-*β*-D-glucopyranosyloxy]benzyl alcohol S143

136 **Fig. S135** Overlaid (a) (–)-TIC of an ethanol extract prepared by refluxing of the freeze-dried sample of the freshly collected *G. elata* rhizomes for 1 h; (b) the chromatogram of the extracted negative ion at *m/z* 447 [M – H]$^-$ from (a); (c)–(g) (–)-TIC of aqueous solutions of compounds **9**, 4-[*β*-D-glucopyranosyl-(1→6)-*β*-D-glucopyranosyloxy]benzyl alcohol, 4-[*a*-D-glucopyranosyl-(1→4)-*β*-D-glucopyranosyloxy]benzyl alcohol, 4-[*β*-D-glucopyranosyl-(1→3)-*β*-D-glucopyranosyloxy]benzyl alcohol, and 4-[*β*-D-glucopyranosyl-(1→4)-*β*-D-glucopyranosyloxy]benzyl alcohol S144

137 **Fig. S136** Overlaid (a) (+)-TIC of an ethanol extract prepared by refluxing of the commercially available “tian ma” sample for 1 h; (b) the chromatogram of the extracted positive ion at *m/z* 471 [M + Na]$^+$ from (a); (c)–(g) (+)-TIC of aqueous solutions of compounds **9**, 4-[*β*-D-glucopyranosyl-(1→6)-*β*-D-glucopyranosyloxy]benzyl alcohol, 4-[*a*-D-glucopyranosyl-(1→4)-*β*-D-glucopyranosyloxy]benzyl alcohol, 4-[*β*-D-glucopyranosyl-(1→3)-*β*-D-glucopyranosyloxy]benzyl alcohol, and 4-[*β*-D-glucopyranosyl-(1→4)-*β*-D-glucopyranosyloxy]benzyl alcohol S145

138 **Fig. S137** Overlaid (a) (–)-TIC of an ethanol extract prepared by refluxing of the commercially available “tian ma” sample for 1 h; (b) the chromatogram of the extracted negative ion at *m/z* 447 [M – H]$^-$ from (a); (c)–(g) (–)-TIC of aqueous solutions of compounds **9**, 4-[*β*-D-glucopyranosyl-(1→6)-*β*-D-glucopyranosyloxy]benzyl alcohol, 4-[*a*-D-glucopyranosyl-(1→4)-*β*-D-glucopyranosyloxy]benzyl alcohol, 4-[*β*-D-glucopyranosyl-(1→3)-*β*-D-glucopyranosyloxy]benzyl alcohol, and 4-[*β*-D-glucopyranosyl-(1→4)-*β*-D-glucopyranosyloxy]benzyl alcohol S146

Table S1 Crystal data and structure refinement for **2**

Identification chromatogram code	exp_5796
Empirical formula	$C_{20}H_{24}O_8$
Formula weight	392.39
Temperature / K	109.90(14)
Crystal system	monoclinic
Space group	$P2_1$
a / Å, b / Å, c / Å	11.8184(9), 5.7327(9), 13.7322(15)
α/°, β/°, γ/°	90, 100.035(9), 90
Volume / $Å^3$	916.15(19)
Z	2
ρ_{calc} / mg mm^{-3}	1.422
μ / mm^{-1}	0.927
F(000)	416.0
Crystal size / mm^3	0.320 × 0.030 × 0.020
2Θ range for data collection chromatogram	9.122 to 142.11°
Index ranges	$-14 \le h \le 14$, $-6 \le k \le 6$, $-16 \le l \le 16$
Reflection chromatograms collected	6920
Independent reflection chromatograms	3331[R(int) = 0.0347 (inf-0.9Å)]
Data/restraints/parameters	3331/1/258
Goodness-of-fit on F^2	1.056
Final R indexes [I>2σ (I) i.e. F_o>4σ (F_o)]	R_1 = 0.0391, wR_2 = 0.0980
Final R indexes [all data]	R_1 = 0.0418, wR_2 = 0.1007
Largest diff. peak/hole / e $Å^{-3}$	0.251/–0.234
Flack Parameters	0.00(13)
Completeness	0.9989

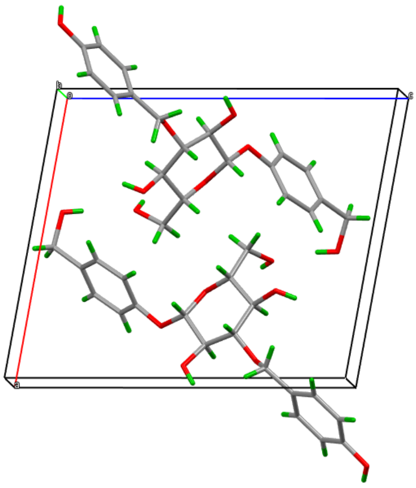

Fig. S1 Crystal cell diagram for compound **2**

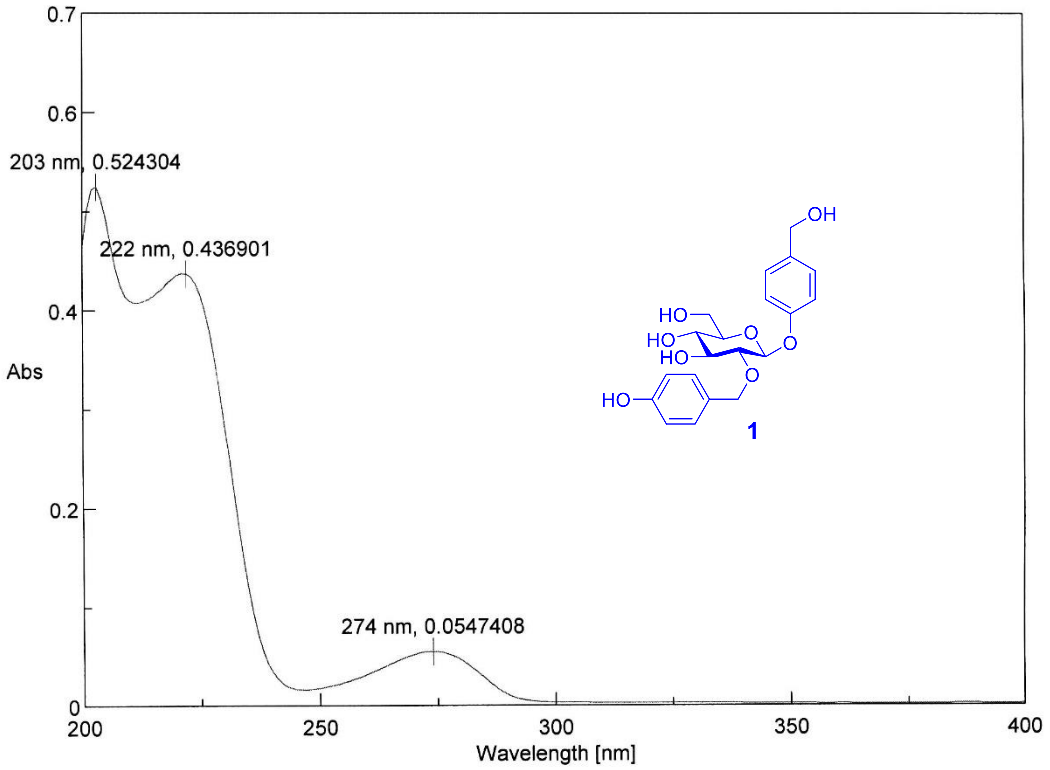

wyn-61-2-w

[Comment]

Sample Name	wyn-61
Comment	
User	王亚男
Division	
Company	324

[Measurement Information]

Instrument Name	V-650
Model Name	V-650
Serial No.	A034461150
Accessory	PSC-718
Accessory S/N	A001761114
Position	1
Cell Length	10 mm
Temperature	19.98 C
Control Sonsor	Holder
Monitor Sensor	Holder
Start Mode	Start immediately
Photometric Mode	Abs
Measurement range	400 - 190 nm
Data pitch	1 nm
Band width(UV/Vis)	1.0 nm
Response	Medium
Scanning speed	200 nm/min
Source Change	340 nm
Light Source	D2/WI
Filter Exchange	Step
Correction	Baseline

[Data Information]

Creation Date	2011-5-11 17:18
Data array type	Linear data array
Horizontal	Wavelength [nm]
Vertical	Abs
Start	400 nm
End	190 nm
Data pitch	1 nm
Data points	211

Fig. S2 The UV spectrum of compound **1**

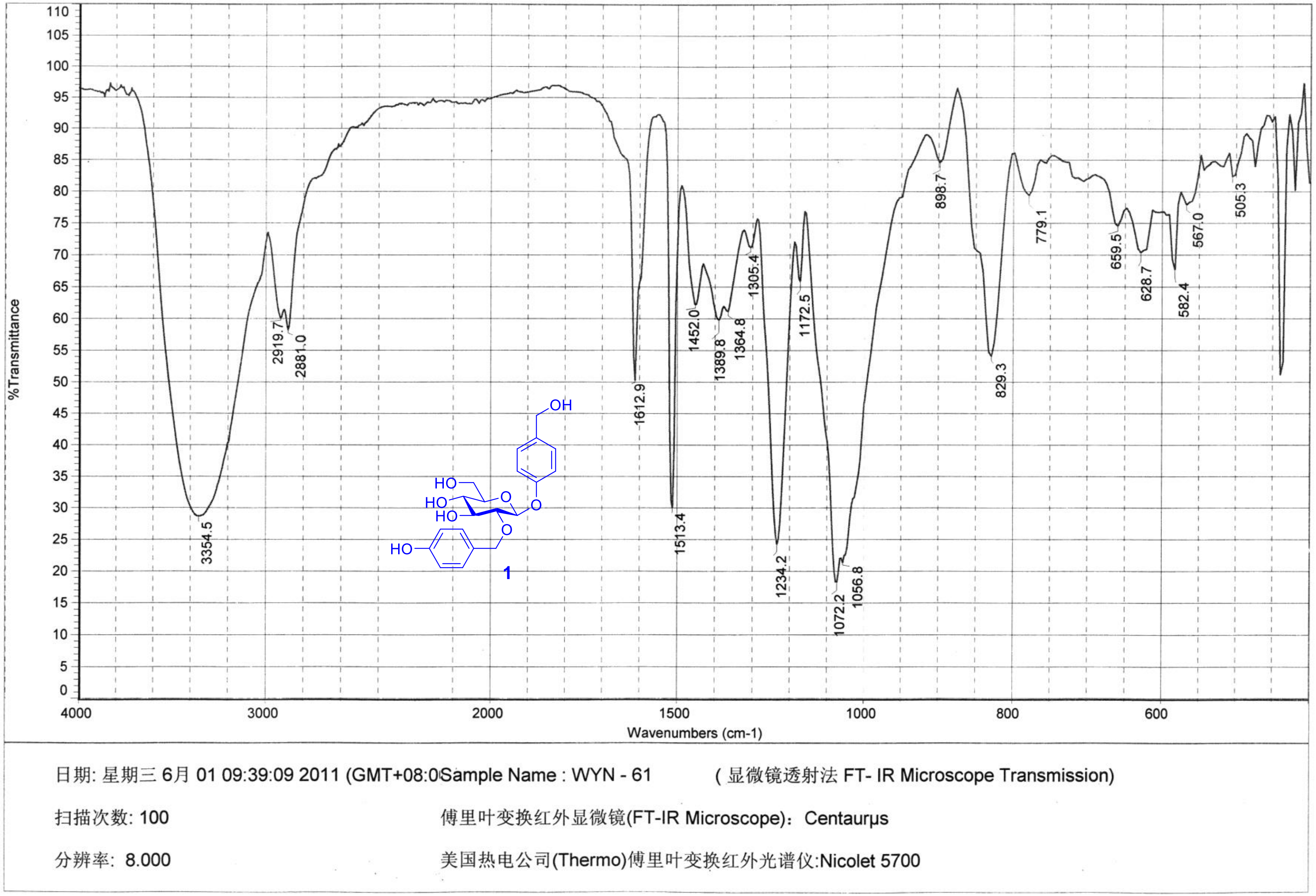

Fig. S3 The IR spectrum of compound **1**

Single Mass Spectrum Deconvolution Report

**Analysis Name:**	wangy024.d	**Instrument:**	LC-MSD-Trap-SL	**Print Date:**	1/5/2011 9:29:46 AM
**Method:**	def_lcms.m	**Operator:**	Operator	**Acq. Date:**	1/5/2011 9:19:35 AM
**Sample Name:**	WYN-61				
**Analysis Info:**					

Acquisition Parameter:

Mass Range Mode	Std/Normal	Trap Drive	36.3	Scan Begin	100 m/z
Ion Polarity	Positive	Octopole RF Amplitude	171.0 Vpp	Scan End	800 m/z
Ion Source Type	ESI	Capillary Exit	106.0 Volt	Averages	5 Spectra
Dry Temp (Set)	330 °C	Skimmer	40.0 Volt	Max. Accu Time	200000 µs
Nebulizer (Set)	15.00 psi	Oct 1 DC	12.00 Volt	ICC Target	30000
Dry Gas (Set)	5.00 l/min	Oct 2 DC	1.70 Volt	Charge Control	on

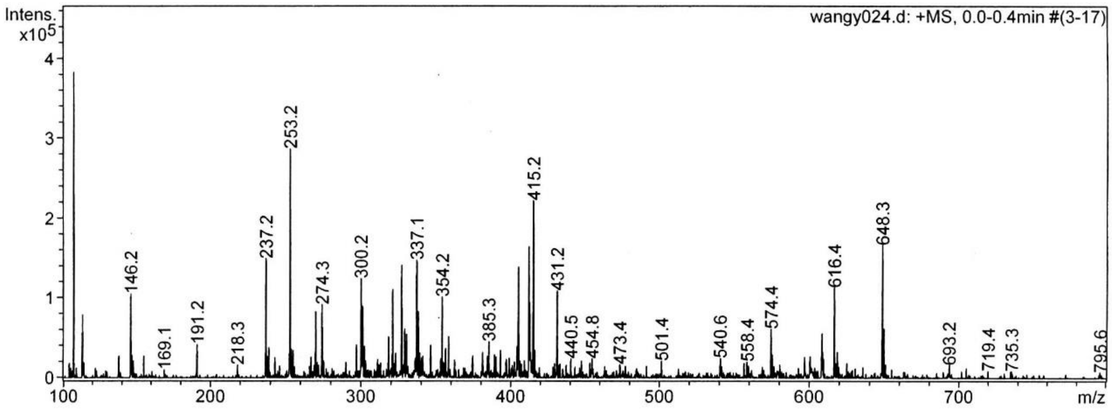

Component	Molecular Mass	Molecule	Absolute Abundance	Relative Abundance

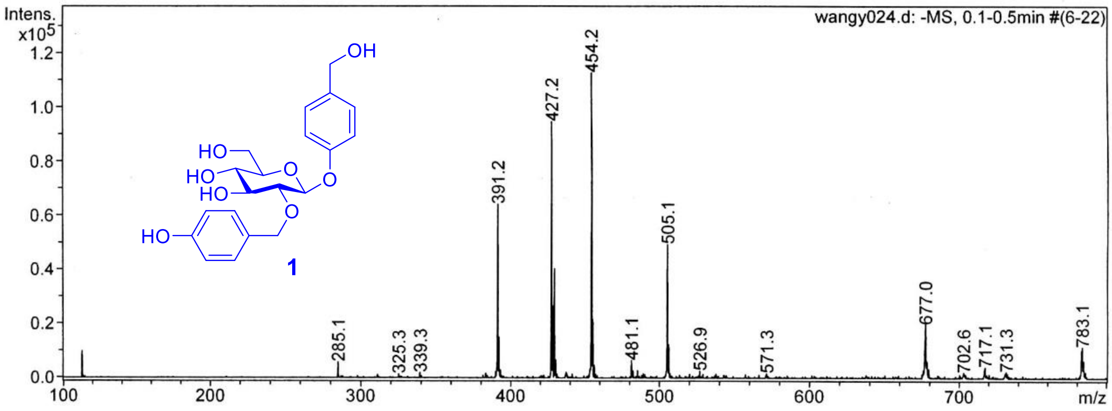

MSD Trap Report v 4 (A4-Opt2) Page 1 of 2 Agilent Technologies

Fig. S4 The ESIMS of compound **1**

Qualitative Analysis Report

**Data Filename**	201101172.d	**Sample Name**	WYN-61
**Sample Type**	Sample	**Position**	P1-C2
**Instrument Name**	Instrument 1	**User Name**	
**Acq Method**		**IRM Calibration Status**	Success
**DA Method**	TEST LCMS.m	**Comment**	

User Chromatograms

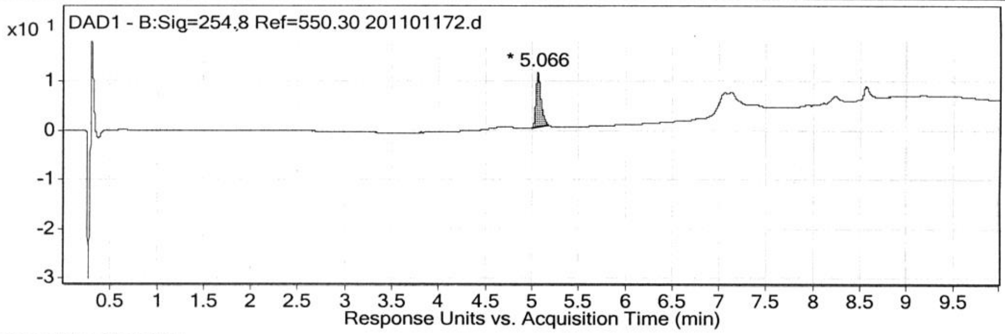

Integration Peak List

Peak	Start	RT	End	Height	Area	Area %
1	4.993	5.066	5.179	10.99	41.56	100

Fragmentor Voltage 135 **Collision Energy** 0 **Ionization Mode** ESI

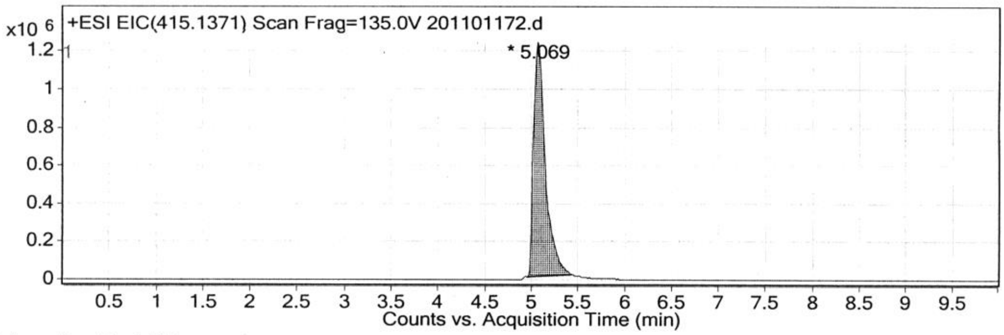

Integration Peak List

Peak	Start	RT	End	Height	Area	Area %
1	4.957	5.069	5.439	1230513	10765008	100

User Spectra

**Fragmentor Voltage**	**Collision Energy**	**Ionization Mode**
135	0	Esi

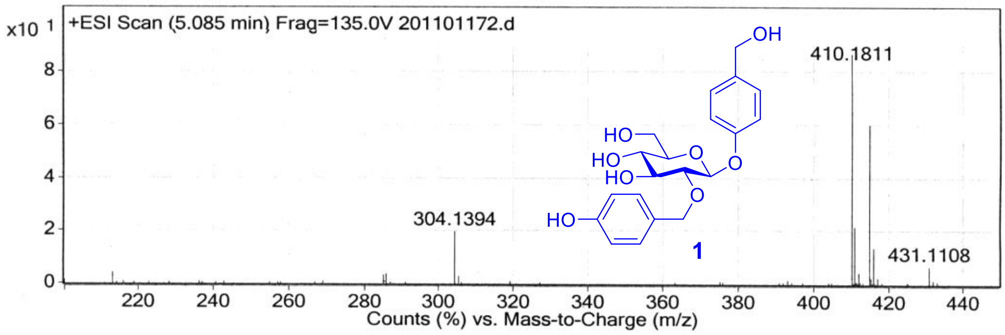

Fig. S5 The (+)-HRESIMS report of compound **1**, Page 1

MS Formula Results: + Scan (5.085 min) (201101172.d)

m/z	Ion	Formula	Abundance
415.1369	(M+Na)+	C20 H24 Na O8	1220320.3

Best	Formula (M)	Ion Formula	Calc m/z	Score	Cross S	Mass	Calc Mass	Diff (ppm)	Abs Diff (ppm)	Abund Match	Spacing Mat	Mass Match	m/z	DBE
☑	C20 H24 O8	C20 H24 Na O8	415.1363	99.93		392.1477	392.1471	-1.56	1.56	99.9	99.98	99.93	415.1369	9

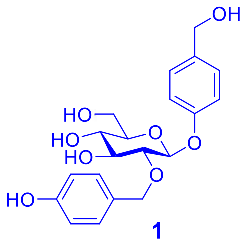

1

page 1

Fig. S6 The (+)-HRESIMS report of compound **1**, Page 2

H1DMSO1217-WYN-61
INOVA-501 1H-NMR WYN-61 IN DMSO 2010.12.17 cold probe

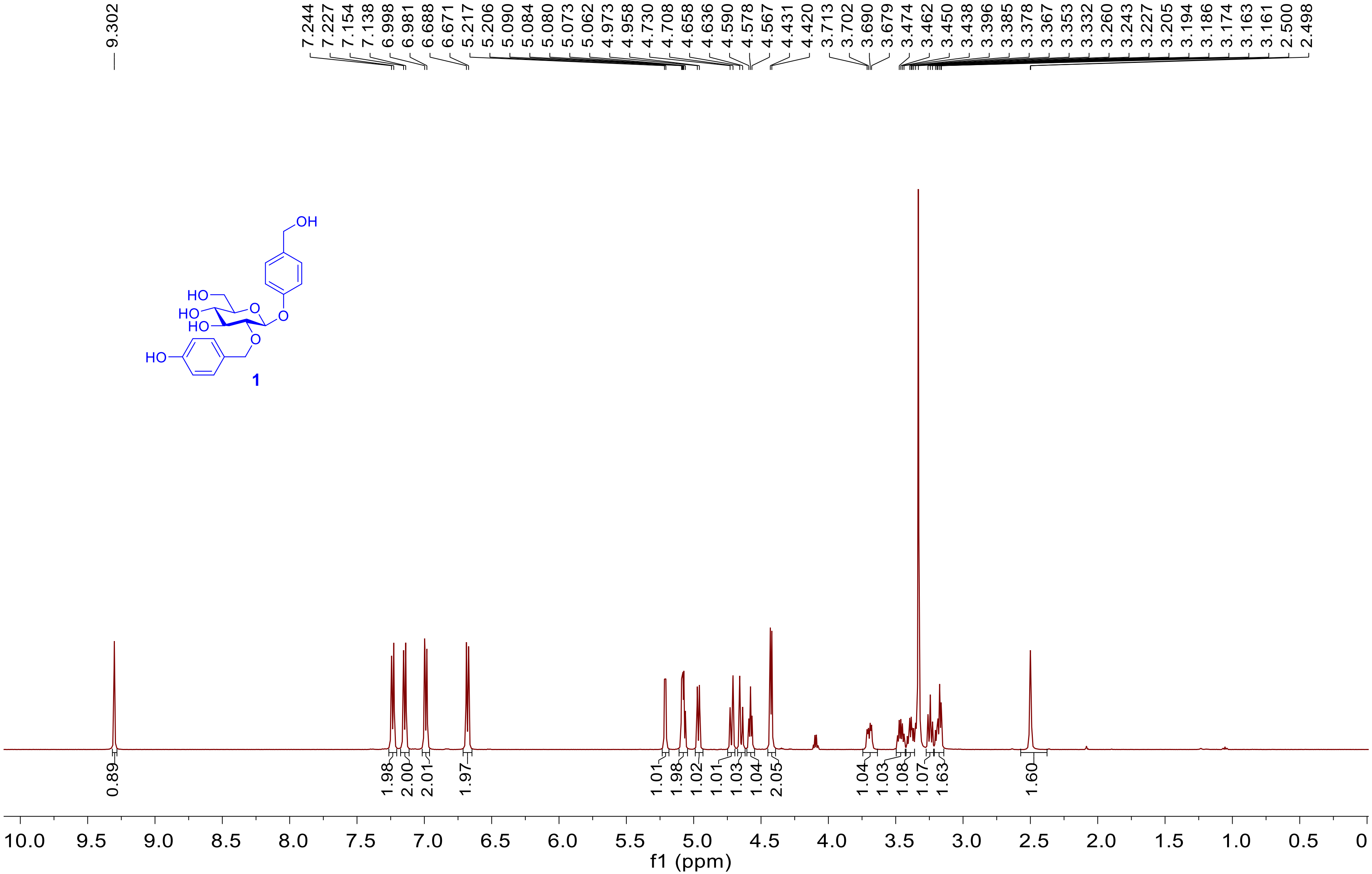

Fig. S7 The ^{1}H NMR spectrum of compound **1** in DMSO-d_6 at 500 MHz

C13DMSO1207-WYN-61
INOVA-501 13C-NMR WYN-61 IN DMSO 2010.12.07

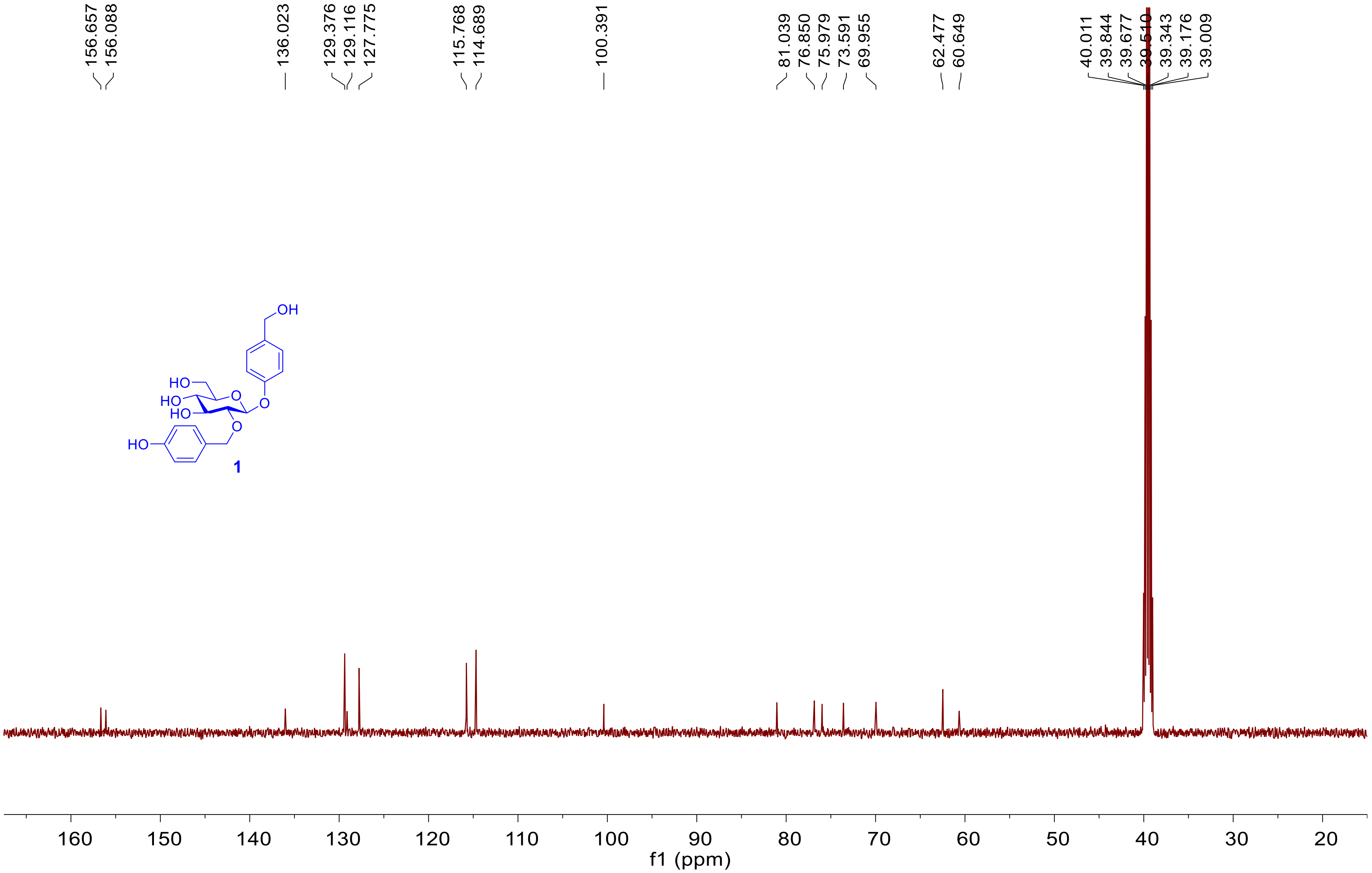

Fig. S8 The ^{13}C NMR spectrum of compound **1** in DMSO-d_6 at 125 MHz

C13DMSO1207-WYN-61
INOVA-501 DEPT WYN-61 IN DMSO 2010.12.07

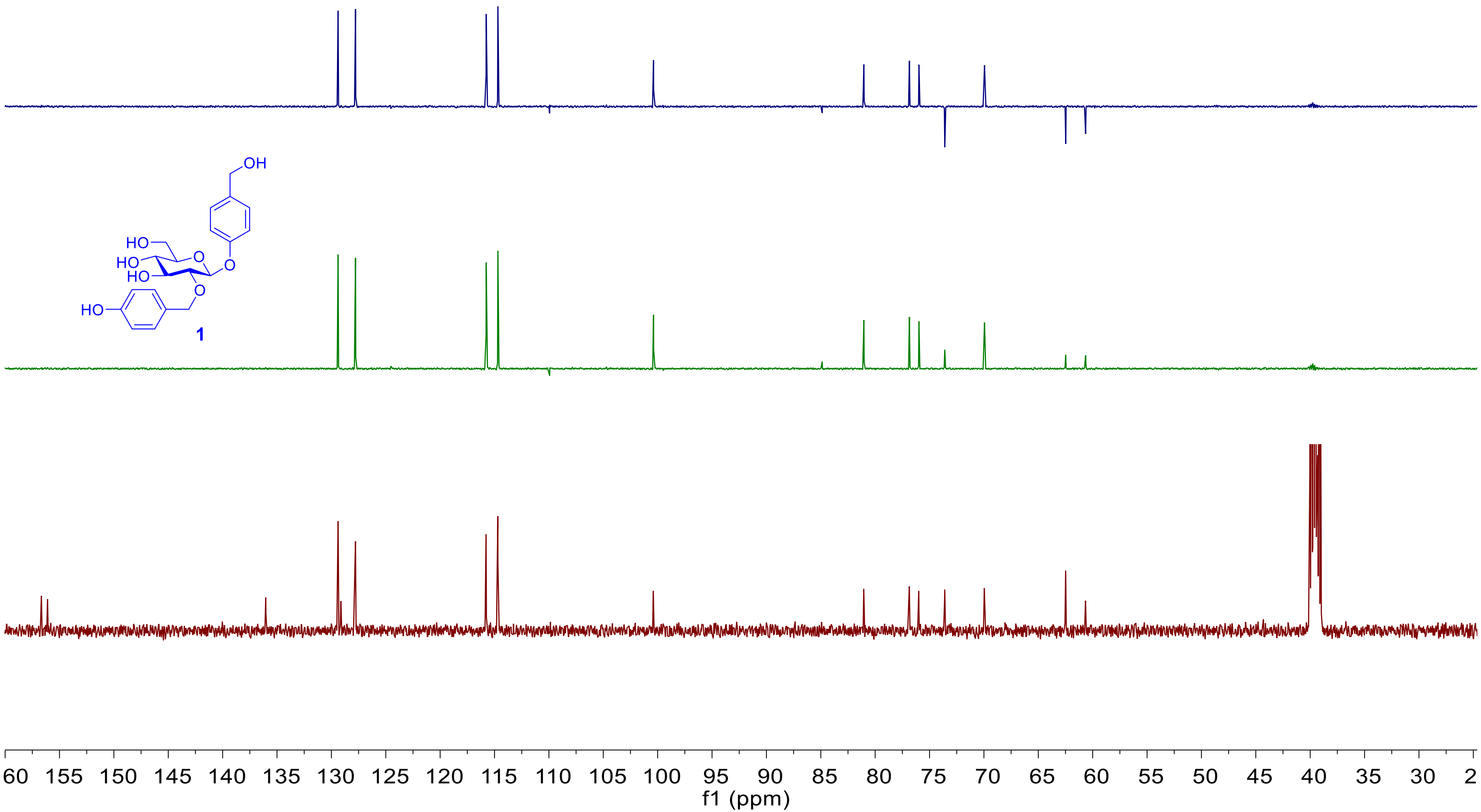

Fig. S9 The DEPT spectrum of compound **1** in DMSO-d_6 at 125 MHz

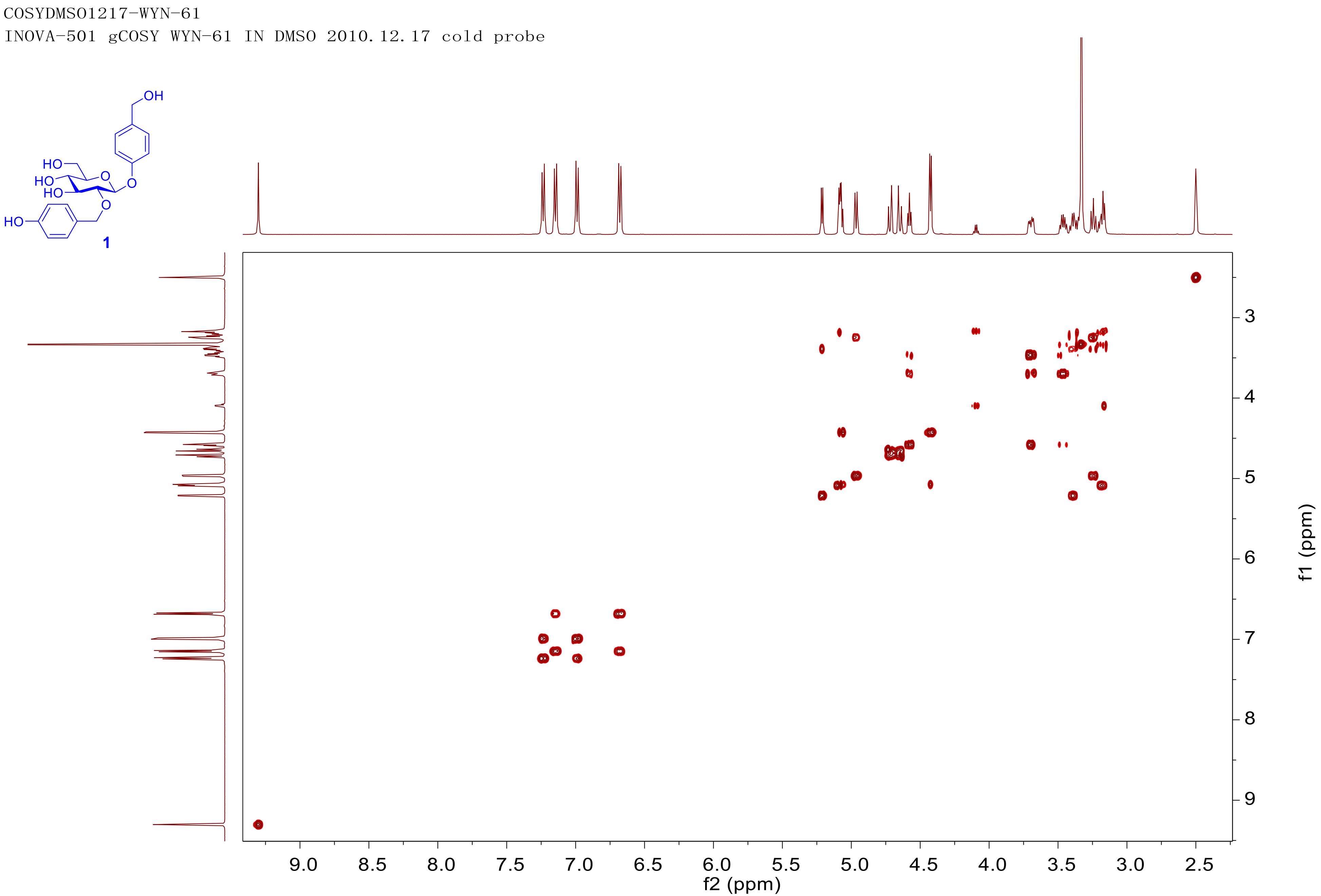

Fig. S10 The ^{1}H-^{1}H COSY spectrum of compound **1** in DMSO-d_6 at 500 MHz

HSQCDMSO1217-WYN-61
INOVA-501 gHSQC WYN-61 IN DMSO 2010.12.17 cold probe

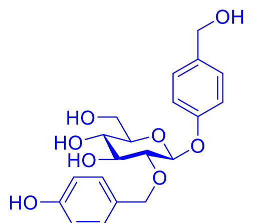

1

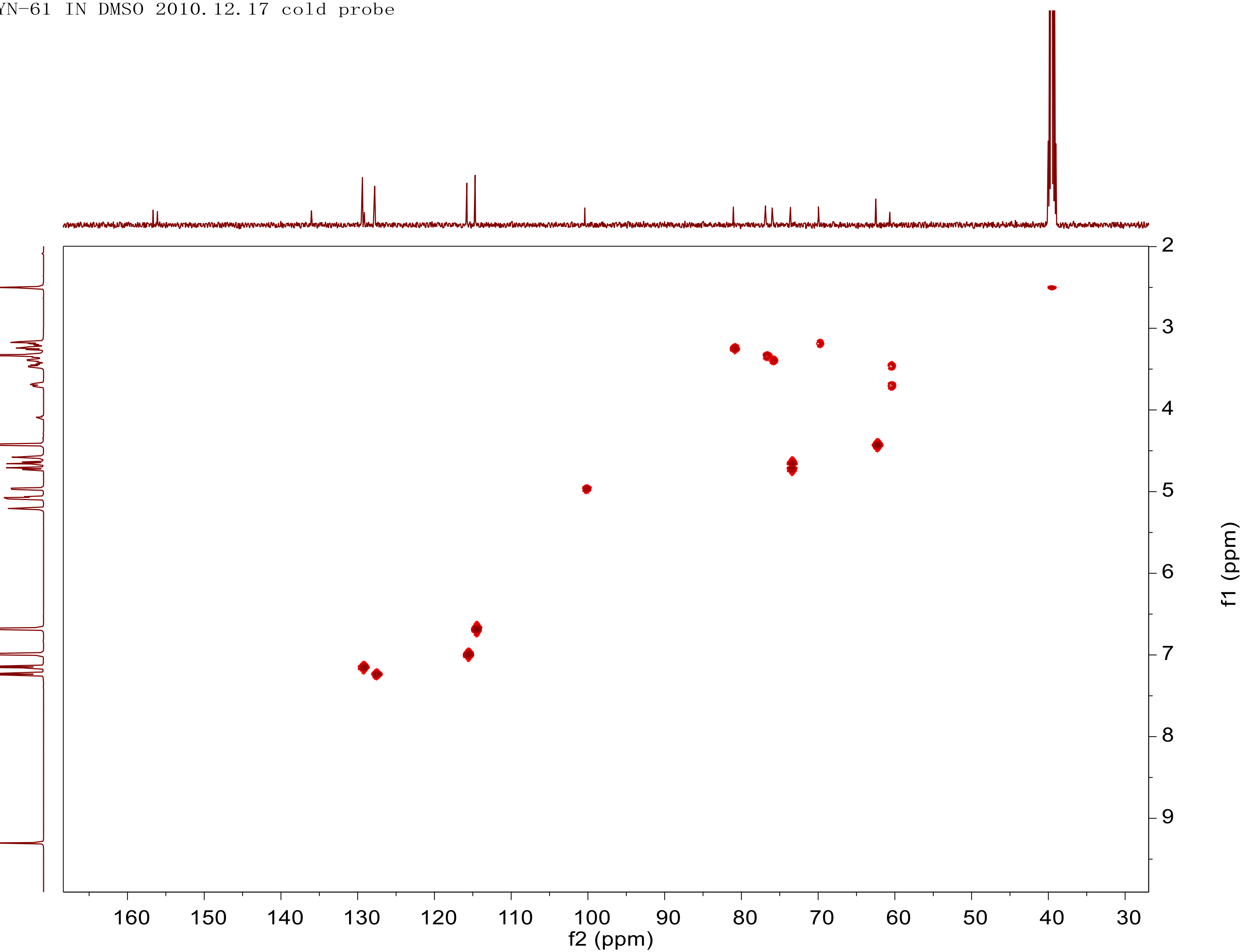

Fig. S11 The HSQC spectrum of compound **1** in DMSO-d_6 (500 MHz for ^{1}H)

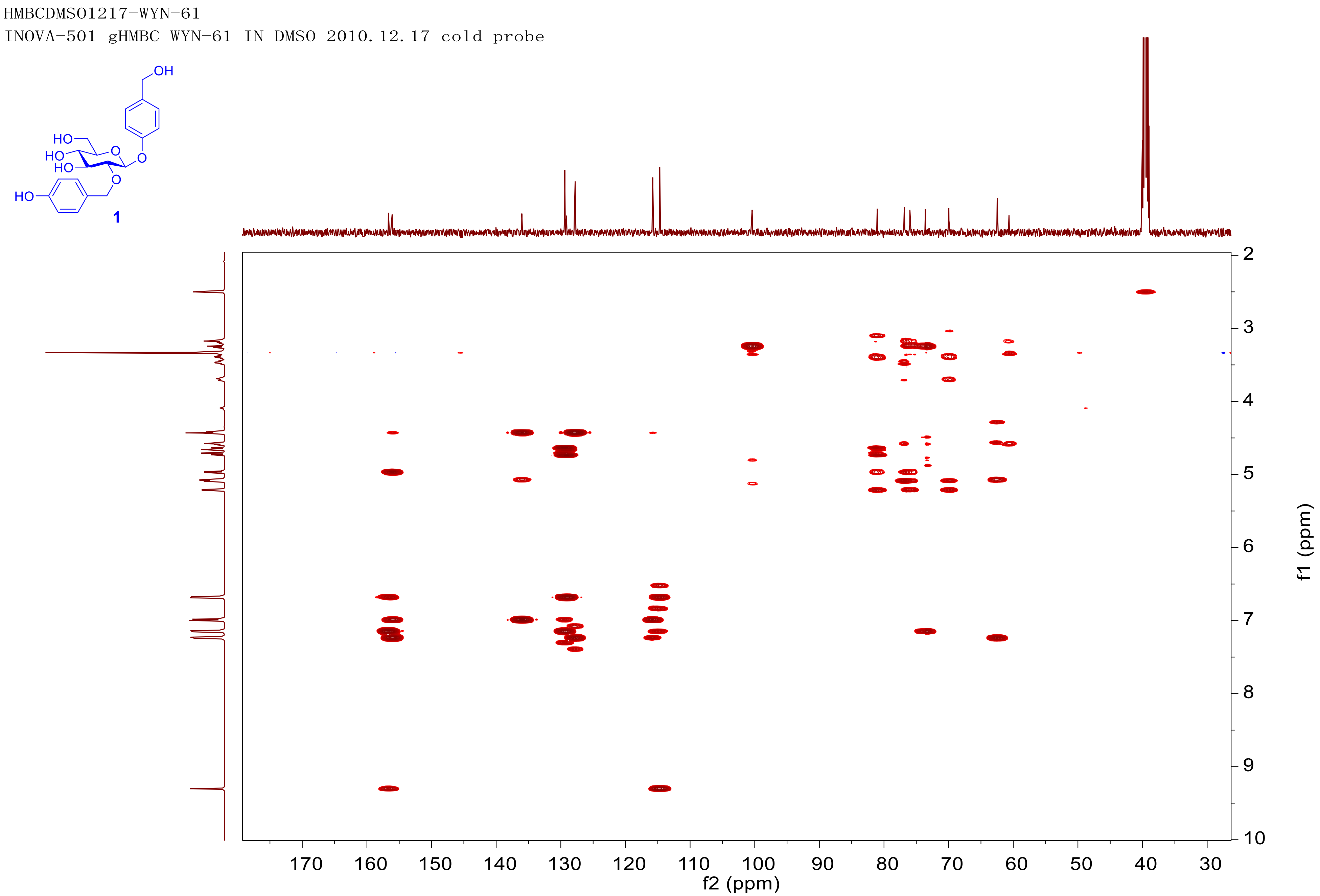

Fig. S12 The HMBC spectrum of compound **1** in DMSO-d_6 (500 MHz for ^{1}H)

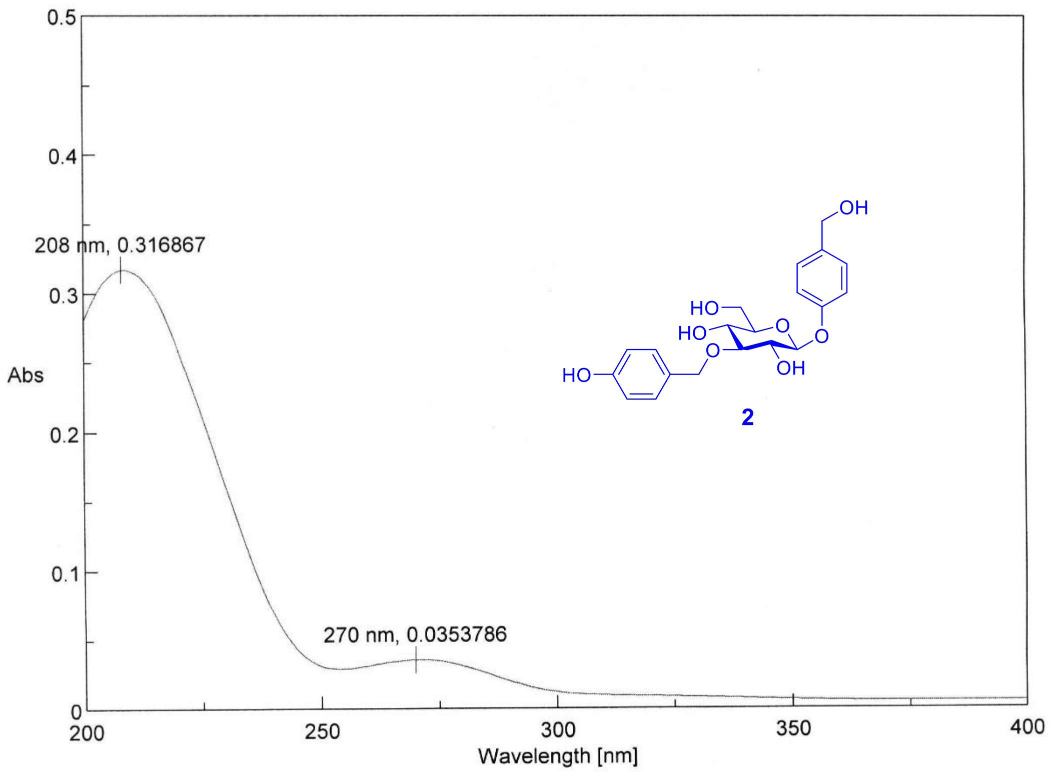

wyn-53-6-w

[Comment]	
Sample Name	wyn-53
Comment	
User	王亚男
Division	
Company	324
[Measurement Information]	
Instrument Name	V-650
Model Name	V-650
Serial No.	A034461150
Accessory	PSC-718
Accessory S/N	A001761114
Position	1
Cell Length	10 mm
Temperature	19.99 C
Control Sonsor	Holder
Monitor Sensor	Holder
Start Mode	Start immediately
Photometric Mode	Abs
Measurement range	400 - 190 nm
Data pitch	1 nm
Band width(UV/Vis)	1.0 nm
Response	Medium
Scanning speed	200 nm/min
Source Change	340 nm
Light Source	D2/WI
Filter Exchange	Step
Correction	Baseline

[Data Information]	
Creation Date	2011-5-11 17:09
Data array type	Linear data array
Horizontal	Wavelength [nm]
Vertical	Abs
Start	400 nm
End	190 nm
Data pitch	1 nm
Data points	211

Fig. S13 The UV spectrum of compound **2**

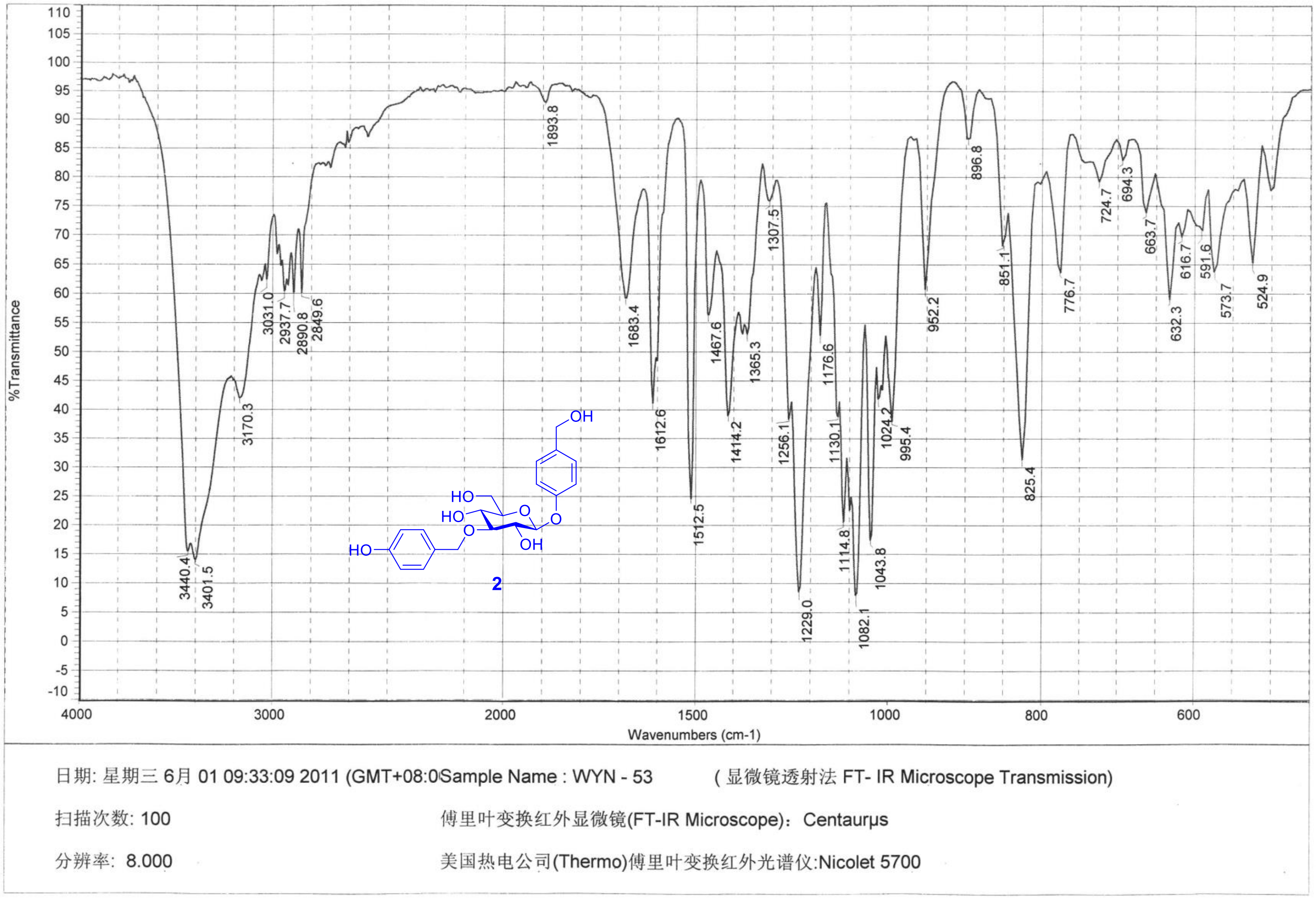

Fig. S14 The IR spectrum of compound **2**

Single Mass Spectrum Deconvolution Report

Analysis Name: wangy035.d
Method: def_lcms.m
Sample Name: WYN-53
Analysis Info:

Instrument: LC-MSD-Trap-SL
Operator: Operator

Print Date: 1/5/2011 9:53:29 AM
Acq. Date: 1/5/2011 9:49:05 AM

Acquisition Parameter:

Mass Range Mode	Std/Normal	Trap Drive	36.3	Scan Begin	100 m/z
Ion Polarity	Positive	Octopole RF Amplitude	171.0 Vpp	Scan End	800 m/z
Ion Source Type	ESI	Capillary Exit	106.0 Volt	Averages	5 Spectra
Dry Temp (Set)	330 °C	Skimmer	40.0 Volt	Max. Accu Time	200000 µs
Nebulizer (Set)	15.00 psi	Oct 1 DC	12.00 Volt	ICC Target	30000
Dry Gas (Set)	5.00 l/min	Oct 2 DC	1.70 Volt	Charge Control	on

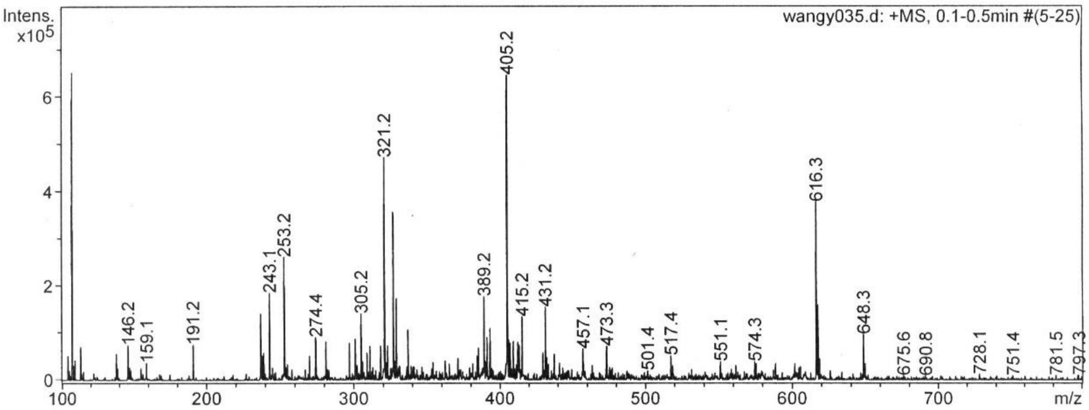

Component	Molecular Mass	Molecule	Absolute Abundance	Relative Abundance

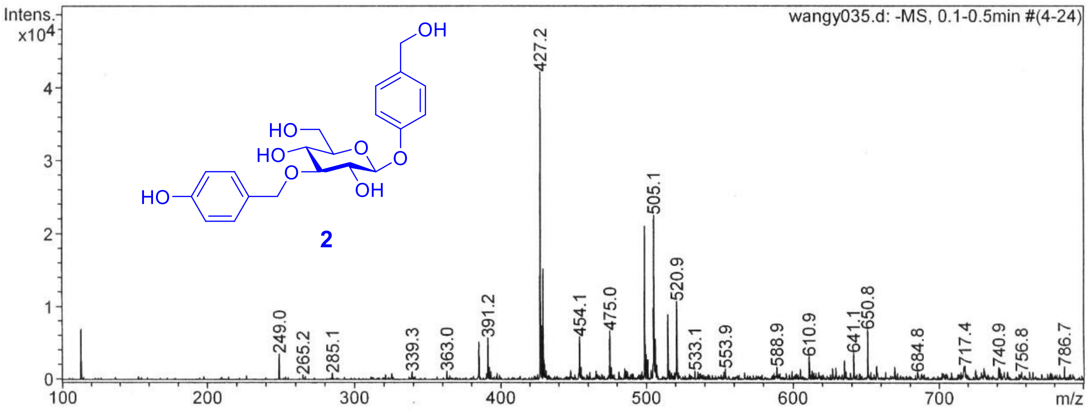

Component	Molecular Mass	Molecule	Absolute Abundance	Relative Abundance

Fig. S15 The ESIMS of compound **2**

Qualitative Analysis Report

**Data Filename**	201101171.d	**Sample Name**	WYN-53
**Sample Type**	Sample	**Position**	P1-C1
**Instrument Name**	Instrument 1	**User Name**	
**Acq Method**		**IRM Calibration Status**	Success
**DA Method**	TEST LCMS.m	**Comment**	

User Chromatograms

Fragmentor Voltage 135 **Collision Energy** 0 **Ionization Mode** ESI

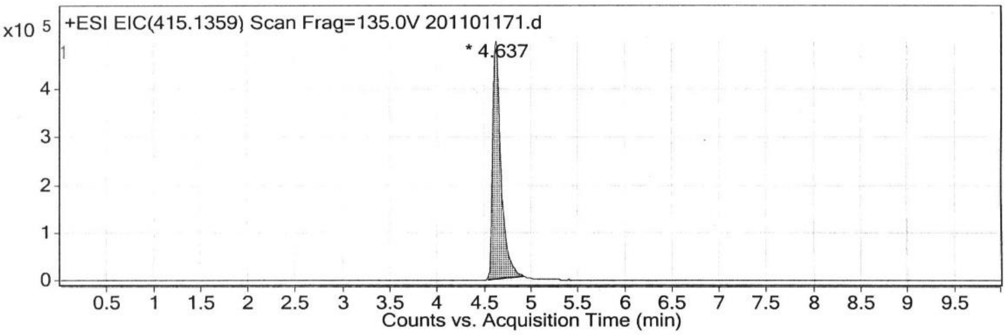

Integration Peak List

Peak	Start	RT	End	Height	Area	Area %
1	4.541	4.637	4.926	496476	3308291	100

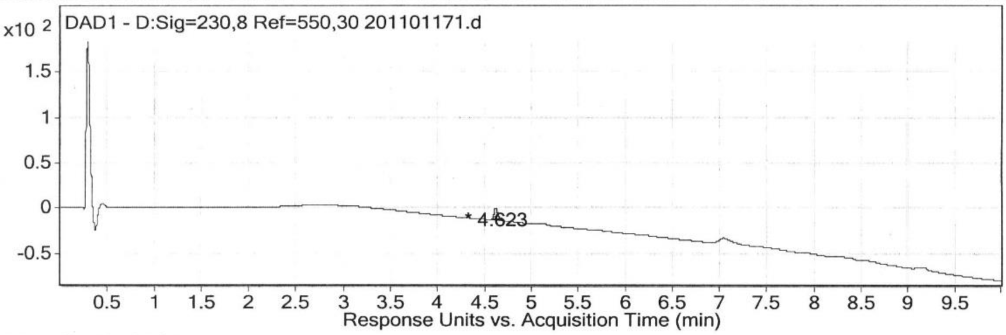

Integration Peak List

Peak	Start	RT	End	Height	Area	Area %
1	4.578	4.623	4.705	12.73	39.96	100

User Spectra

Fragmentor Voltage	Collision Energy	Ionization Mode
135	0	Esi

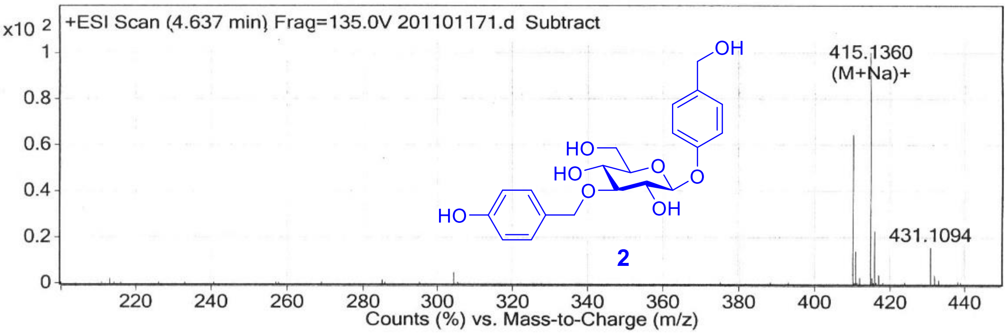

Fig. S16 The (+)-HRESIMS report of compound **2**, Page 1

MS Formula Results: + Scan (4.637 min) Sub (201101171.d)

m/z	Ion	Formula	Abundance
415.136	(M+Na)+	C20 H24 Na O8	499468.5

Best	Formula (M)	Ion Formula	Calc m/z	Score	Cross S	Mass	Calc Mass	Diff (ppm)	Abs Diff (ppm)	Abund Match	Spacing Mat	Mass Match	m/z	DBE
☑	C20 H24 O8	C20 H24 Na O8	415.1363	99.96		392.1468	392.1471	0.89	0.89	99.93	99.97	99.98	415.136	9
☐	C15 H24 N2 O10	C15 H24 N2 Na O10	415.1323	97.97		392.1468	392.1431	-9.38	9.38	97.21	100	97.42	415.136	5

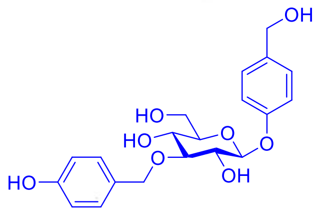

2

page 1

Fig. S17 The (+)-HRESIMS report of compound **2**, Page 2

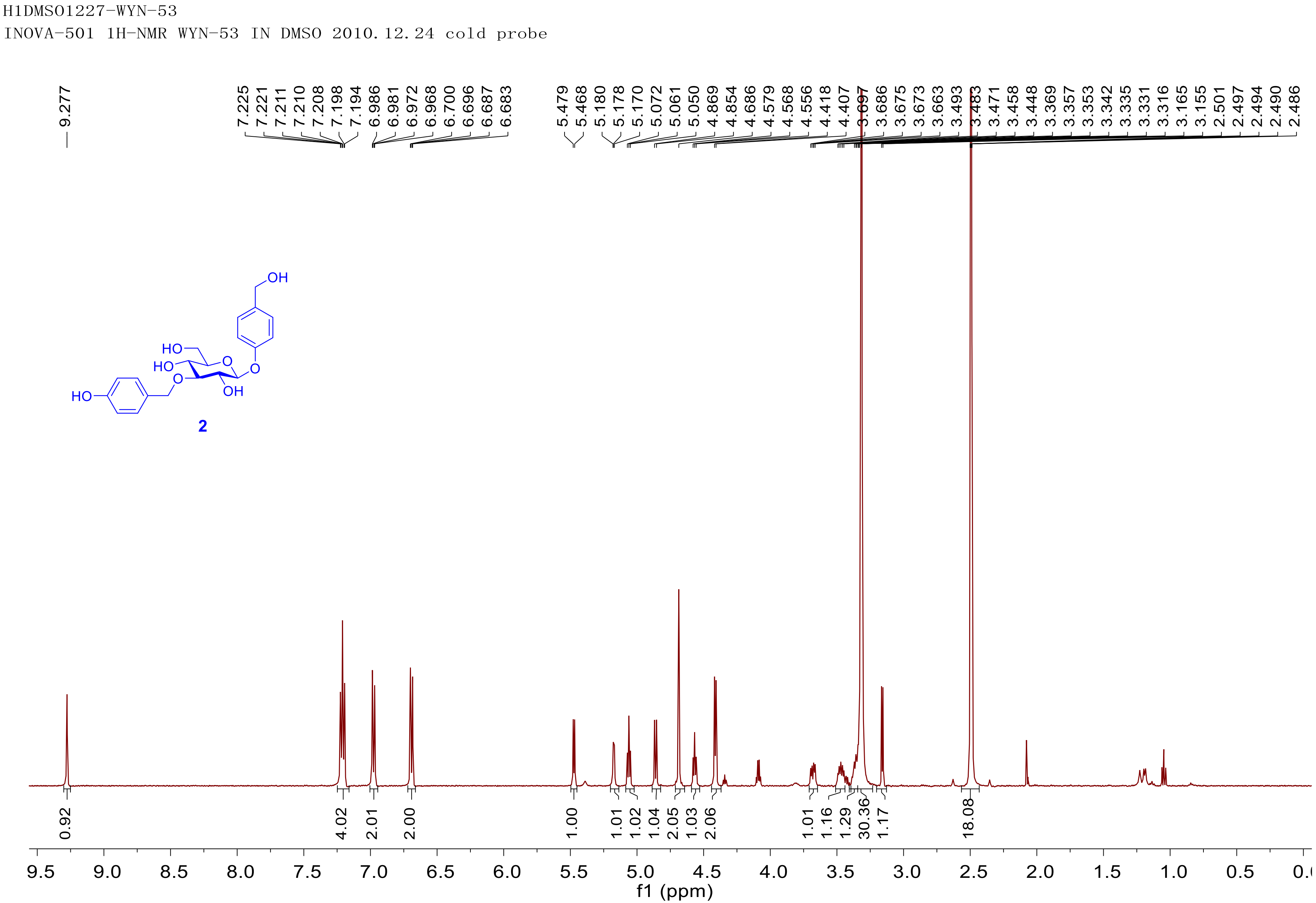

Fig. S18 The ^{1}H NMR spectrum of compound **2** in DMSO-d_6 at 500 MHz

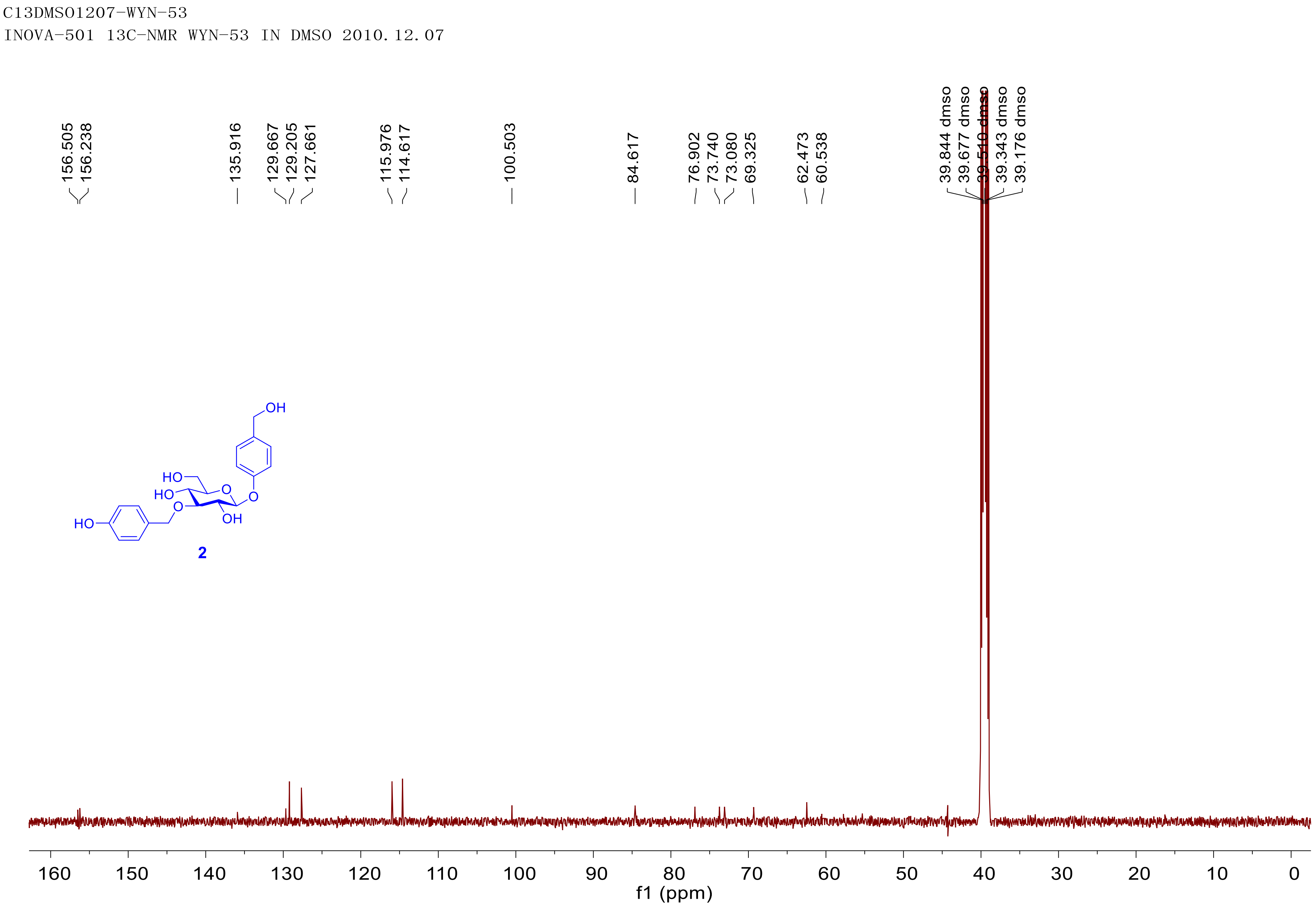

Fig. S19 The ^{13}C NMR spectrum of compound **2** in DMSO-d_6 at 125 MHz

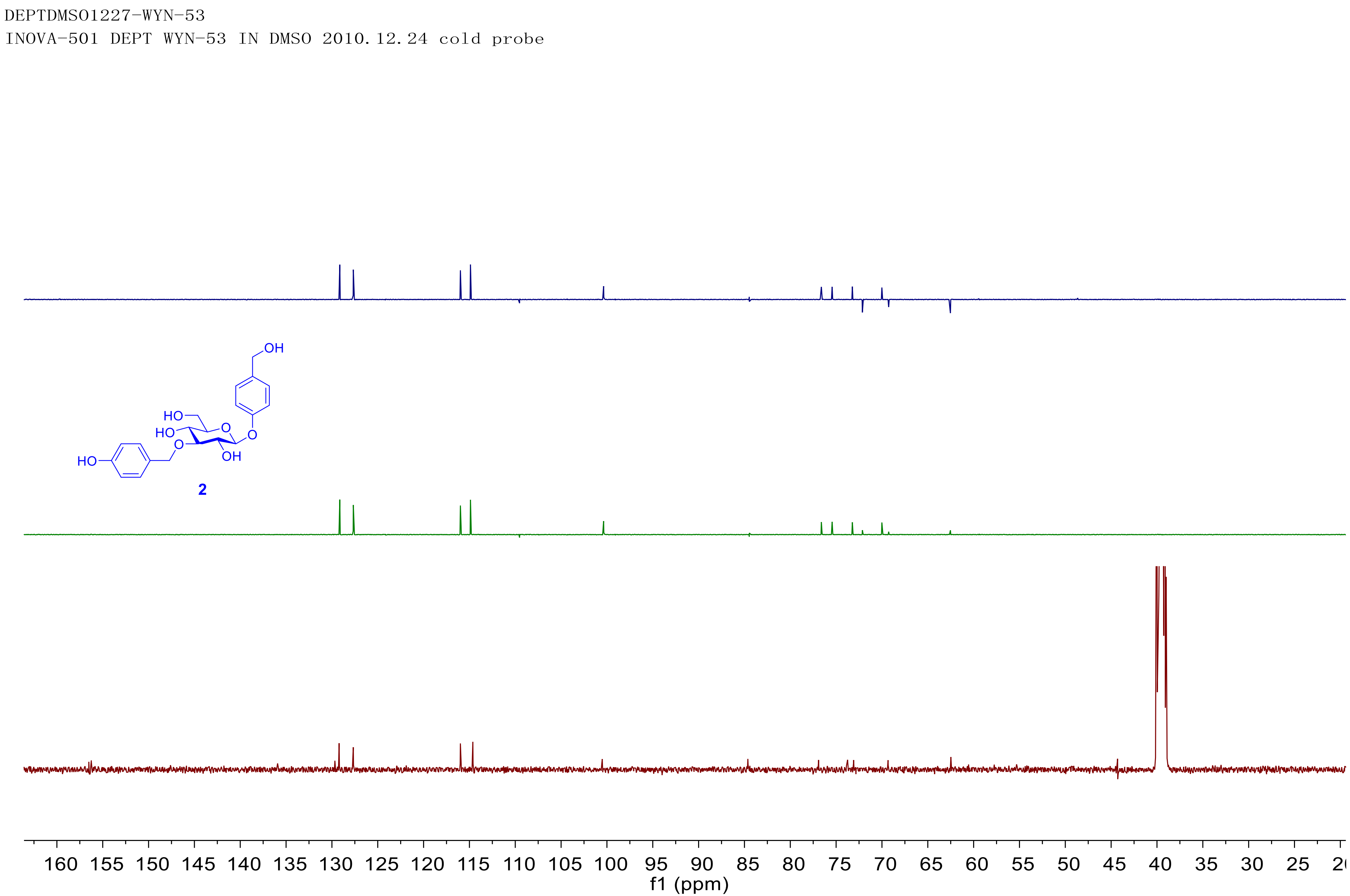

Fig. S20 The DEPT spectrum of compound **2** in DMSO-d_6 at 125 MHz

COSYDMSO1227-WYN-53
INOVA-501 gCOSY WYN-53 IN DMSO 2010.12.24 cold probe

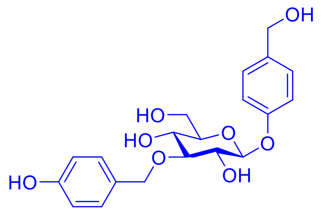

2

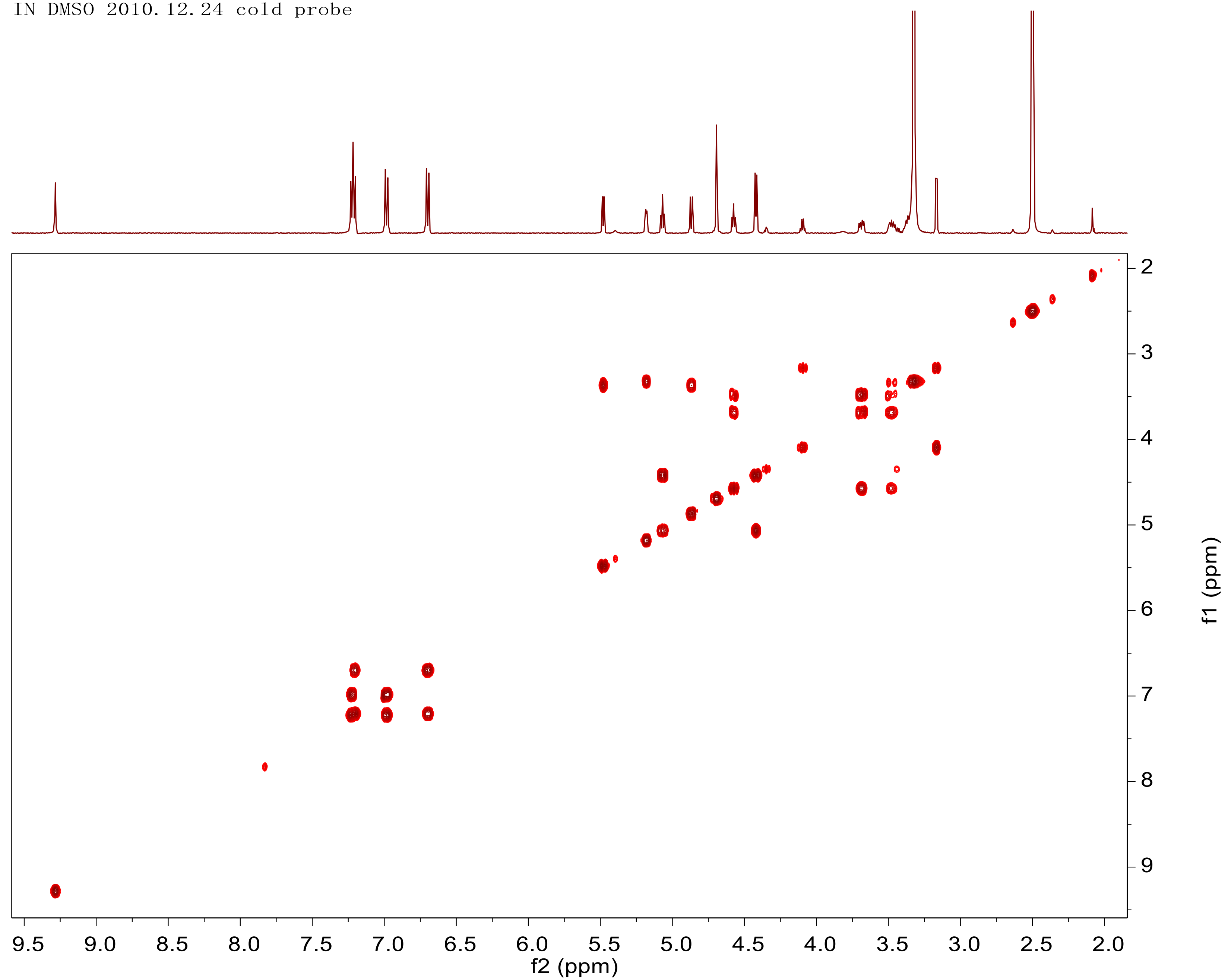

Fig. S21 The ^{1}H-^{1}H COSY spectrum of compound **2** in DMSO-d_6 at 500 MHz

HSQCDMSO1227-WYN-53
INOVA-501 gHSQC WYN-53 IN DMSO 2010.12.24 cold probe

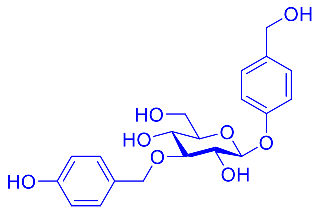

2

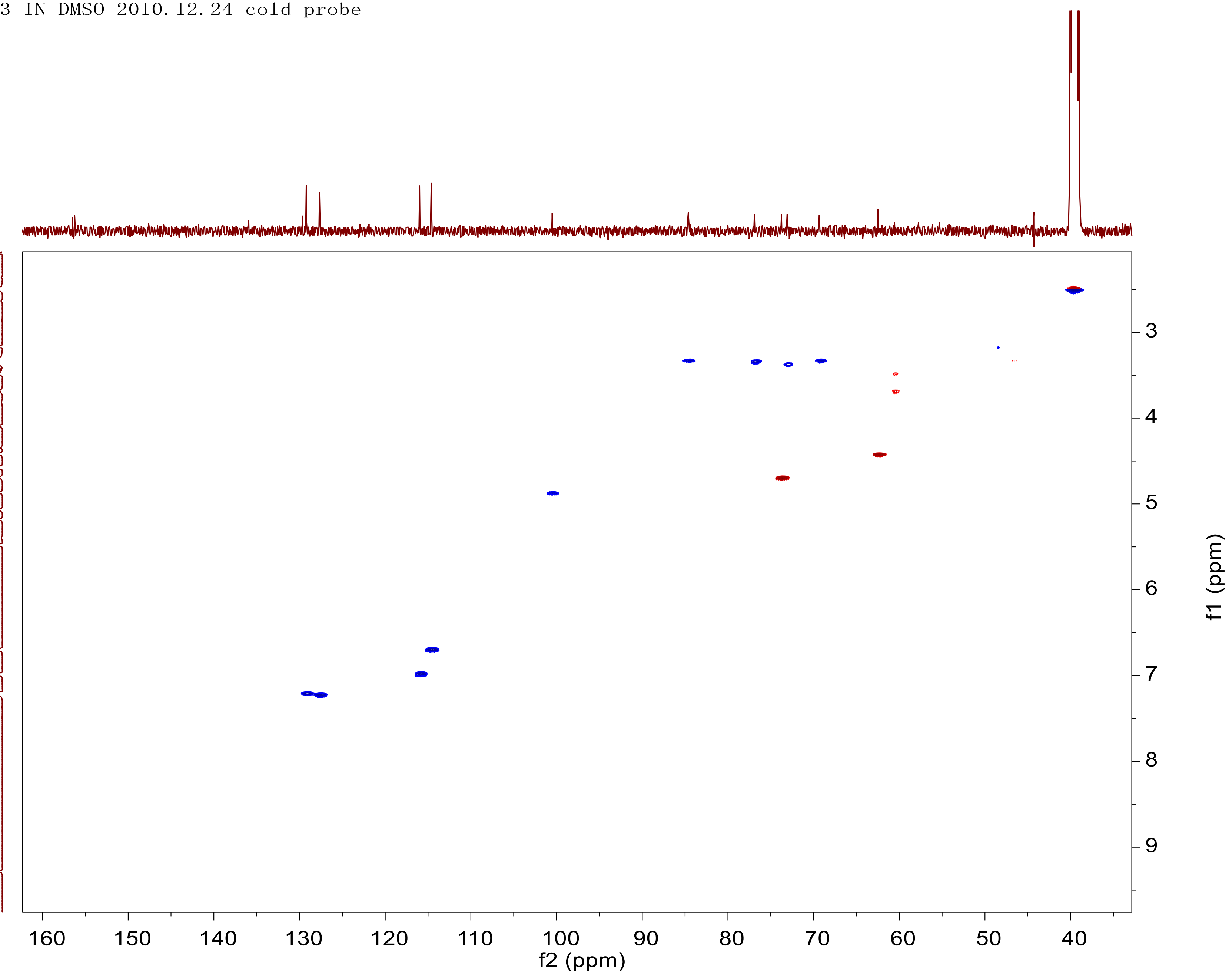

Fig. S22 The HSQC spectrum of compound **2** in DMSO-d_6 (500 MHz for ^{1}H)

HMBCDMSO1227-WYN-53
INOVA-501 gHMBC WYN-53 IN DMSO 2010.12.24 cold probe

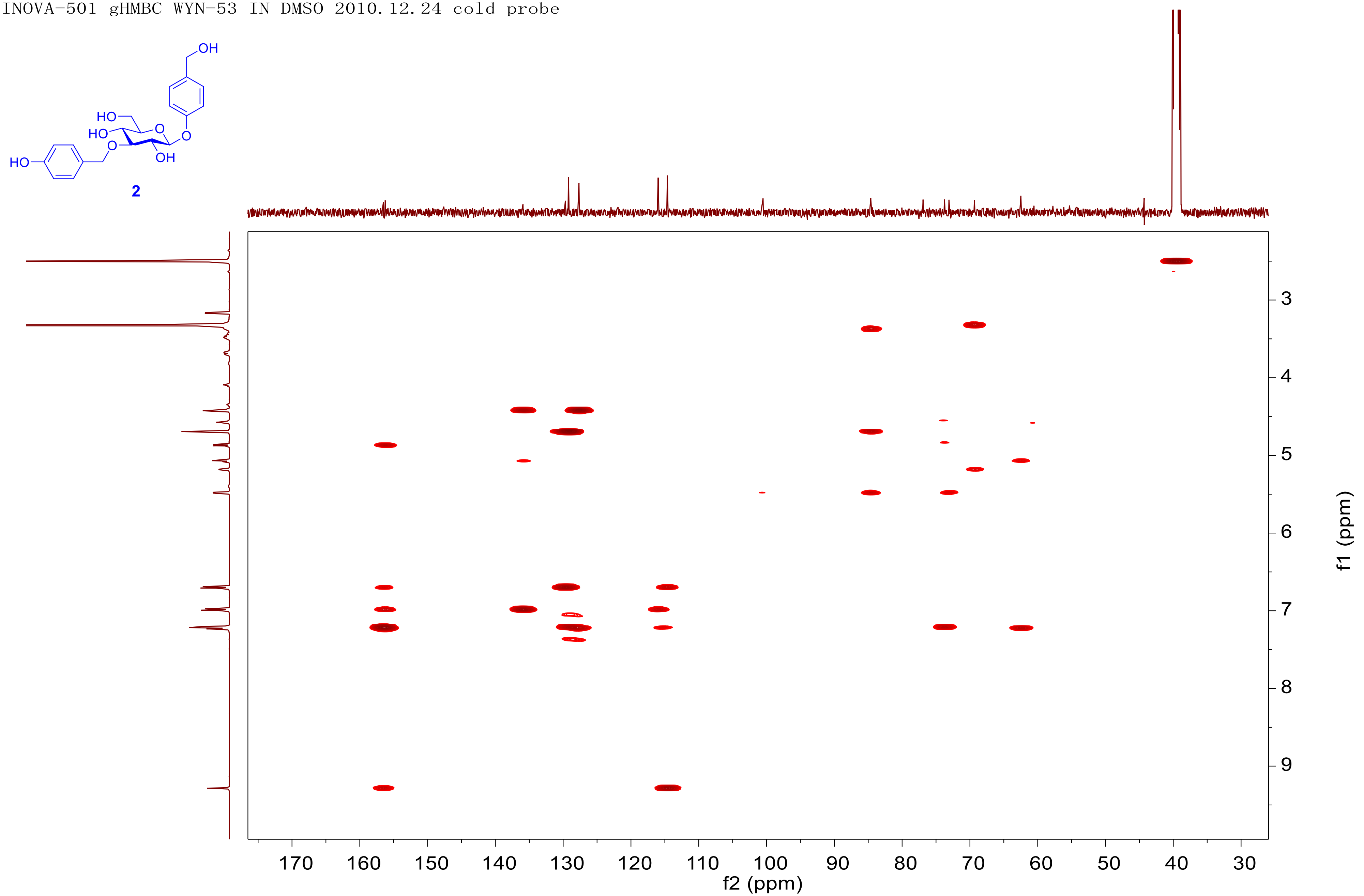

Fig. S23 The HMBC spectrum of compound **2** in DMSO-d_6 (500 MHz for ^{1}H)

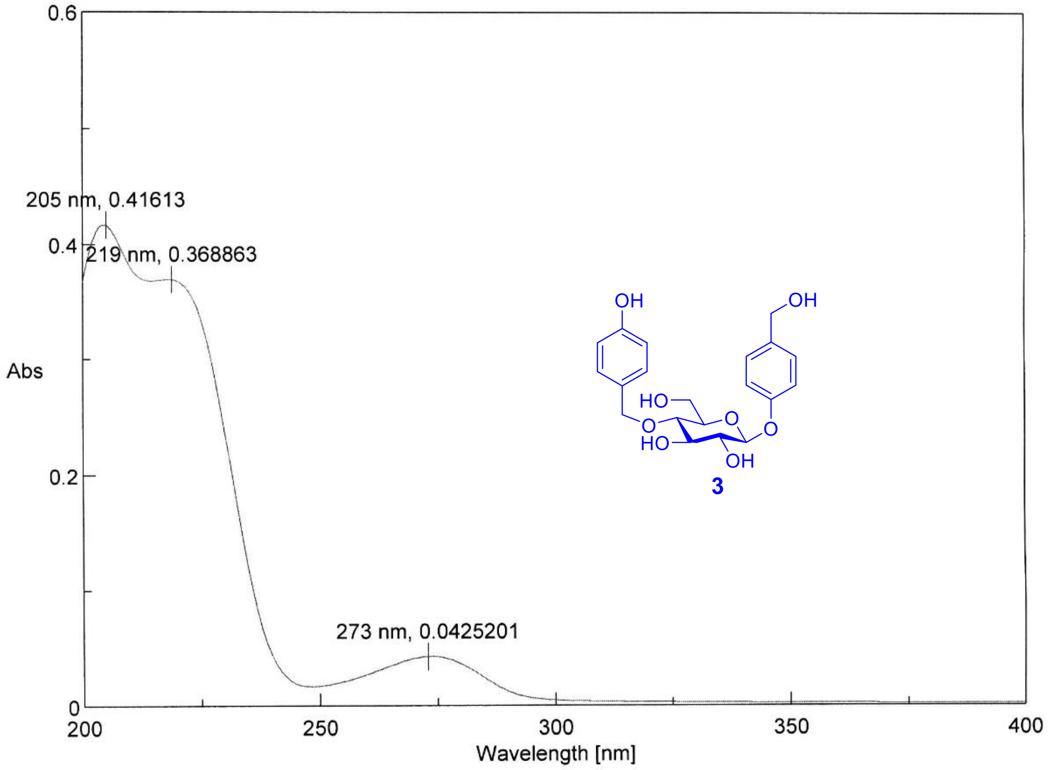

wyn-51-4-w

[Comment]
Sample Name wyn-51
Comment
User 王亚男
Division
Company 324

[Measurement Information]
Instrument Name V-650
Model Name V-650
Serial No. A034461150

Accessory PSC-718
Accessory S/N A001761114
Position 1
Cell Length 10 mm
Temperature 19.99 C
Control Sensor Holder
Monitor Sensor Holder
Start Mode Start immediately

Photometric Mode Abs
Measurement range 400 - 190 nm
Data pitch 1 nm
Band width(UV/Vis) 1.0 nm
Response Medium
Scanning speed 200 nm/min
Source Change 340 nm
Light Source D2/WI
Filter Exchange Step
Correction Baseline

[Data Information]
Creation Date 2011-5-11 17:32

Data array type Linear data array
Horizontal Wavelength [nm]
Vertical Abs
Start 400 nm
End 190 nm
Data pitch 1 nm
Data points 211

Fig. S24 The UV spectrum of compound **3**

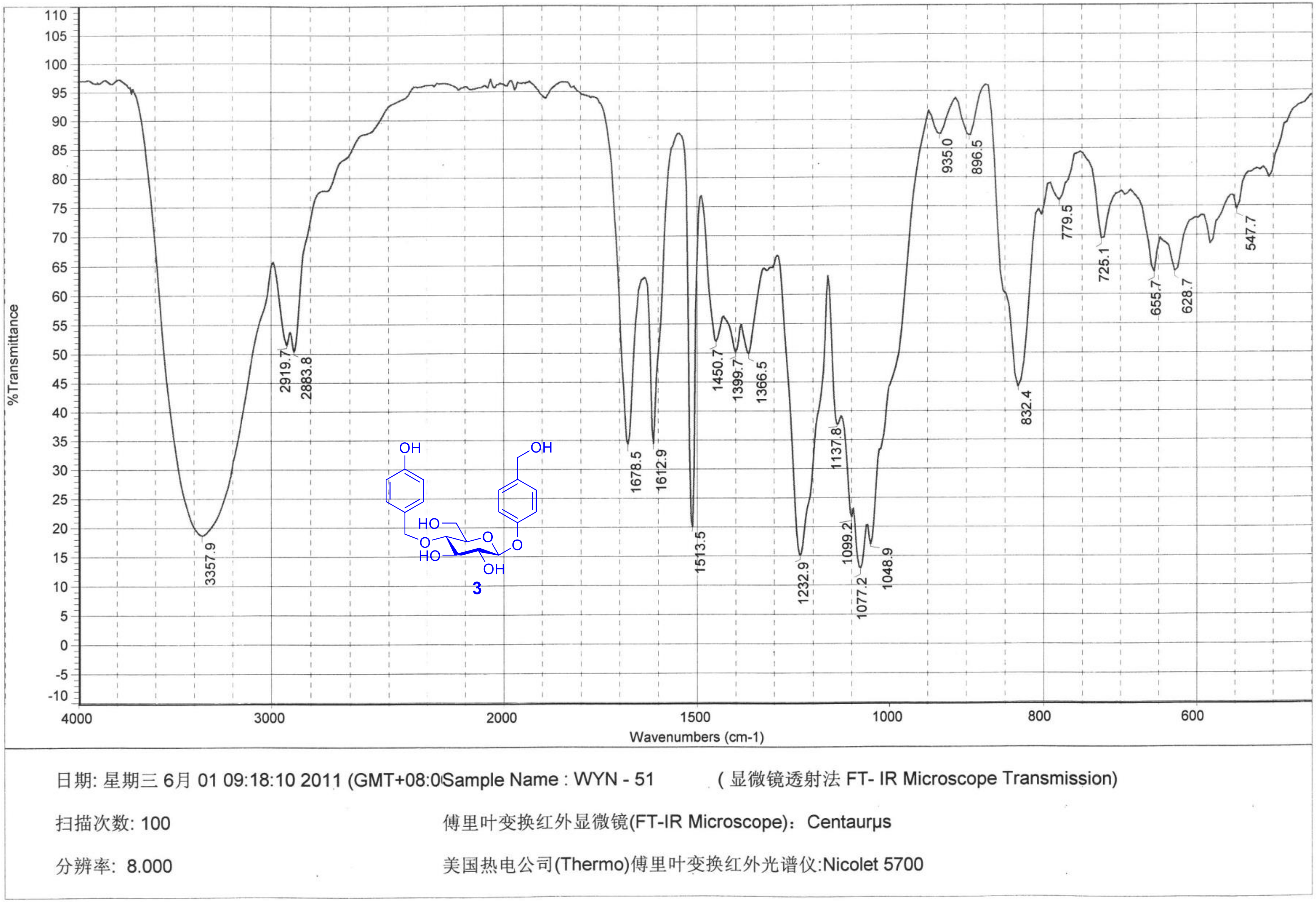

Fig. S25 The IR spectrum of compound **3**

Single Mass Spectrum Deconvolution Report

Analysis Name: wangy099.d
Method: TEST.MS
Sample Name: WYN-51
Analysis Info:

Instrument: LC-MSD-Trap-SL
Operator: Operator

Print Date: 11/16/2010 1:55:30 PM
Acq. Date: 11/16/2010 1:45:14 PM

Acquisition Parameter:

Mass Range Mode	Std/Normal	Trap Drive	45.5	Scan Begin	100 m/z
Ion Polarity	Positive	Octopole RF Amplitude	152.8 Vpp	Scan End	800 m/z
Ion Source Type	ESI	Capillary Exit	-102.3 Volt	Averages	5 Spectra
Dry Temp (Set)	330 °C	Skimmer	-40.0 Volt	Max. Accu Time	200000 µs
Nebulizer (Set)	15.00 psi	Oct 1 DC	-12.00 Volt	ICC Target	20000
Dry Gas (Set)	5.00 l/min	Oct 2 DC	-1.70 Volt	Charge Control	on

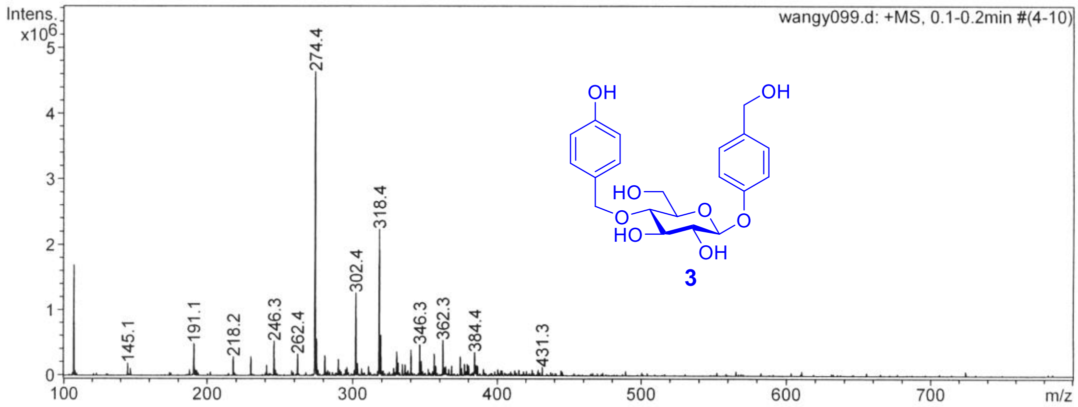

Component	Molecular Mass	Molecule	Absolute Abundance	Relative Abundance

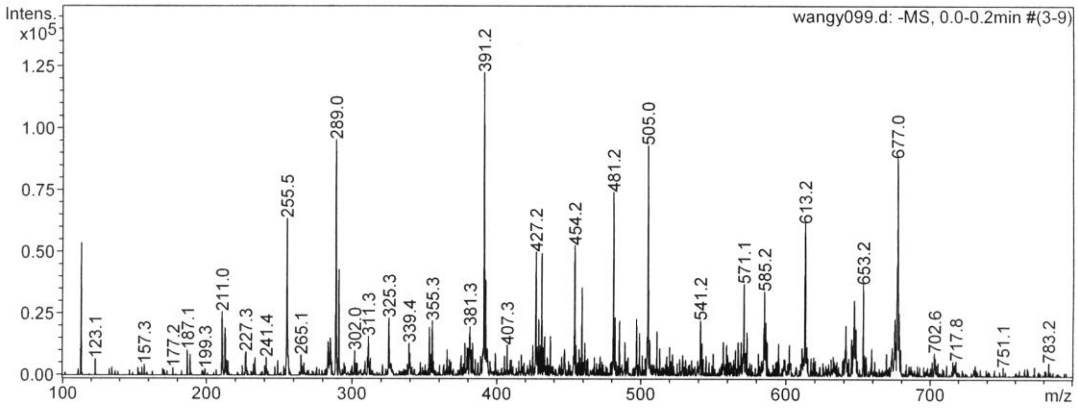

Component	Molecular Mass	Molecule	Absolute Abundance	Relative Abundance

MSD Trap Report v 4 (A4-Opt2) Page 1 of 1 Agilent Technologies

Fig. S26 The ESIMS of compound **3**

Qualitative Analysis Report

**Data Filename**	201109021.d	**Sample Name**	WYN-51
**Sample Type**	Sample	**Position**	P1-F2
**Instrument Name**	Instrument 1	**User Name**	
**Acq Method**		**IRM Calibration Status**	Success
**DA Method**	TEST LCMS.m	**Comment**	

User Chromatograms

Fragmentor Voltage 135 **Collision Energy** 0 **Ionization Mode** ESI

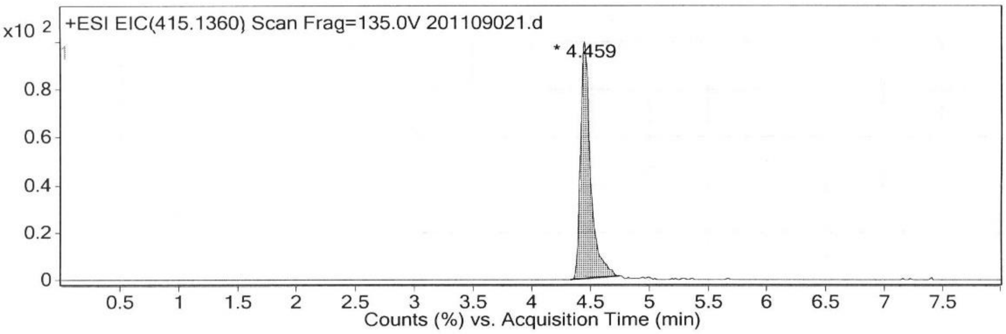

Integration Peak List

Peak	Start	RT	End	Height	Area	Area %
1	4.331	4.459	4.749	202092	1248130	100

User Spectra

**Fragmentor Voltage**	**Collision Energy**	**Ionization Mode**
135	0	Esi

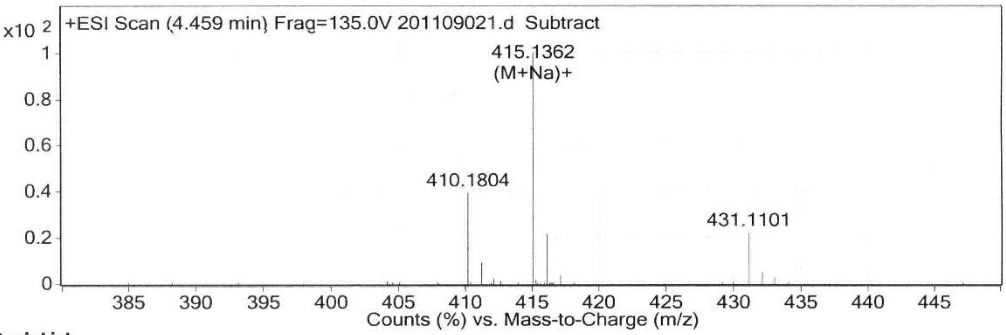

Peak List

m/z	z	Abund	Formula	Ion
101.0036		24153		
104.9926		24903		
107.049		19173		
410.1804	1	79544		
415.1362	1	201661	C20 H24 Na O8	(M+Na)+
416.1391	1	43934	C20 H24 Na O8	(M+Na)+
431.1101		45063		
500.113		32726		
807.2828	1	103034		
808.2858	1	44934		

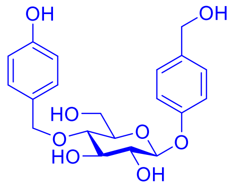

3

Formula Calculator Element Limits

Element	Min	Max
C	3	100
H	0	120
O	0	30

Fig. S27 The (+)-HRESIMS report of compound **3**, Page 1

MS Formula Results: + Scan (4.459 min) Sub (201109021.d)

m/z	Ion	Formula	Abundance
415.1362	(M+Na)+	C20 H24 Na O8	201661.1

Best	Formula (M)	Ion Formula	Calc m/z	Score	Cross S	Mass	Calc Mass	Diff (ppm)	Abs Diff (ppm)	Abund Match	Spacing Mat	Mass Match	m/z	DBE
☑	C20 H24 O8	C20 H24 Na O8	415.1363	99.98		392.1469	392.1471	0.46	0.46	99.98	99.95	99.99	415.1362	9
☐	C21 H20 N4 O4	C21 H20 N4 Na O4	415.1377	99.54		392.1469	392.1485	3.86	3.86	99.14	99.99	99.56	415.1362	14
☐	C24 H24 O3 S	C24 H24 Na O3 S	415.1338	98.31		392.1469	392.1446	-5.92	5.92	96	99.78	98.96	415.1362	13
☐	C15 H24 N2 O10	C15 H24 N2 Na O10	415.1323	98.11		392.1469	392.1431	-9.8	9.8	98.09	99.99	97.19	415.1362	5
☐	C17 H28 O8 S	C17 H28 Na O8 S	415.1397	97.87		392.1469	392.1505	9.05	9.05	96.79	99.7	97.6	415.1362	4
☐	C12 H28 N2 O10 S	C12 H28 N2 Na O10 S	415.1357	97.58		392.1469	392.1465	-1.21	1.21	91.9	99.66	99.96	415.1362	0
☐	C21 H28 O3 S2	C21 H28 Na O3 S2	415.1372	97.39		392.1469	392.148	2.67	2.67	91.62	99.54	99.79	415.1362	8
☐	C16 H28 N2 O5 S2	C16 H28 N2 Na O5 S2	415.1332	96.43		392.1469	392.144	-7.6	7.6	90.77	99.47	98.3	415.1362	4

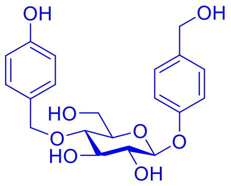

3

page 1

Fig. S28 The (+)-HRESIMS report of compound **3**, Page 2

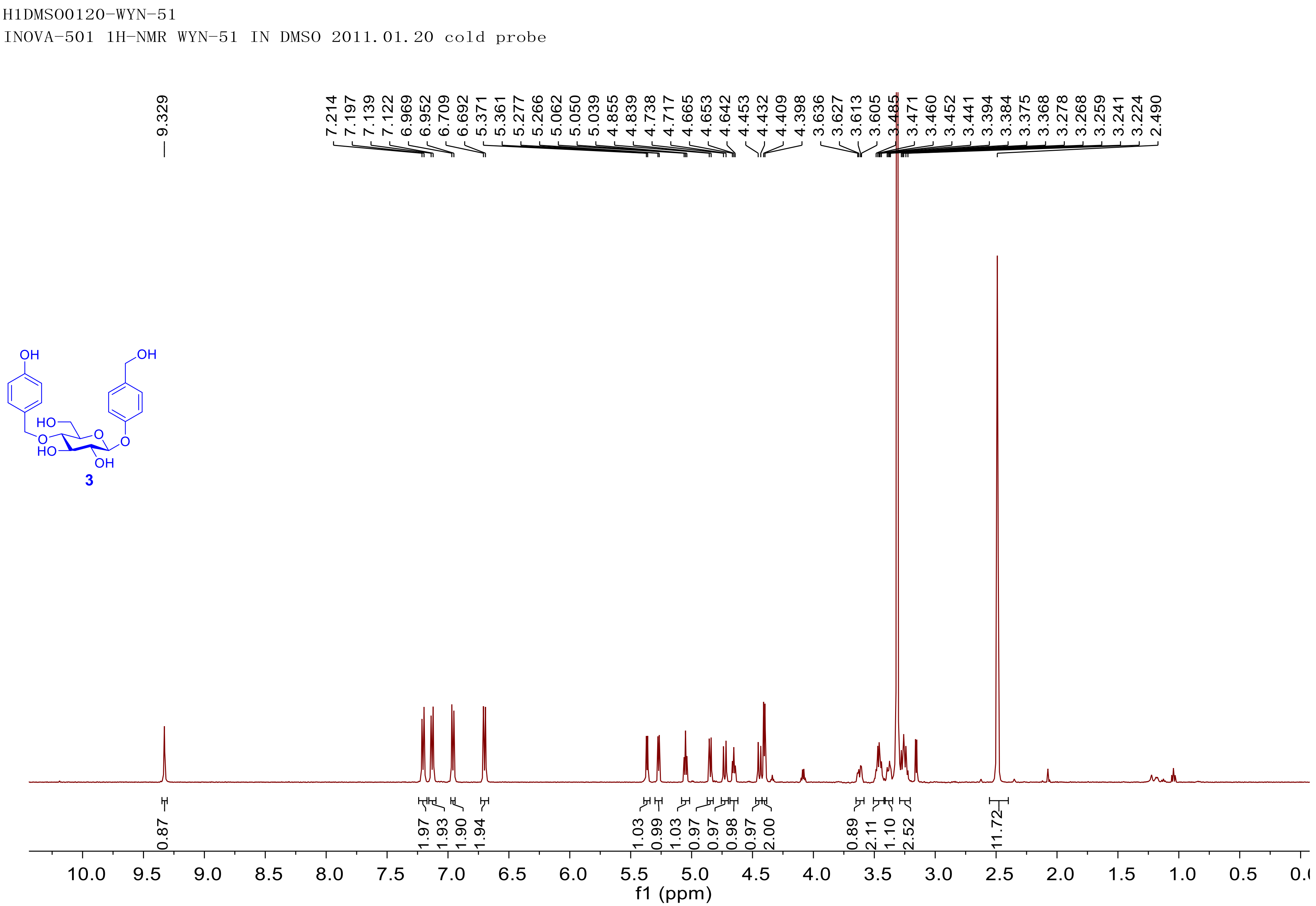

Fig. S29 The ^{1}H NMR spectrum of compound **3** in DMSO-d_6 at 500 MHz

C13DMSO0107-WYN-51
INOVA-501 13C-NMR WYN-51 IN DMSO 2011.01.07 cold probe

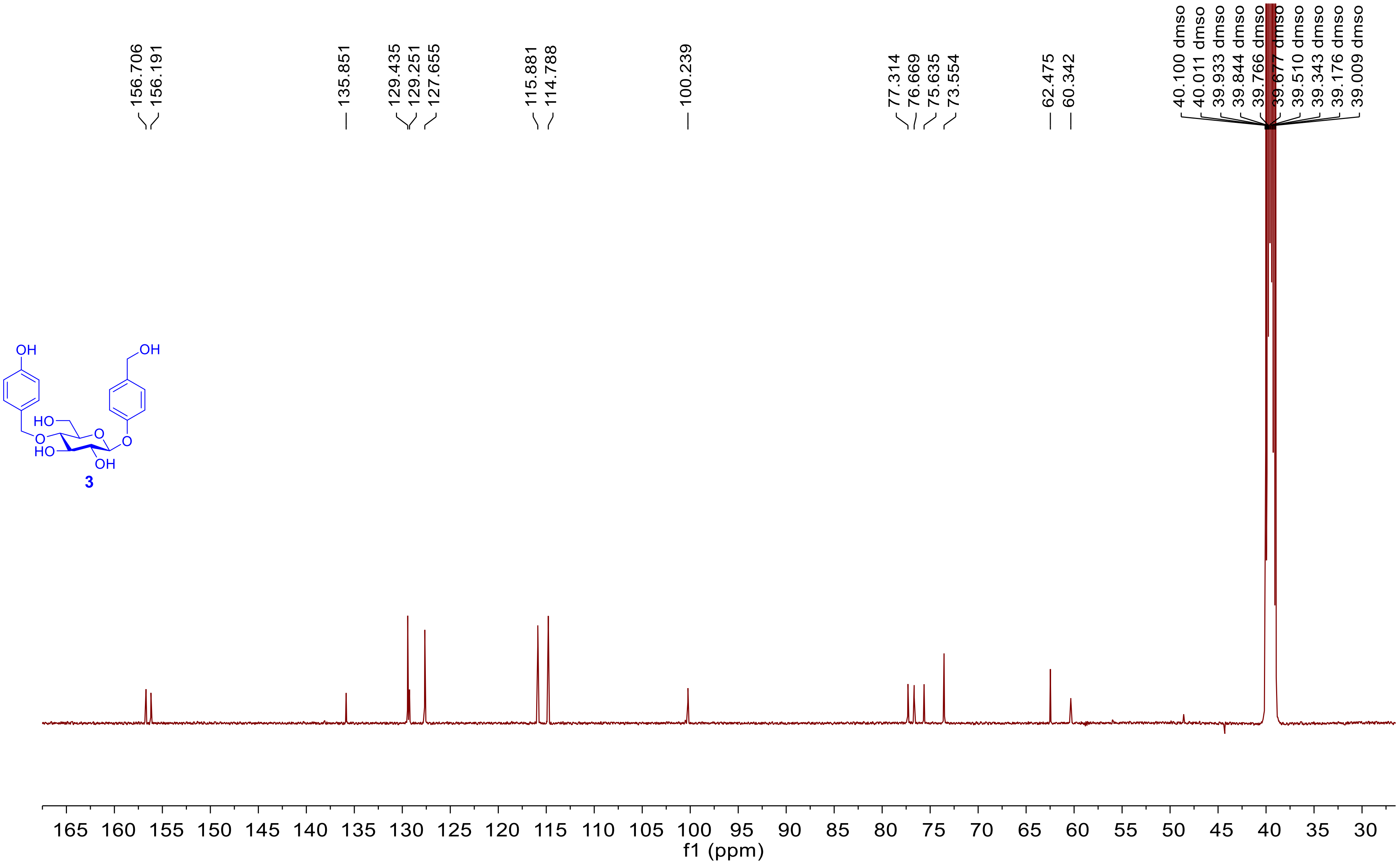

3

Fig. S30 The ^{13}C NMR spectrum of compound **3** in DMSO-d_6 at 125 MHz

C13DMSO0107-WYN-51
INOVA-501 DEPT WYN-51 IN DMSO 2011.01.07 cold probe

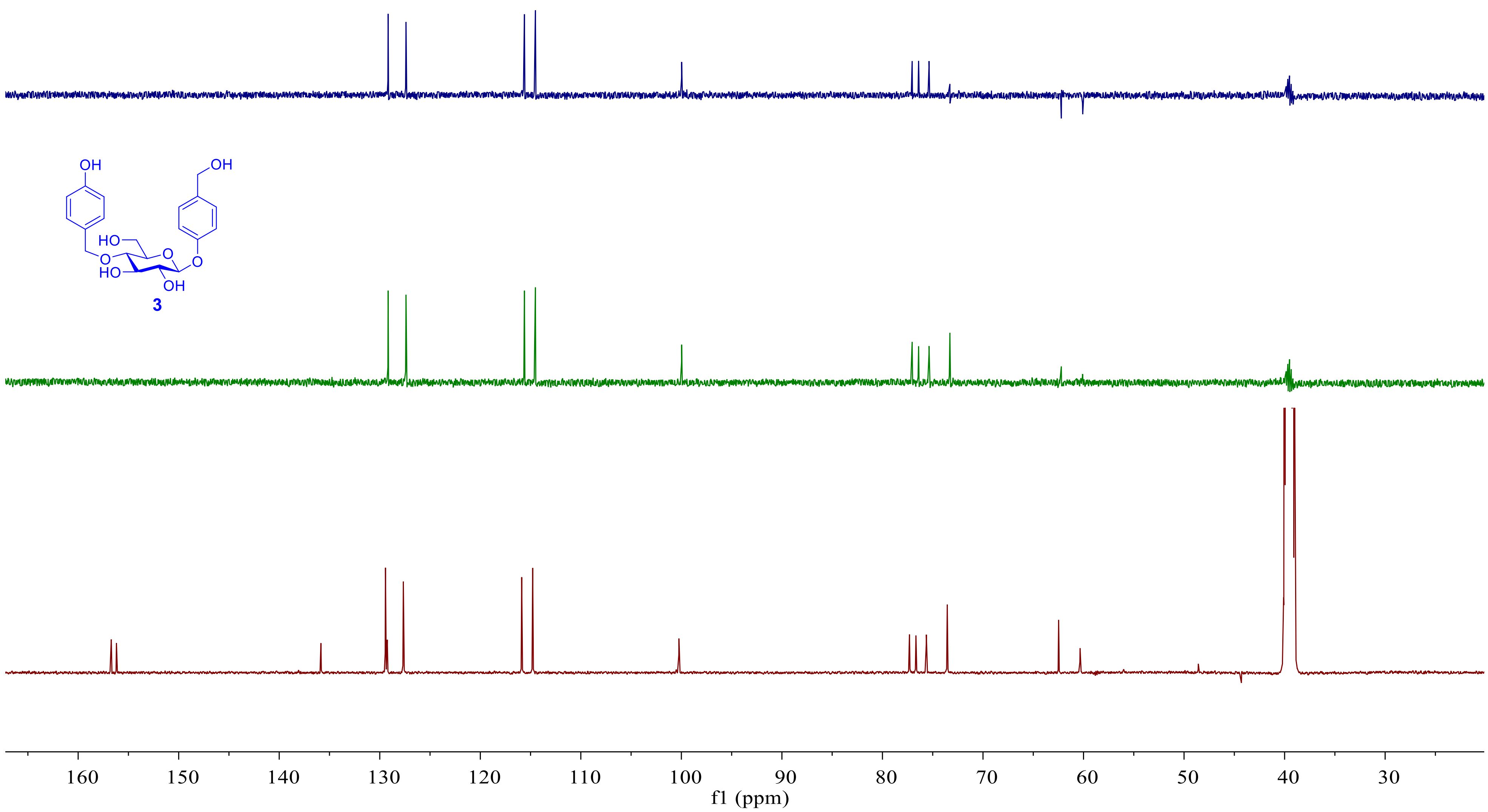

Fig. S31 The DEPT spectrum of compound **3** in DMSO-d_6 at 125 MHz

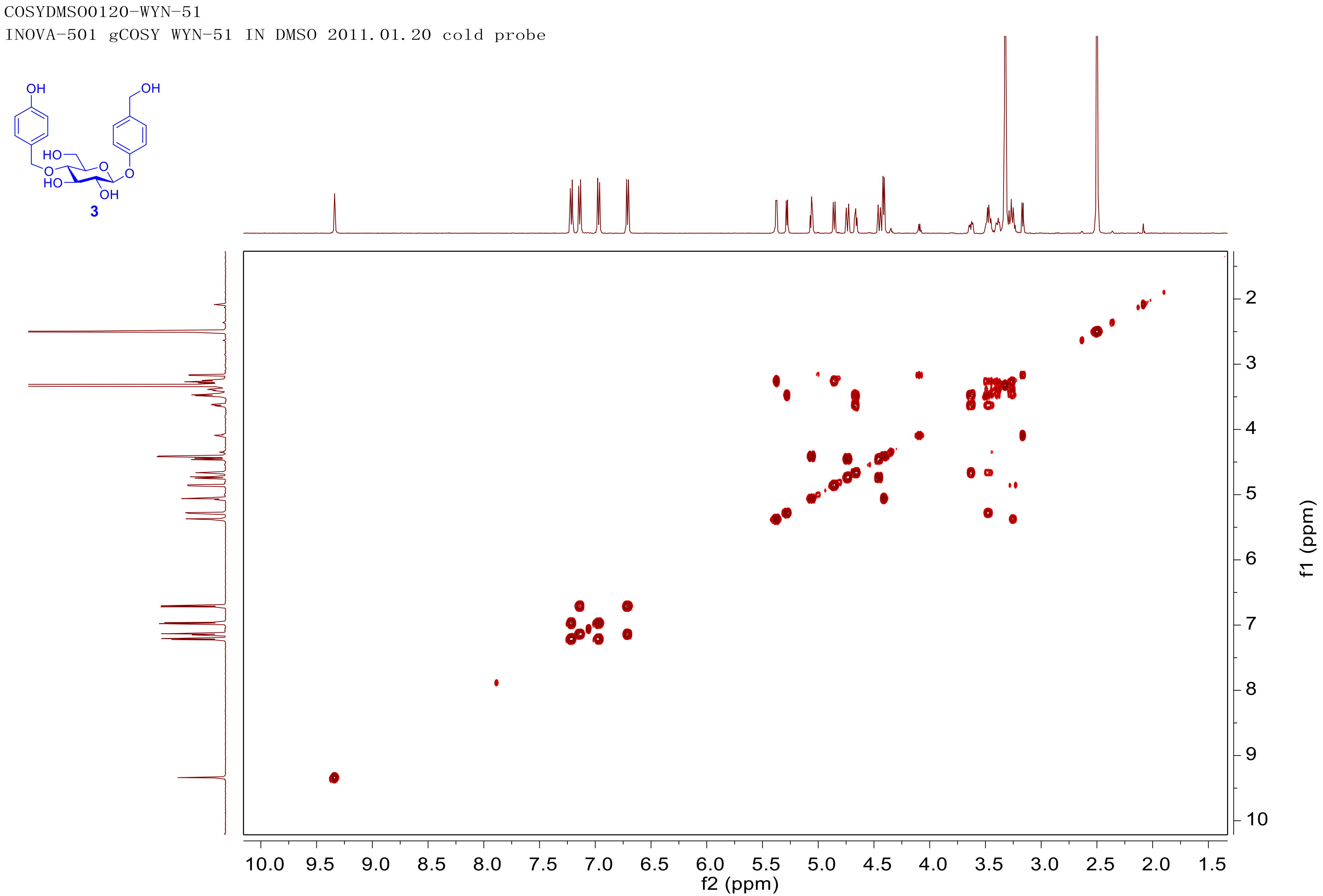

Fig. S32 The ^{1}H-^{1}H COSY spectrum of compound **3** in DMSO-d_6 at 500 MHz

HMQCDMSO0120-WYN-51
INOVA-501 gHMQC WYN-51 IN DMSO 2011.01.20 cold probe

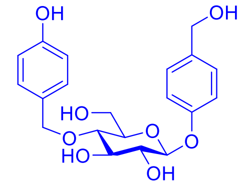

3

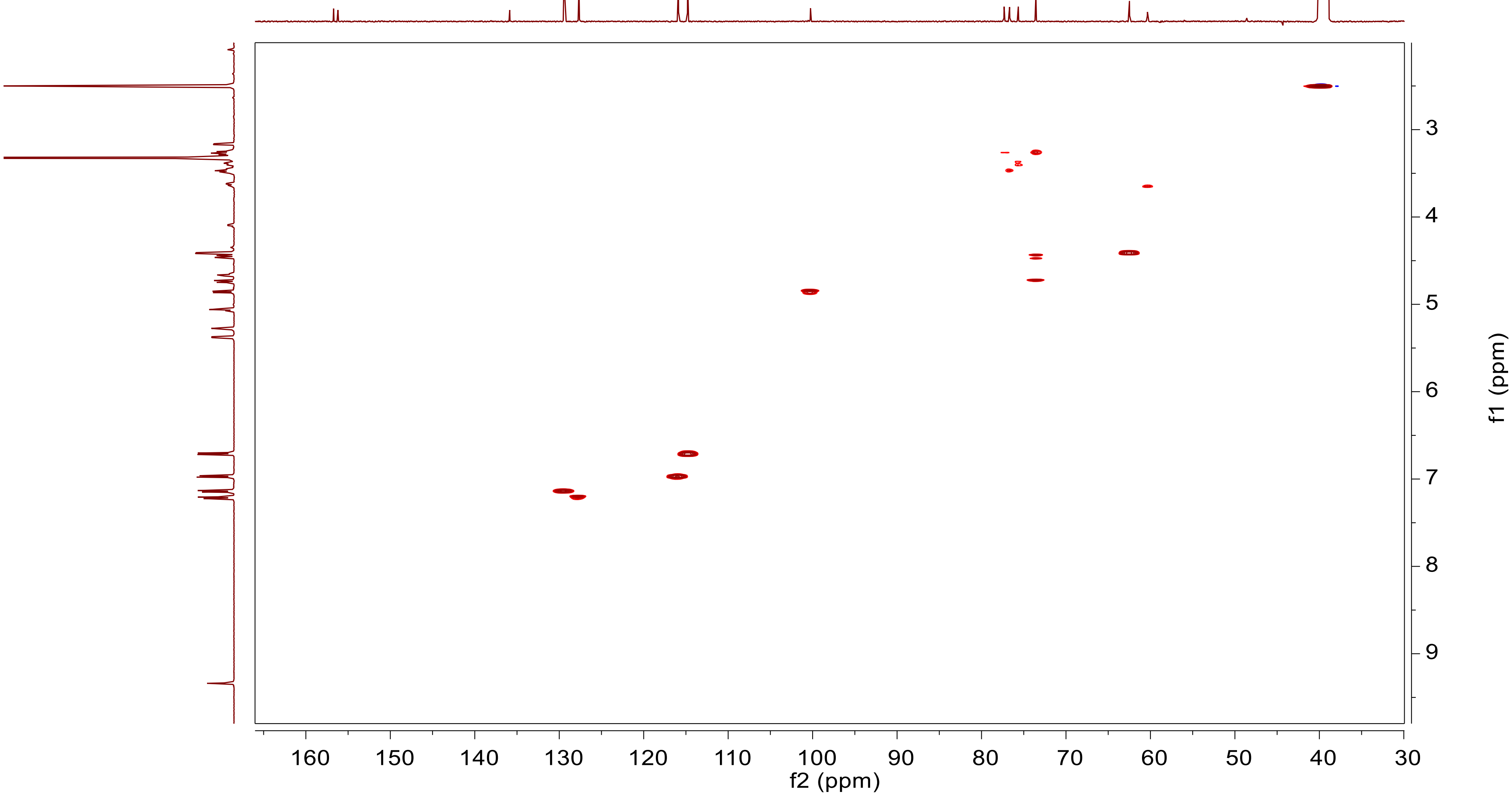

Fig. S33 The HMQC spectrum of compound **3** in DMSO-d_6 (500 MHz for ^{1}H)

HMBCDMSO0120-WYN-51
INOVA-501 gHMBC WYN-51 IN DMSO 2011.01.20 cold probe

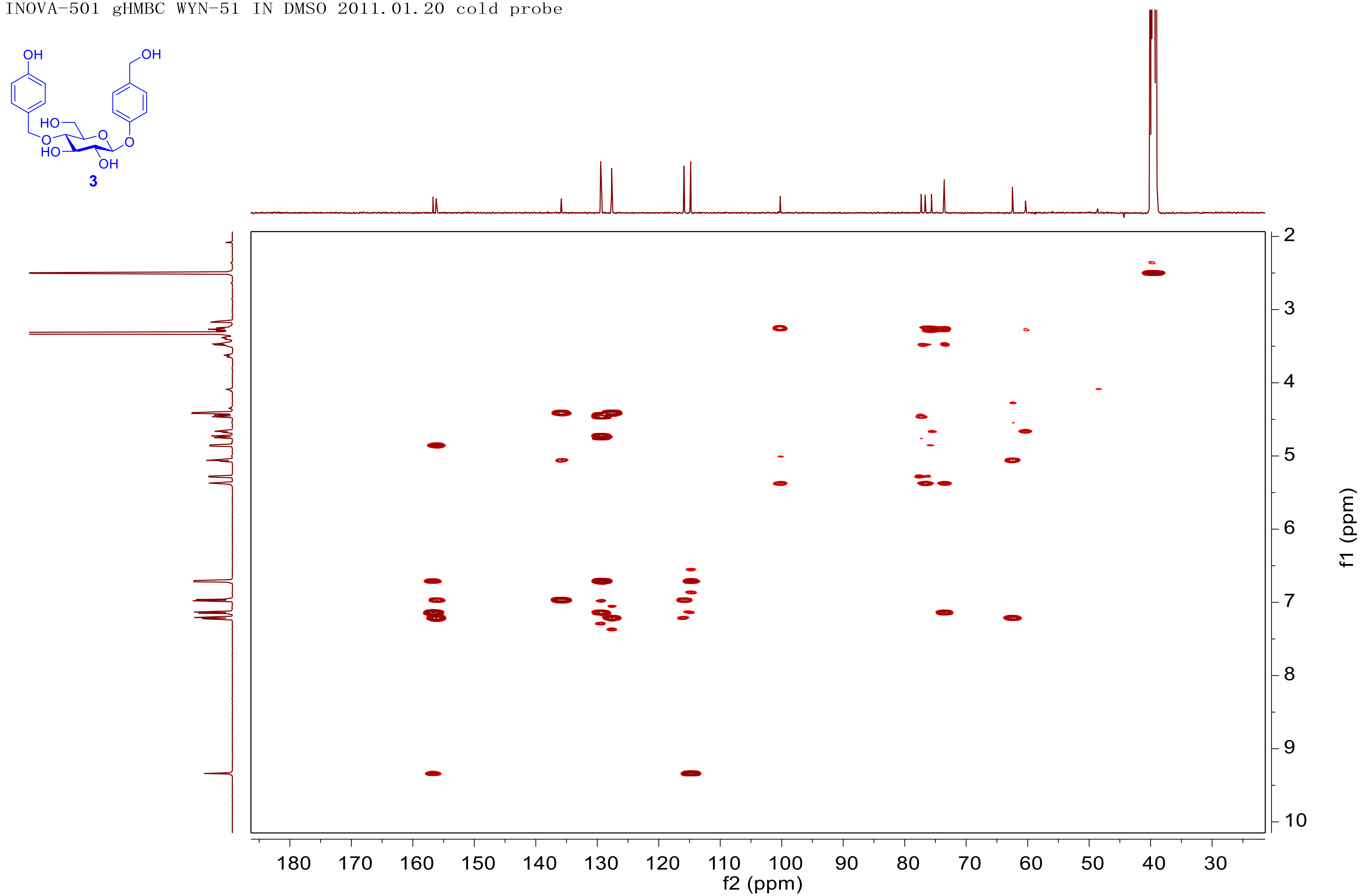

Fig. S34 The HMBC spectrum of compound **3** in DMSO-d_6 (500 MHz for ^1H)

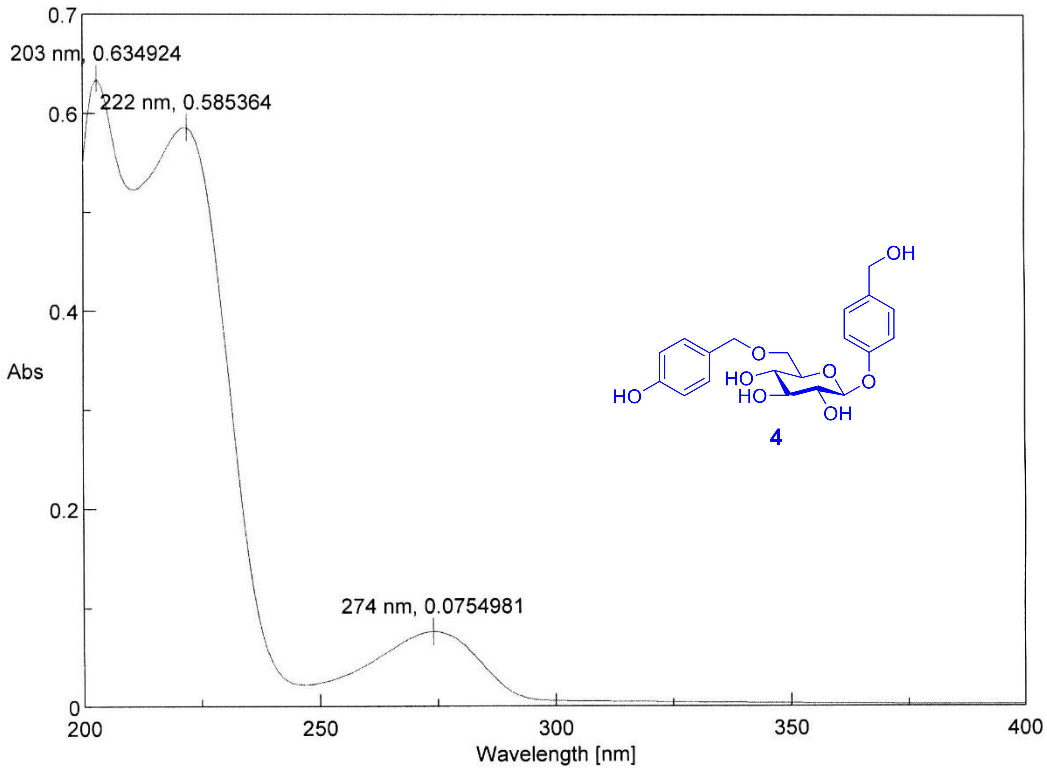

[Comment]
Sample Name wyn-52
Comment
User 王亚男
Division
Company 324

wyn-52-2-w

[Measurement Information]

Instrument Name	V-650
Model Name	V-650
Serial No.	A034461150
Accessory	PSC-718
Accessory S/N	A001761114
Position	1
Cell Length	10 mm
Temperature	19.98 C
Control Sensor	Holder
Monitor Sensor	Holder
Start Mode	Start immediately
Photometric Mode	Abs
Measurement range	400 - 190 nm
Data pitch	1 nm
Band width(UV/Vis)	1.0 nm
Response	Medium
Scanning speed	200 nm/min
Source Change	340 nm
Light Source	D2/WI
Filter Exchange	Step
Correction	Baseline

[Data Information]

Creation Date	2011-5-11 18:08
Data array type	Linear data array
Horizontal	Wavelength [nm]
Vertical	Abs
Start	400 nm
End	190 nm
Data pitch	1 nm
Data points	211

Fig. S35 The UV spectrum of compound **4**

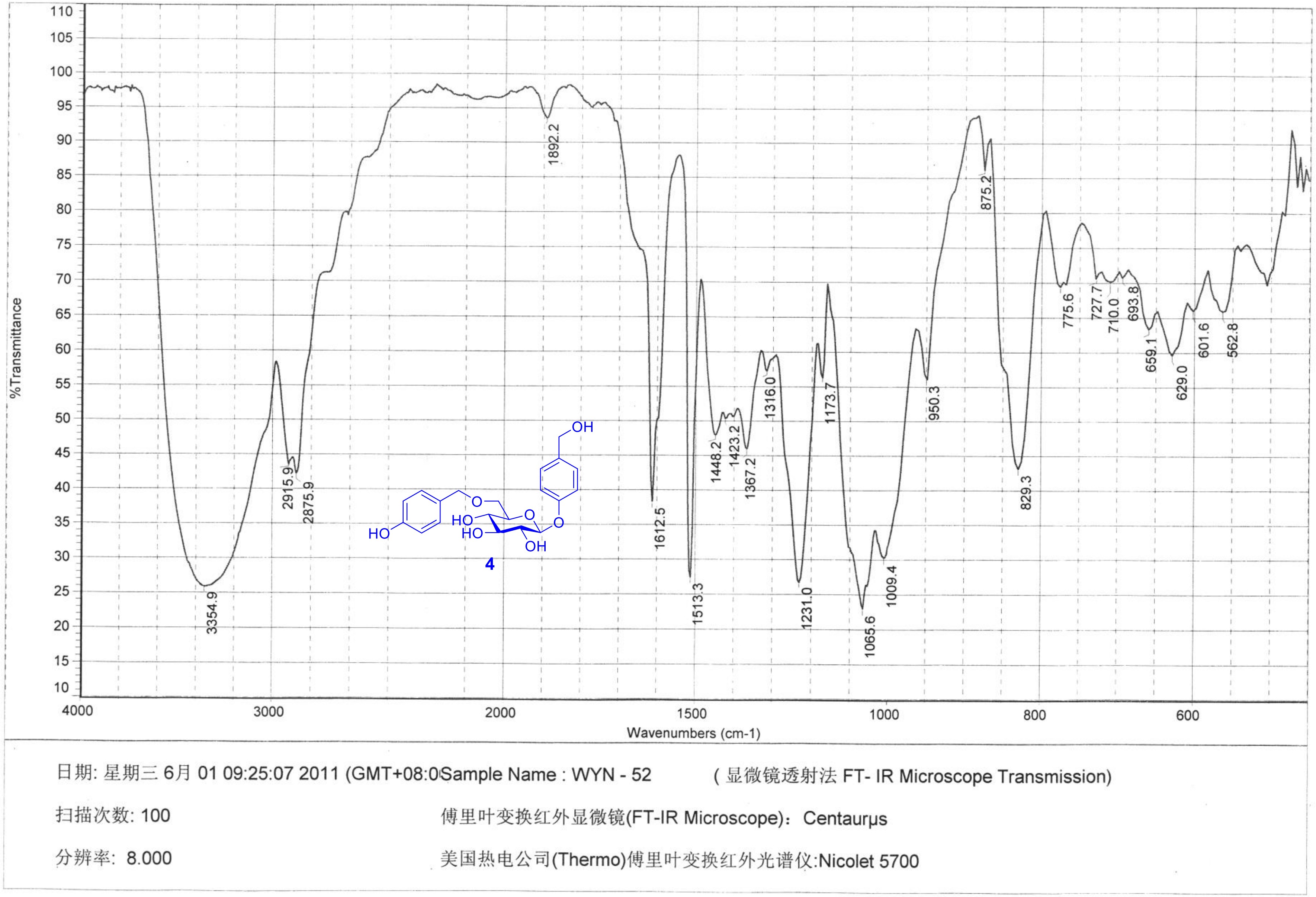

Fig. S36 The IR spectrum of compound **4**

Single Mass Spectrum Deconvolution Report

Analysis Name: WANGY014.d **Instrument:** LC-MSD-Trap-SL **Print Date:** 7/9/2010 12:07:58 PM
Method: TEST.MS **Operator:** Operator **Acq. Date:** 7/9/2010 12:06:39 PM
Sample Name: WYN-52
Analysis Info:

Acquisition Parameter:

Mass Range Mode	Std/Normal	Trap Drive	53.0	Scan Begin	100 m/z
Ion Polarity	Positive	Octopole RF Amplitude	171.0 Vpp	Scan End	700 m/z
Ion Source Type	ESI	Capillary Exit	-106.0 Volt	Averages	5 Spectra
Dry Temp (Set)	330 °C	Skimmer	-40.0 Volt	Max. Accu Time	200000 µs
Nebulizer (Set)	15.00 psi	Oct 1 DC	-12.00 Volt	ICC Target	20000
Dry Gas (Set)	5.00 l/min	Oct 2 DC	-1.70 Volt	Charge Control	on

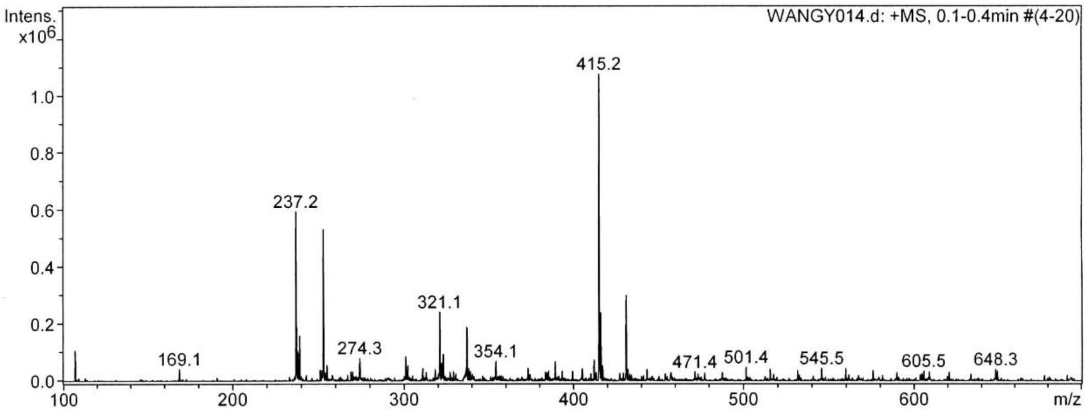

Component	Molecular Mass	Molecule	Absolute Abundance	Relative Abundance

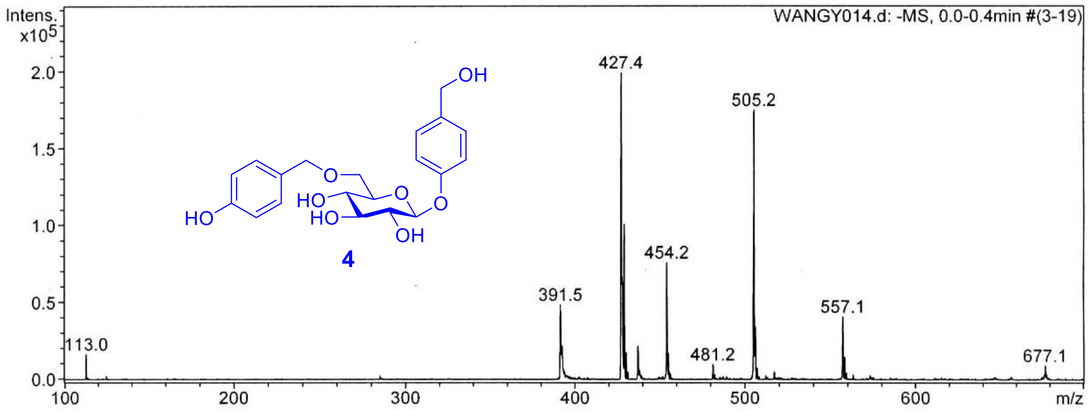

Component	Molecular Mass	Molecule	Absolute Abundance	Relative Abundance

Fig. S37 The ESIMS of compound **4**

Qualitative Analysis Report

**Data Filename**	201101173.d	**Sample Name**	WYN-52
**Sample Type**	Sample	**Position**	P1-C3
**Instrument Name**	Instrument 1	**User Name**	
**Acq Method**		**IRM Calibration Status**	Success
**DA Method**	TEST LCMS.m	**Comment**	

User Chromatograms

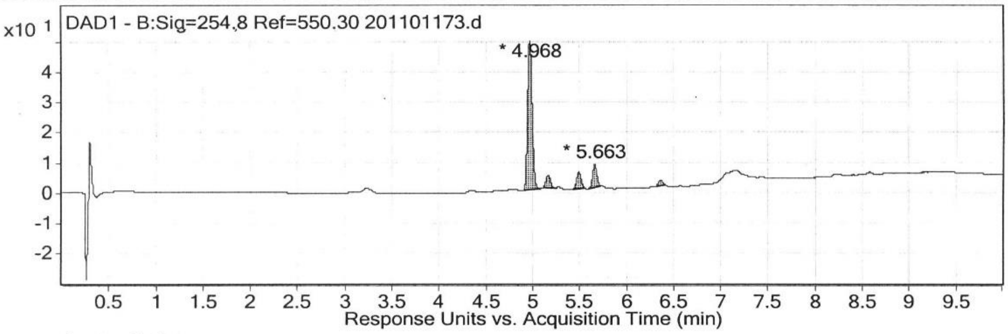

Integration Peak List

Peak	Start	RT	End	Height	Area	Area %
1	4.905	4.968	5.068	49.13	165.6	100
2	5.114	5.167	5.254	4.09	12.97	7.83
3	5.428	5.493	5.556	5.53	18.16	10.97
4	5.603	5.663	5.73	7.56	23.73	14.33
5	6.323	6.363	6.415	1.88	5.52	3.33

Fragmentor Voltage 135 **Collision Energy** 0 **Ionization Mode** ESI

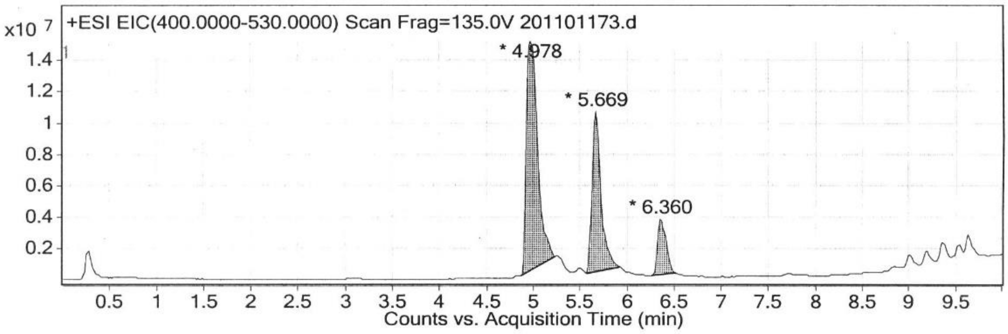

Integration Peak List

Peak	Start	RT	End	Height	Area	Area %
1	4.866	4.978	5.235	14548216	121611483	100
2	5.573	5.669	5.926	10125505	69387570	57.06
3	6.264	6.36	6.521	3470173	21547589	17.72

User Spectra

Fragmentor Voltage	Collision Energy	Ionization Mode
135	0	Esi

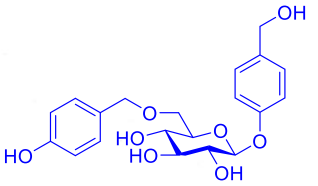

4

Fig. S38 The (+)-HRESIMS report of compound **4**, Page 1

Qualitative Analysis Report

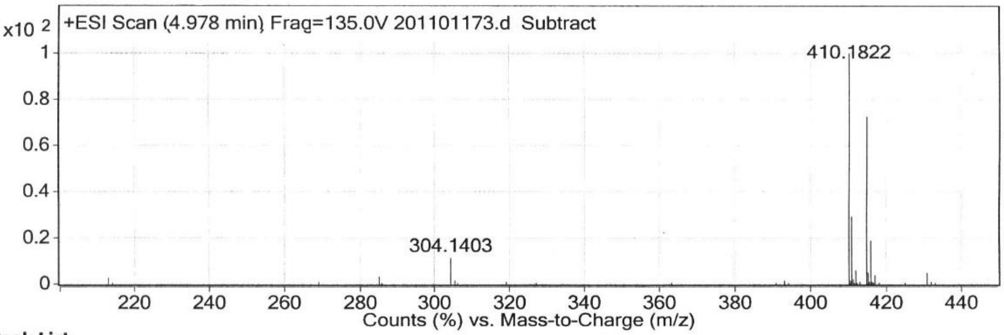

Peak List

m/z	z	Abund	Formula	Ion
304.1403		614777		
410.1822	1	5302159		
410.3524		372484		
411.1853	1	1562264		
412.1873	1	309604		
415.1375	1	3853534	C20 H24 Na O8	(M+Na)+
416.1415	1	1004769	C20 H24 Na O8	(M+Na)+
807.2851	1	1168622		
808.2891	1	558351		

Formula Calculator Element Limits

Element	Min	Max
C	3	100
H	0	120
O	0	30
N	0	3
S	0	0
Cl	0	0

Formula Calculator Results

Formula	Best	Mass	Tgt Mass	Diff (ppm)	Ion Species	Score
C20 H24 O8	TRUE	392.1483	392.1471	-2.9	C20 H24 Na O8	99.43

--- End Of Report ---

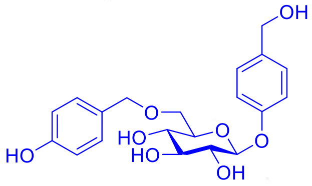

4

Fig. S39 The (+)-HRESIMS report of compound **4**, Page 2

MS Formula Results: + Scan (4.978 min) Sub (201101173.d)

m/z	Ion	Formula	Abundance
415.1375	(M+Na)+	C20 H24 Na O8	3853533.8

Best	Formula (M)	Ion Formula	Calc m/z	Score	Cross S	Mass	Calc Mass	Diff (ppm)	Abs Diff (ppm)	Abund Match	Spacing Mat	Mass Match	m/z	DBE
☑	C20 H24 O8	C20 H24 Na O8	415.1363	99.43		392.1483	392.1471	-2.9	2.9	98.46	99.93	99.75	415.1375	9

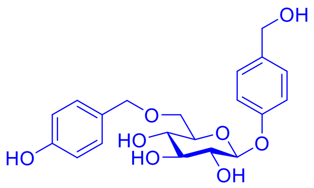

4

page 1

Fig. S40 The (+)-HRESIMS report of compound **4**, Page 3

H1DMSO1227-WYN-52
INOVA-501 1H-NMR WYN-52 IN DMSO 2010.12.24 cold probe

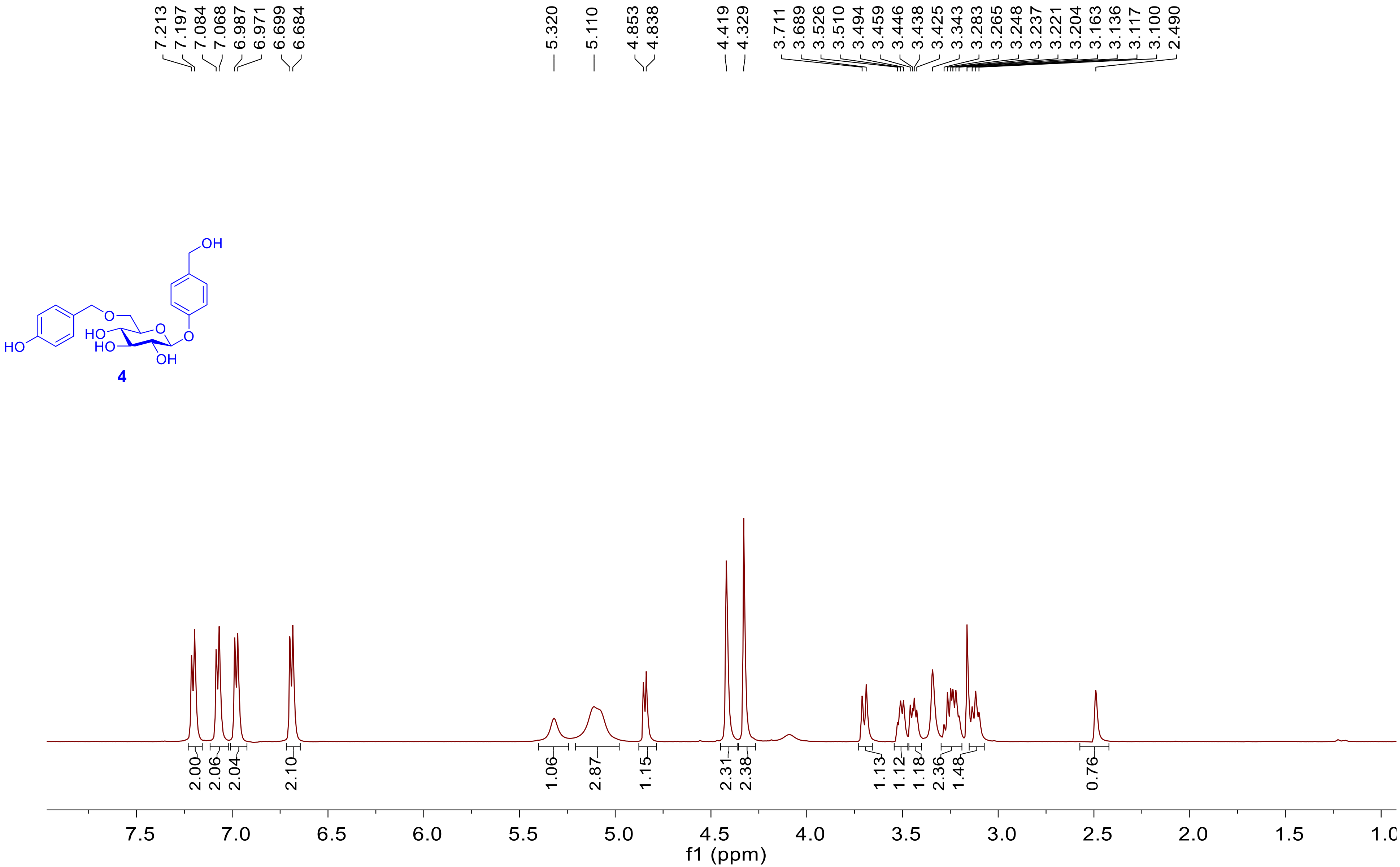

Fig. S41 The ^{1}H NMR spectrum of compound **4** in DMSO-d_6 at 500 MHz

C13DMSO1203-WYN-52
INOVA-501 13C-NMR WYN-52 IN DMSO 2010.12.03

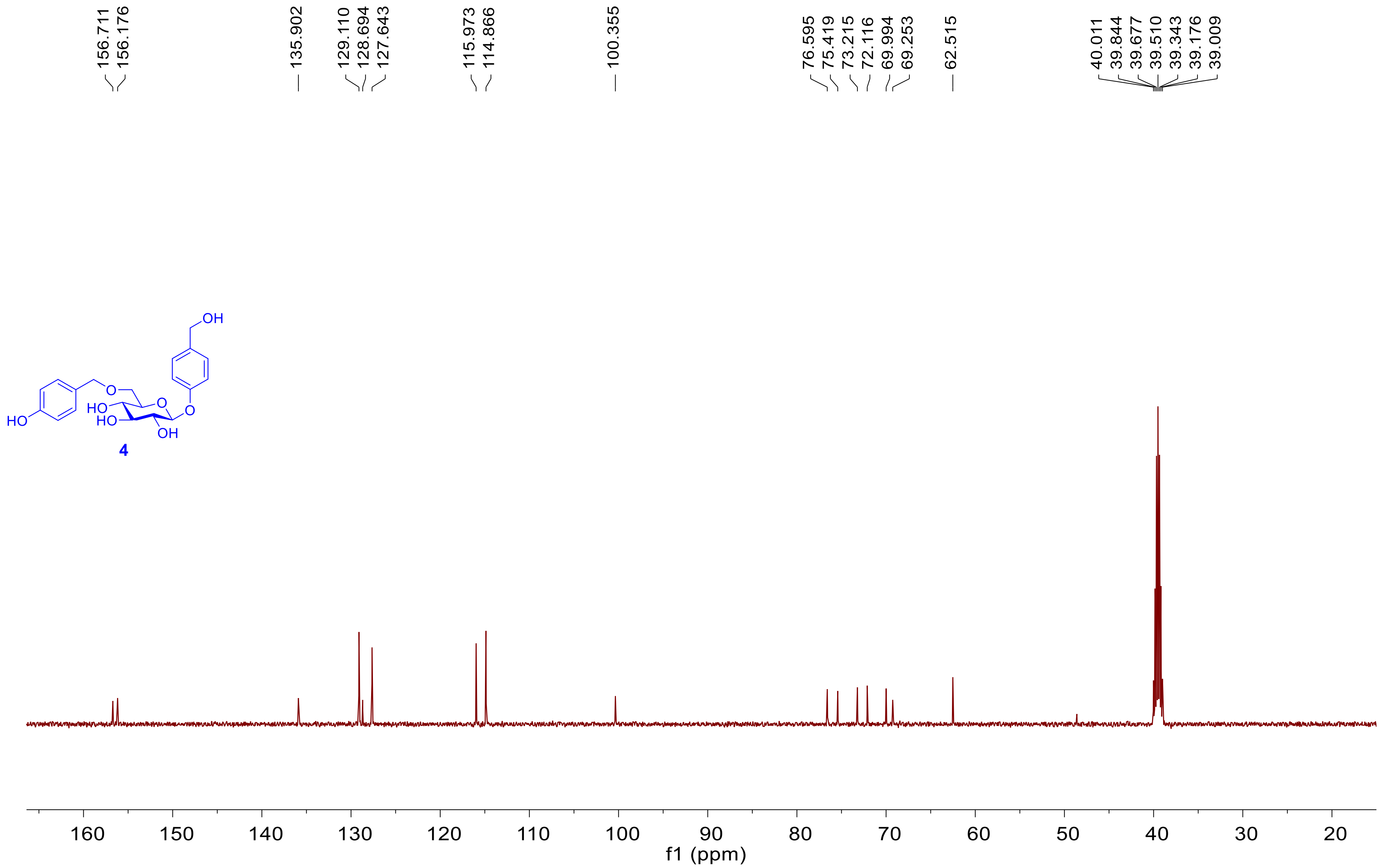

Fig. S42 The ^{13}C NMR spectrum of compound **4** in DMSO-d_6 at 125 MHz

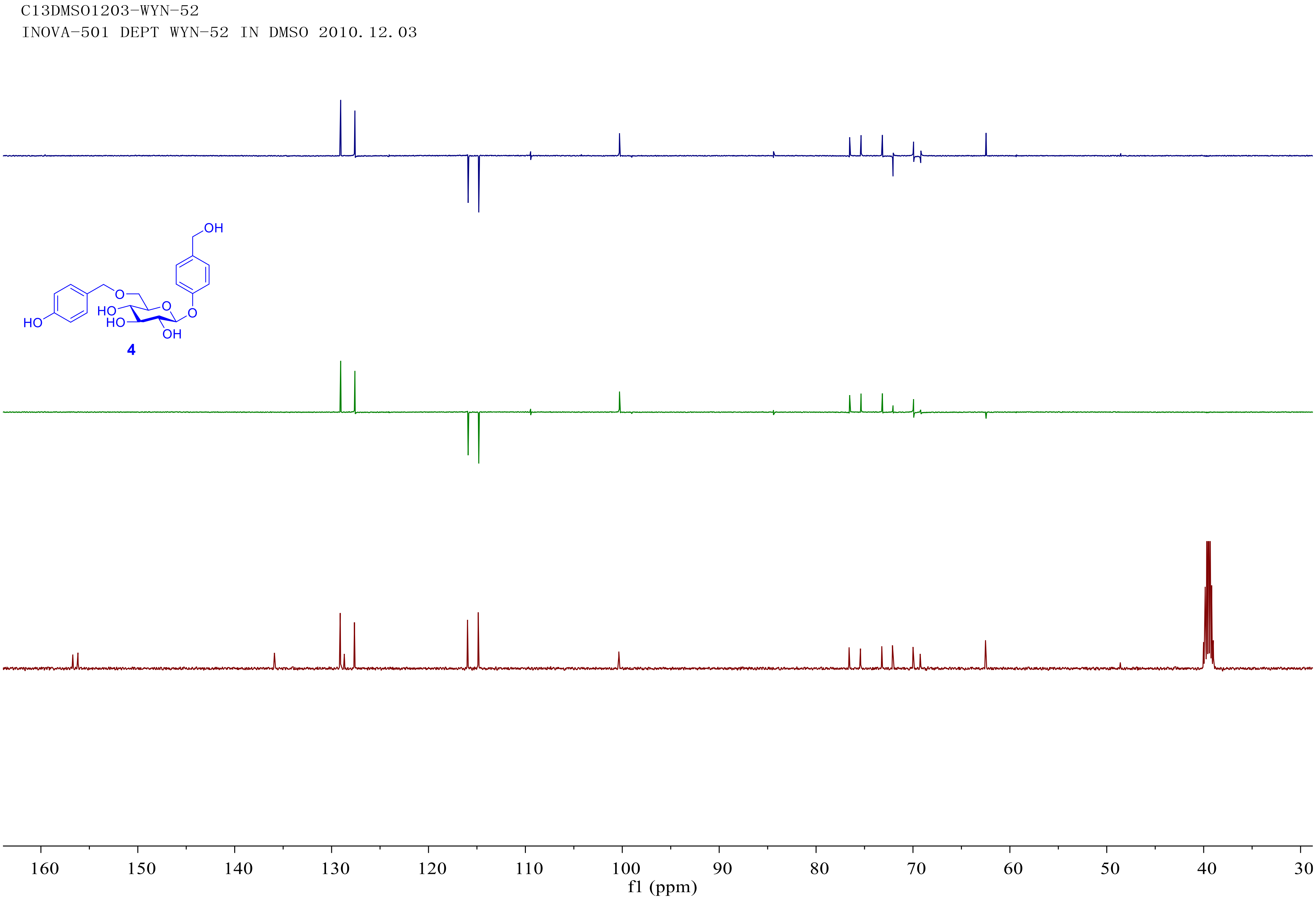

Fig. S43 The DEPT spectrum of compound **4** in DMSO-d_6 at 125 MHz

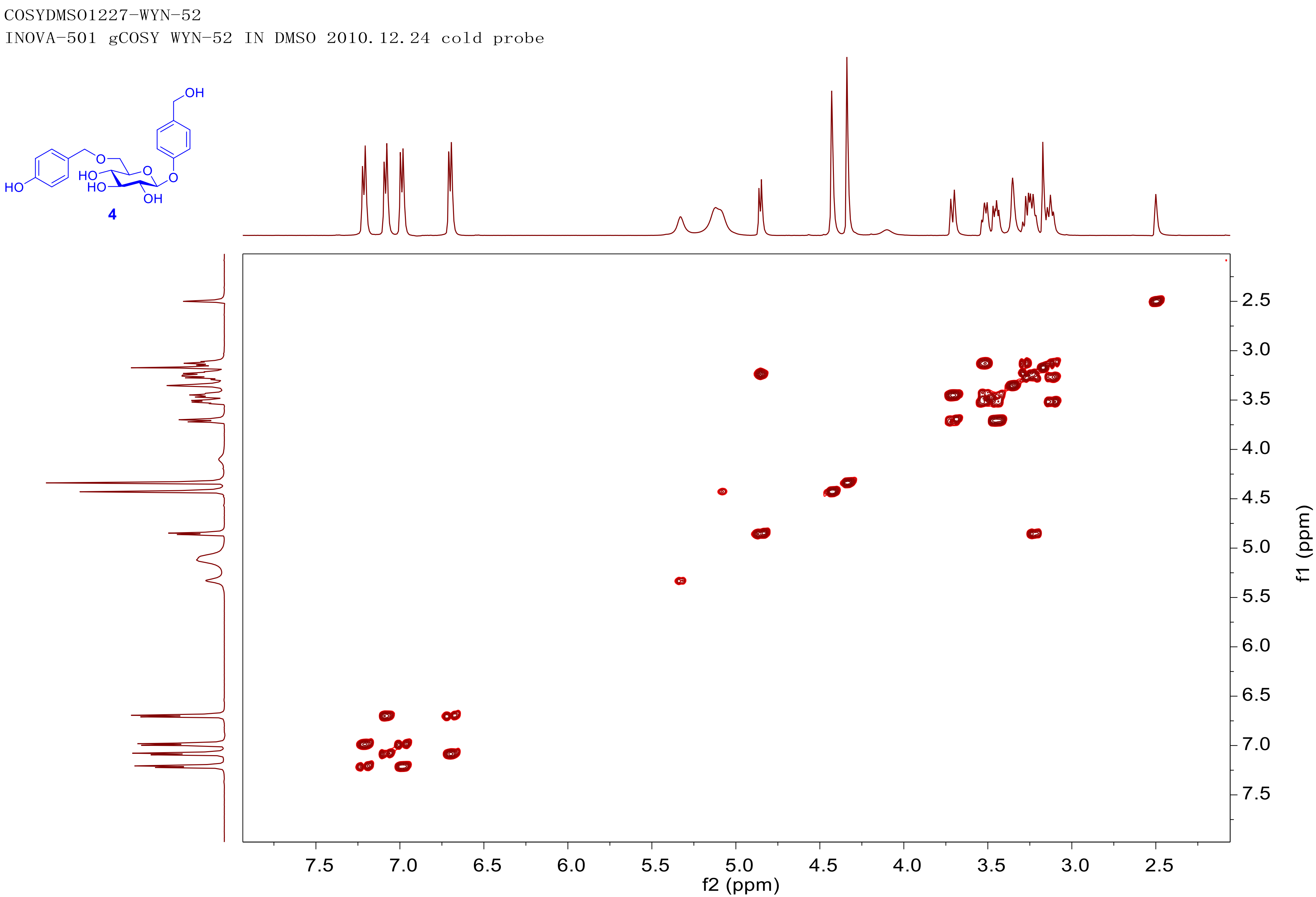

Fig. S44 The ^{1}H-^{1}H COSY spectrum of compound **4** in DMSO-d_6 at 500 MHz

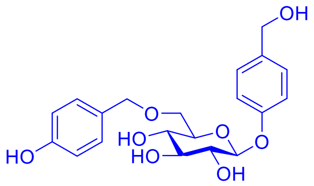

4

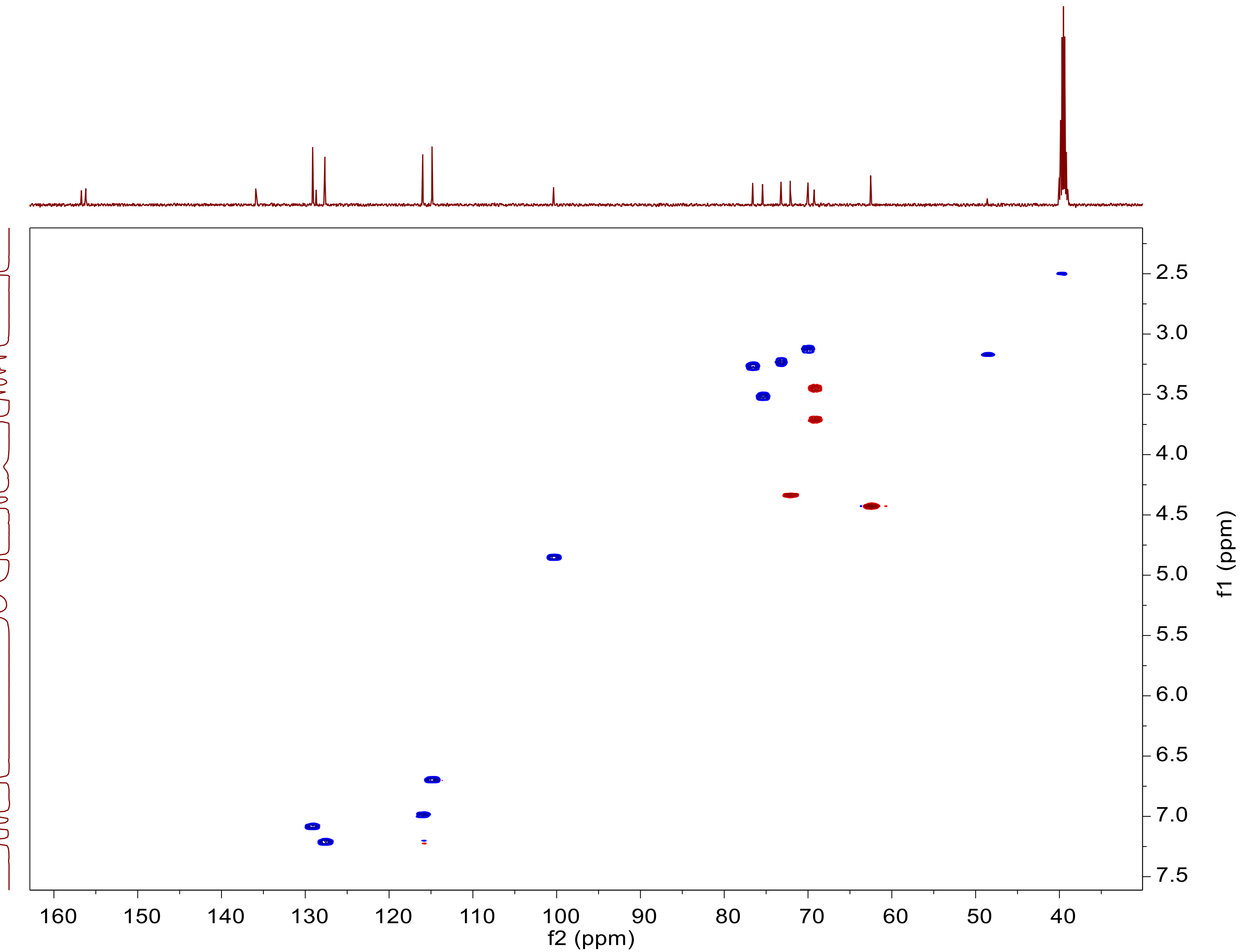

Fig. S45 The HSQC spectrum of compound **4** in DMSO-d_6 (500 MHz for ^{1}H)

HMBCDMSO1227-WYN-52
INOVA-501 gHMBC WYN-52 IN DMSO 2010.12.24 cold probe

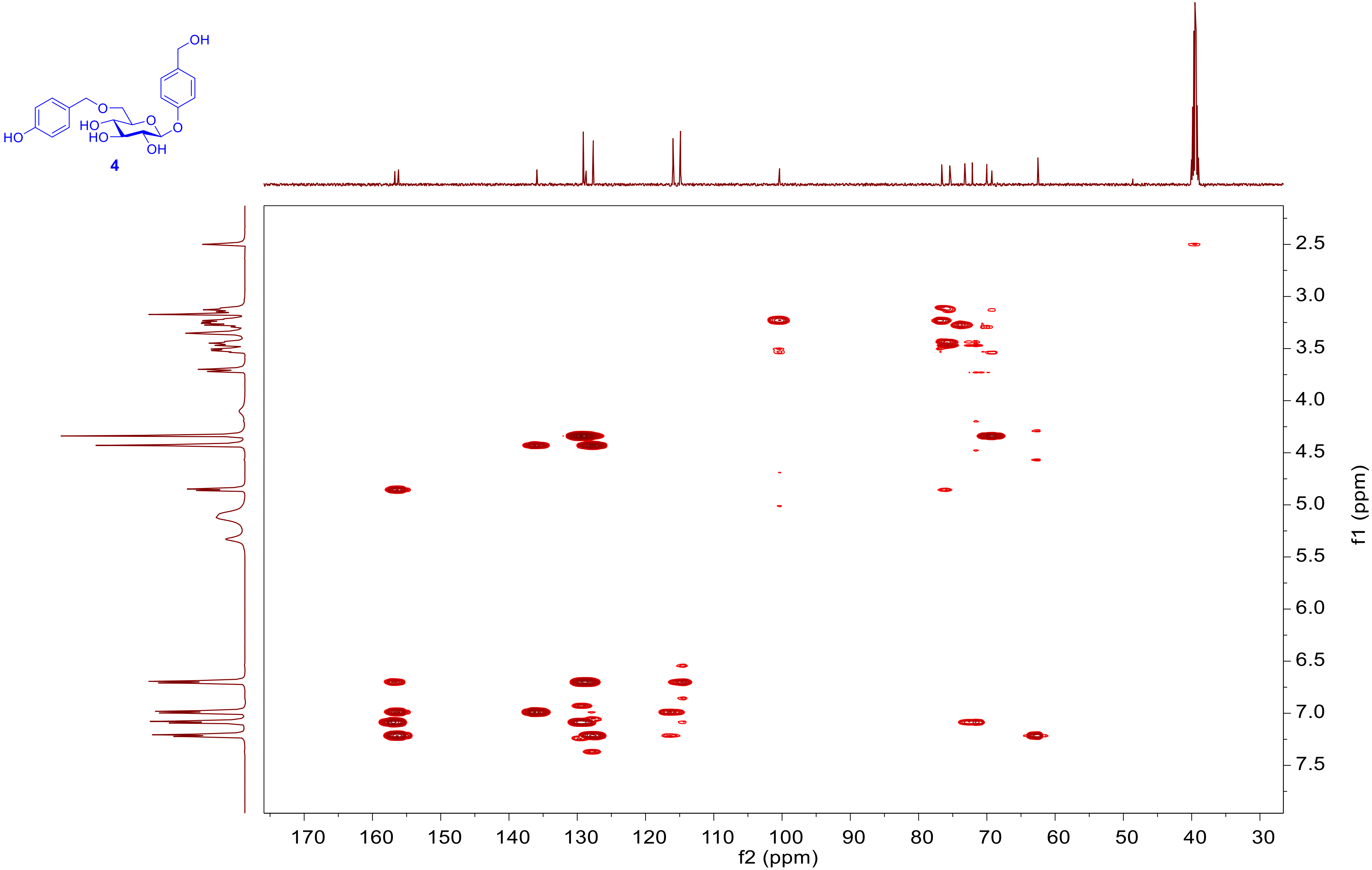

4

Fig. S46 The HMBC spectrum of compound **4** in DMSO-d_6 (500 MHz for ^{1}H)

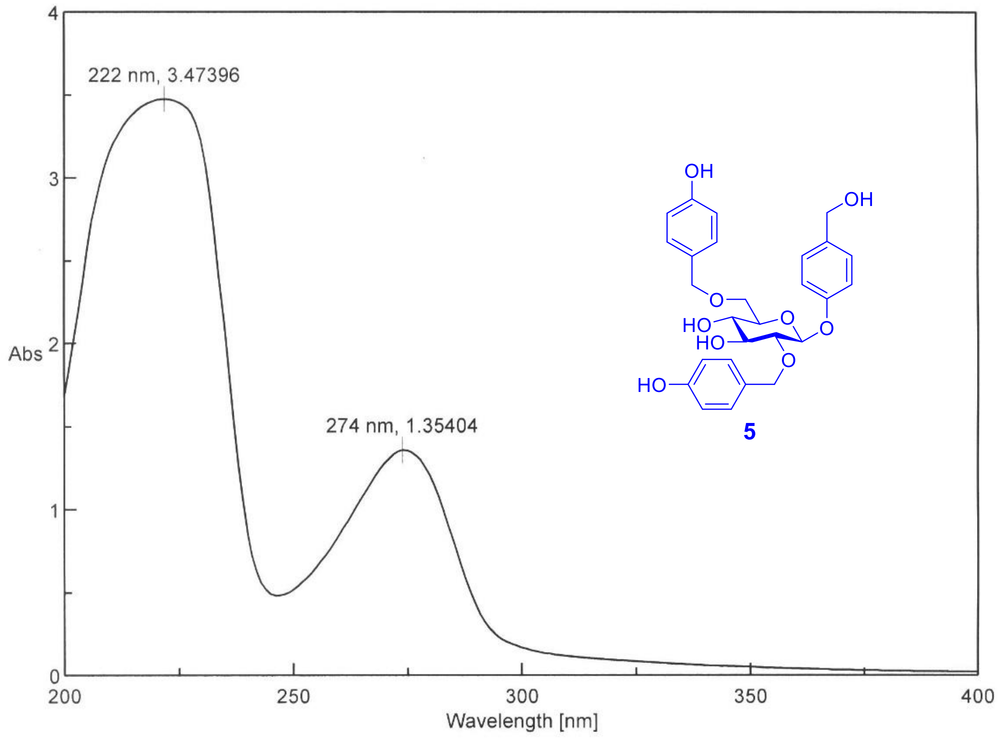

Memory-7

[Comment]	
Sample Name	WYN-74
Comment	
User	wangyanan
Division	
Company	324
[Measurement Information]	
Instrument Name	V-650
Model Name	V-650
Serial No.	A034461150
Accessory	PSC-718
Accessory S/N	A001761114
Position	1
Cell Length	10 mm
Temperature	19.98 C
Control Sonsor	Holder
Monitor Sensor	Holder
Start Mode	Start immediately
Photometric Mode	Abs
Measurement range	400 - 200 nm
Data pitch	1 nm
Band width(UV/Vis)	1.0 nm
Response	Medium
Scanning speed	200 nm/min
Source Change	340 nm
Light Source	D2/WI
Filter Exchange	Step
Correction	Baseline

[Data Information]	
Creation Date	2011-11-23 11:05
Data array type	Linear data array
Horizontal	Wavelength [nm]
Vertical	Abs
Start	400 nm
End	200 nm
Data pitch	1 nm
Data points	201

Fig. S47 The UV spectrum of compound **5**

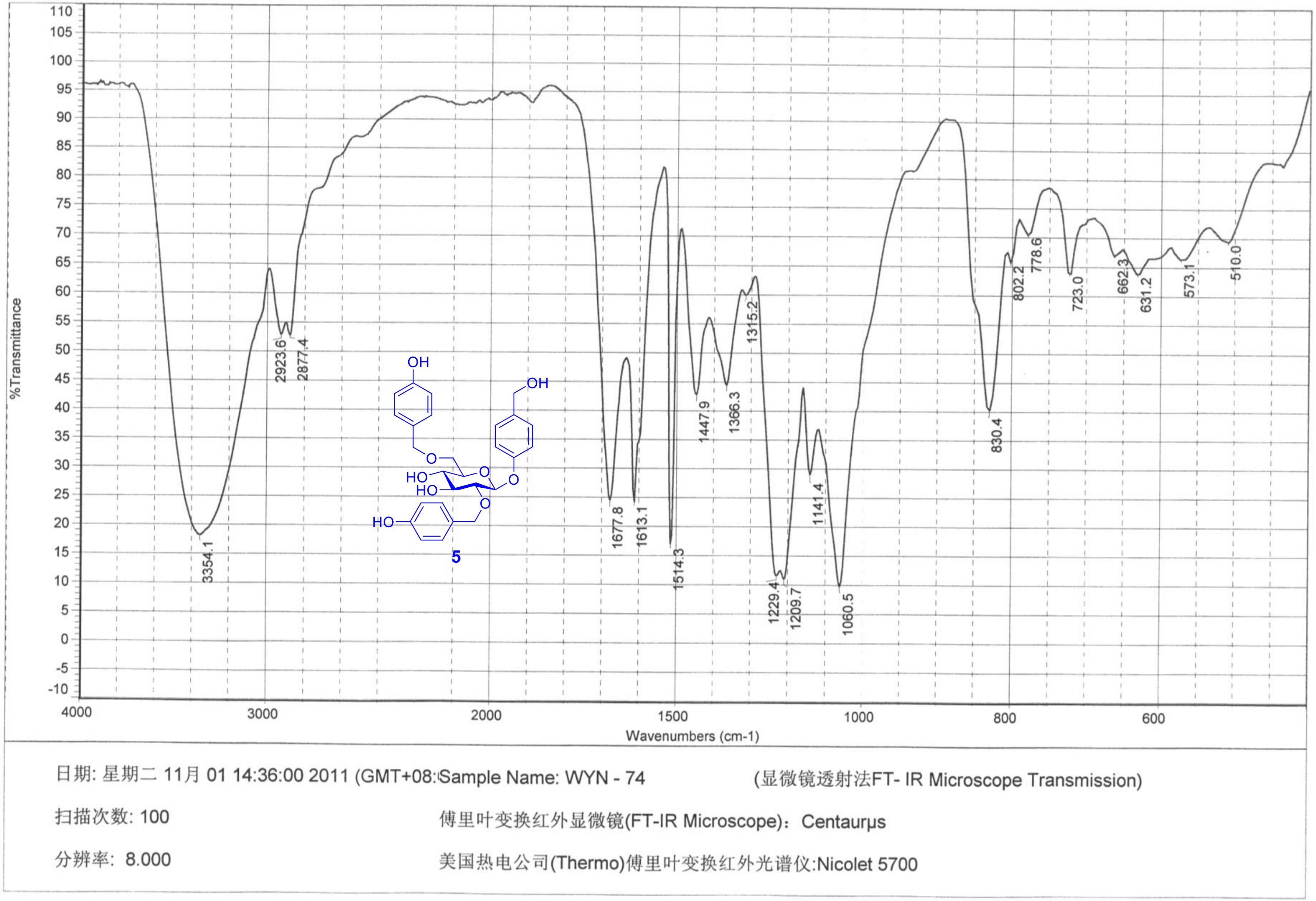

Fig. S48 The IR spectrum of compound **5**

Single Mass Spectrum Deconvolution Report

**Analysis Name:**	shijg038.d	**Instrument:**	LC-MSD-Trap-SL	**Print Date:**	3/21/2011 11:16:54 AM
**Method:**	def_lcms.m	**Operator:**	Operator	**Acq. Date:**	3/21/2011 11:11:13 AM
**Sample Name:**	WYN-74				
**Analysis Info:**					

Acquisition Parameter:

Mass Range Mode	Std/Normal	Trap Drive	45.5	Scan Begin	100 m/z
Ion Polarity	Positive	Octopole RF Amplitude	152.8 Vpp	Scan End	1000 m/z
Ion Source Type	ESI	Capillary Exit	-102.3 Volt	Averages	7 Spectra
Dry Temp (Set)	330 °C	Skimmer	-40.0 Volt	Max. Accu Time	200000 µs
Nebulizer (Set)	15.00 psi	Oct 1 DC	-12.00 Volt	ICC Target	10000
Dry Gas (Set)	5.00 l/min	Oct 2 DC	-1.70 Volt	Charge Control	on

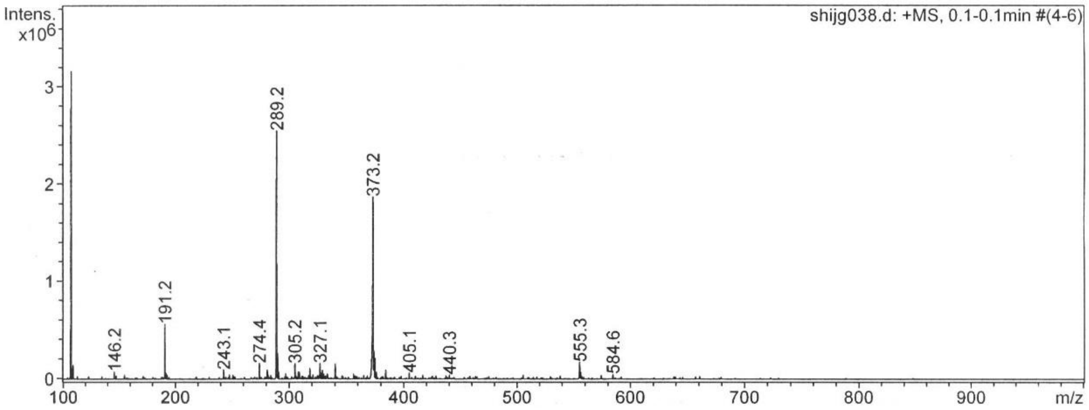

Component Molecular Mass Molecule Absolute Abundance Relative Abundance

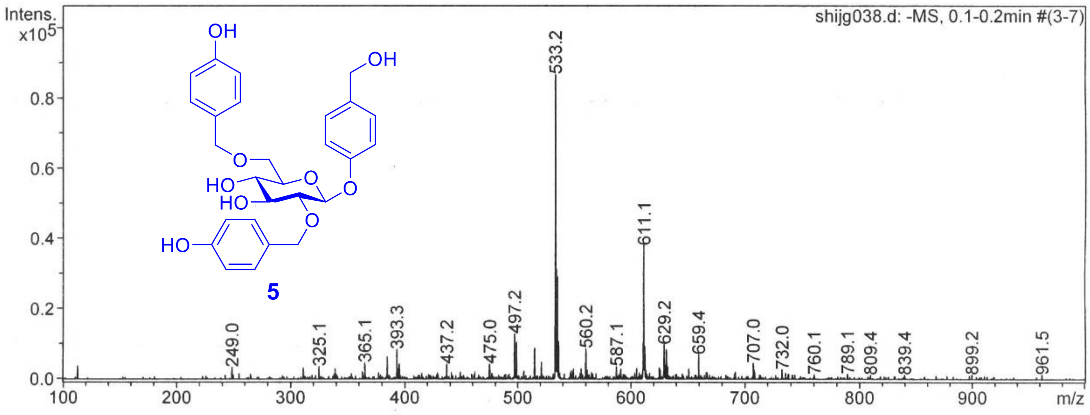

Component Molecular Mass Molecule Absolute Abundance Relative Abundance

Fig. S49 The ESIMS of compound **5**

Qualitative Analysis Report

**Data Filename**	201105054.d	**Sample Name**	WYN-74
**Sample Type**	Sample	**Position**	P1-B4
**Instrument Name**	Instrument 1	**User Name**	
**Acq Method**		**IRM Calibration Status**	Success
**DA Method**	TEST LCMS.m	**Comment**	

User Chromatograms

Fragmentor Voltage 135 **Collision Energy** 0 **Ionization Mode** ESI

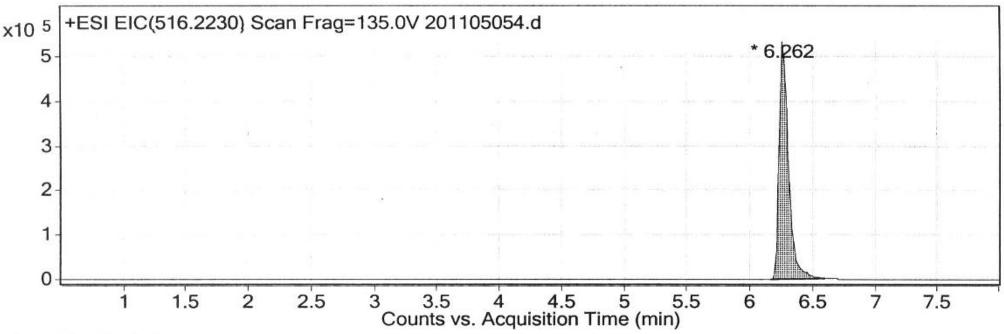

Integration Peak List

Peak	Start	RT	End	Height	Area	Area %
1	6.165	6.262	6.599	533578	2860872	100

User Spectra

Fragmentor Voltage	Collision Energy	Ionization Mode
135	0	Esi

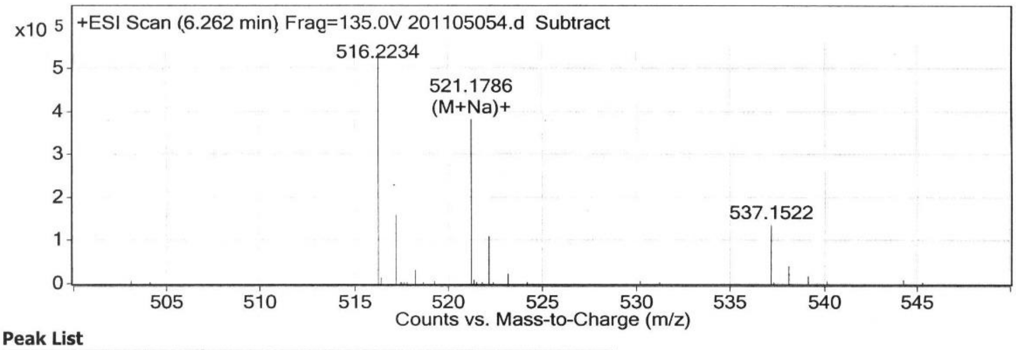

Peak List

m/z	z	Abund	Formula	Ion
516.2234	1	533970		
517.2266	1	158572		
518.2283	1	33006		
521.1786	1	380334	C27 H30 Na O9	(M+Na)+
522.1816	1	110149	C27 H30 Na O9	(M+Na)+
537.1522	1	136084		
538.1555	1	41374		
619.2396		53322		

Formula Calculator Element Limits

Element	Min	Max
C	3	100
H	0	120
O	0	30
N	0	2
S	0	0

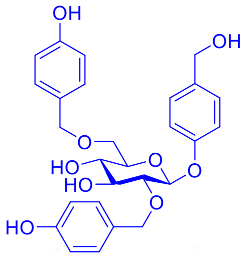

5

Fig. S50 The (+)-HESI-MS report of compound **5**, Page 1

MS Formula Results: + Scan (6.262 min) Sub (201105054.d)

m/z	Ion	Formula	Abundance
521.1786	(M+Na)+	C27 H30 Na O9	380333.8

Best	Formula (M)	Ion Formula	Calc m/z	Score	Cross S	Mass	Calc Mass	Diff (ppm)	Abs Diff (ppm)	Abund Match	Spacing Mat	Mass Match	m/z	DBE
☑	C27 H30 O9	C27 H30 Na O9	521.1782	99.96		498.1894	498.189	-0.82	0.82	99.93	99.98	99.98	521.1786	13
☐	C22 H30 N2 O11	C22 H30 N2 Na O11	521.1742	98.51		498.1894	498.185	-8.9	8.9	98.9	99.99	97.53	521.1786	9
☐	C33 H26 N2 O3	C33 H26 N2 Na O3	521.1836	97.34		498.1894	498.1943	9.94	9.94	95.83	99.98	96.94	521.1786	22
☐	C15 H34 N2 O16	C15 H34 N2 Na O16	521.1801	96.77		498.1894	498.1908	2.89	2.89	89.16	99.99	99.74	521.1786	0

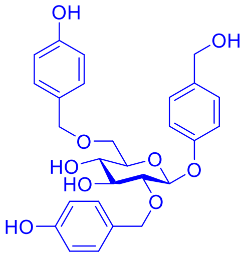

5

page 1

Fig. S51 The (+)-HRESIMS report of compound **5**, Page 2

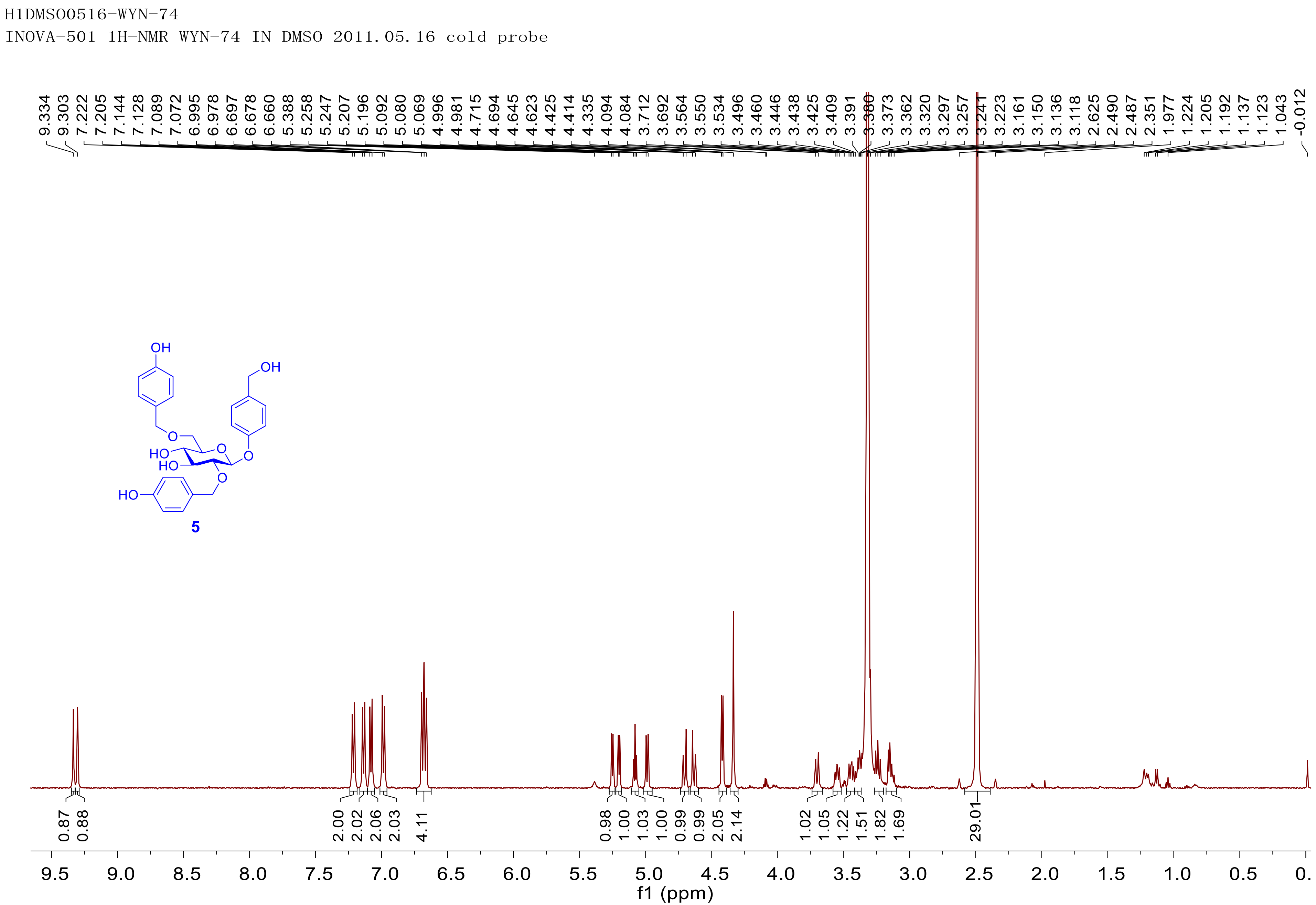

Fig. S52 The ^{1}H NMR spectrum of compound **5** in DMSO-d_6 at 500 MHz

C13DMSO218-WYN-74
INOVA-501 13C-NMR WYN-74 IN DMSO 2011.02.18 cold probe

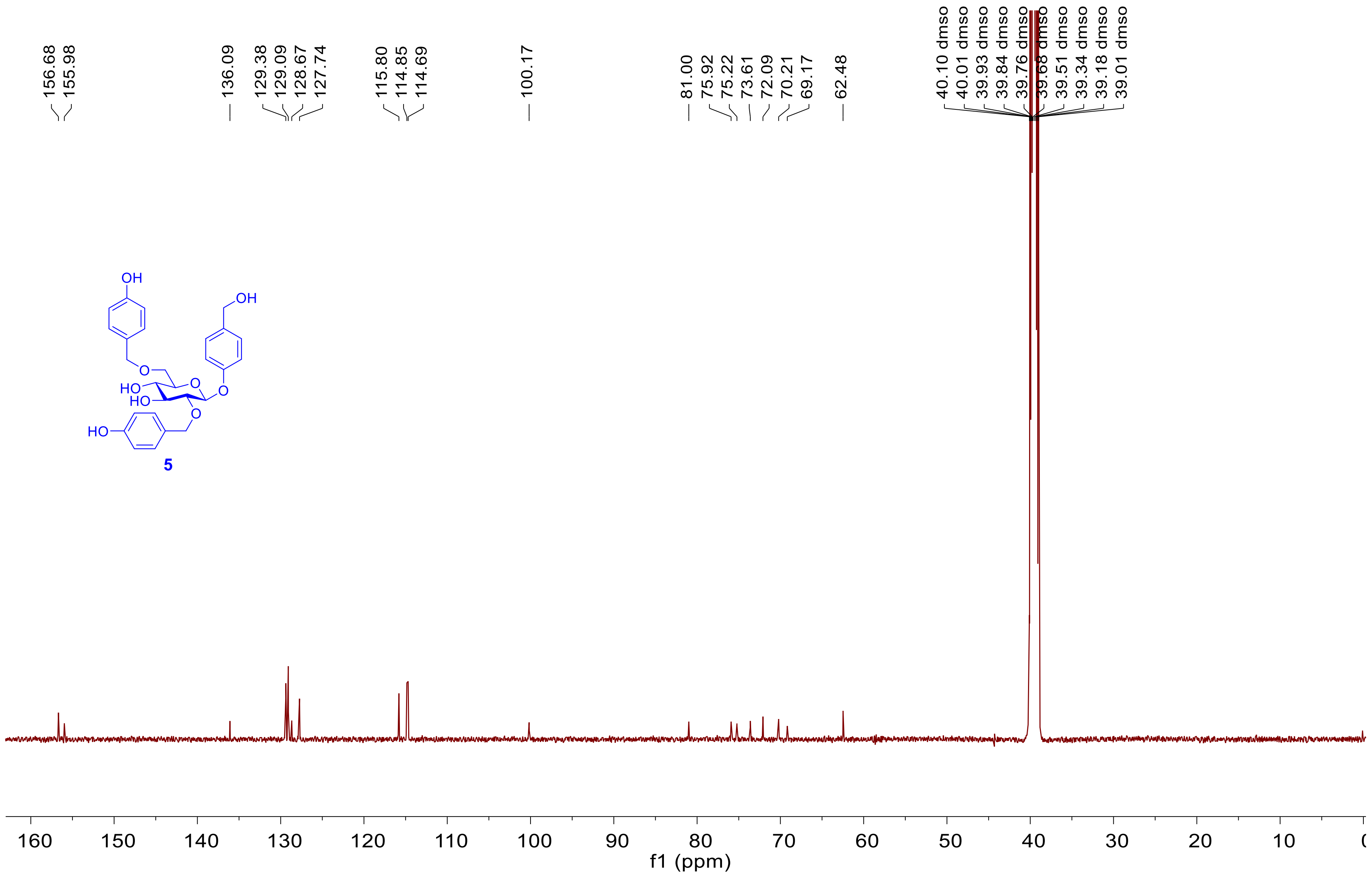

Fig. S53 The ^{13}C NMR spectrum of compound **5** in DMSO-d_6 at 125 MHz

C13DMSO218-WYN-74
INOVA-501 DEPT WYN-74 IN DMSO 2011.02.18 cold probe

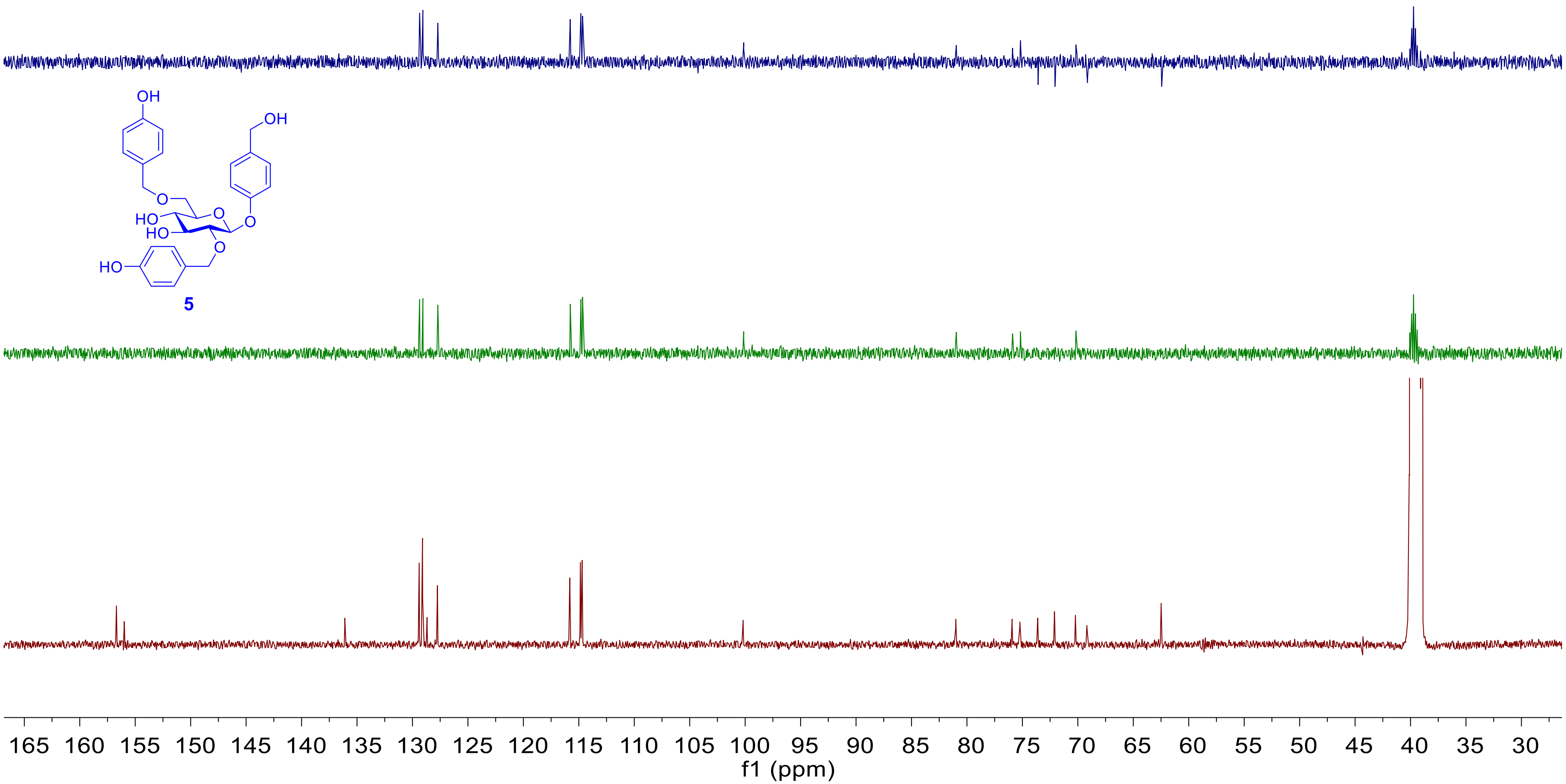

Fig. S54 The DEPT spectrum of compound **5** in DMSO-d_6 at 125 MHz

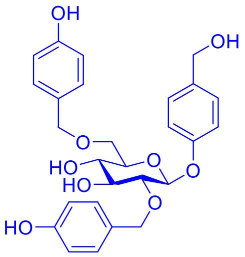

5

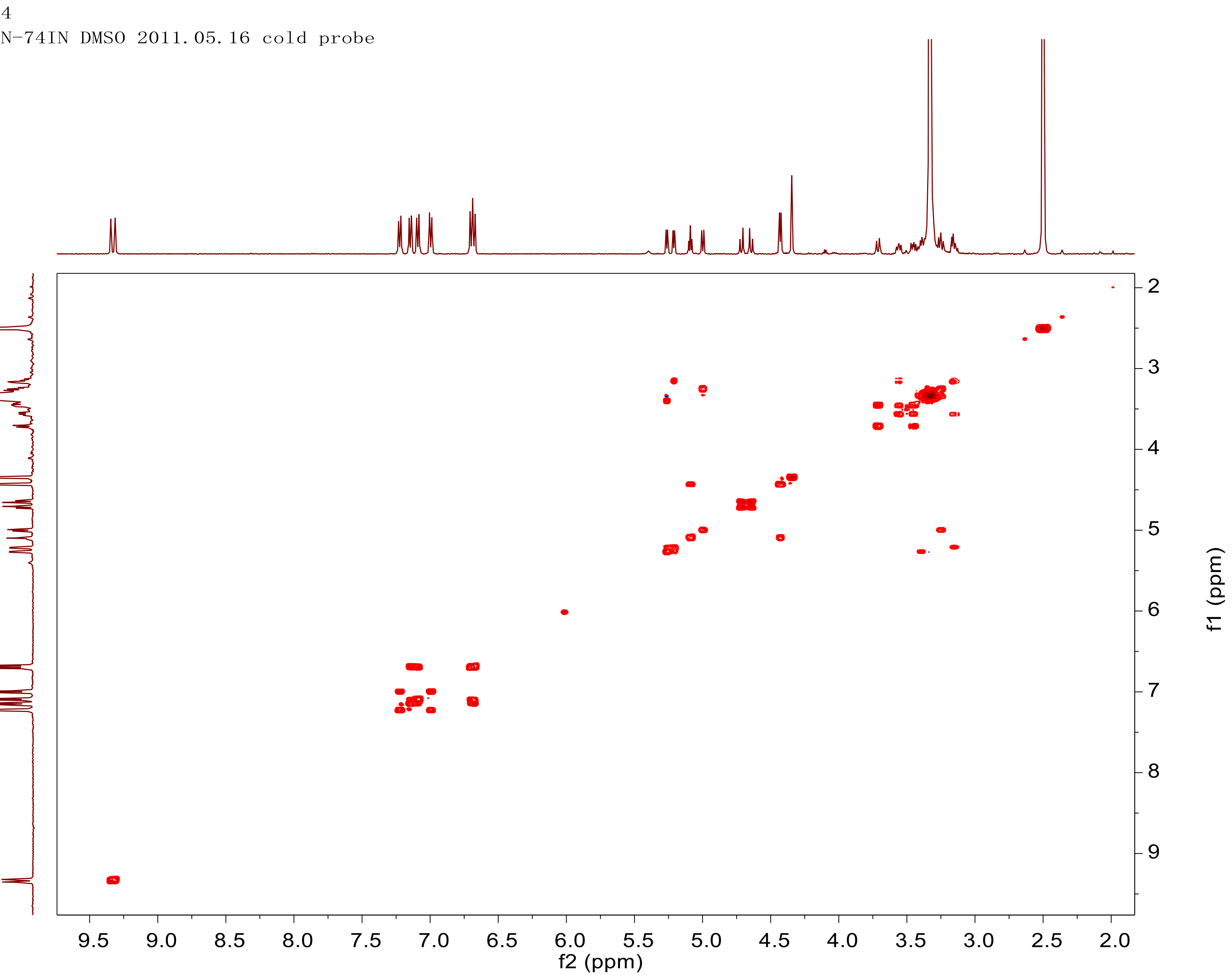

Fig. S55 The ^{1}H-^{1}H COSY spectrum of compound **5** in DMSO-d_6 at 500 MHz

HSQCDMSO0627-WYN-74
INOVA-501 gHSQC WYN-74 IN DMSO 2011.06.27 cold probe

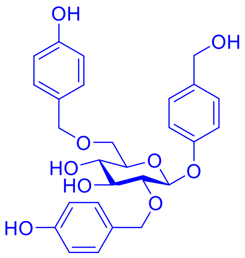

5

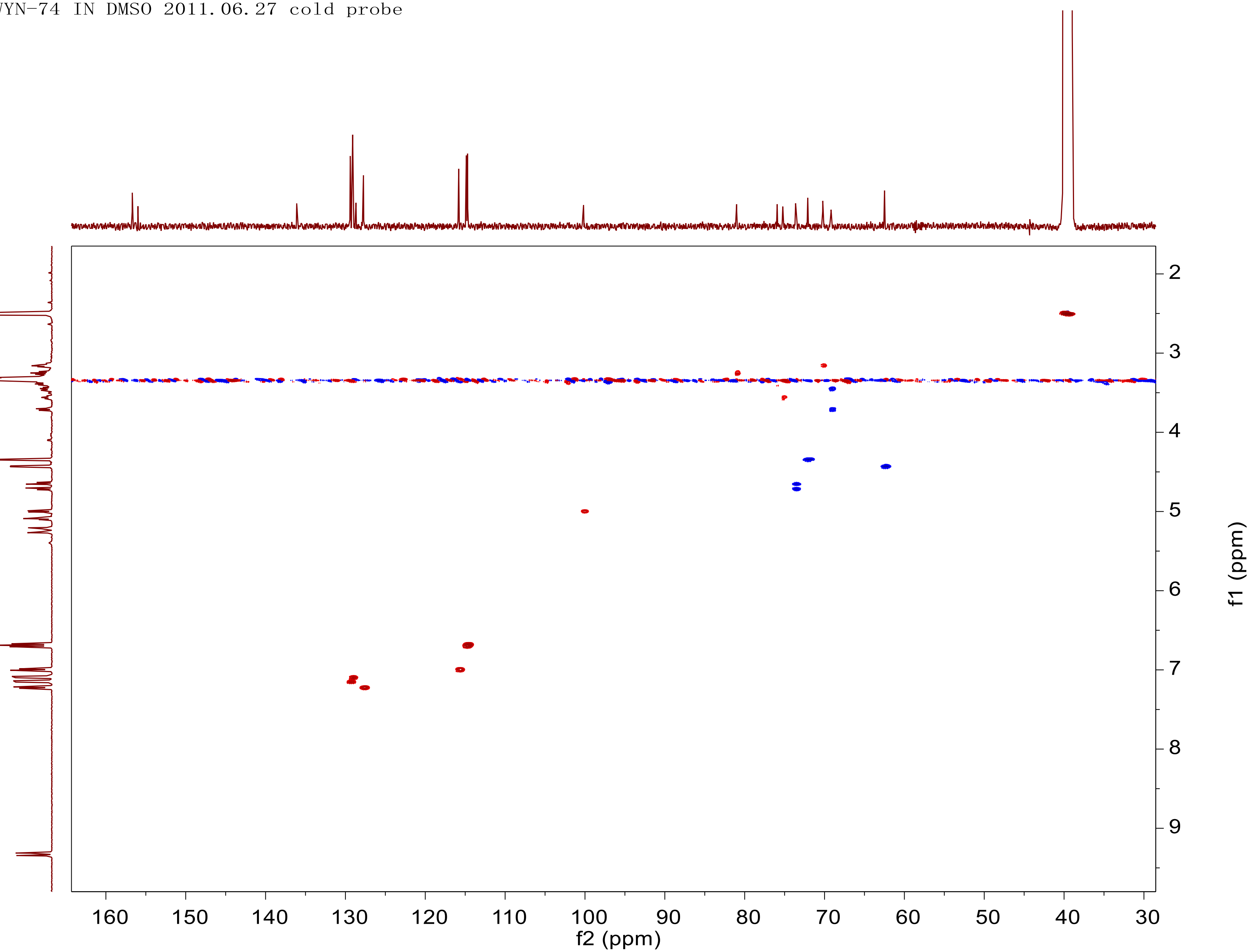

Fig. S56 The HSQC spectrum of compound **5** in DMSO-d_6 (500 MHz for ^{1}H)

HMBCDMSO0627-WYN-74
INOVA-501 gHMBC WYN-74 IN DMSO 2011.06.27 cold probe

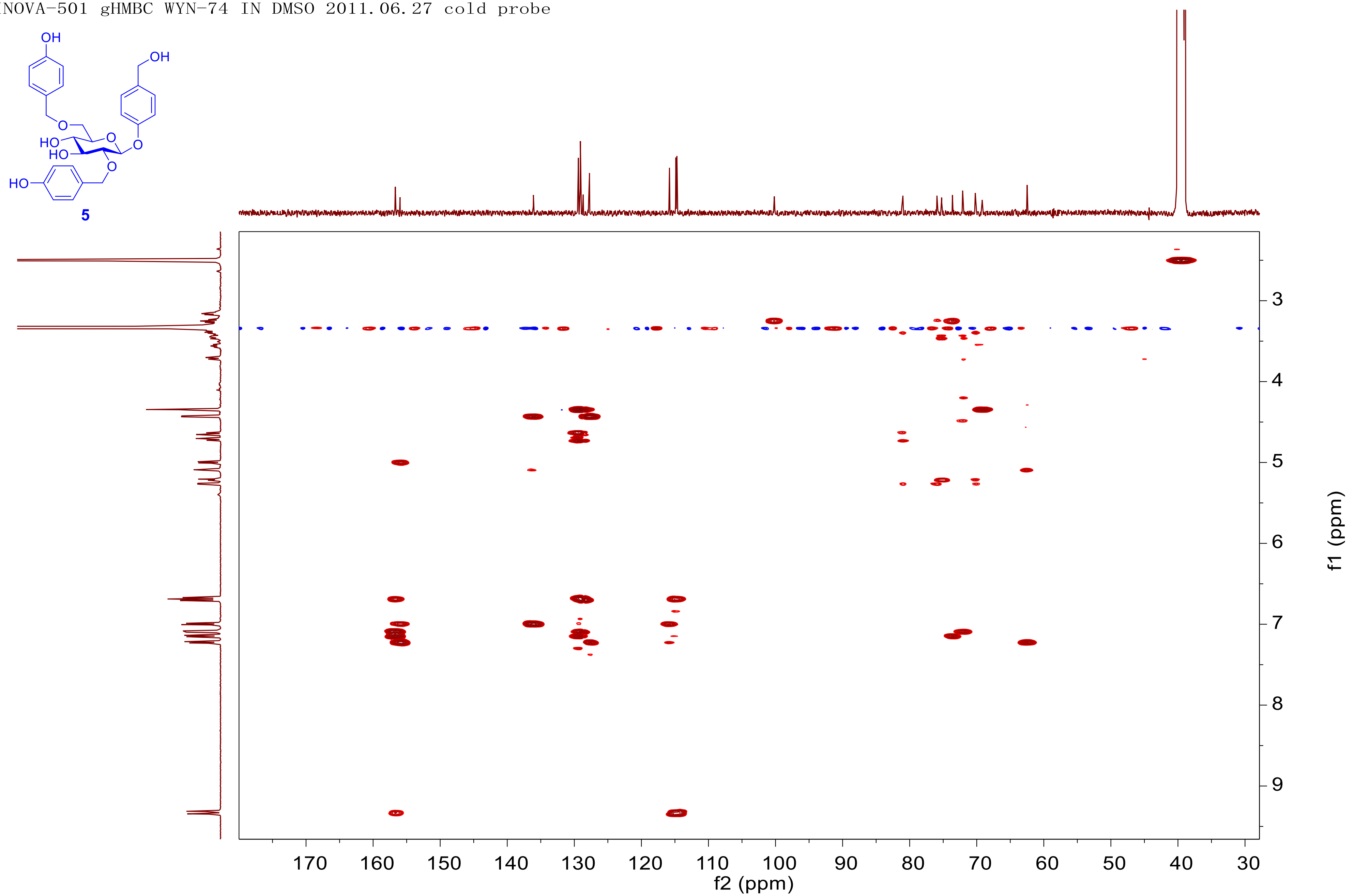

Fig. S57 The HMBC spectrum of compound **5** in DMSO-d_6 (500 MHz for ^{1}H)

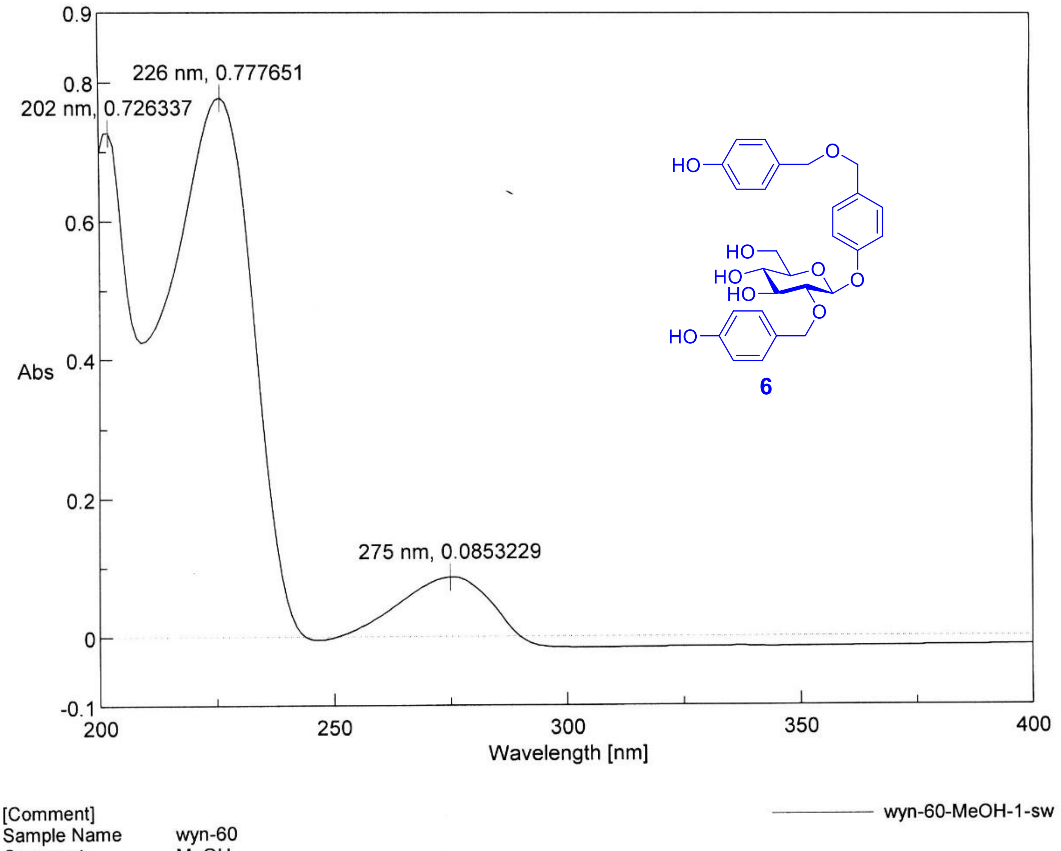

[Comment]
Sample Name wyn-60
Comment MeOH
User 王亚男
Division UV
Company 324

[Measurement Information]
Instrument Name V-650
Model Name V-650
Serial No. A034461150

Accessory PSC-718
Accessory S/N A001761114
Position 1
Cell Length 10 mm
Temperature 19.96 C
Control Sonsor Holder
Monitor Sensor Holder
Start Mode Start immediately

Photometric Mode Abs
Measurement range 400 - 200 nm
Data pitch 1 nm
Band width(UV/Vis) 1.0 nm
Response Medium
Scanning speed 200 nm/min
Source Change 340 nm
Light Source D2/WI
Filter Exchange Step
Correction Baseline

[Data Information]
Creation Date 2012-5-3 11:27

Data array type Linear data array
Horizontal Wavelength [nm]
Vertical Abs
Start 400 nm
End 200 nm
Data pitch 1 nm
Data points 201

Fig. S58 The UV spectrum of compound **6**

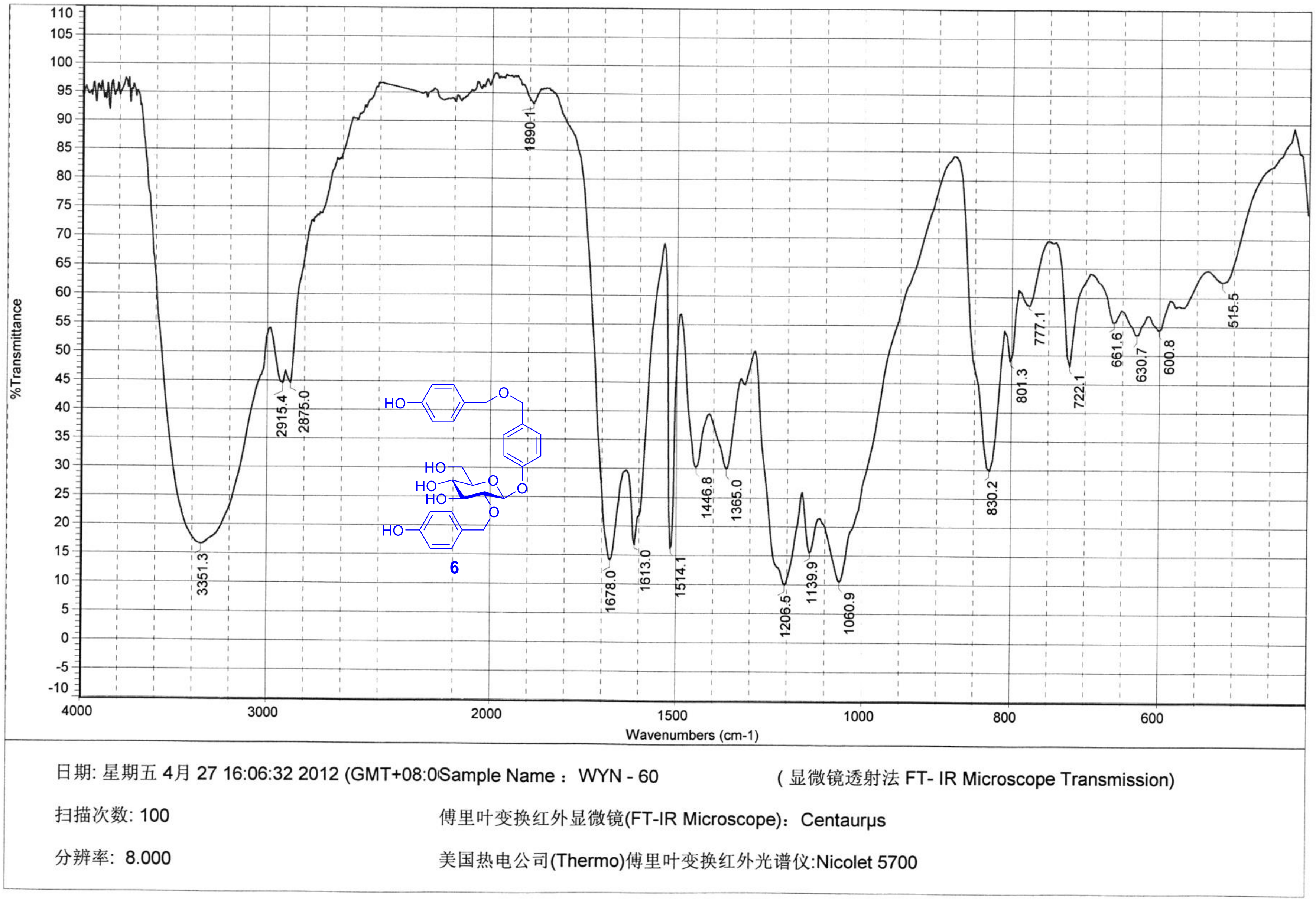

Fig. S59 The IR spectrum of compound **6**

Single Mass Spectrum Deconvolution Report

Analysis Name: wangy040.d
Method: def_lcms.m
Sample Name: WYN-60
Analysis Info:

Instrument: LC-MSD-Trap-SL
Operator: Operator

Print Date: 7/11/2011 10:00:54 AM
Acq. Date: 7/11/2011 9:59:09 AM

Acquisition Parameter:

Mass Range Mode	Std/Normal	Trap Drive	53.0	Scan Begin	100 m/z
Ion Polarity	Positive	Octopole RF Amplitude	171.0 Vpp	Scan End	1000 m/z
Ion Source Type	ESI	Capillary Exit	-106.0 Volt	Averages	7 Spectra
Dry Temp (Set)	330 °C	Skimmer	-40.0 Volt	Max. Accu Time	200000 µs
Nebulizer (Set)	15.00 psi	Oct 1 DC	-12.00 Volt	ICC Target	10000
Dry Gas (Set)	5.00 l/min	Oct 2 DC	-1.70 Volt	Charge Control	on

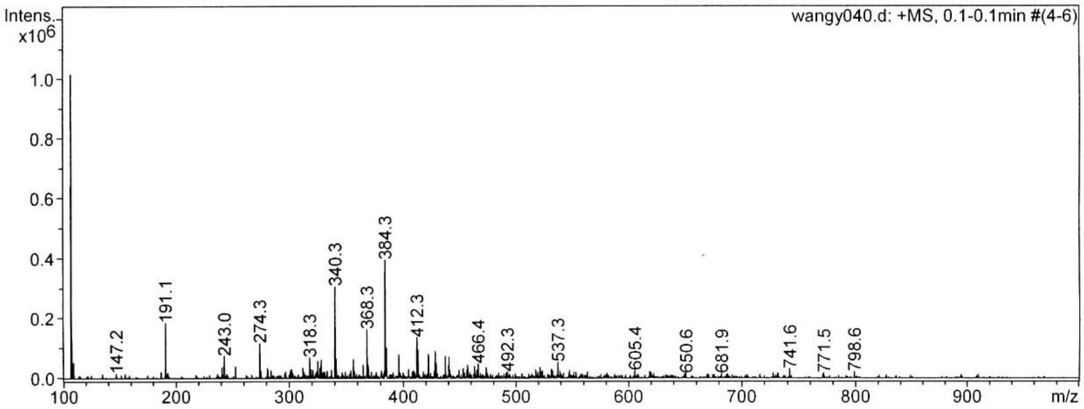

Component	Molecular Mass	Molecule	Absolute Abundance	Relative Abundance

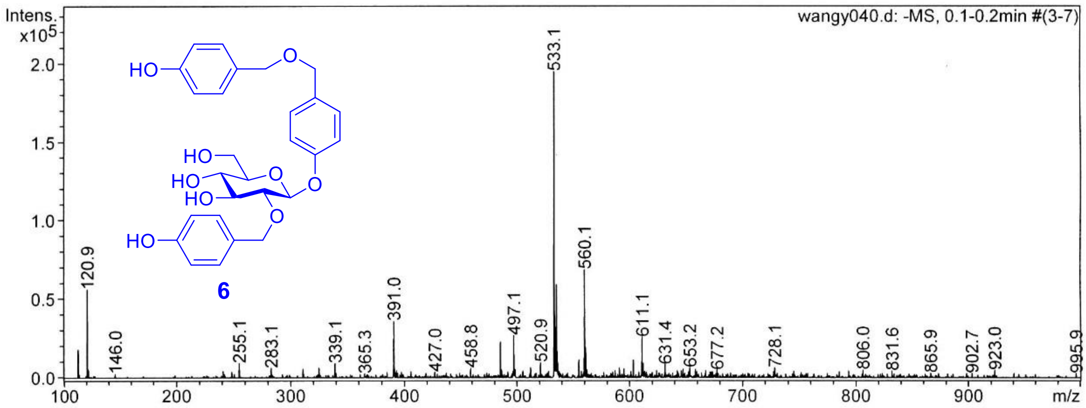

Component	Molecular Mass	Molecule	Absolute Abundance	Relative Abundance

Fig. S60 The ESIMS of compound **6**

Qualitative Analysis Report

**Data Filename**	2011111801.d	**Sample Name**	WYN-60
**Sample Type**	Sample	**Position**	P1-D1
**Instrument Name**	Instrument 1	**User Name**	
**Acq Method**		**IRM Calibration Status**	Success
**DA Method**	TEST LCMS.m	**Comment**	

User Chromatograms

Fragmentor Voltage 135 **Collision Energy** 0 **Ionization Mode** ESI

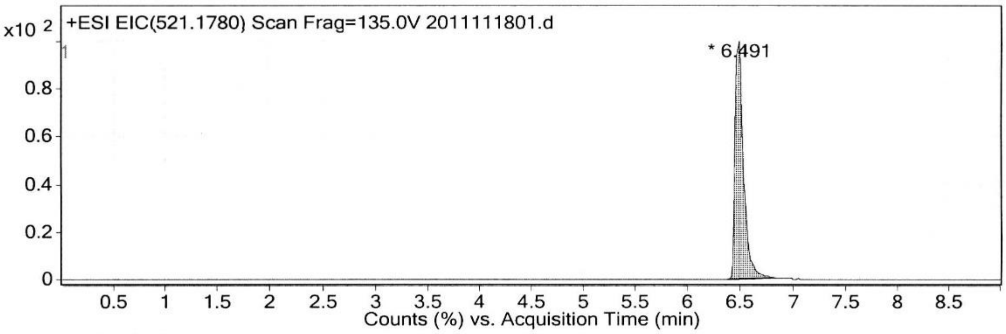

Integration Peak List

Peak	Start	RT	End	Height	Area	Area %
1	6.378	6.491	6.828	264397	1498780	100

User Spectra

Fragmentor Voltage 135 **Collision Energy** 0 **Ionization Mode** Esi

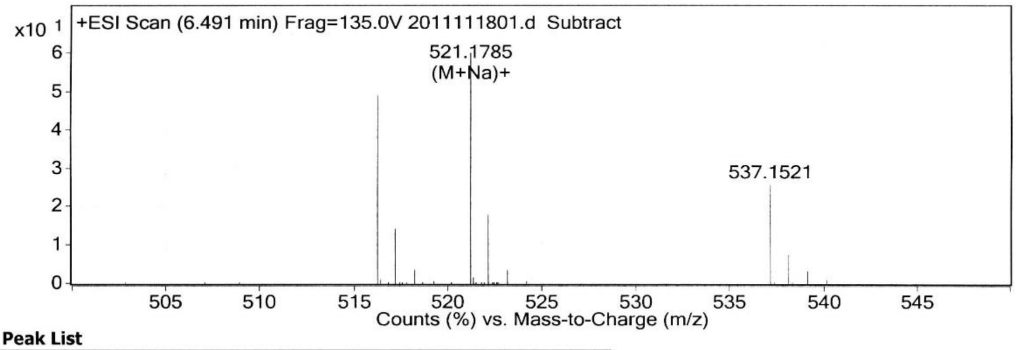

Peak List

m/z	z	Abund	Formula	Ion
107.0413		219933		
169.1102		50085		
191.0921		42825		
516.2231	1	215848		
517.2262	1	62527		
521.1785	1	264827	C27 H30 Na O9	(M+Na)+
522.182	1	78427	C27 H30 Na O9	(M+Na)+
537.1521	1	112819		
538.1556	1	33468		
657.1523		41790		

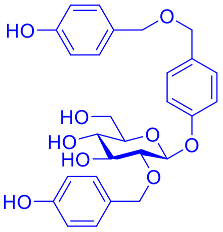

6

Formula Calculator Element Limits

Element	Min	Max
C	3	100
H	0	120
O	0	30

Fig. S61 The (+)-HRESIMS report of compound **6**, Page 1

MS Formula Results: + Scan (6.491 min) Sub (2011111801.d)

m/z	Ion	Formula	Abundance
521.1785	(M+Na)+	C27 H30 Na O9	264827.4

Best	Formula (M)	Ion Formula	Calc m/z	Score	Cross S	Mass	Calc Mass	Diff (ppm)	Abs Diff (ppm)	Abund Match	Spacing Mat	Mass Match	m/z	DBE
☑	C27 H30 O9	C27 H30 Na O9	521.1782	99.99		498.1893	498.189	-0.69	0.69	99.98	100	99.98	521.1785	13
☐	C28 H26 N4 O5	C28 H26 N4 Na O5	521.1795	99.78		498.1893	498.1903	1.98	1.98	99.43	99.99	99.88	521.1785	18
☐	C24 H34 O9 S	C24 H34 Na O9 S	521.1816	98.51		498.1893	498.1924	6.07	6.07	96.97	99.67	98.85	521.1785	8
☐	C32 H26 N4 S	C32 H26 N4 Na S	521.177	98.49		498.1893	498.1878	-3.05	3.05	95.42	99.75	99.71	521.1785	22
☐	C31 H30 O4 S	C31 H30 Na O4 S	521.1757	98.48		498.1893	498.1865	-5.72	5.72	96.58	99.78	98.97	521.1785	17
☐	C22 H30 N2 O11	C22 H30 N2 Na O11	521.1742	98.43		498.1893	498.185	-8.78	8.78	98.5	99.99	97.6	521.1785	9
☐	C25 H30 N4 O5 S	C25 H30 N4 Na O5 S	521.1829	98.25		498.1893	498.1937	8.73	8.73	98.14	99.63	97.62	521.1785	13
☐	C19 H34 N2 O11 S	C19 H34 N2 Na O11 S	521.1776	97.9		498.1893	498.1883	-2.02	2.02	93.24	99.56	99.87	521.1785	4
☐	C28 H34 O4 S2	C28 H34 Na O4 S2	521.1791	97.61		498.1893	498.1899	1.04	1.04	92.15	99.44	99.97	521.1785	12
☐	C29 H30 N4 S2	C29 H30 N4 Na S2	521.1804	97.48		498.1893	498.1912	3.71	3.71	92.42	99.39	99.57	521.1785	17
☐	C35 H22 N4	C35 H22 N4 Na	521.1737	96.77		498.1893	498.1844	-9.81	9.81	93.7	99.99	97.01	521.1785	27
☐	C23 H34 N2 O6 S2	C23 H34 N2 Na O6 S2	521.175	96.64		498.1893	498.1858	-7.05	7.05	91.42	99.3	98.45	521.1785	8
☐	C15 H34 N2 O16	C15 H34 N2 Na O16	521.1801	96.44		498.1893	498.1908	3.01	3.01	88.02	99.98	99.71	521.1785	0

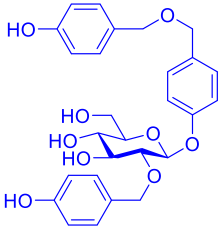

6

page 1

Fig. S62 The (+)-HRESIMS report of compound **6**, Page 2

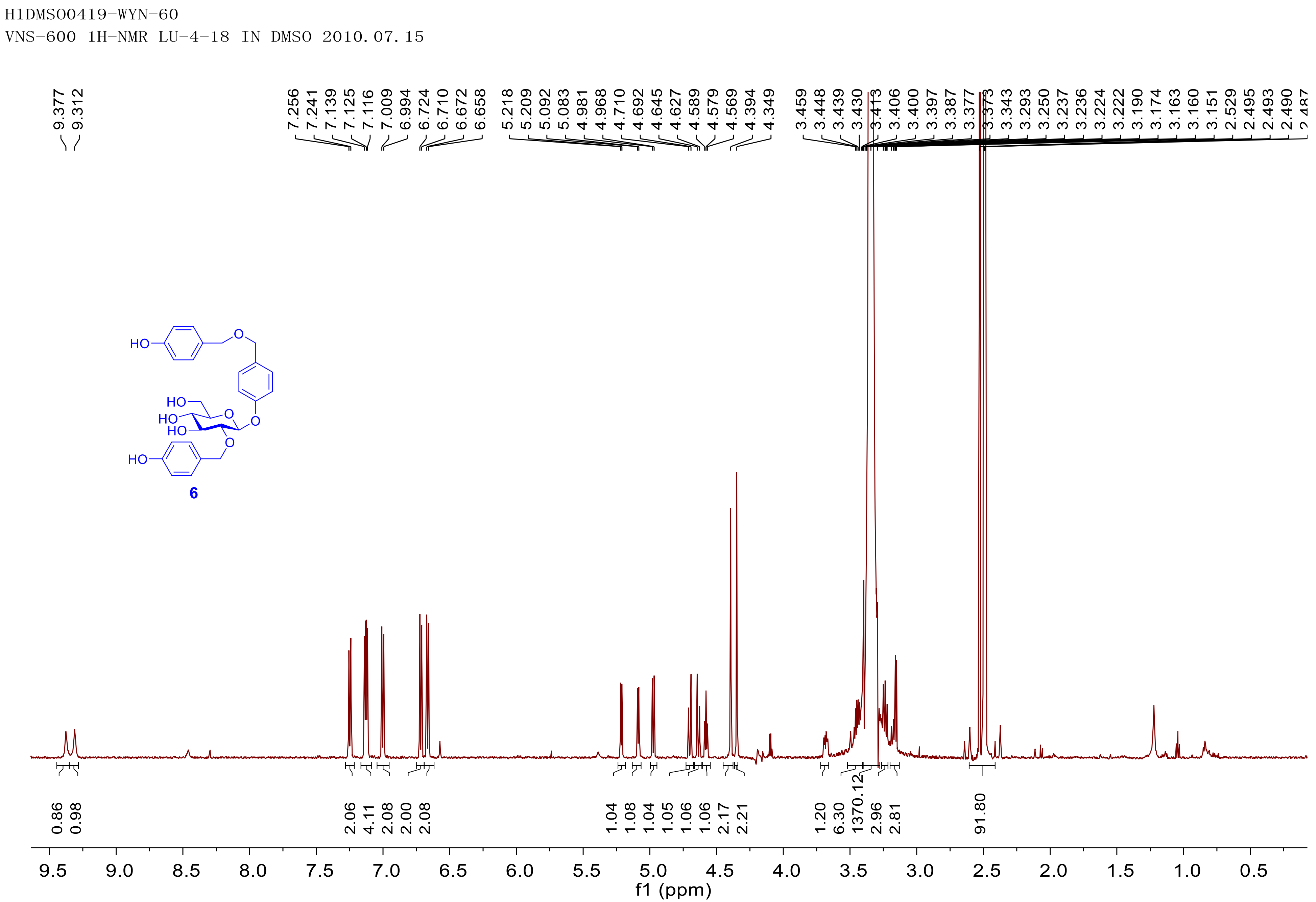

Fig. S63 The ^{1}H NMR spectrum of compound **6** in DMSO-d_6 at 500 MHz

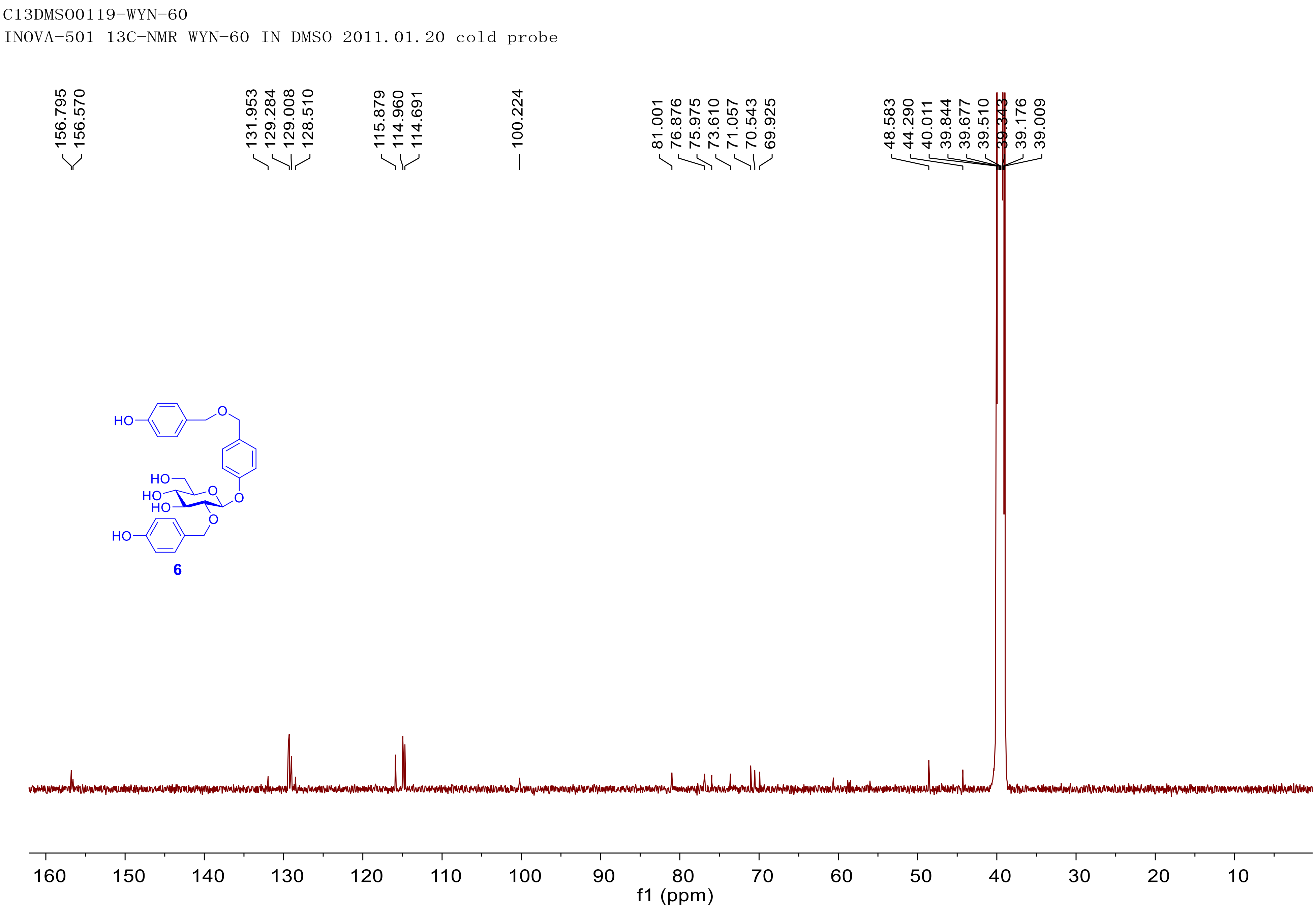

Fig. S64 The ^{13}C NMR spectrum of compound **6** in DMSO-d_6 at 125 MHz

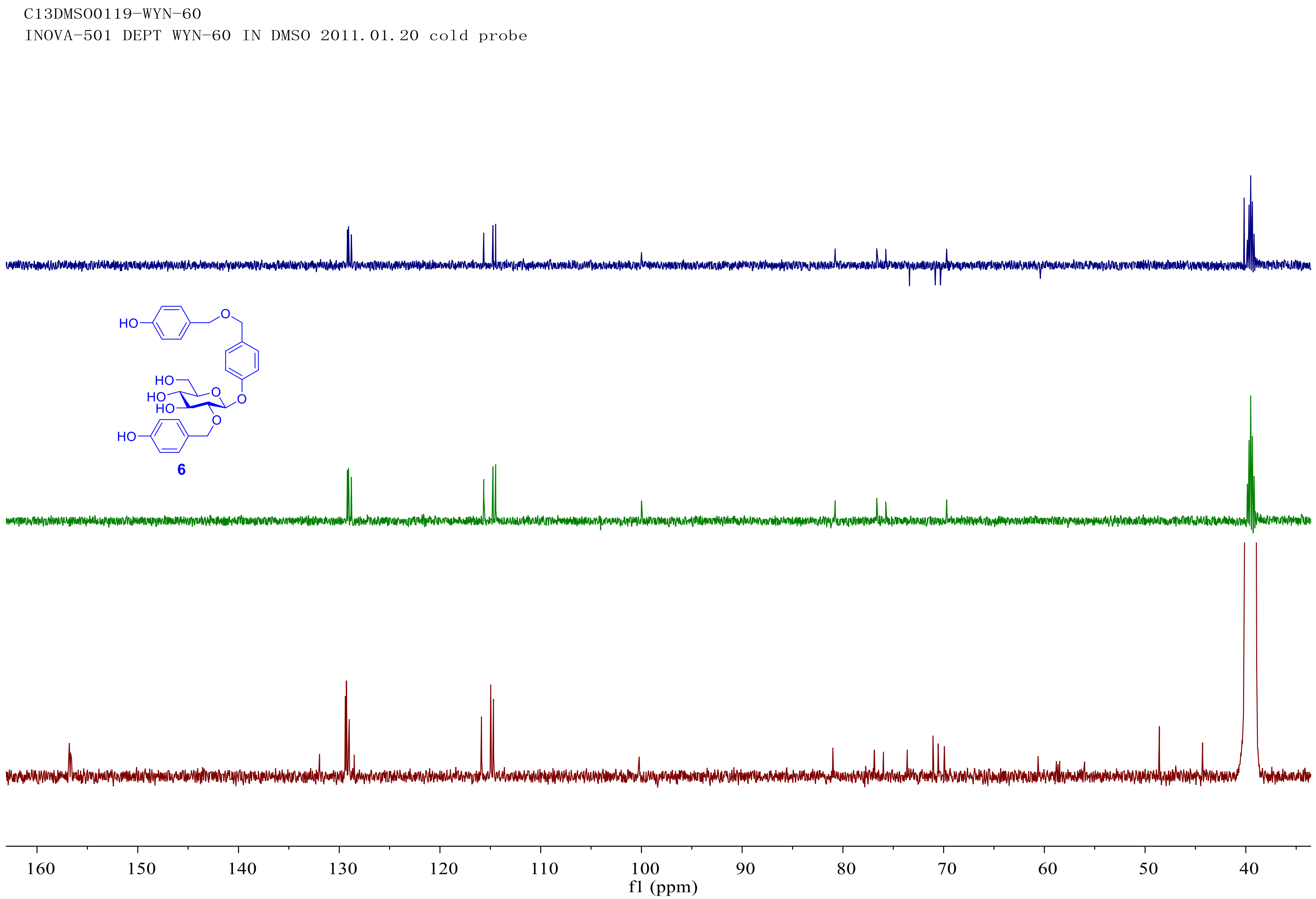

Fig. S65 The DEPT spectrum of compound **6** in DMSO-d_6 at 125 MHz

INOVA-501 gCOSY WYN-60 IN DMSO 2011.06.16 cold probe

Solvent: dmso
Temp. 25.0 C / 298.1 K
Sample #7, Operator: walkup
File: COSYDMSO0616-WYN-60
INOVA-500 "IMM-501"

Relax. delay 1.301 sec
Acq. time 0.221 sec
Width 4638.2 Hz
2D Width 4638.2 Hz
2 repetitions
256 increments
OBSERVE H1, 499.7704835 MHz
DATA PROCESSING
Sine bell 0.110 sec
F1 DATA PROCESSING
Sine bell 0.028 sec
FT size 4096 x 4096
Total time 13 min, 44 sec

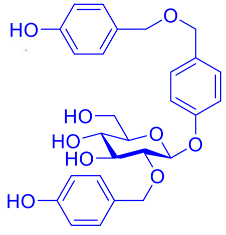

6

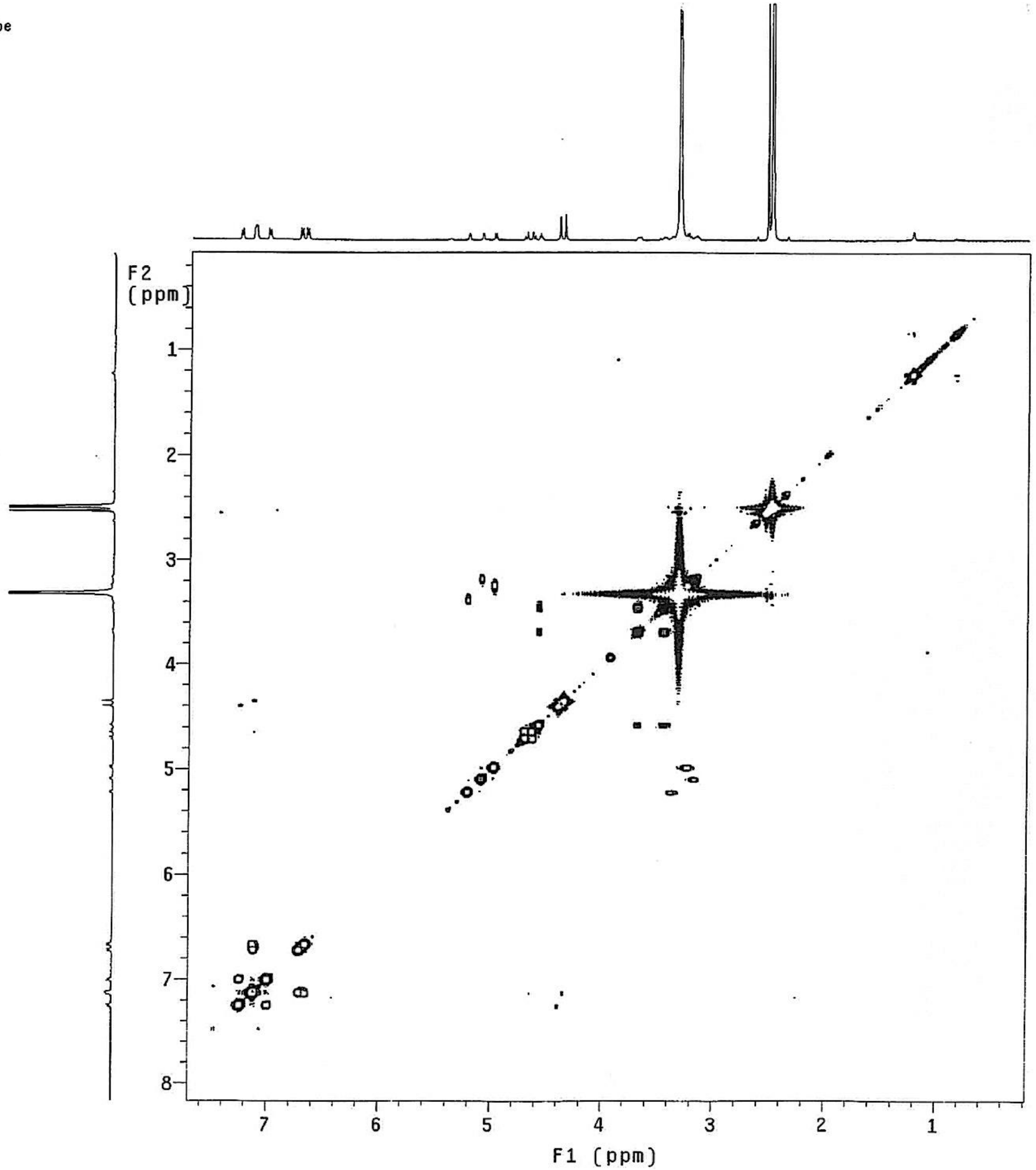

Fig. S66 The ^{1}H-^{1}H COSY spectrum of compound **6** in DMSO-d_6 at 500 MHz

HSQCDMSO0419-WYN-60
VNS-600 gHSQC WYN-60 in DMSO-d6 2012.04.20

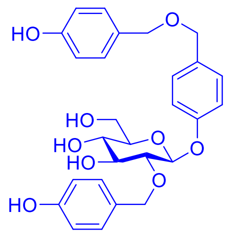

6

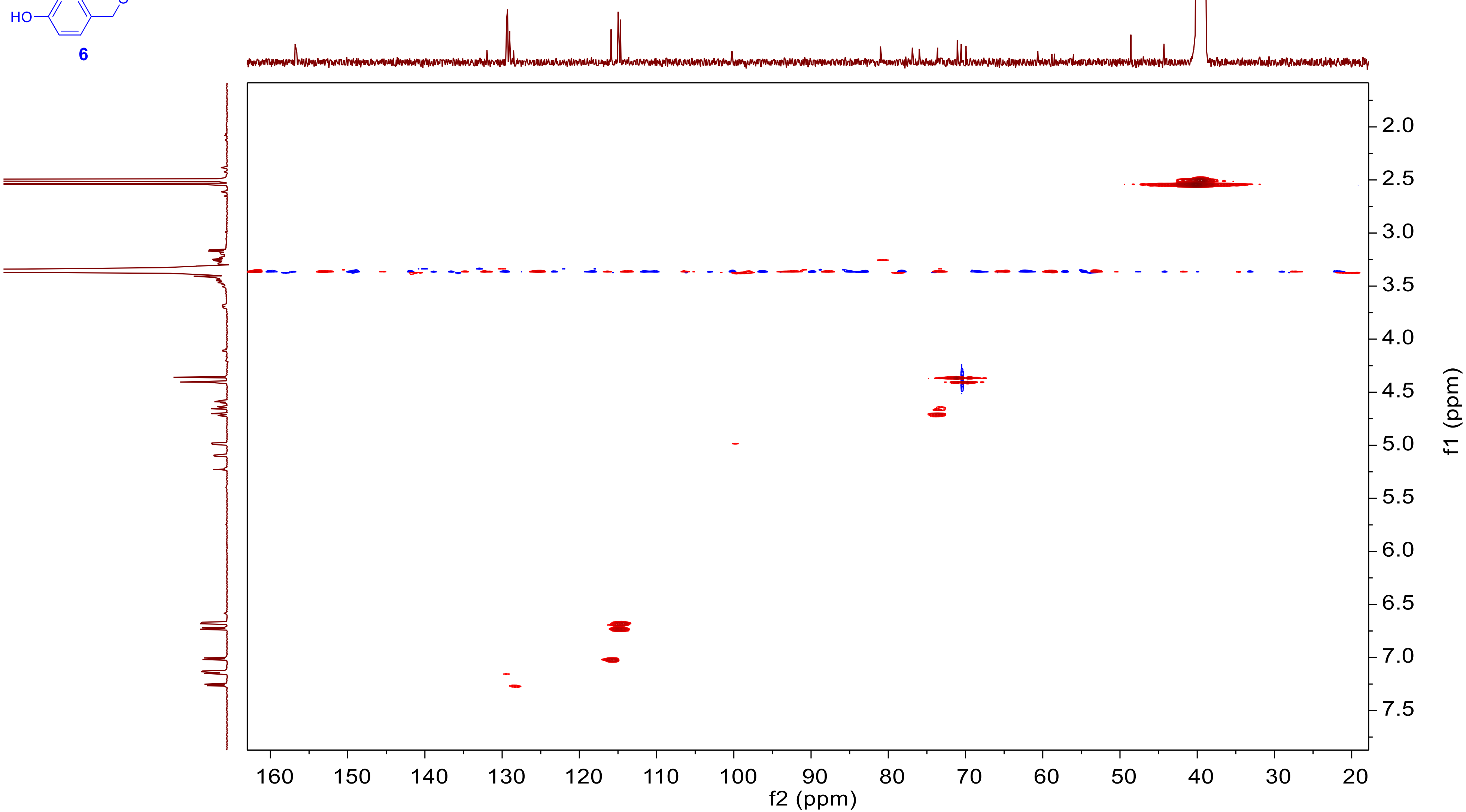

Fig. S67 The HSQC spectrum of compound **6** in DMSO-d_6 (500 MHz for ^{1}H)

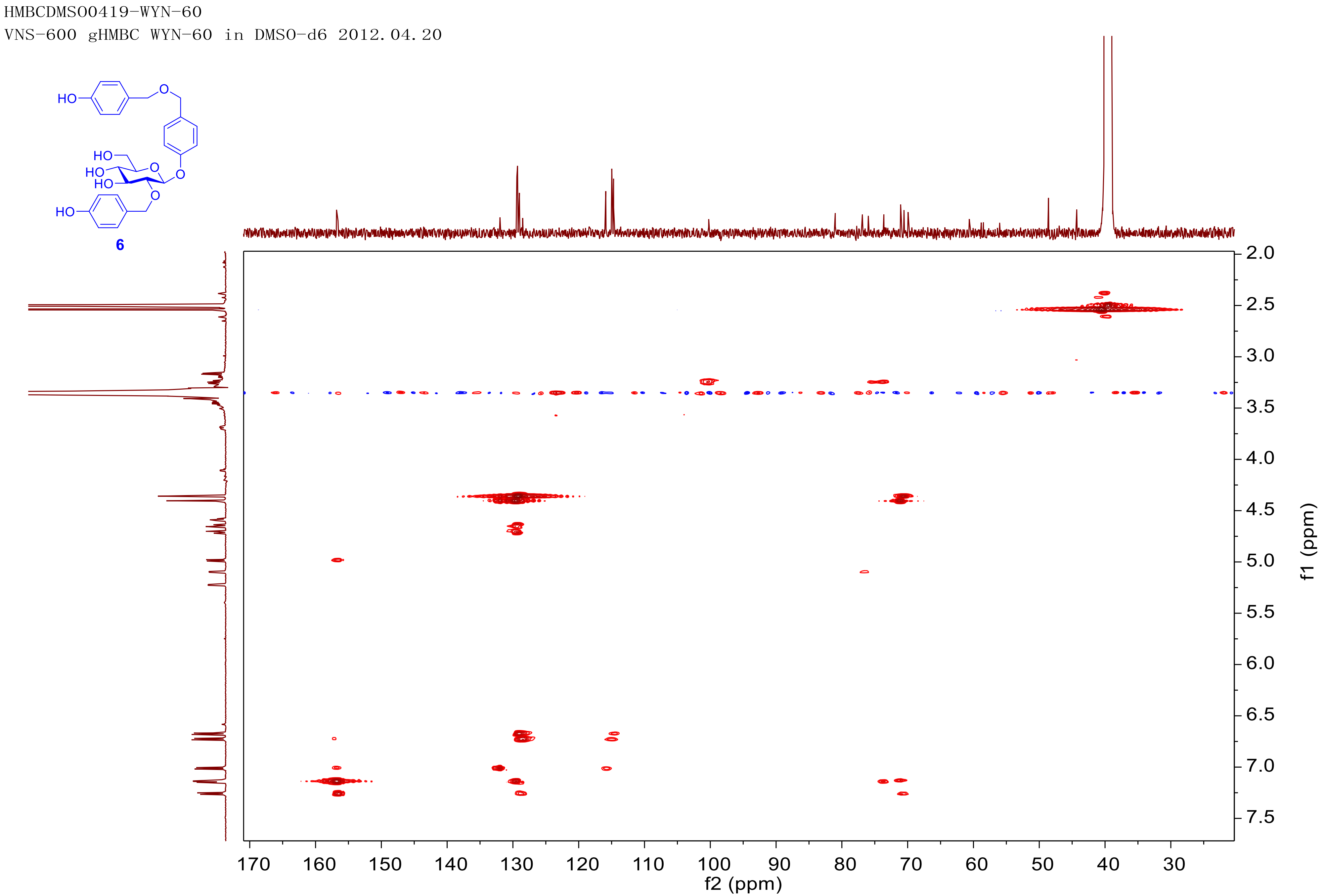

Fig. S68 The HMBC spectrum of compound **6** in DMSO-d_6 (500 MHz for ^{1}H)

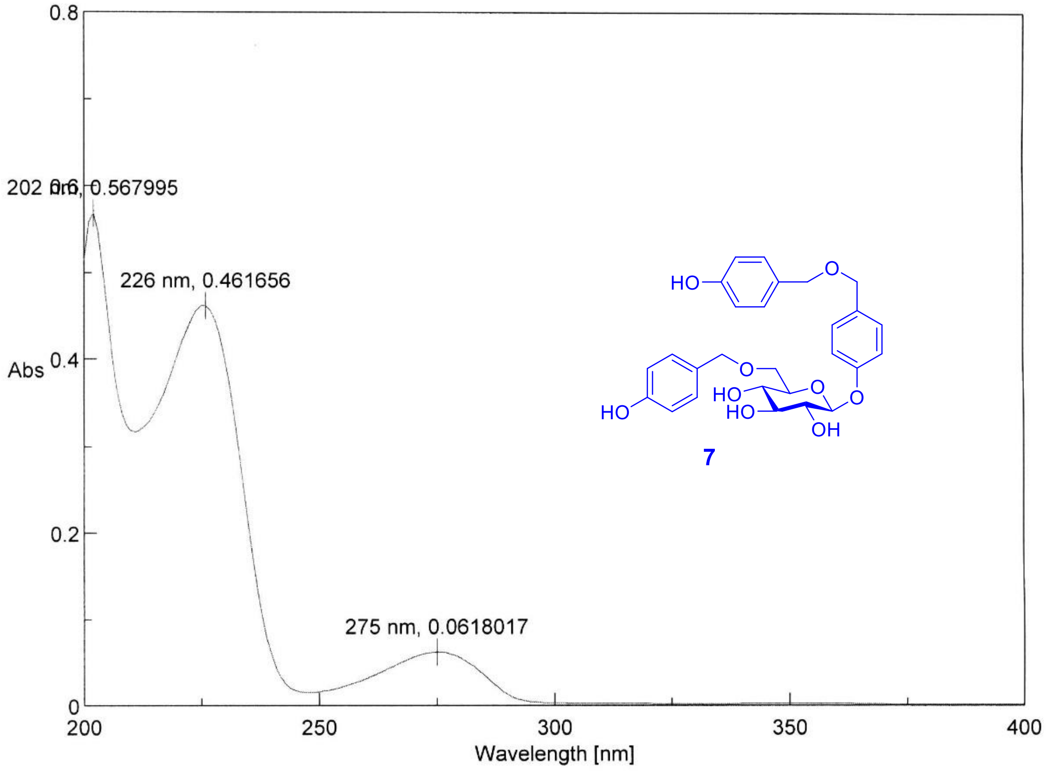

WYN-59-4-S

[Comment]
Sample Name WYN-59
Comment
User
Division
Company 324

[Measurement Information]
Instrument Name V-650
Model Name V-650
Serial No. A034461150

Accessory PSC-718
Accessory S/N A001761114
Position 1
Cell Length 10 mm
Temperature 19.96 C
Control Sensor Holder
Monitor Sensor Holder
Start Mode Start immediately

Photometric Mode Abs
Measurement range 400 - 190 nm
Data pitch 1 nm
Band width(UV/Vis) 1.0 nm
Response Medium
Scanning speed 200 nm/min
Source Change 340 nm
Light Source D2/WI
Filter Exchange Step
Correction Baseline

[Data Information]
Creation Date 2011-6-1 17:09

Data array type Linear data array
Horizontal Wavelength [nm]
Vertical Abs
Start 400 nm
End 190 nm
Data pitch 1 nm
Data points 211

Fig. S69 The UV spectrum of compound **7**

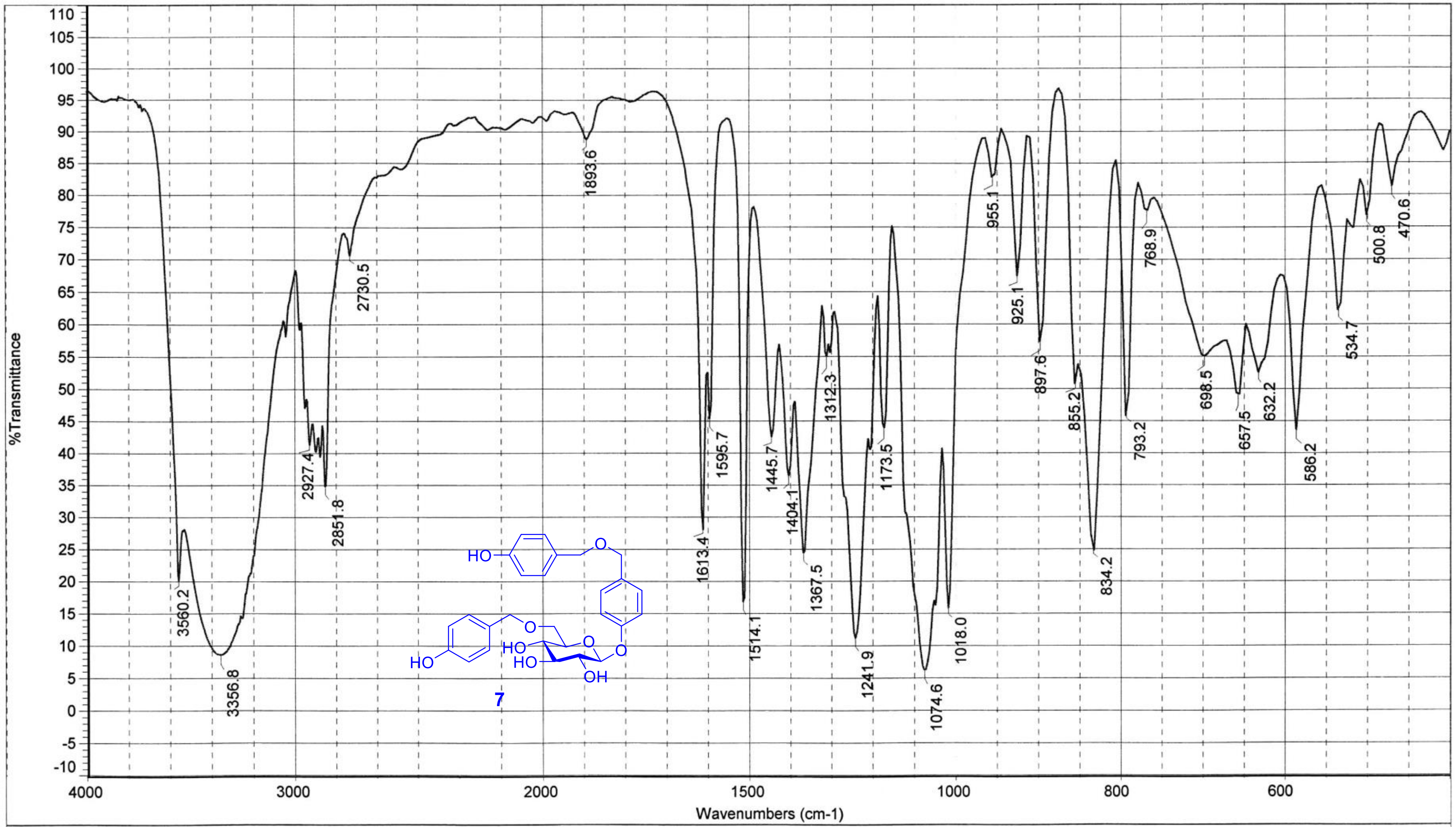

日期: 星期四 11月 24 11:01:35 2011 (GMT+08:Sample Name : WYN - 59 (显微镜透射法 FT- IR Microscope Transmission)

扫描次数: 100 傅里叶变换红外显微镜(FT-IR Microscope): Centaurμs

分辨率: 8.000 美国热电公司(Thermo)傅里叶变换红外光谱仪:Nicolet 5700

Fig. S70 The IR spectrum of compound **7**

Single Mass Spectrum Deconvolution Report

**Analysis Name:**	WANGY018.d	**Instrument:**	LC-MSD-Trap-SL	**Print Date:**	7/9/2010 12:18:25 PM
**Method:**	TEST.MS	**Operator:**	Operator	**Acq. Date:**	7/9/2010 12:16:50 PM
**Sample Name:**	WYN-59				
**Analysis Info:**					

Acquisition Parameter:

Mass Range Mode	Std/Normal	Trap Drive	36.3	Scan Begin	100 m/z
Ion Polarity	Positive	Octopole RF Amplitude	171.0 Vpp	Scan End	700 m/z
Ion Source Type	ESI	Capillary Exit	106.0 Volt	Averages	5 Spectra
Dry Temp (Set)	330 °C	Skimmer	40.0 Volt	Max. Accu Time	200000 µs
Nebulizer (Set)	15.00 psi	Oct 1 DC	12.00 Volt	ICC Target	20000
Dry Gas (Set)	5.00 l/min	Oct 2 DC	1.70 Volt	Charge Control	on

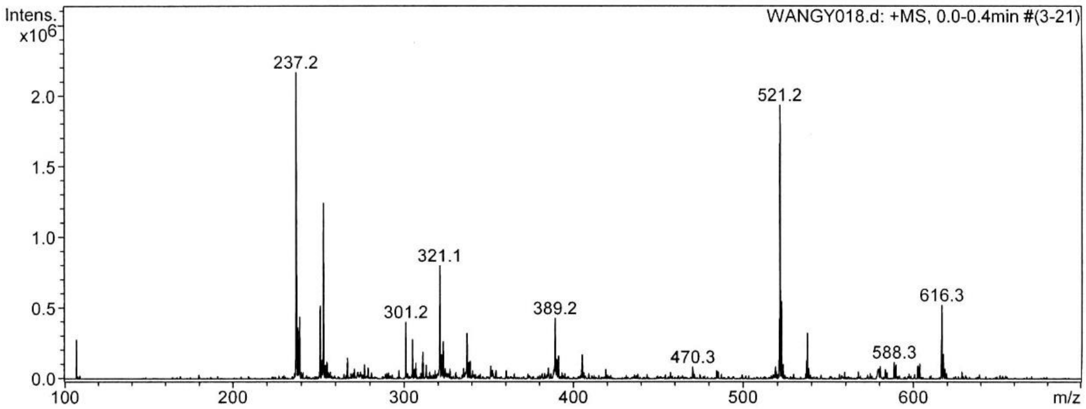

Component	Molecular Mass	Molecule	Absolute Abundance	Relative Abundance

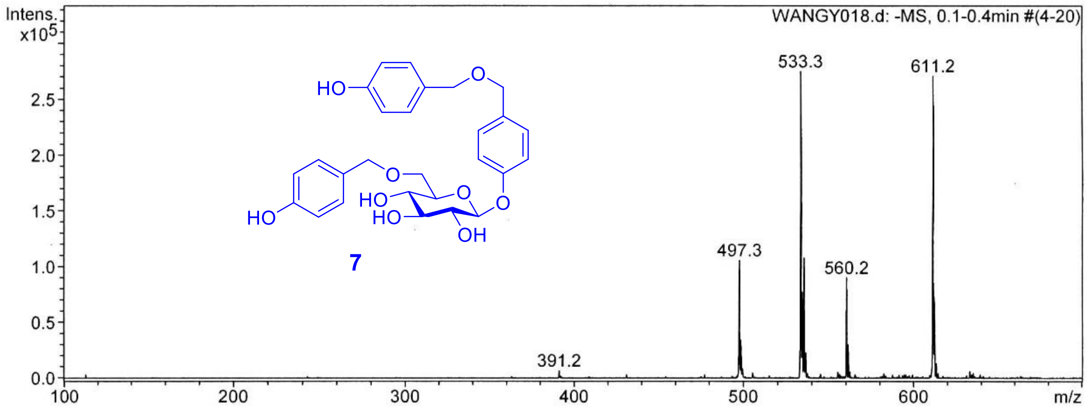

Component	Molecular Mass	Molecule	Absolute Abundance	Relative Abundance

MSD Trap Report v 4 (A4-Opt2) Page 1 of 1 Agilent Technologies

Fig. S71 The ESIMS of compound **7**

Qualitative Analysis Report

**Data Filename**	201101179.d	**Sample Name**	WYN-59
**Sample Type**	Sample	**Position**	P1-C9
**Instrument Name**	Instrument 1	**User Name**	
**Acq Method**		**IRM Calibration Status**	Success
**DA Method**	TEST LCMS.m	**Comment**	

User Chromatograms

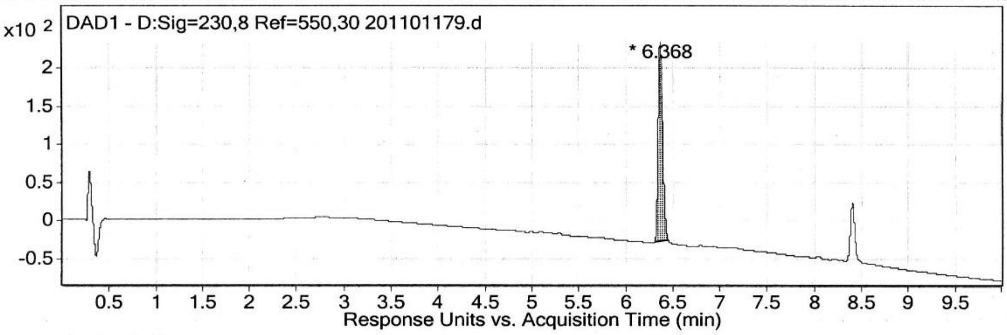

Integration Peak List

Peak	Start	RT	End	Height	Area	Area %
1	6.312	6.368	6.452	261.14	855.35	100

Fragmentor Voltage 135 **Collision Energy** 0 **Ionization Mode** ESI

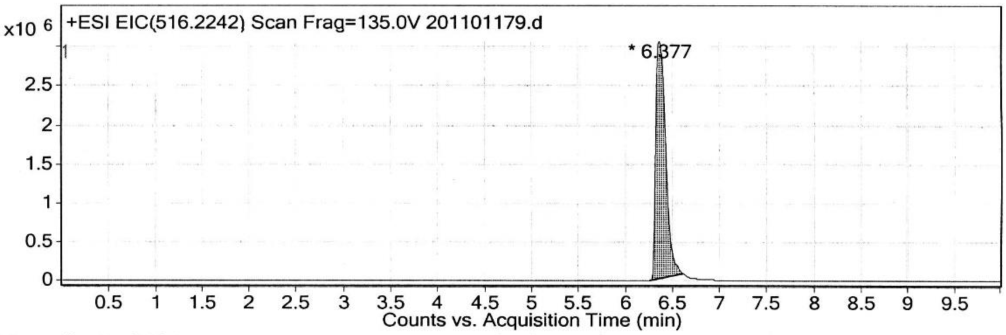

Integration Peak List

Peak	Start	RT	End	Height	Area	Area %
1	6.265	6.377	6.618	3041867	21737360	100

User Spectra

Fragmentor Voltage	Collision Energy	Ionization Mode
135	0	Esi

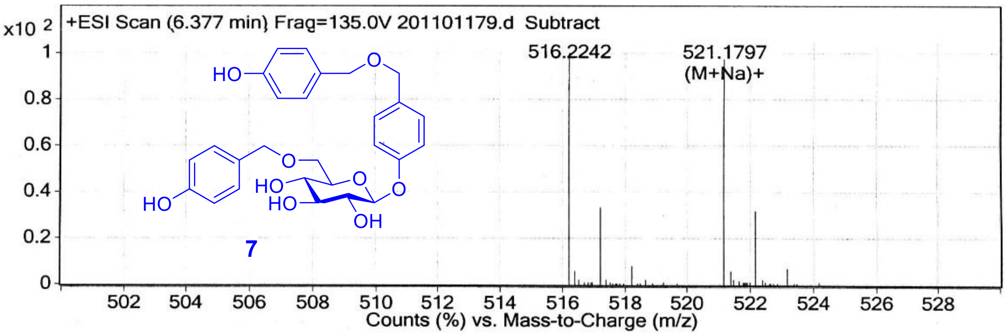

Fig. S72 The (+)-HRESIMS report of compound **7**, Page 1

MS Formula Results: + Scan (6.377 min) Sub (201101179.d)

m/z	Ion	Formula	Abundance
521.1797	(M+Na)+	C27 H30 Na O9	2990242.5

Best	Formula (M)	Ion Formula	Calc m/z	Score	Cross S	Mass	Calc Mass	Diff (ppm)	Abs Diff (ppm)	Abund Match	Spacing Mat	Mass Match	m/z	DBE
☑	C27 H30 O9	C27 H30 Na O9	521.1782	99.68		498.1905	498.189	-3.13	3.13	99.49	99.9	99.69	521.1797	13
☐	C33 H26 N2 O3	C33 H26 N2 Na O3	521.1836	98.75		498.1905	498.1943	7.62	7.62	98.75	99.88	98.19	521.1797	22
☐	C20 H34 O14	C20 H34 Na O14	521.1841	96.64		498.1905	498.1949	8.66	8.66	92.21	99.9	97.66	521.1797	4
☐	C15 H34 N2 O16	C15 H34 N2 Na O16	521.1801	95.05		498.1905	498.1908	0.57	0.57	82.84	99.82	99.99	521.1797	0

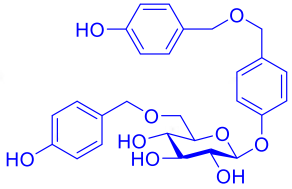

7

page 1

Fig. S73 The (+)-HRESIMS report of compound **7**, Page 2

H1DMSO1227-WYN-59
INOVA-501 1H-NMR WYN-59 IN DMSO 2010.12.24 cold probe

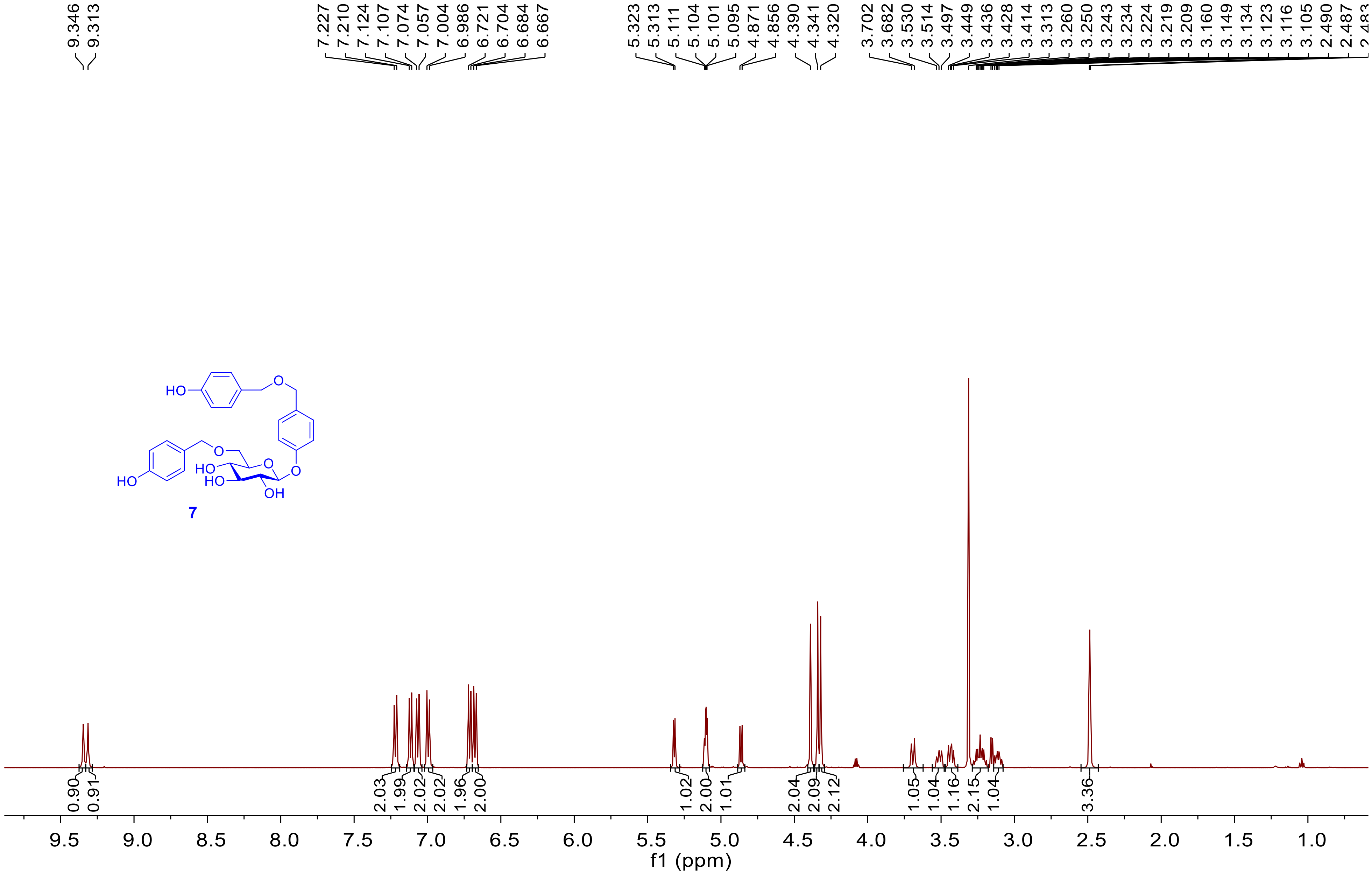

7

Fig. S74 The ^{1}H NMR spectrum of compound **7** in DMSO-d_6 at 500 MHz

C13DMSO1208-WYN-59
INOVA-501 13C-NMR WYN-59 IN DMSO 2010.12.08

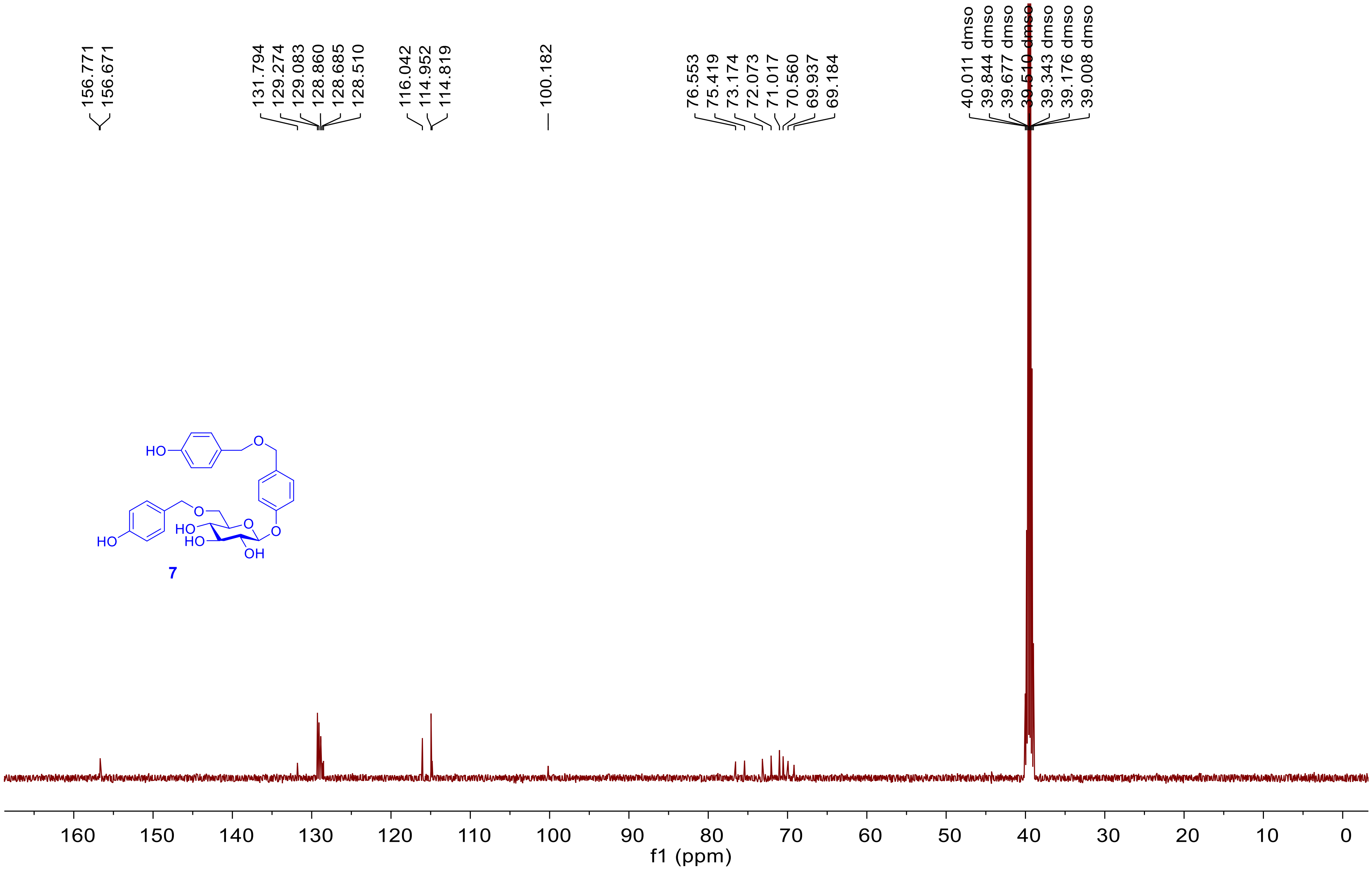

Fig. S75 The ^{13}C NMR spectrum of compound **7** in DMSO-d_6 at 125 MHz

C13DMSO1208-WYN-59
INOVA-501 DEPT WYN-59 IN DMSO 2010.12.08

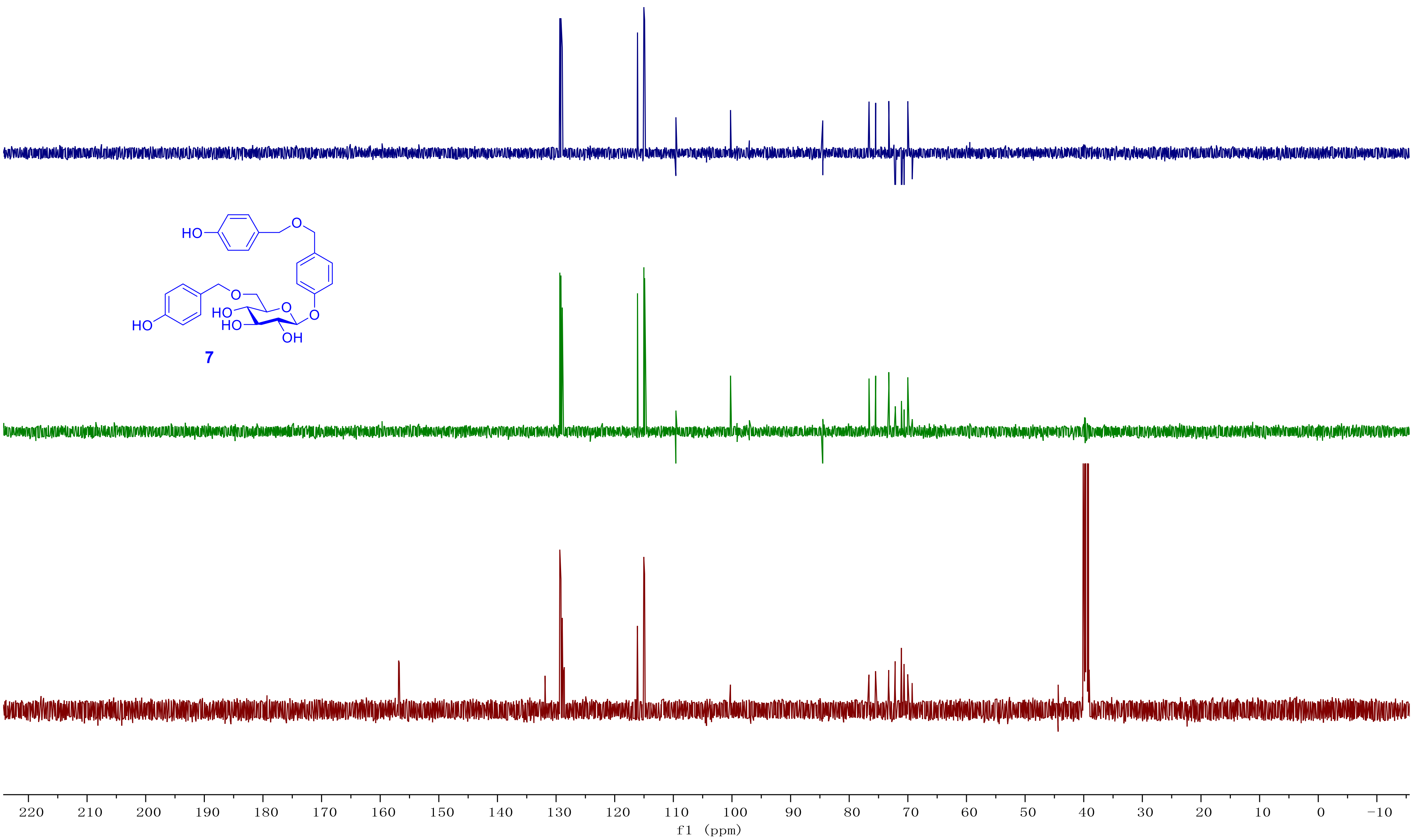

Fig. S76 The DEPT spectrum of compound **7** in DMSO-d_6 at 125 MHz

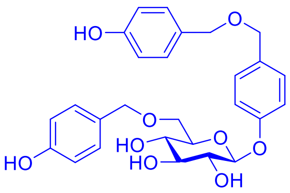

7

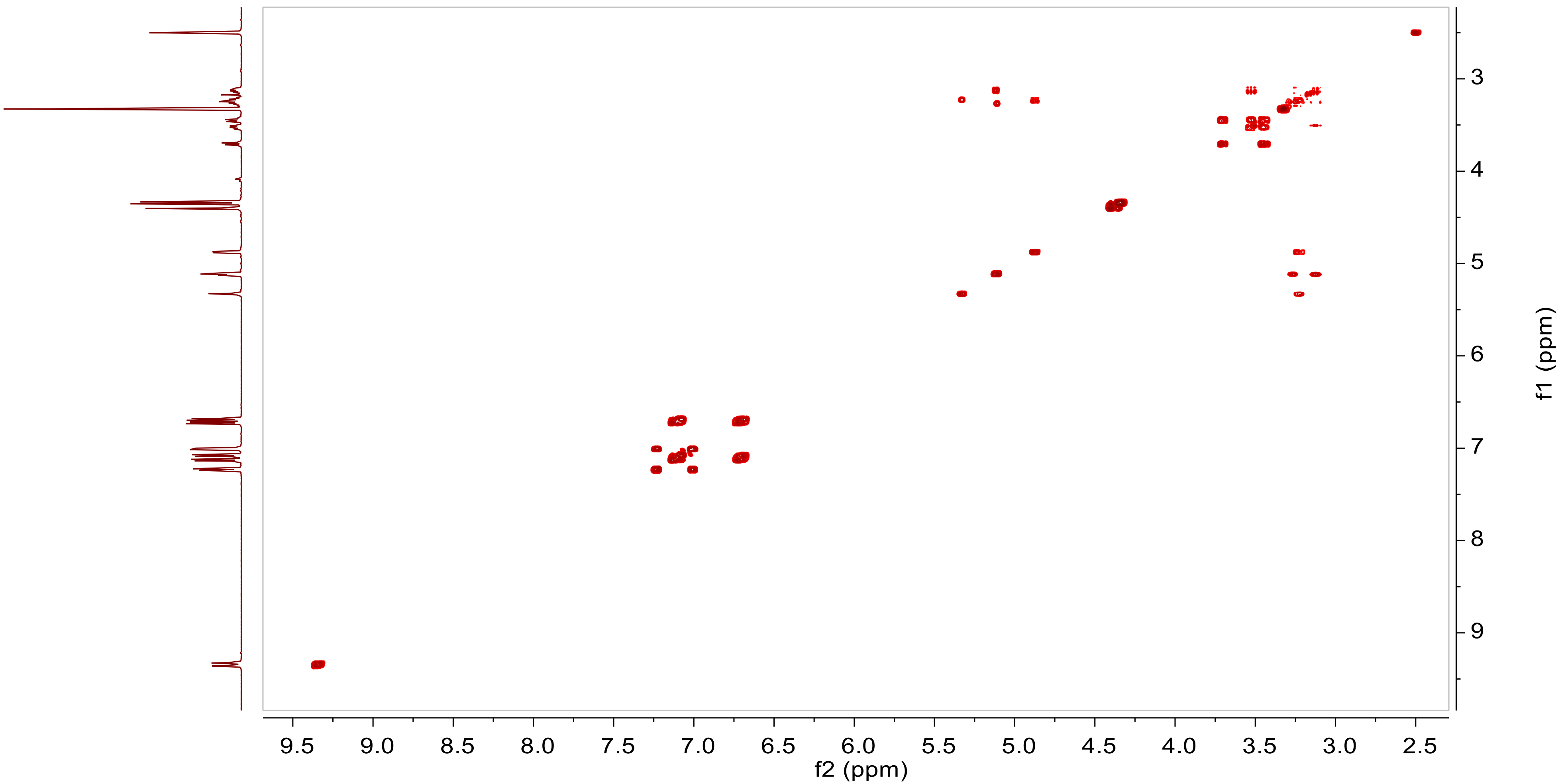

Fig. S77 The ^{1}H-^{1}H COSY spectrum of compound **7** in DMSO-d_6 at 500 MHz

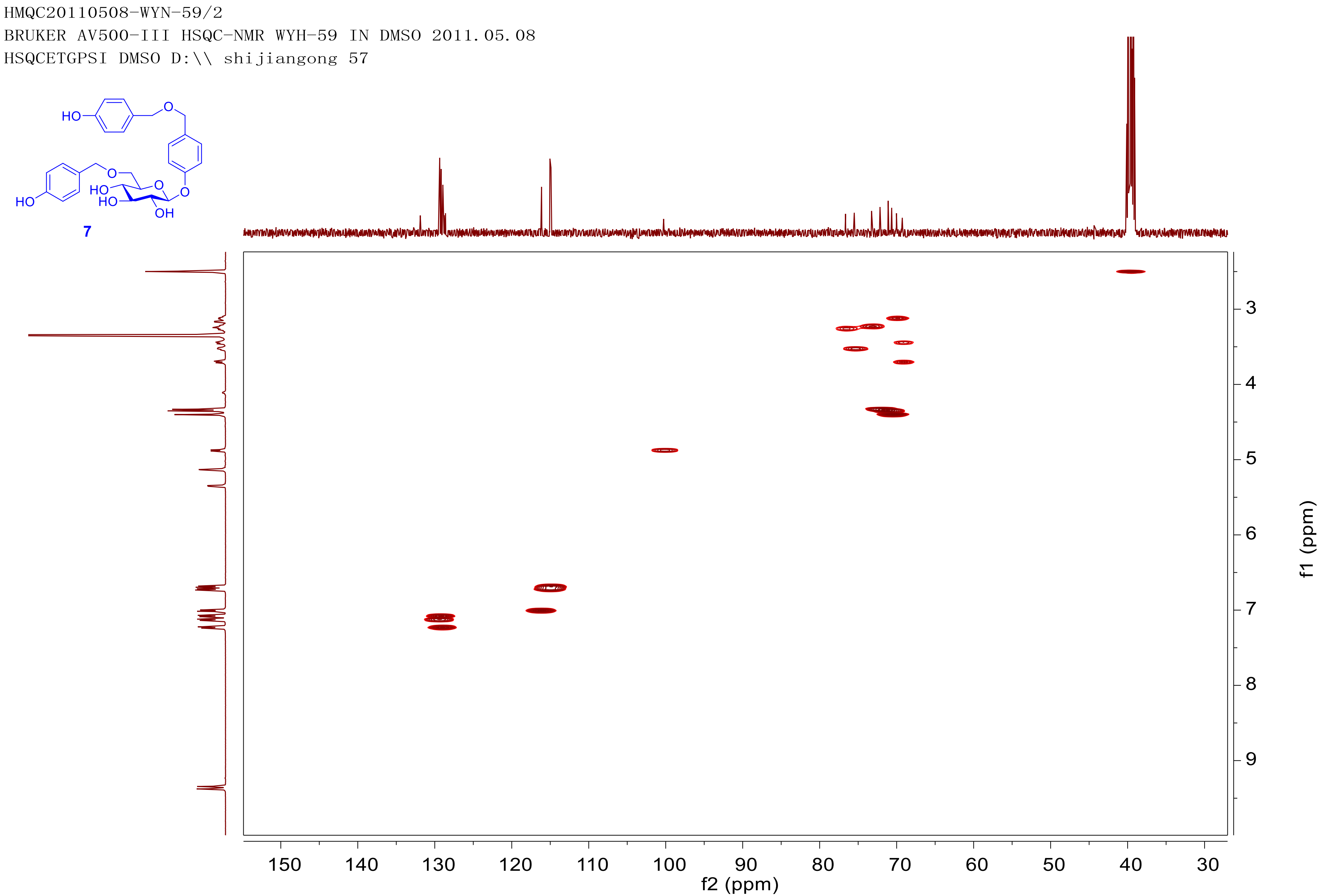

Fig. S78 The HMQC spectrum of compound **7** in DMSO-d_6 (500 MHz for ^{1}H)

HMBCDMSO1227-WYN-59
INOVA-501 gHMBC WYN-59 IN DMSO 2010.12.24 cold probe

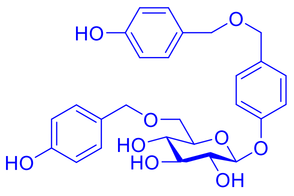

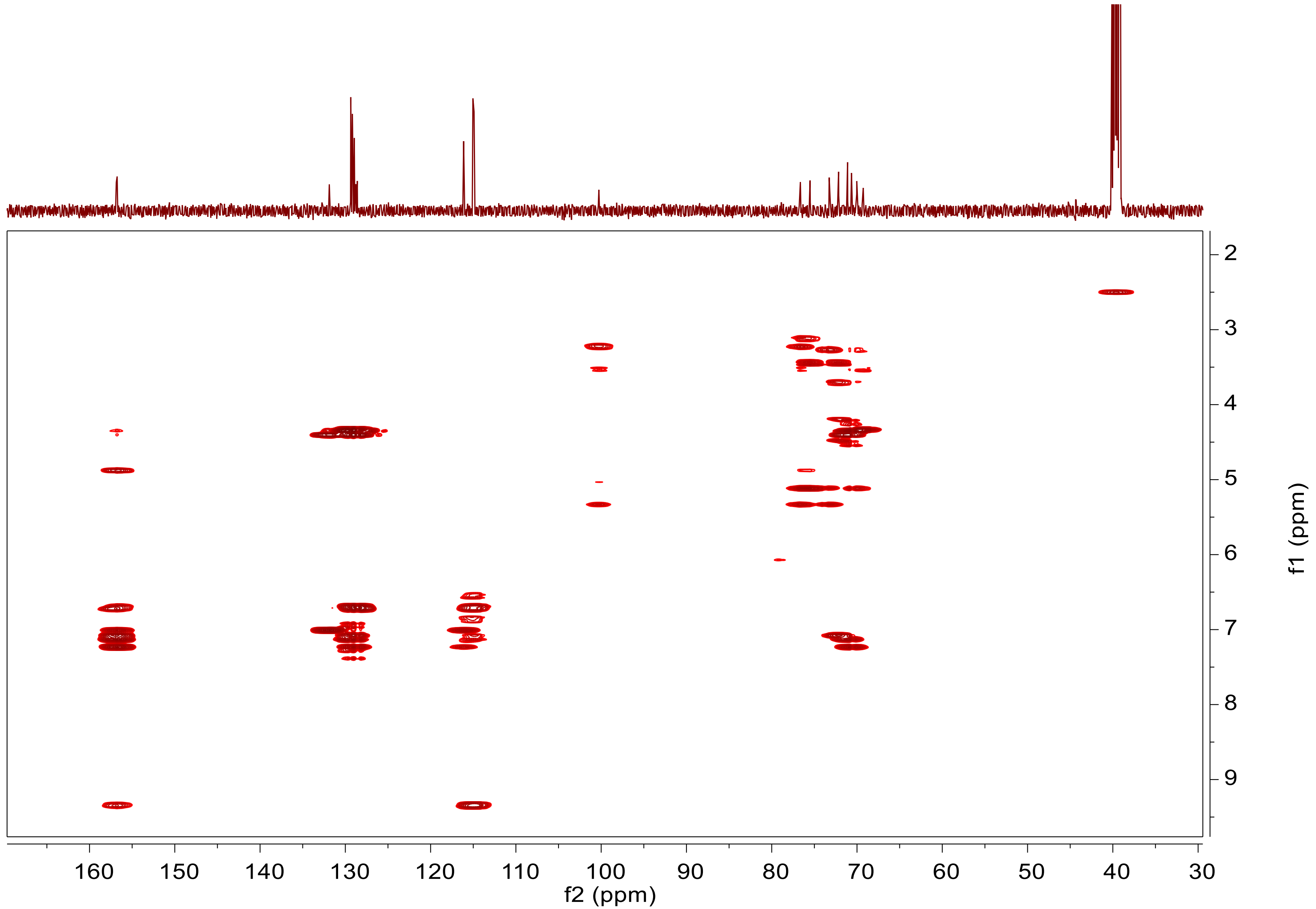

Fig. S79 The HMBC spectrum of compound **7** in DMSO-d_6 (500 MHz for ^{1}H)

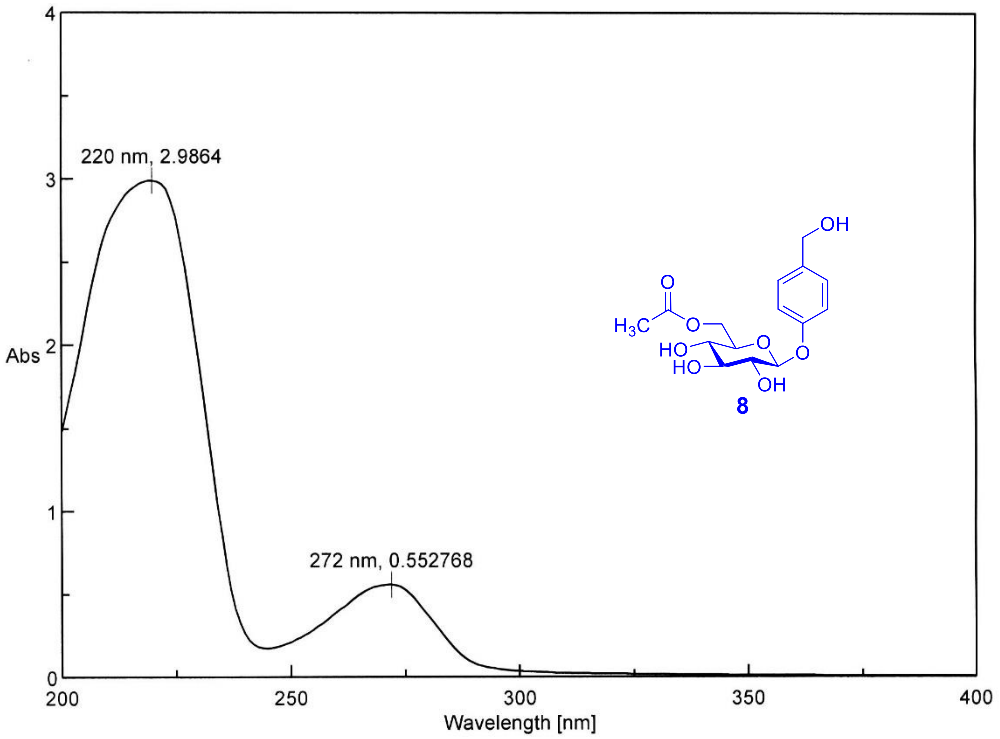

—— Memory-10

[Comment]

Sample Name	WYN-47B
Comment	
User	wangyanan
Division	
Company	324

[Measurement Information]

Instrument Name	V-650
Model Name	V-650
Serial No.	A034461150
Accessory	PSC-718
Accessory S/N	A001761114
Position	1
Cell Length	10 mm
Temperature	19.98 C
Control Sonsor	Holder
Monitor Sensor	Holder
Start Mode	Start immediately
Photometric Mode	Abs
Measurement range	400 - 200 nm
Data pitch	1 nm
Band width(UV/Vis)	1.0 nm
Response	Medium
Scanning speed	200 nm/min
Source Change	340 nm
Light Source	D2/WI
Filter Exchange	Step
Correction	Baseline

[Data Information]

Creation Date	2011-11-23 11:23
Data array type	Linear data array
Horizontal	Wavelength [nm]
Vertical	Abs
Start	400 nm
End	200 nm
Data pitch	1 nm
Data points	201

Fig. S80 The UV spectrum of compound **8**

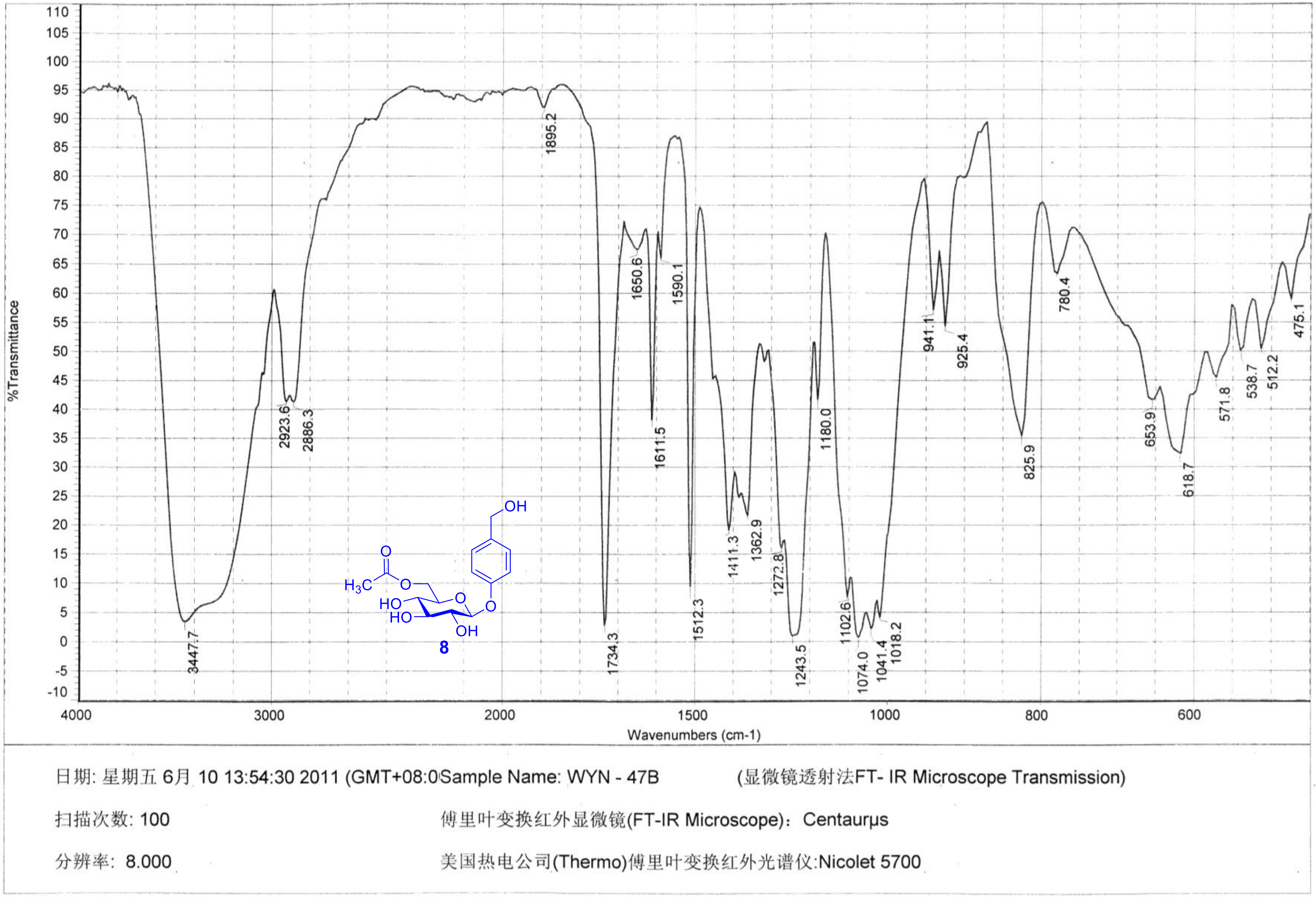

Fig. S81 The IR spectrum of compound **8**

Single Mass Spectrum Deconvolution Report

Analysis Name: WANGY011.d **Instrument:** LC-MSD-Trap-SL **Print Date:** 7/9/2010 12:01:53 PM
Method: TEST.MS **Operator:** Operator **Acq. Date:** 7/9/2010 11:55:59 AM
Sample Name: WYN-47B
Analysis Info:

Acquisition Parameter:

Mass Range Mode	Std/Normal	Trap Drive	53.0	Scan Begin	100 m/z
Ion Polarity	Positive	Octopole RF Amplitude	171.0 Vpp	Scan End	700 m/z
Ion Source Type	ESI	Capillary Exit	-106.0 Volt	Averages	5 Spectra
Dry Temp (Set)	330 °C	Skimmer	-40.0 Volt	Max. Accu Time	200000 µs
Nebulizer (Set)	15.00 psi	Oct 1 DC	-12.00 Volt	ICC Target	20000
Dry Gas (Set)	5.00 l/min	Oct 2 DC	-1.70 Volt	Charge Control	on

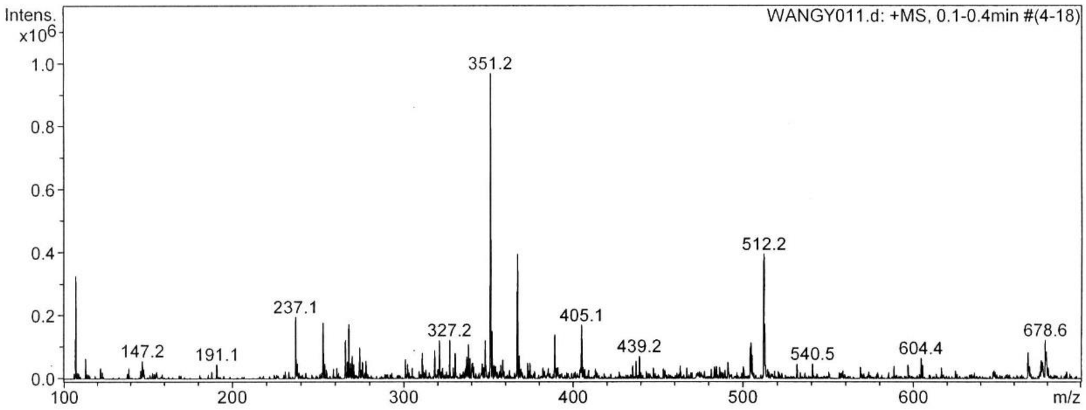

Component	Molecular Mass	Molecule	Absolute Abundance	Relative Abundance

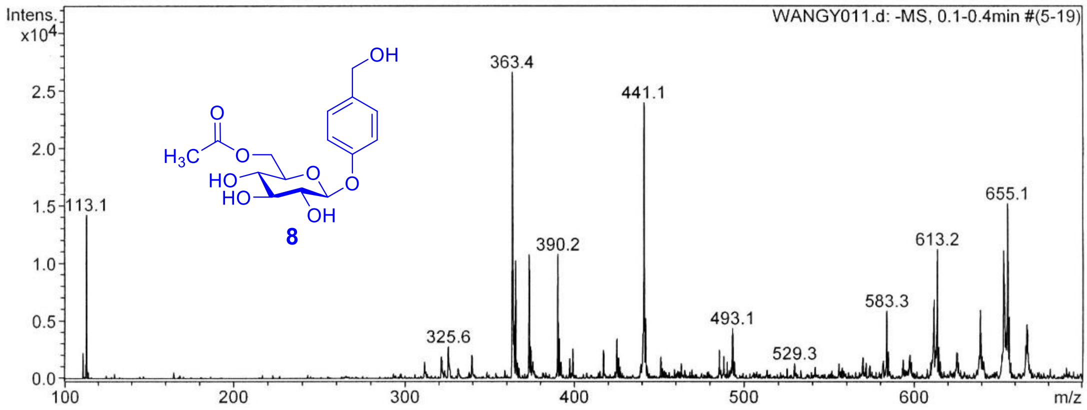

Component	Molecular Mass	Molecule	Absolute Abundance	Relative Abundance

Fig. S82 The ESIMS of compound **8**

Qualitative Analysis Report

**Data Filename**	201109022.d	**Sample Name**	WYN-47B
**Sample Type**	Sample	**Position**	P1-F3
**Instrument Name**	Instrument 1	**User Name**	
**Acq Method**		**IRM Calibration Status**	Success
**DA Method**	TEST LCMS.m	**Comment**	

User Chromatograms

Fragmentor Voltage 135 **Collision Energy** 0 **Ionization Mode** ESI

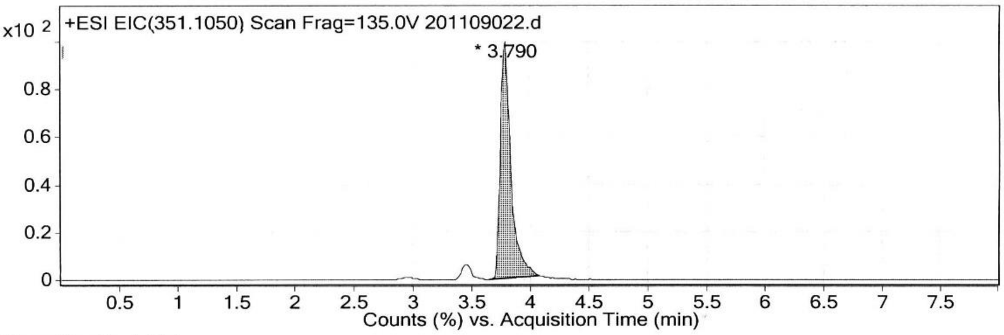

Integration Peak List

Peak	Start	RT	End	Height	Area	Area %
1	3.645	3.79	4.079	629563	3973484	100

User Spectra

Fragmentor Voltage	Collision Energy	Ionization Mode
135	0	Esi

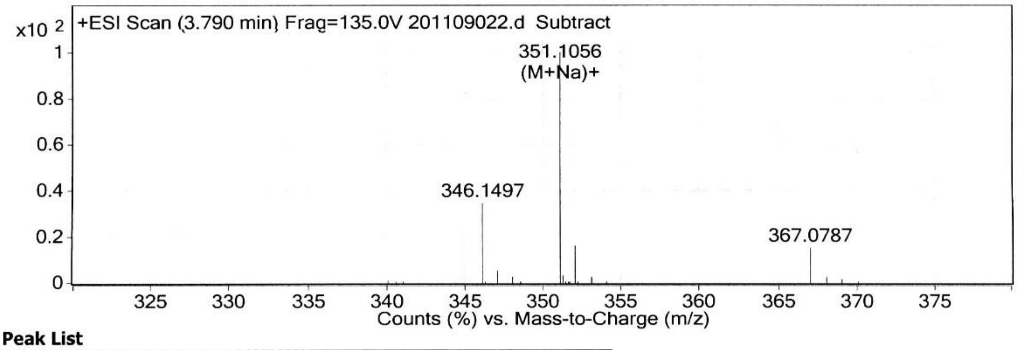

Peak List

m/z	z	Abund	Formula	Ion
346.1497	1	217735		
347.1523	1	35613		
351.1056	1	632368	C15 H20 Na O8	(M+Na)+
352.1082	1	102519	C15 H20 Na O8	(M+Na)+
367.0787		95101		

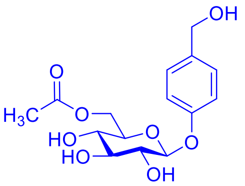

8

Formula Calculator Element Limits

Element	Min	Max
C	3	100
H	0	120
O	0	30
N	0	5
S	0	2
Cl	0	0

Formula Calculator Results

Formula	Best	Mass	Tgt Mass	Diff (ppm)	Ion Species	Score
C15 H20 O8	TRUE	328.1164	328.1158	-1.7	C15 H20 Na O8	99.92

Fig. S83 The (+)-HRESIMS report of compound **8**, Page 1

MS Formula Results: + Scan (3.790 min) Sub (201109022.d)

m/z	Ion	Formula	Abundance
351.1056	(M+Na)+	C15 H20 Na O8	632367.9

Best	Formula (M)	Ion Formula	Calc m/z	Score	Cross S	Mass	Calc Mass	Diff (ppm)	Abs Diff (ppm)	Abund Match	Spacing Mat	Mass Match	m/z	DBE
☑	C15 H20 O8	C15 H20 Na O8	351.105	99.92		328.1164	328.1158	-1.7	1.7	99.96	99.87	99.92	351.1056	6
☐	C16 H16 N4 O4	C16 H16 N4 Na O4	351.1064	99.6		328.1164	328.1172	2.37	2.37	98.87	99.98	99.84	351.1056	11
☐	C12 H24 O8 S	C12 H24 Na O8 S	351.1084	97.93		328.1164	328.1192	8.57	8.57	96.41	99.71	97.95	351.1056	1
☐	C19 H20 O3 S	C19 H20 Na O3 S	351.1025	97.41		328.1164	328.1133	-9.33	9.33	95.17	99.75	97.58	351.1056	10
☐	C16 H24 O3 S2	C16 H24 Na O3 S2	351.1059	97.25		328.1164	328.1167	0.94	0.94	90.72	99.62	99.97	351.1056	5

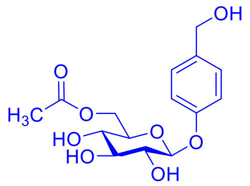

8

page 1

Fig. S84 The (+)-HRESIMS report of compound **8**, Page 1

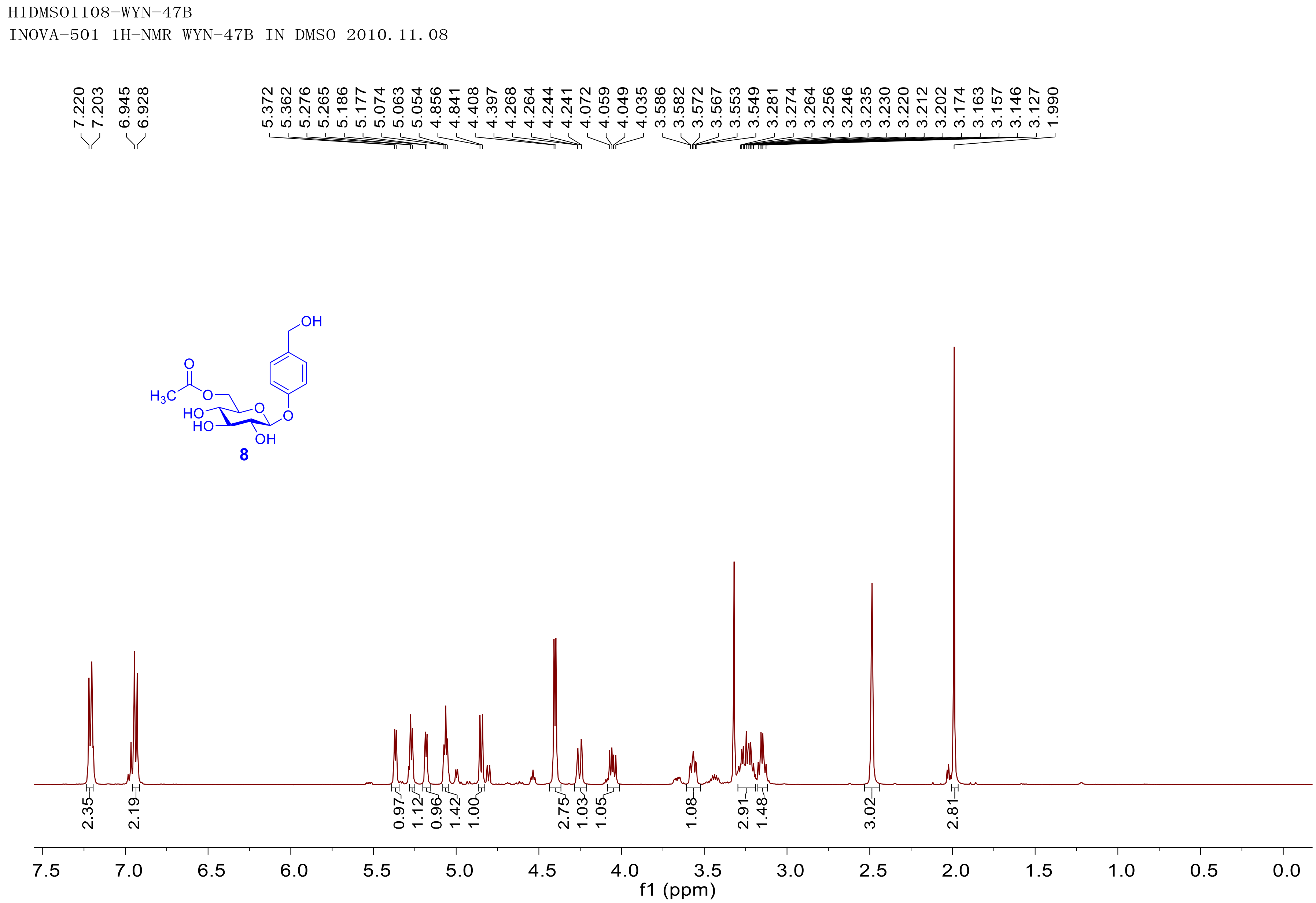

Fig. S85 The ^{1}H NMR spectrum of compound **8** in DMSO-d_6 at 500 MHz

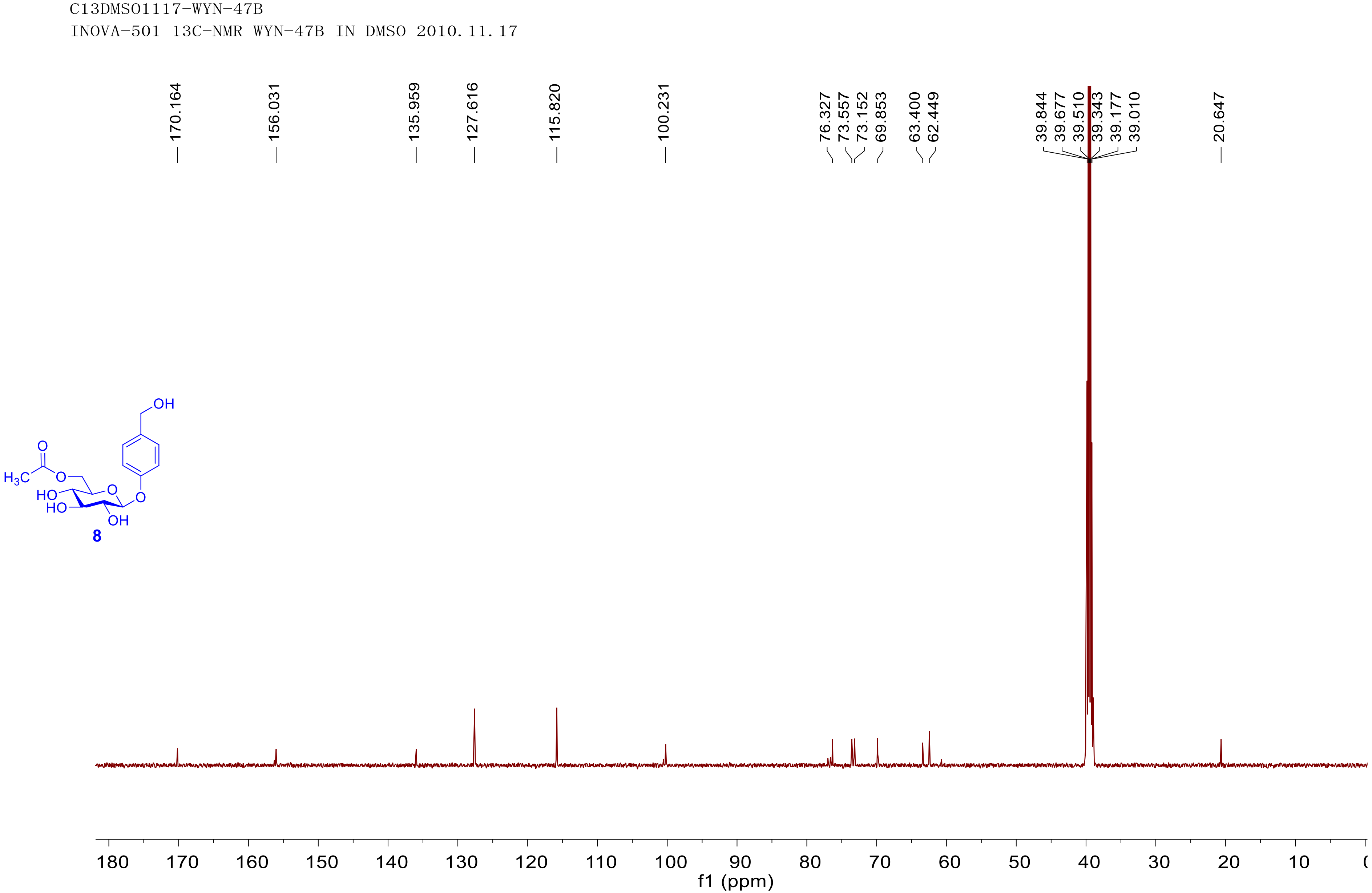

Fig. S86 The ^{13}C NMR spectrum of compound **8** in DMSO-d_6 at 125 MHz

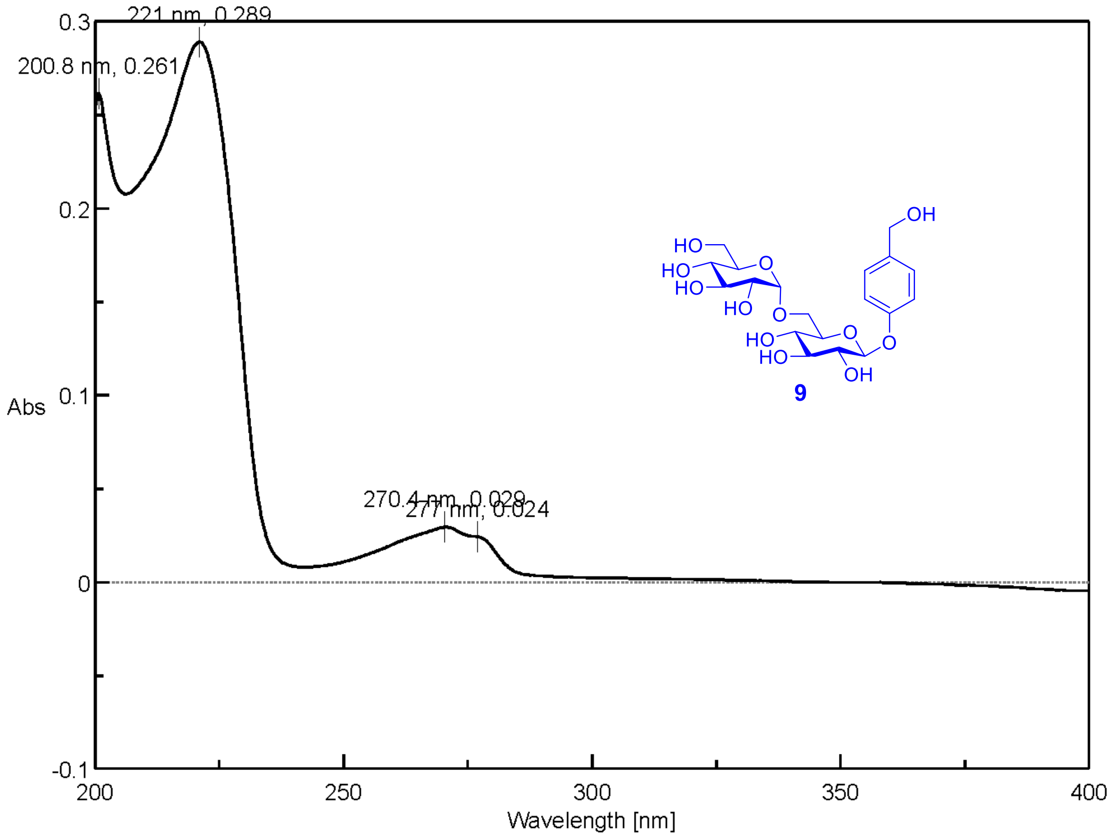

[Comment]

Sample Name	DBT
Comment	0.02
User	
Division	UV
Company	324

wyn-52

[Measurement Information]

Instrument Name	V-650
Model Name	V-650
Serial No.	A034461150
Accessory	PSC-718
Accessory S/N	A001761114
Position	1
Cell Length	10 mm
Temperature	20.00 C
Control Sonsor	Holder
Monitor Sensor	Holder
Start Mode	Start immediately
Photometric Mode	Abs
Measurement range	600 - 190 nm
Data pitch	0.2 nm
Band width(UV/Vis)	2.0 nm
Response	Medium
Scanning speed	200 nm/min
Source Change	340 nm
Light Source	D2/WI
Filter Exchange	Step
Correction	Baseline

[Data Information]

Creation Date	2019-10-11 16:24
Data array type	Linear data array
Horizontal	Wavelength [nm]
Vertical	Abs
Start	600 nm
End	190 nm
Data pitch	0.2 nm
Data points	2051

Fig. S87 The UV spectrum of compound **9** in MeOH

Single Mass Spectrum Deconvolution Report

Analysis Name: linsh033.d **Instrument:** LC-MSD-Trap-SL **Print Date:** 1/5/2011 11:35:42 AM
Method: def_lcms.m **Operator:** Operator **Acq. Date:** 1/5/2011 11:33:51 AM
Sample Name: TM-10
Analysis Info:

Acquisition Parameter:

Mass Range Mode	Std/Normal	Trap Drive	53.0	Scan Begin	100 m/z
Ion Polarity	Positive	Octopole RF Amplitude	171.0 Vpp	Scan End	1200 m/z
Ion Source Type	ESI	Capillary Exit	-106.0 Volt	Averages	5 Spectra
Dry Temp (Set)	330 °C	Skimmer	-40.0 Volt	Max. Accu Time	200000 µs
Nebulizer (Set)	15.00 psi	Oct 1 DC	-12.00 Volt	ICC Target	10000
Dry Gas (Set)	5.00 l/min	Oct 2 DC	-1.70 Volt	Charge Control	on

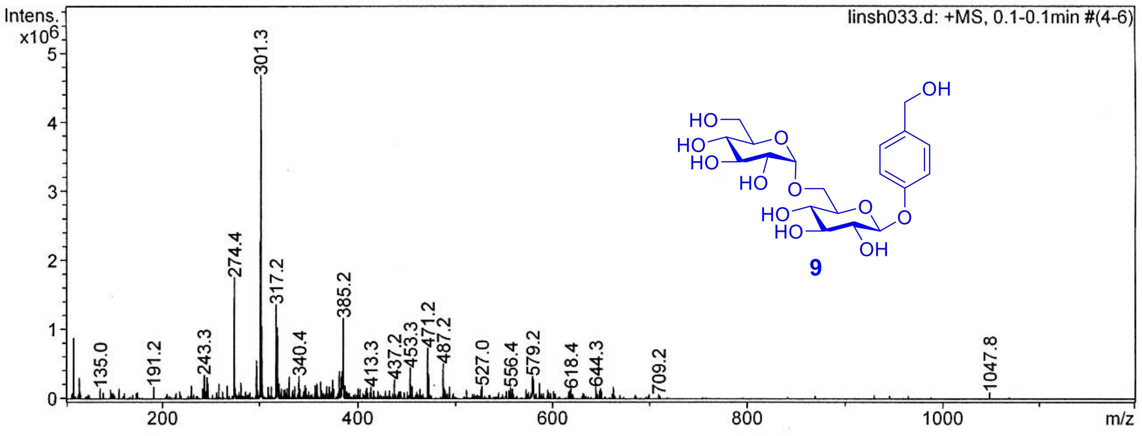

Component Molecular Mass Molecule Absolute Abundance Relative Abundance

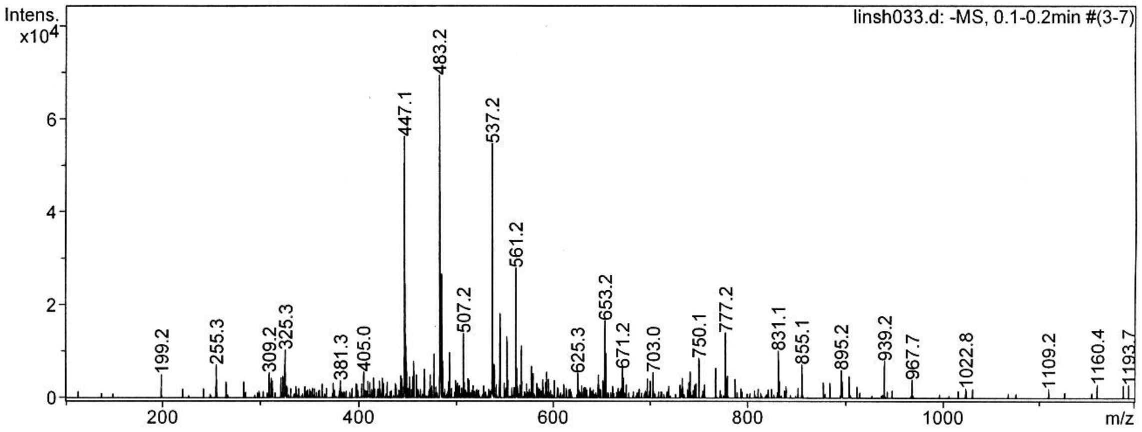

Component Molecular Mass Molecule Absolute Abundance Relative Abundance

MSD Trap Report v 4 (A4-Opt2) Page 1 of 1 Agilent Technologies

Fig. S88 The ESIMS of compound **9**

Qualitative Analysis Report

**Data Filename**	2012122401.d	**Sample Name**	GE-7
**Sample Type**	Sample	**Position**	P1-D6
**Instrument Name**	Instrument 1	**User Name**	
**Acq Method**		**IRM Calibration Status**	Some Ions Missed
**DA Method**	TEST LCMS.m	**Comment**	

User Chromatograms

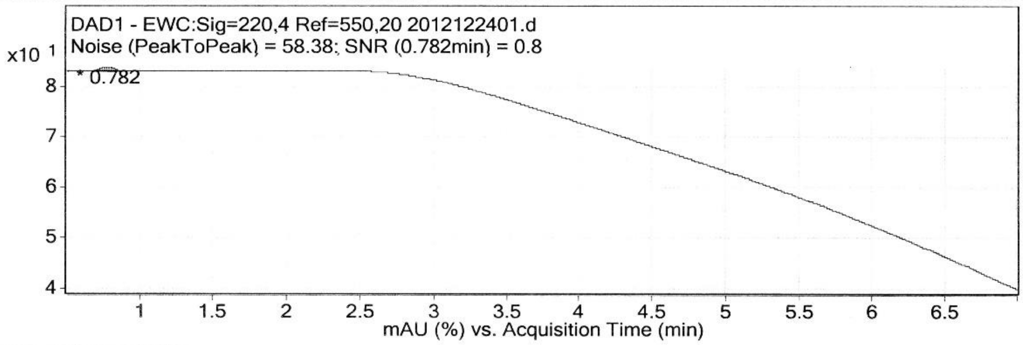

Integration Peak List

Peak	Start	RT	End	Height	Area	Area %	Signal To Noise
1	0.682	0.782	0.913	7.06	44.617	100	0.8

Noise Measurements

Noise Type	Signal Definition	Noise Multiplier	Noise Value
Peak-to-Peak	Area	1	58.37646484

Noise Regions

Start	End
1	2
6.5	7
9.6	10.4
9.8	11

Fragmentor Voltage 135 **Collision Energy** 0 **Ionization Mode** ESI

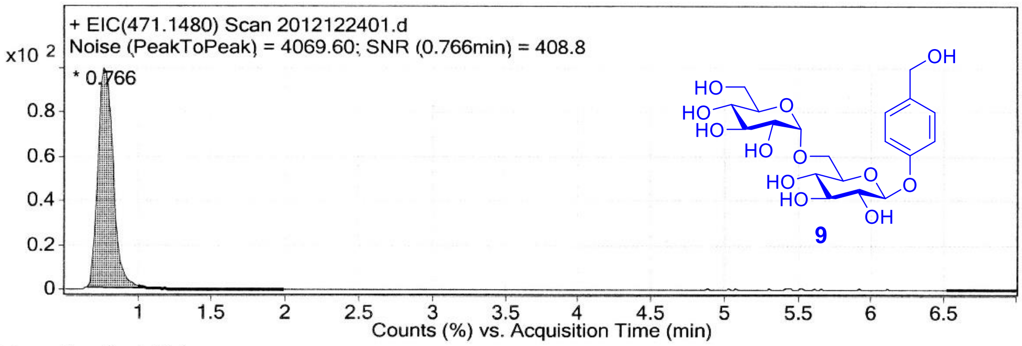

Integration Peak List

Peak	Start	RT	End	Height	Area	Area %	Signal To Noise
1	0.653	0.766	1.072	226820	1663708	100	408.8

Noise Measurements

Noise Type	Signal Definition	Noise Multiplier	Noise Value
Peak-to-Peak	Area	1	4069.595215

Noise Regions

Start	End
1	2
6.5	7
9.6	10.4
9.8	11

Fig. S89 The (+)-HRESIMS report of compound **9**, Page 1

Qualitative Analysis Report

User Spectra

Fragmentor Voltage	Collision Energy	Ionization Mode
135	0	Esi

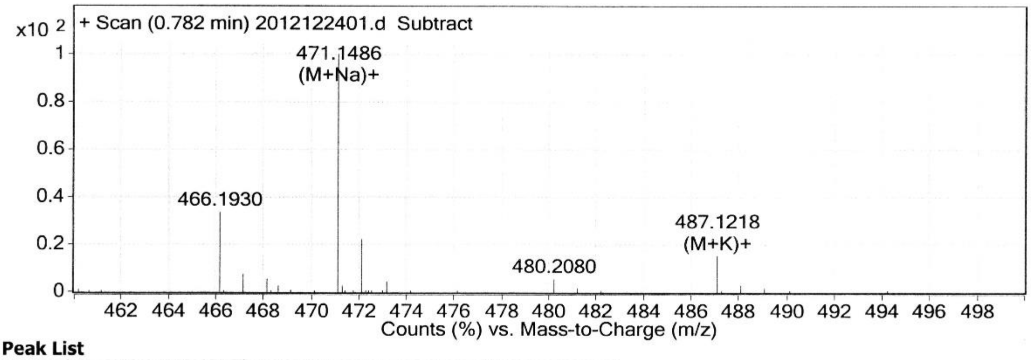

Peak List

m/z	z	Abund	Formula	Ion
107.0492		56969		
244.0599	2	47256		
244.5623	2	11944		
275.079		13574		
466.193	1	75029		
467.196	1	16495		
471.1486	1	222775	C19 H28 Na O12	(M+Na)+
472.1515	1	48949	C19 H28 Na O12	(M+Na)+
480.208		12477		
487.1218	1	33671	C19 H28 K O12	(M+K)+

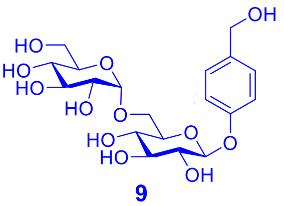

9

Formula Calculator Element Limits

Element	Min	Max
C	3	100
H	0	500
O	0	90
N	0	3
S	0	0
Cl	0	0
Br	0	1
Si	0	1

Formula Calculator Results

Formula	Best	Mass	Tgt Mass	Diff (ppm)	Ion Species	Score
C19 H28 O12	TRUE	448.1594	448.1581	-2.89	C19 H28 Na O12	99.83
C15 H32 O13 Si		448.1594	448.1612	4.1	C15 H32 Na O13 Si	99.05
C19 H28 O12	TRUE	448.1586	448.1581	-1.17	C19 H28 K O12	99.8
C15 H32 O13 Si		448.1586	448.1612	5.82	C15 H32 K O13 Si	98.66

--- End Of Report ---

Fig. S90 The (+)-HRESIMS report of compound **9**, Page 2

MS Formula Results: + Scan (0.782 min) Sub (2012122401.d)

m/z	Ion	Formula	Abundance
471.1486	(M+Na)+	C19 H28 Na O12	222775.1

Best	Formula (M)	Ion Formula	Calc m/z	Score	Cross S	Mass	Calc Mass	Diff (ppm)	Abs Diff (ppm)	Abund Match	Spacing Mat	Mass Match	m/z	DBE
☑	C19 H28 O12	C19 H28 Na O12	471.1473	99.83		448.1594	448.1581	-2.89	2.89	99.84	99.97	99.74	471.1486	6
☐	C15 H32 O13 Si	C15 H32 Na O13 Si	471.1504	99.05		448.1594	448.1612	4.1	4.1	97.7	99.81	99.48	471.1486	1

m/z	Ion	Formula	Abundance
487.1218	(M+K)+	C19 H28 K O12	33671.3

Best	Formula (M)	Ion Formula	Calc m/z	Score	Cross S	Mass	Calc Mass	Diff (ppm)	Abs Diff (ppm)	Abund Match	Spacing Mat	Mass Match	m/z	DBE
☑	C19 H28 O12	C19 H28 K O12	487.1212	99.8		448.1586	448.1581	-1.17	1.17	99.42	99.94	99.96	487.1218	6
☐	C15 H32 O13 Si	C15 H32 K O13 Si	487.1244	98.66		448.1586	448.1612	5.82	5.82	97.04	99.87	99.03	487.1218	1

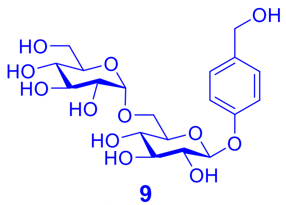

9

page 1

Fig. S91 The (+)-HRESIMS report of compound **9**, Page 3

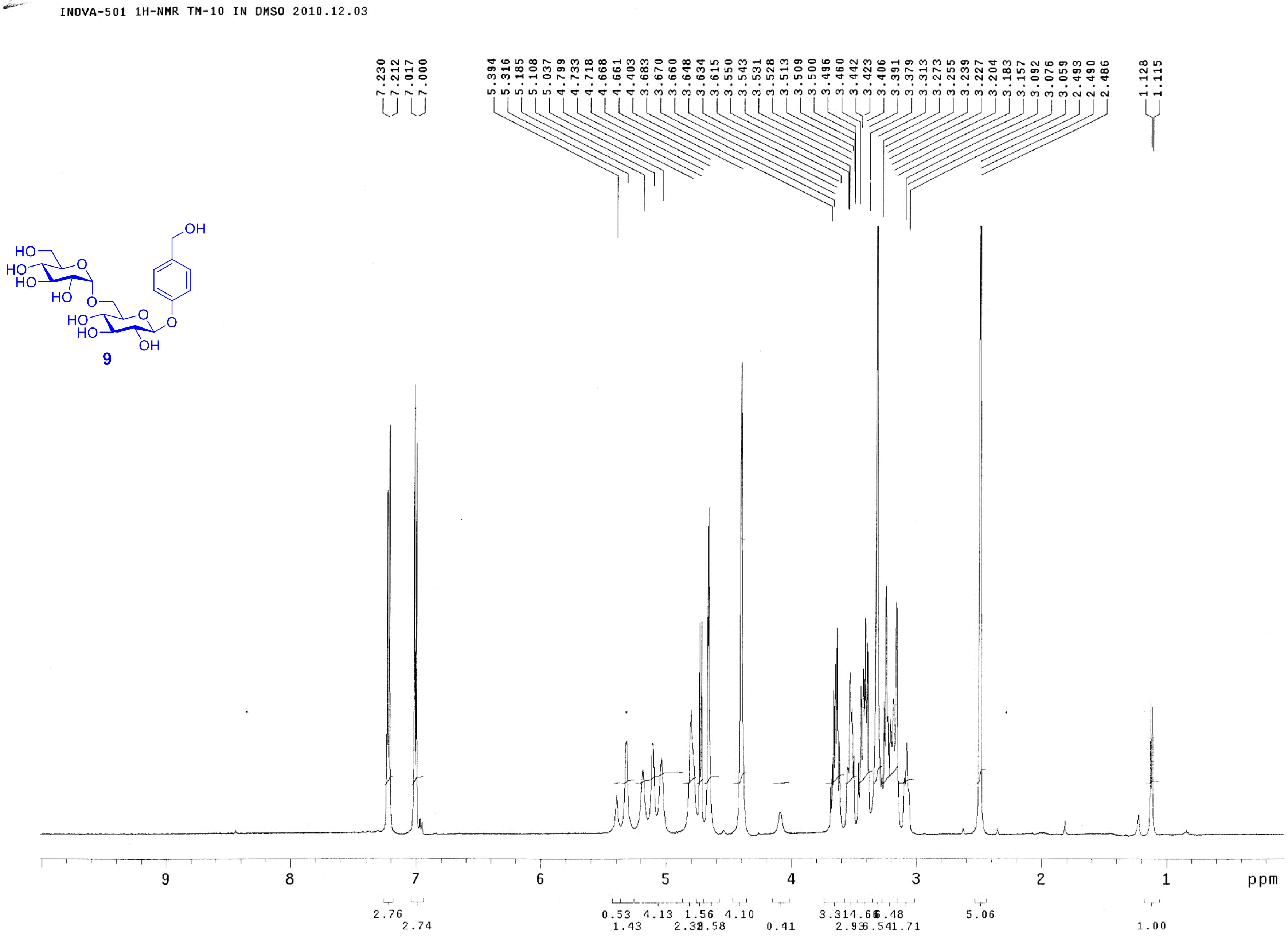

Fig. S92 The ^{1}H NMR spectrum of compound **9** in DMSO-d_6 at 500 MHz

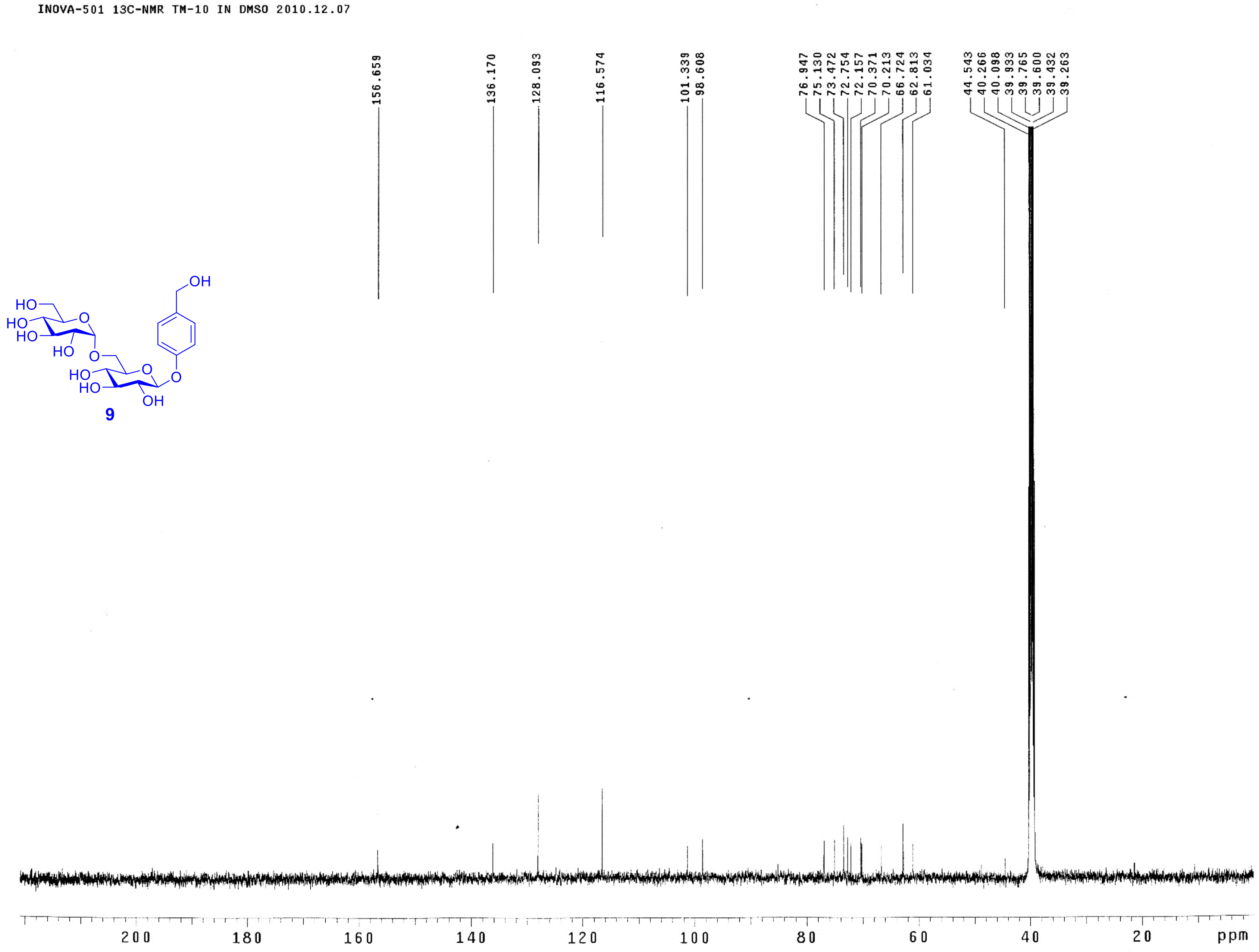

Fig. S93 The ^{13}C NMR spectrum of compound **9** in DMSO-d_6 at 125 MHz

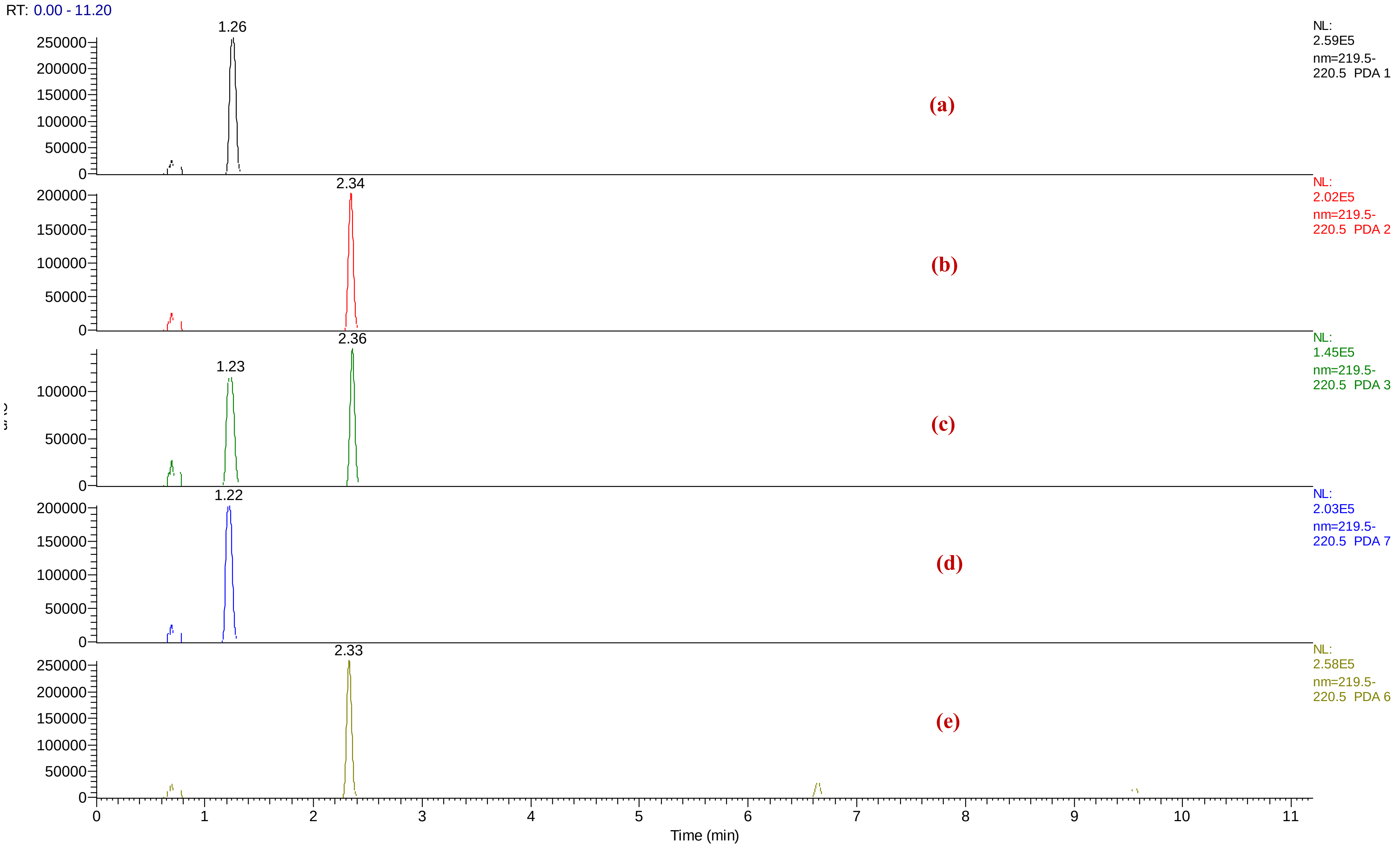

Fig. S94 Overlaid HPLC-UV chromatograms of the reaction mixtures of: (a) gastrodin only in H_2O refluxed for 12 h; (b) D-glucose and 4-hydroxybenzyl alcohol (molar ratio,1:1) in H_2O refluxed for 12 h; (c) gastrodin and 4-hydroxybenzyl alcohol (molar ratio, 1:1) in H_2O refluxed for 12 h; (d) D-glucose and gastrodin (molar ratio, 1:1) in H_2O refluxed for 12 h; (e) 4-hydroxybenzyl alcohol in H_2O refluxed for 12 h

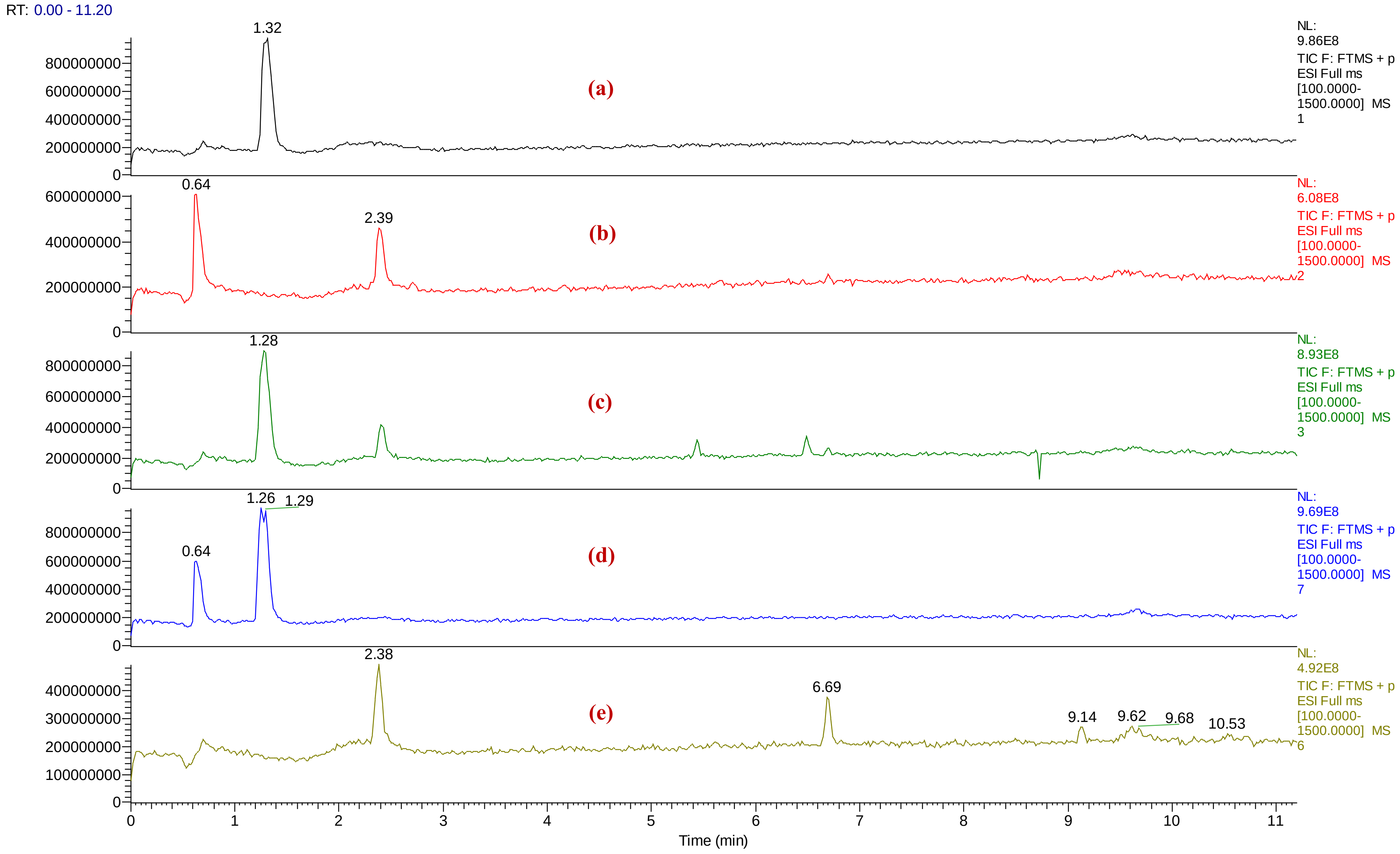

Fig. S95 Overlaid UPLC-HRESIMS (+)-TIC of the reaction mixtures of: (a) gastrodin only in H_2O refluxed for 12 h; (b) D-glucose and 4-hydroxybenzyl alcohol (molar ratio,1:1) in H_2O refluxed for 12 h; (c) gastrodin and 4-hydroxybenzyl alcohol (molar ratio, 1:1) in H_2O refluxed for 12 h; (d) D-glucose and gastrodin (molar ratio, 1:1) in H_2O refluxed for 12 h; (e) 4-hydroxybenzyl alcohol in H_2O refluxed for 12 h

.

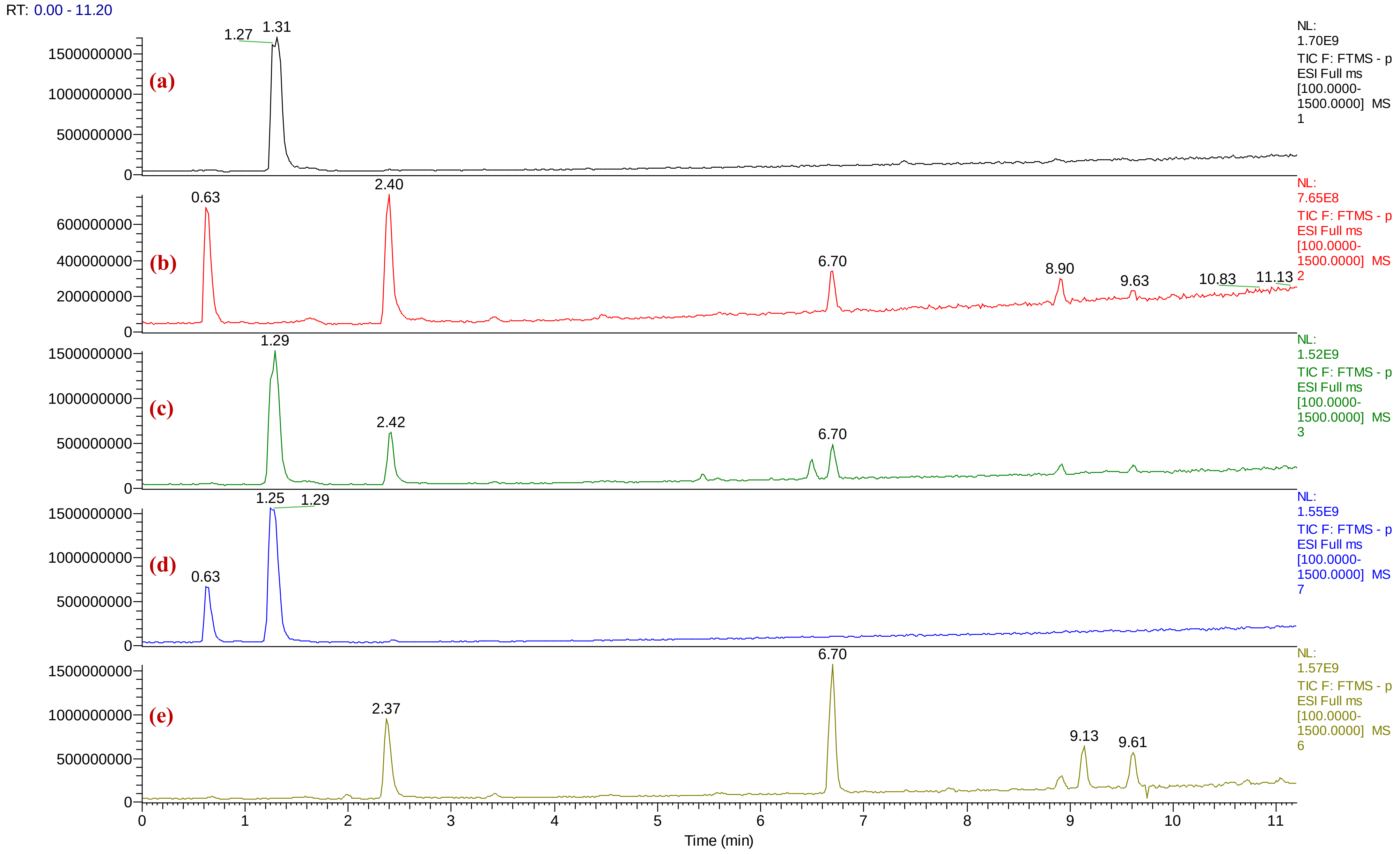

Fig. S96 Overlaid UPLC-HRESIMS (–)-TIC of the reaction mixtures of: (a) gastrodin only in H_2O refluxed for 12 h; (b) D-glucose and 4-hydroxybenzyl alcohol (molar ratio,1:1) in H_2O refluxed for 12 h; (c) gastrodin and 4-hydroxybenzyl alcohol (molar ratio, 1:1) in H_2O refluxed for 12 h; (d) D-glucose and gastrodin (molar ratio, 1:1) in H_2O refluxed for 12 h; (e) 4-hydroxybenzyl alcohol in H_2O refluxed for 12 h

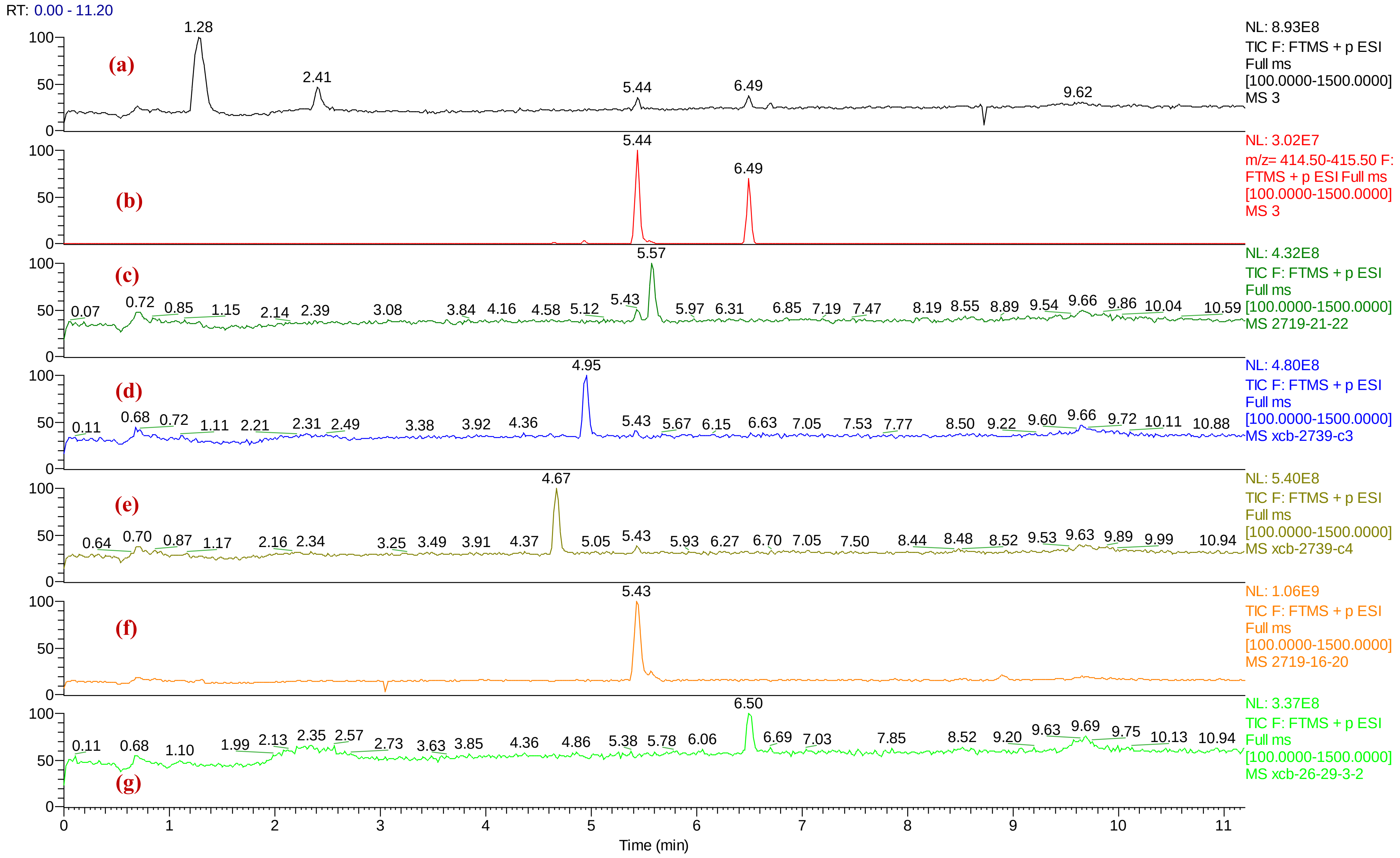

Fig. S97 Overlaid (a) (+)-TIC of the reaction mixture of gastrodin and 4-hydroxybenzyl alcohol (molar ratio, 1:1) in H_2O refluxed for 12 h; (b) the chromatogram of the extracted positive ion at *m/z* 415 $[M + Na]^+$ from (a); (c)–(g) (+)-TIC of aqueous solutions of compounds **1**–**4** and **10**

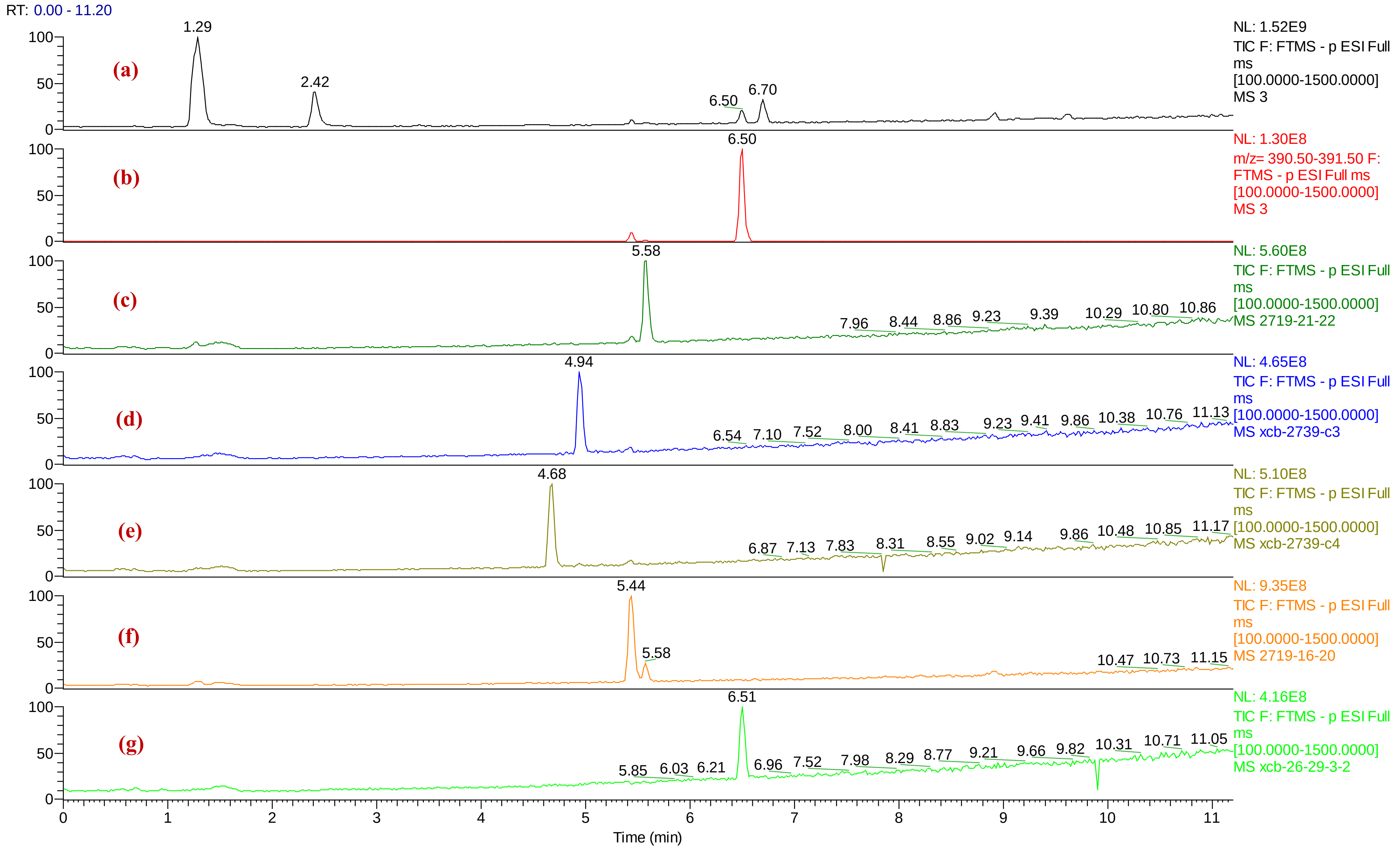

Fig. S98 Overlaid (a) (+)-TIC of the reaction mixture of gastrodin and 4-hydroxybenzyl alcohol (molar ratio, 1:1) in H_2O refluxed for 12 h; (b) the chromatogram of the extracted negative ion at *m/z* 391 [M – H] $^-$ from (a); (c)–(g) (–)-TIC of aqueous solutions of compounds **1**–**4** and **10**

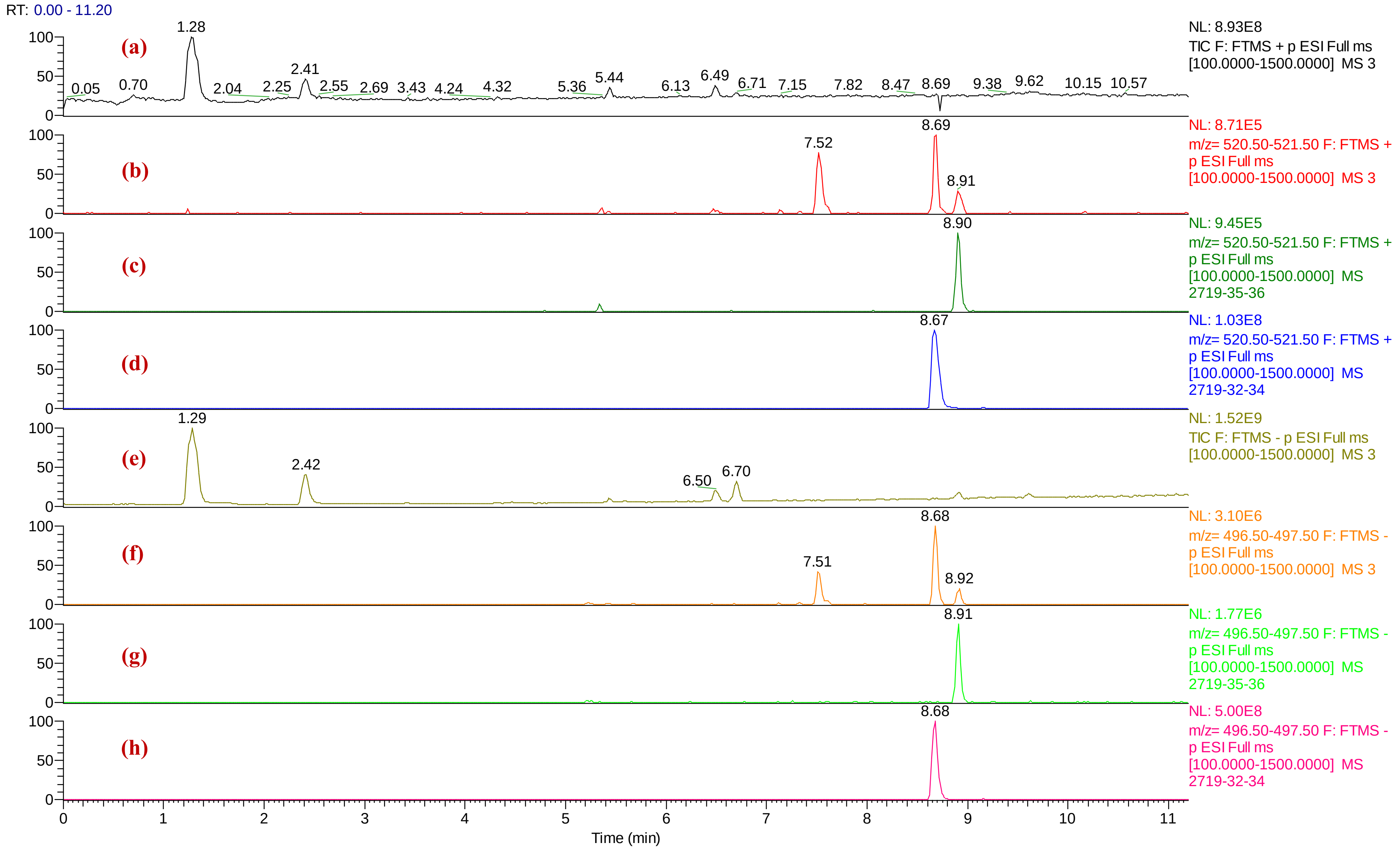

Fig. S99 Overlaid (a) (+)-TIC of the reaction mixture of gastrodin and 4-hydroxybenzyl alcohol (molar ratio, 1:1) in H_2O refluxed for 12 h; (b) the chromatogram of the extracted positive ion at *m/z* 521 $[M + Na]^+$ from (a); (c) and (d) (+)-TIC of aqueous solutions of compounds **7** and **11**; (e) (–)-TIC of the reaction mixture of gastrodin and 4-hydroxybenzyl alcohol (molar ratio, 1:1) in H_2O refluxed for 12 h; (f) the chromatogram of the extracted negative ion at *m/z* 497 $[M – H]^-$ from (e); (g) and (h) (–)-TIC of aqueous solutions of compounds **7** and **11**

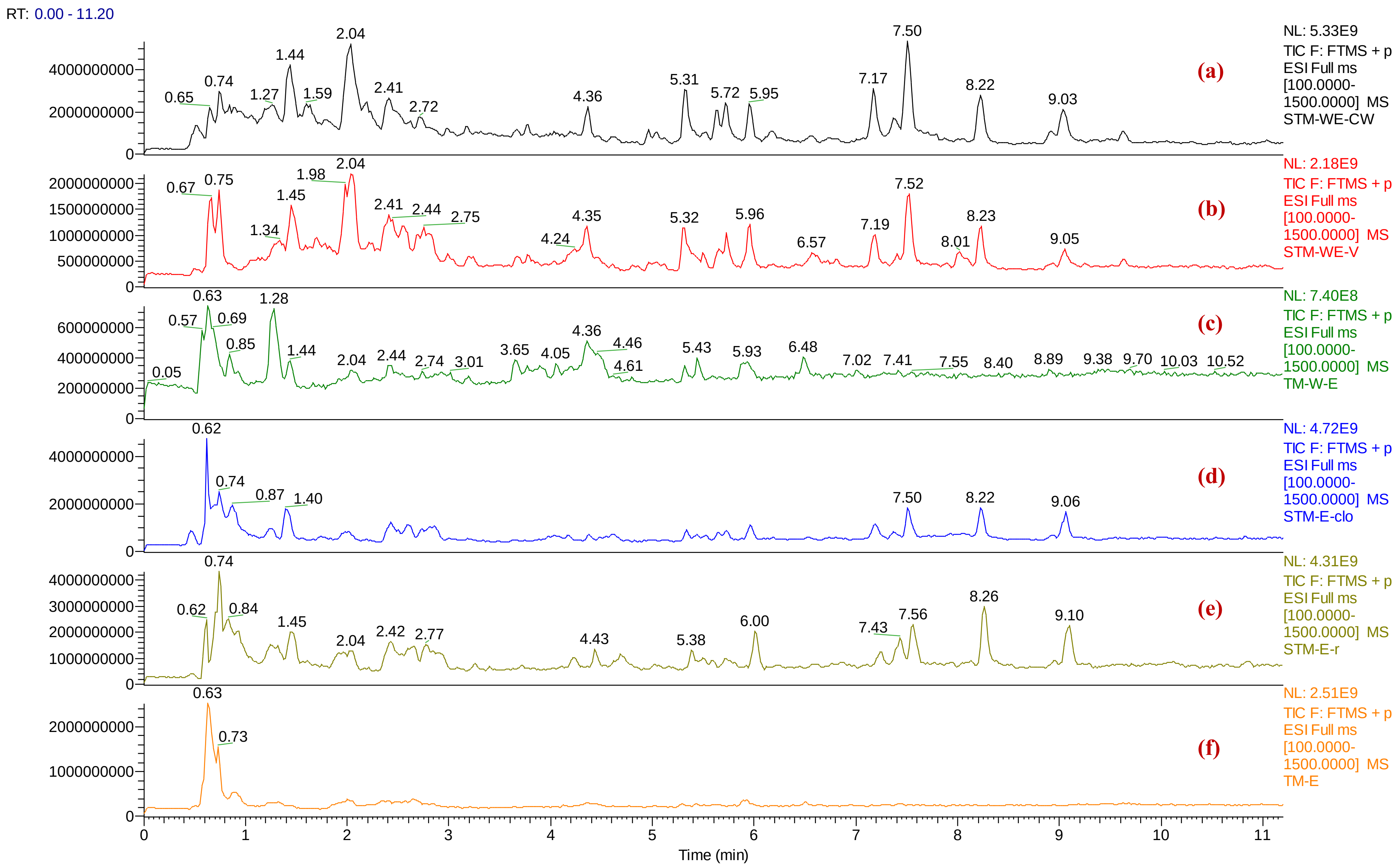

Fig. S100 The overlaid UPLC-HRESIMS (+)-TIC of: (a) an aqueous extract prepared by soaking of the freeze-dried sample of the freshly collected *G. elata* rhizomes at room temperature for 24 h; (b) an aqueous extract prepared by refluxing of the freeze-dried sample of the freshly collected *G. elata* rhizomes for 1 h; (c) an aqueous extract prepared by refluxing of the commercially available "tian ma" sample for 1 h; (d) an ethanol extract prepared by soaking of the freeze-dried sample of the freshly collected *G. elata* rhizomes at room temperature for 24 h; (e) an ethanol extract prepared by refluxing of the freeze-dried sample of the freshly collected *G. elata* rhizomes at room temperature for 1 h; (f) ethanol extract by refluxing of the commercially available "tian ma" sample for 1 h

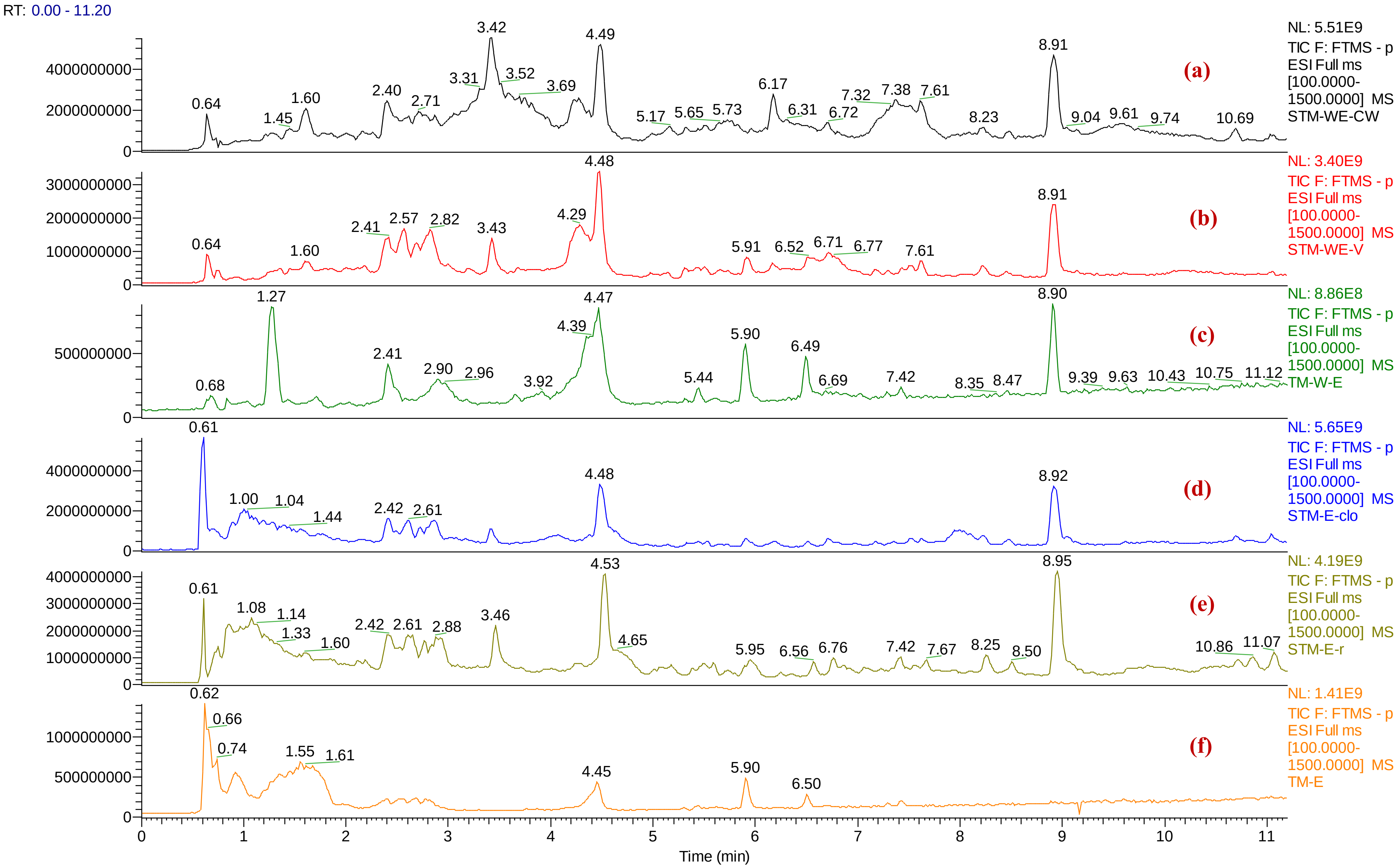

Fig. S101 The overlaid UPLC-HRESIMS (–)-TIC of: (a) an aqueous extract prepared by soaking of the freeze-dried sample of the freshly collected *G. elata* rhizomes at room temperature for 24 h; (b) an aqueous extract prepared by refluxing of the freeze-dried sample of the freshly collected *G. elata* rhizomes for 1 h; (c) an aqueous extract prepared by refluxing of the commercially available “tian ma” sample for 1 h; (d) an ethanol extract prepared by soaking of the freeze-dried sample of the freshly collected *G. elata* rhizomes at room temperature for 24 h; (e) an ethanol extract prepared by refluxing of the freeze-dried sample of the freshly collected *G. elata* rhizomes at room temperature for 1 h; (f) ethanol extract by refluxing of the commercially available “tian ma” sample for 1 h

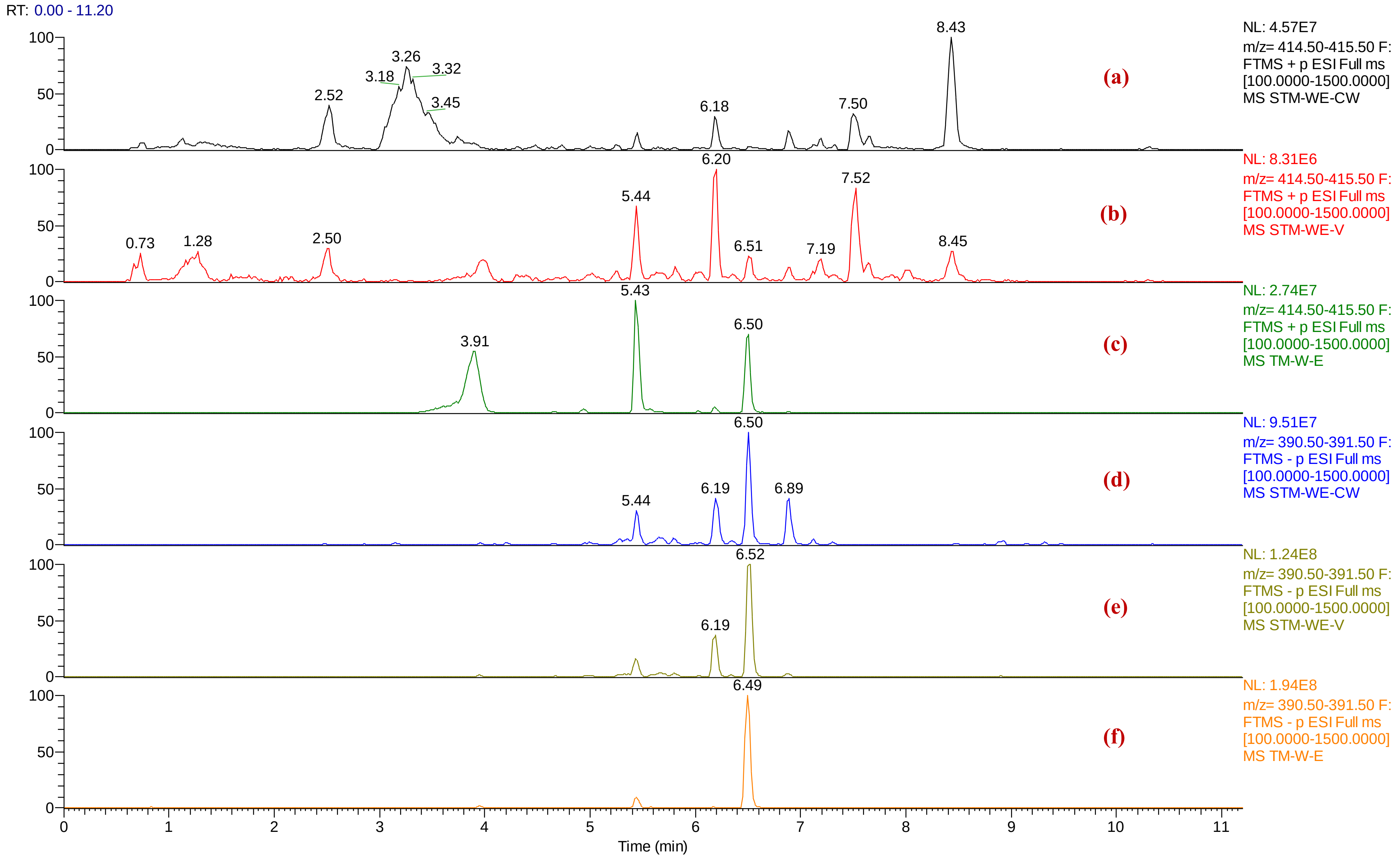

Fig. S102 Overlaid UPLC-HRESIMS chromatograms of the extracted positive ion at *m/z* 415 $[M + Na]^+$ from (+)-TIC of: (a) an aqueous extract prepared by soaking of the freeze-dried sample of the freshly collected *G. elata* rhizomes at room temperature for 24 h; (b) an aqueous extract prepared by refluxing of the freeze-dried sample of the freshly collected *G. elata* rhizomes for 1 h; (c) an aqueous extract prepared by refluxing of the commercially available "tian ma" sample for 1 h; and (d)–(f) for the extracted negative ion at *m/z* 391 $[M - H]^-$ from (–)-TIC of (a)–(c), respectively

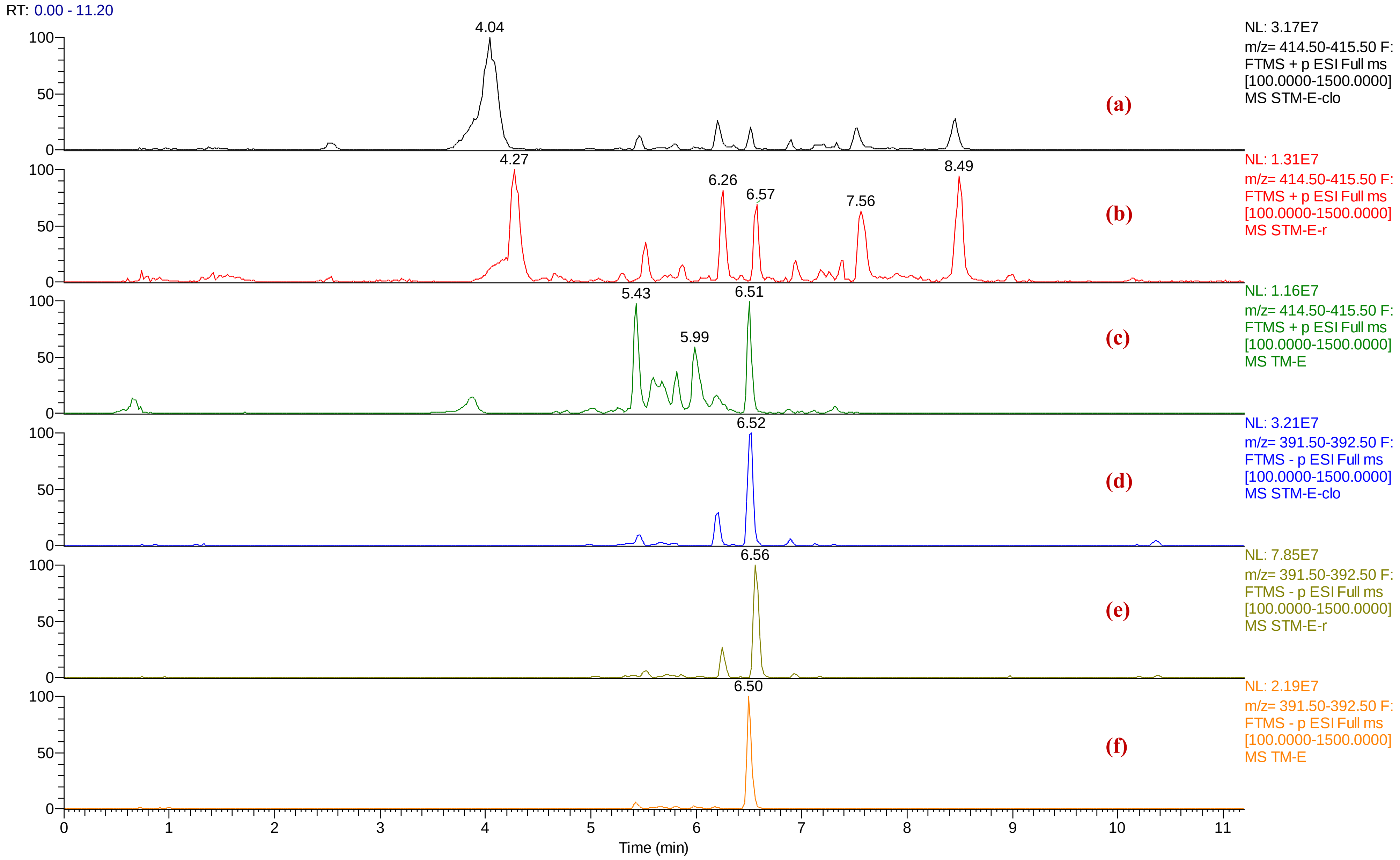

Fig. S103 Overlaid UPLC-HRESIMS chromatograms of the extracted positive ion at *m/z* 415 $[M + Na]^+$ from (+)-TIC of: (a) an ethanol extract prepared by soaking of the freeze-dried sample of the freshly collected *G. elata* rhizomes at room temperature for 24 h; (b) an ethanol extract prepared by refluxing of the freeze-dried sample of the freshly collected *G. elata* rhizomes for 1 h; (c) an ethanol extract prepared by refluxing of the commercially available “tian ma” sample for 1 h; and (d)–(f) for the extracted negative ion at *m/z* 391 $[M - H]^-$ from (–)-TIC of (a)–(c), respectively

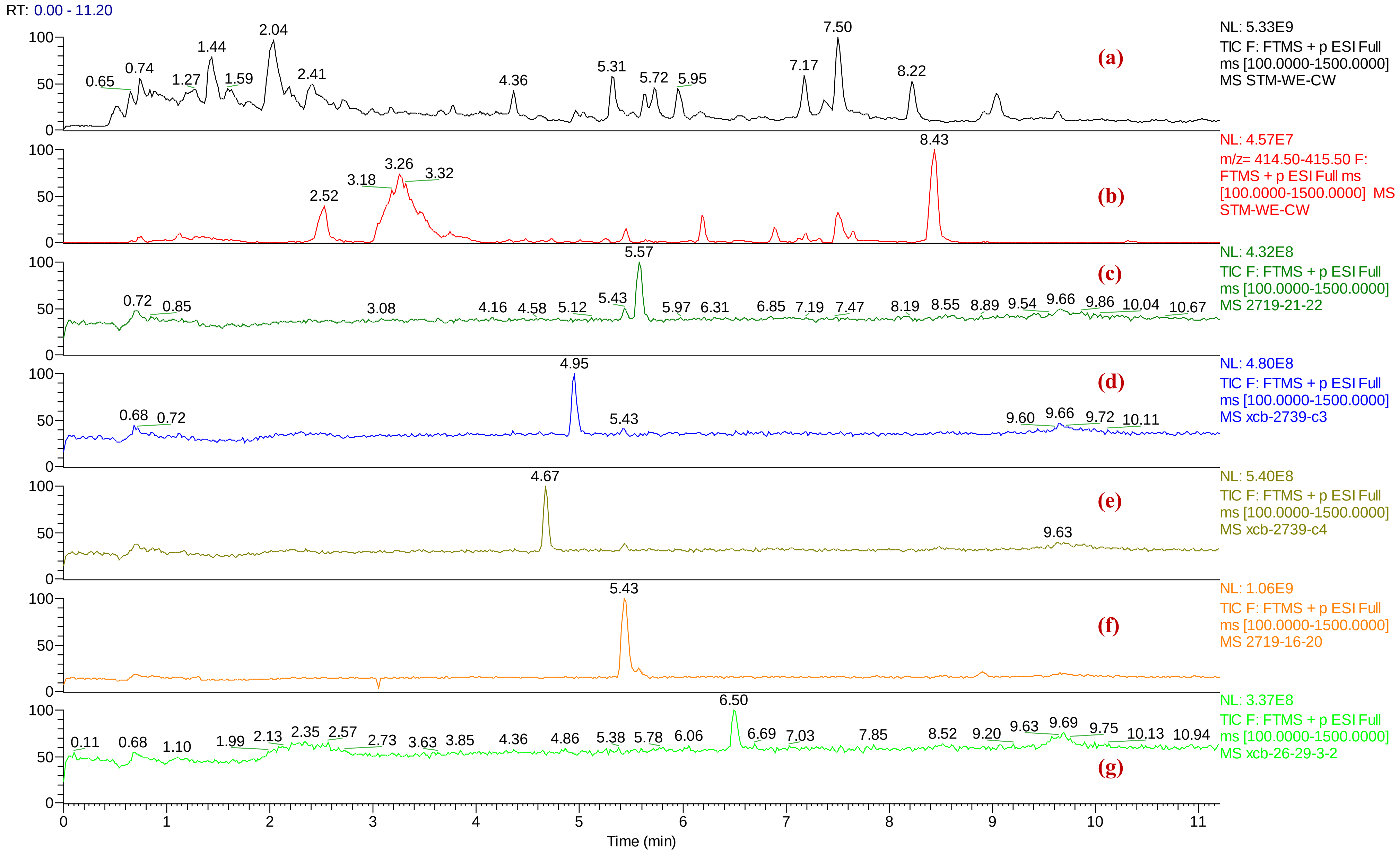

Fig. S104 Overlaid (a) (+)-TIC of an aqueous extract prepared by soaking of the freeze-dried sample of the freshly collected *G. elata* rhizomes at room temperature for 24 h; (b) the chromatogram of the extracted positive ion at *m/z* 415 $[M + Na]^+$ from (a); (c)–(g) (+)-TIC of aqueous solutions of compounds **1–4** and **10**, respectively

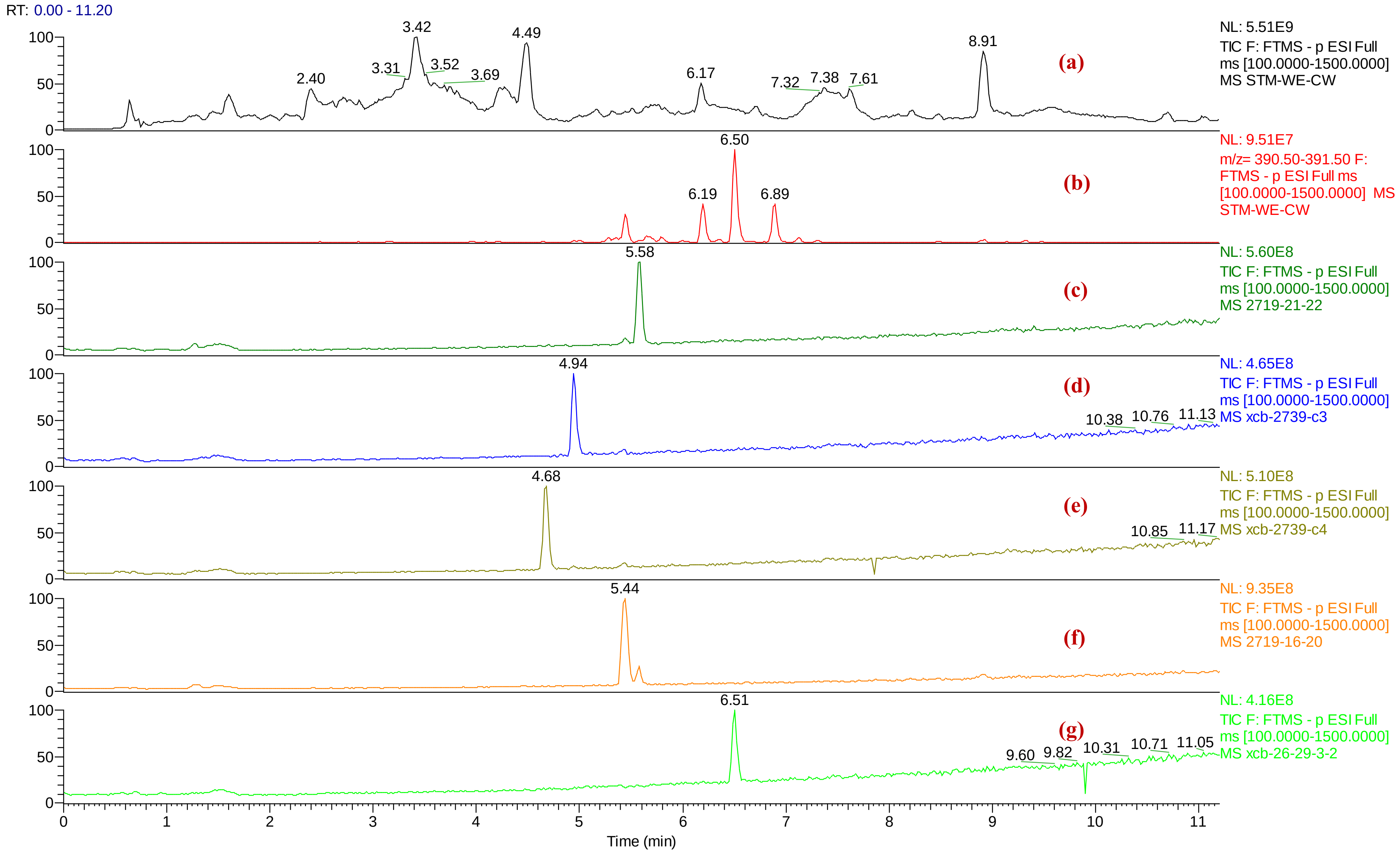

Fig. S105 Overlaid (a) (–)-TIC of an aqueous extract prepared by soaking of the freeze-dried sample of the freshly collected *G. elata* rhizomes at room temperature for 24 h; (b) the chromatogram of the extracted negative ion at *m/z* 391 $[M - H]^-$ from (a); (c)–(g) (–)-TIC of aqueous solutions of compounds **1**–**4** and **10**, respectively

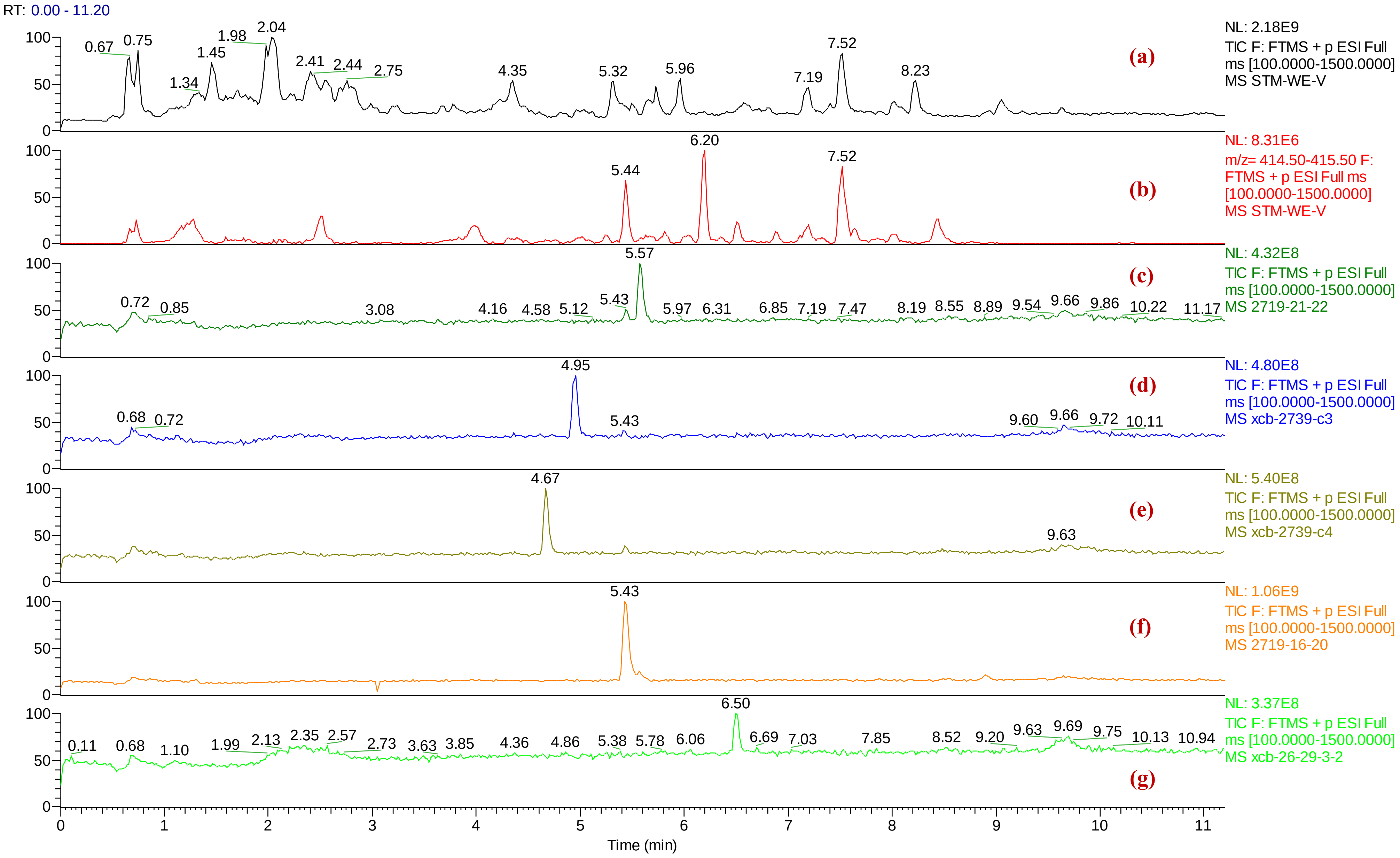

Fig. S106 Overlaid (a) (+)-TIC of an aqueous extract prepared by refluxing of the freeze-dried sample of the freshly collected *G. elata* rhizomes for 1 h; (b) the chromatogram of the extracted positive ion at *m/z* 415 $[M + Na]^+$ from (a); (c)–(g) (+)-TIC of aqueous solutions of compounds **1–4** and **10**, respectively

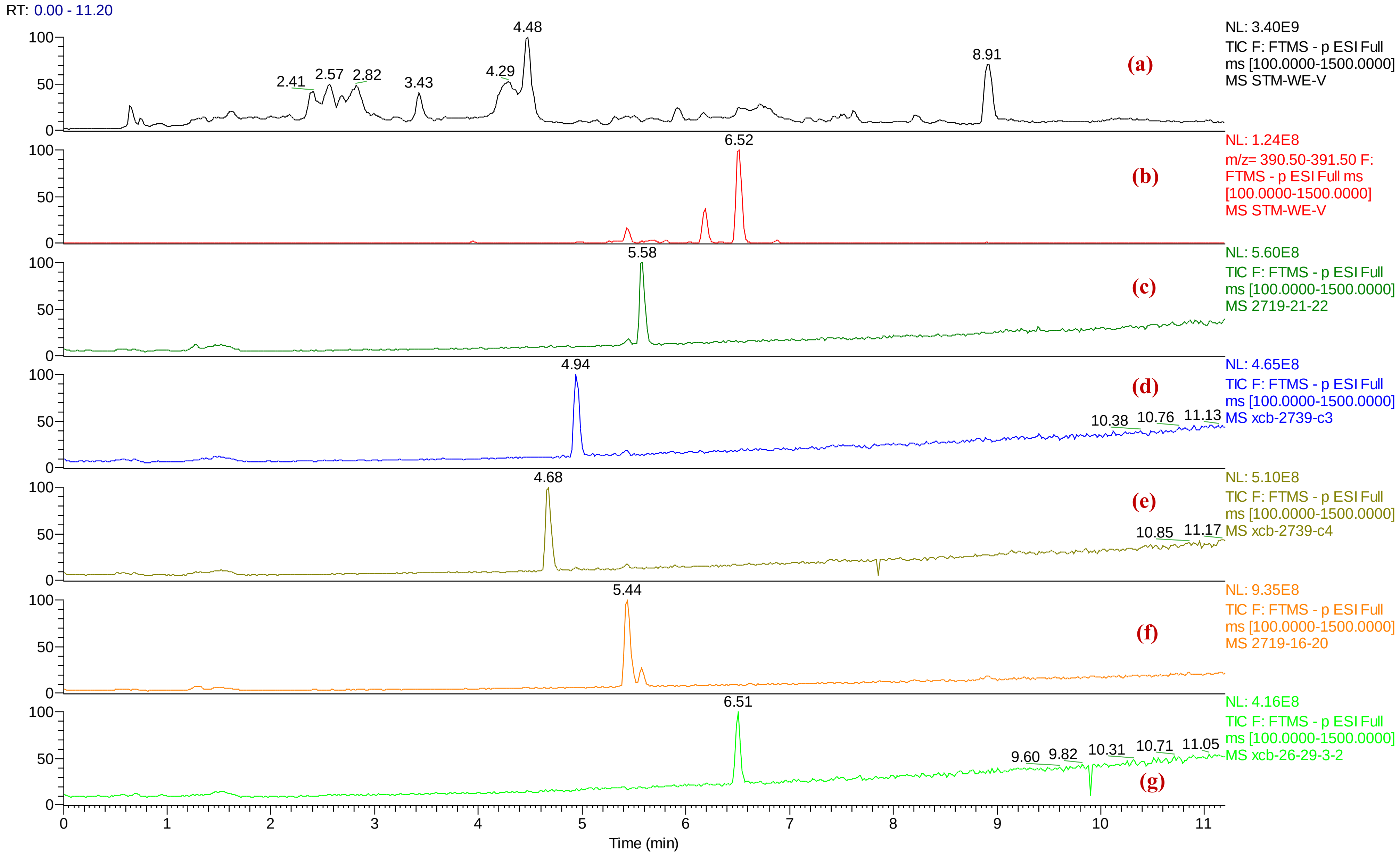

Fig. S107 Overlaid (a) (–)-TIC of an aqueous extract prepared by refluxing of the freeze-dried sample of the freshly collected *G. elata* rhizomes for 1 h; (b) the chromatogram of the extracted negative ion at *m/z* 391 $[M-H]^-$ from (a); (c)–(g) (–)-TIC of aqueous solutions of compounds **1–4** and **10**, respectively

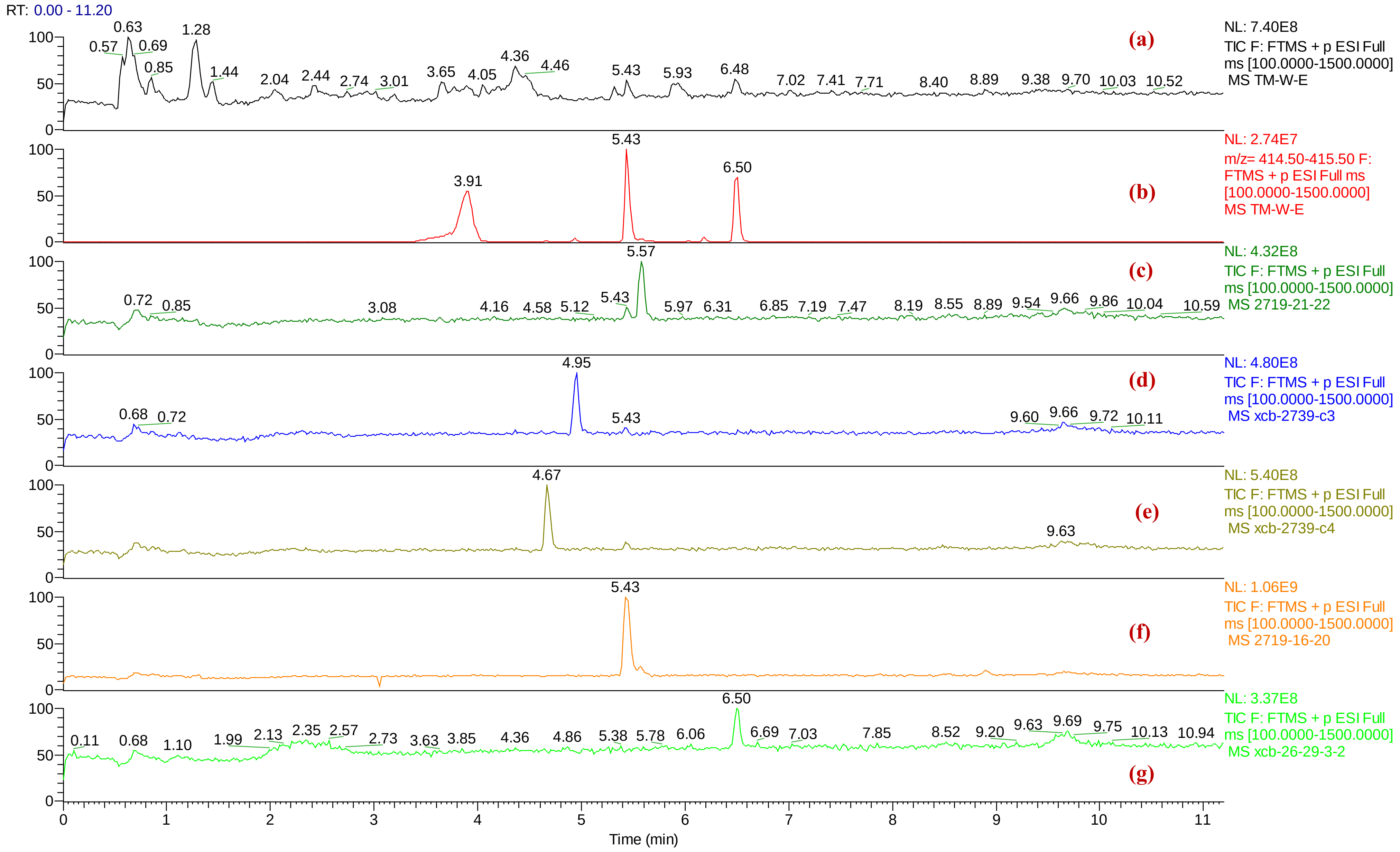

Fig. S108 Overlaid (a) (+)-TIC of an aqueous extract prepared by refluxing of the commercially available "tian ma" sample for 1 h; (b) the chromatogram of the extracted positive ion at *m/z* 415 $[M + Na]^+$ from (a); (c)–(g) (+)-TIC of aqueous solutions of compounds **1–4** and **10**, respectively

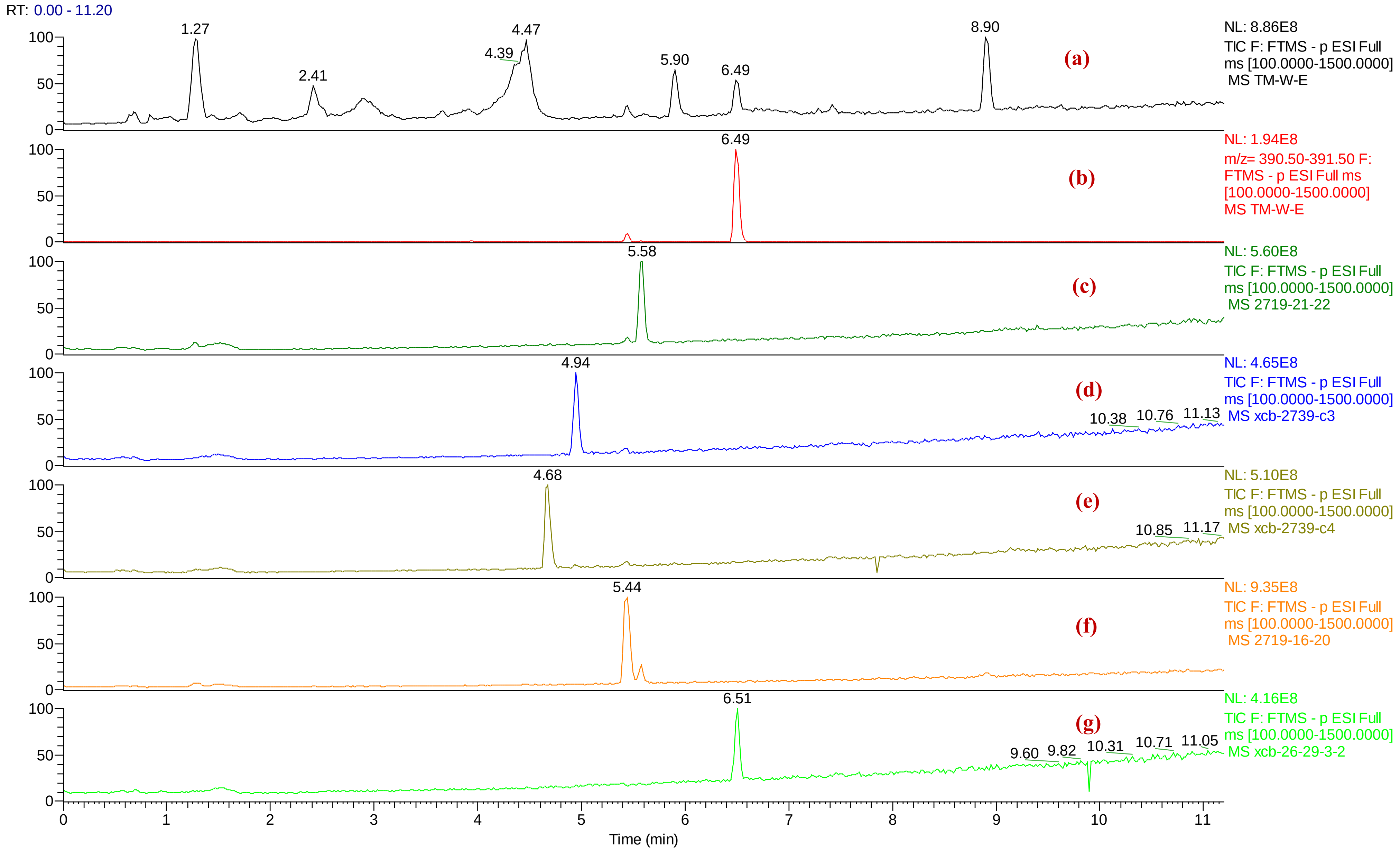

Fig. S109 Overlaid (a) (–)-TIC of an aqueous extract prepared by refluxing of the commercially available "tian ma" sample for 1 h; (b) the chromatogram of the extracted negative ion at *m/z* 391 $[M - H]^-$ from (a); (c)–(g) (–)-TIC of aqueous solutions of compounds **1–4** and **10**, respectively

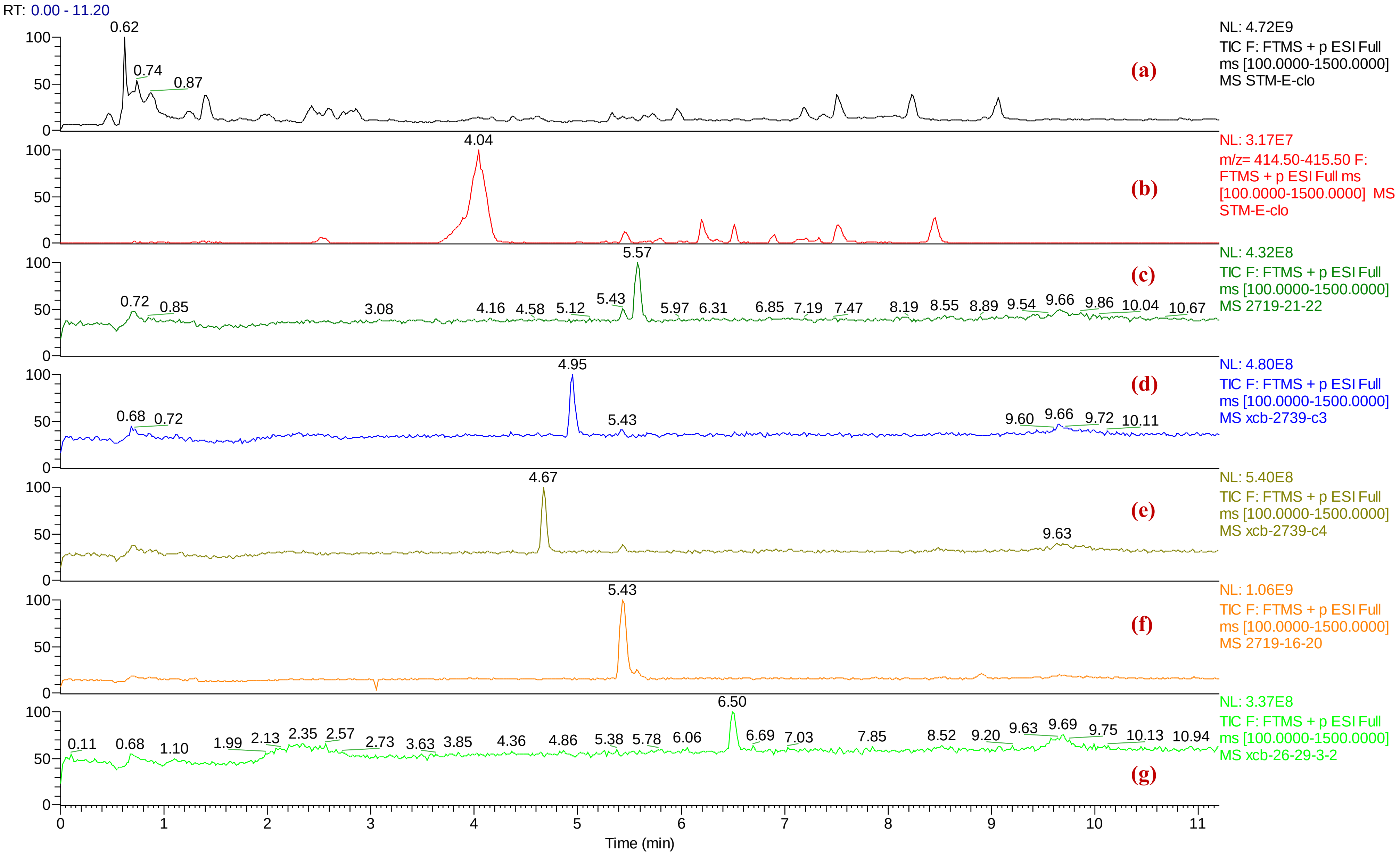

Fig. S110 Overlaid (a) (+)-TIC of an ethanol extract prepared by soaking of the freeze-dried sample of the freshly collected *G. elata* rhizomes at room temperature for 24 h; (b) the chromatogram of the extracted positive ion at *m/z* 415 $[M + Na]^+$ from (a); (c)–(g) (+)-TIC of aqueous solutions of compounds **1–4** and **10**, respectively

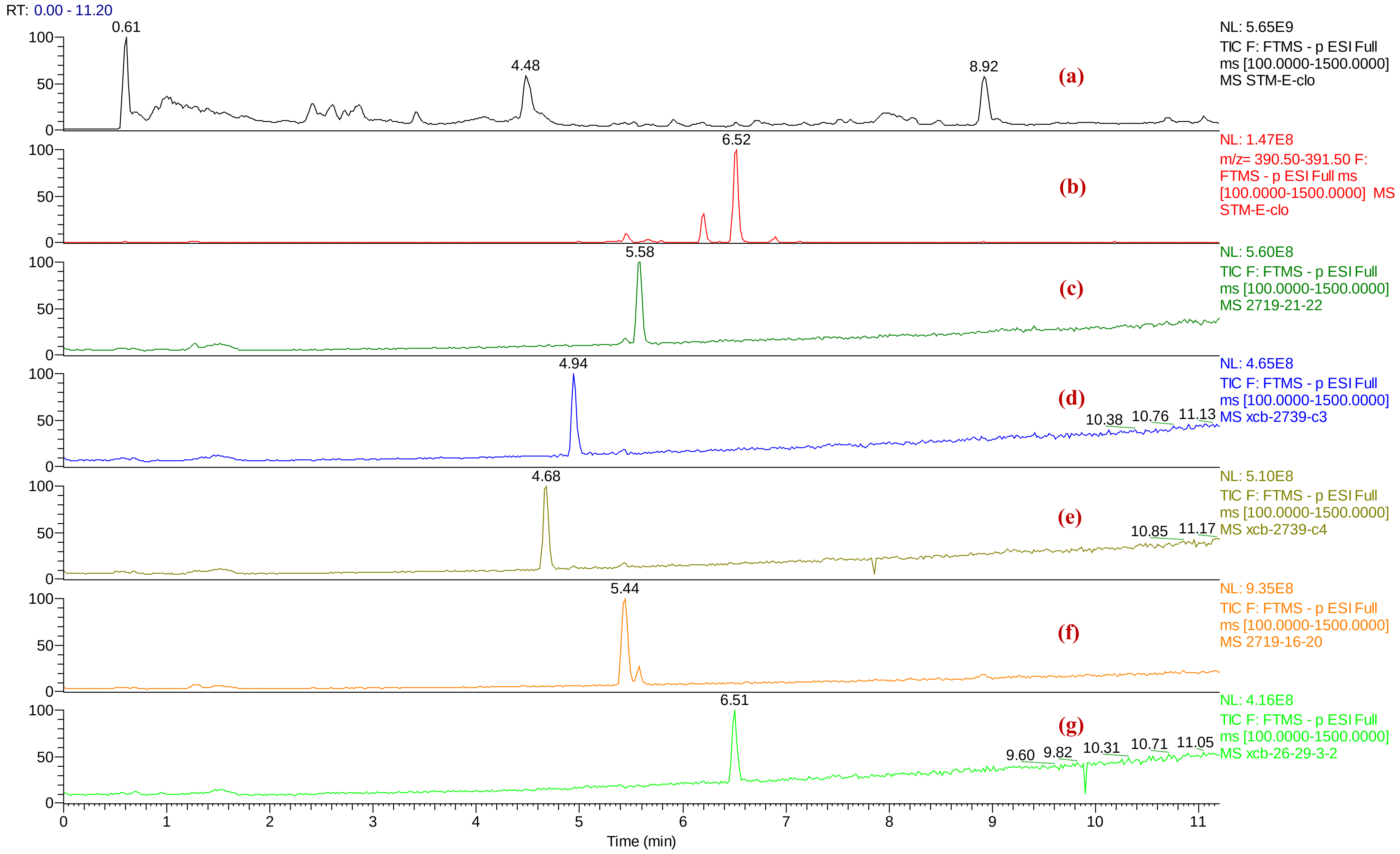

Fig. S111 Overlaid (a) (–)-TIC of an ethanol extract prepared by soaking of the freeze-dried sample of the freshly collected *G. elata* rhizomes at room temperature for 24 h; (b) the chromatogram of the extracted negative ion at *m/z* 391 $[M - H]^-$ from (a); (c)–(g) (–)-TIC of aqueous solutions of compounds **1–4** and **10**, respectively

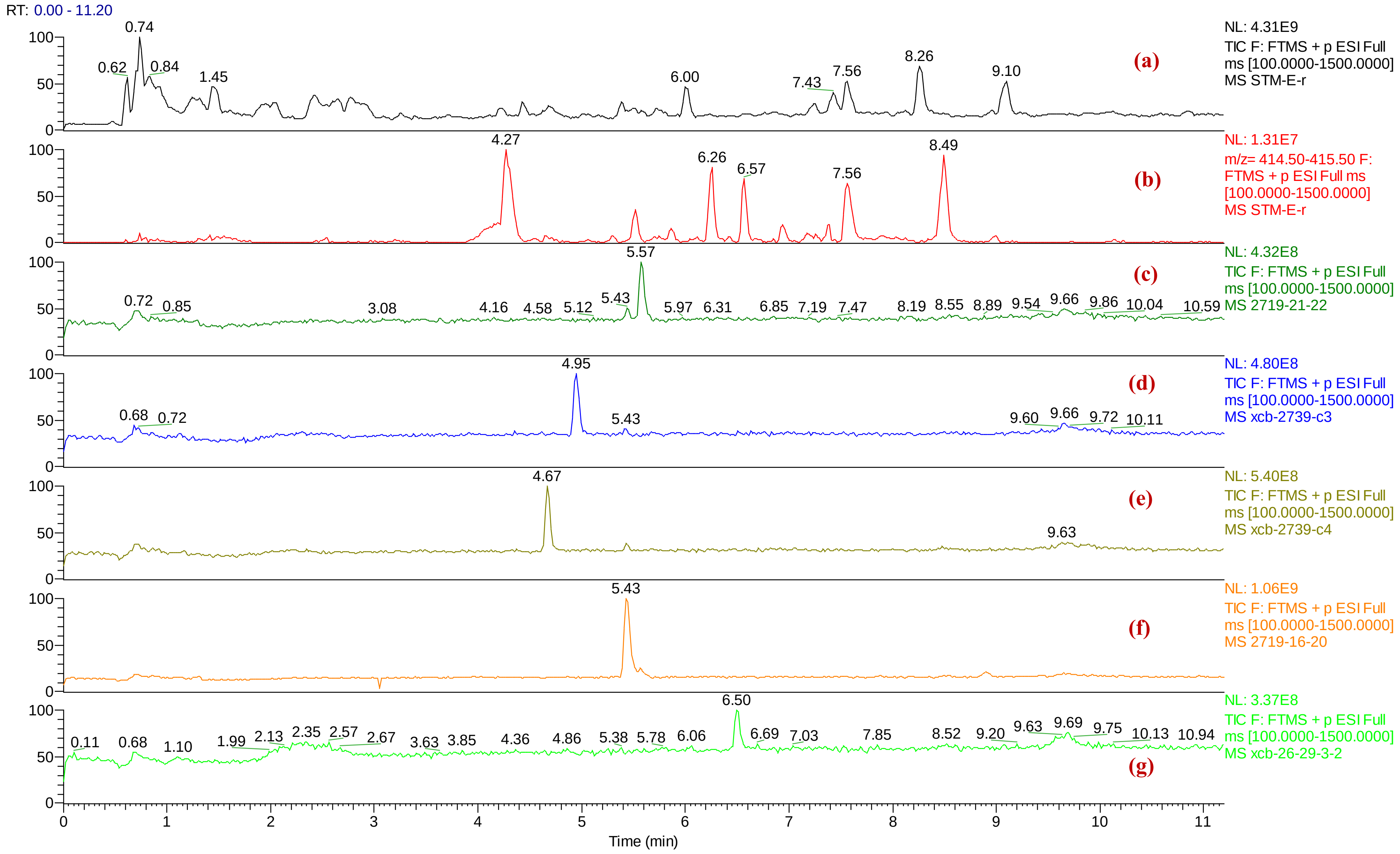

Fig. S112 Overlaid (a) (+)-TIC of an ethanol extract prepared by refluxing of the freeze-dried sample of the freshly collected *G. elata* rhizomes for 1 h; (b) the chromatogram of the extracted positive ion at *m/z* 415 $[M + Na]^+$ from (a); (c)–(g) (+)-TIC of aqueous solutions of compounds **1–4** and **10**, respectively

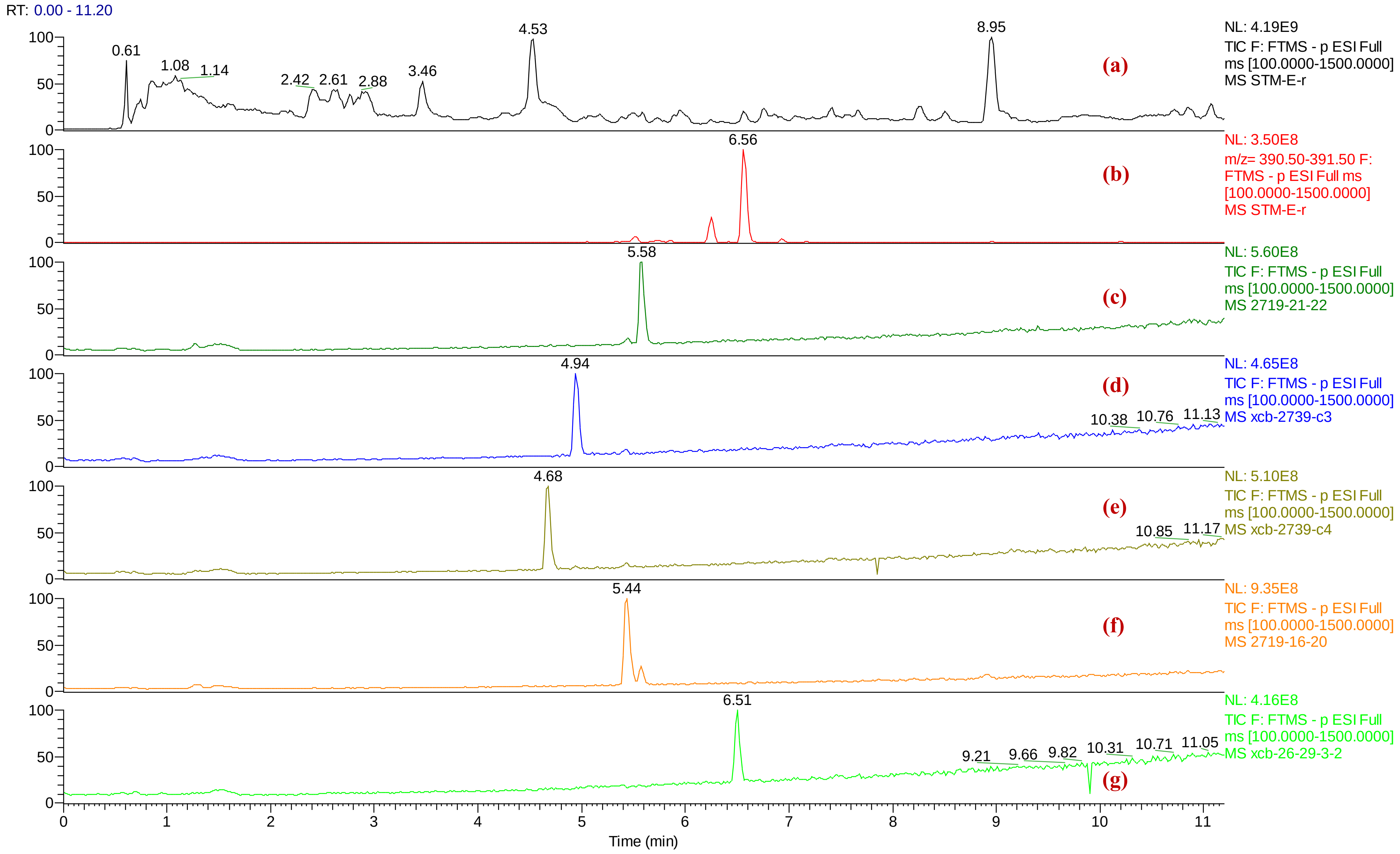

Fig. S113 Overlaid (a) (–)-TIC of an ethanol extract prepared by refluxing of the freeze-dried sample of the freshly collected *G. elata* rhizomes for 1 h; (b) the chromatogram of the extracted negative ion at *m/z* 391 $[M-H]^-$ from (a); (c)–(g) (–)-TIC of aqueous solutions of compounds **1–4** and **10**, respectively

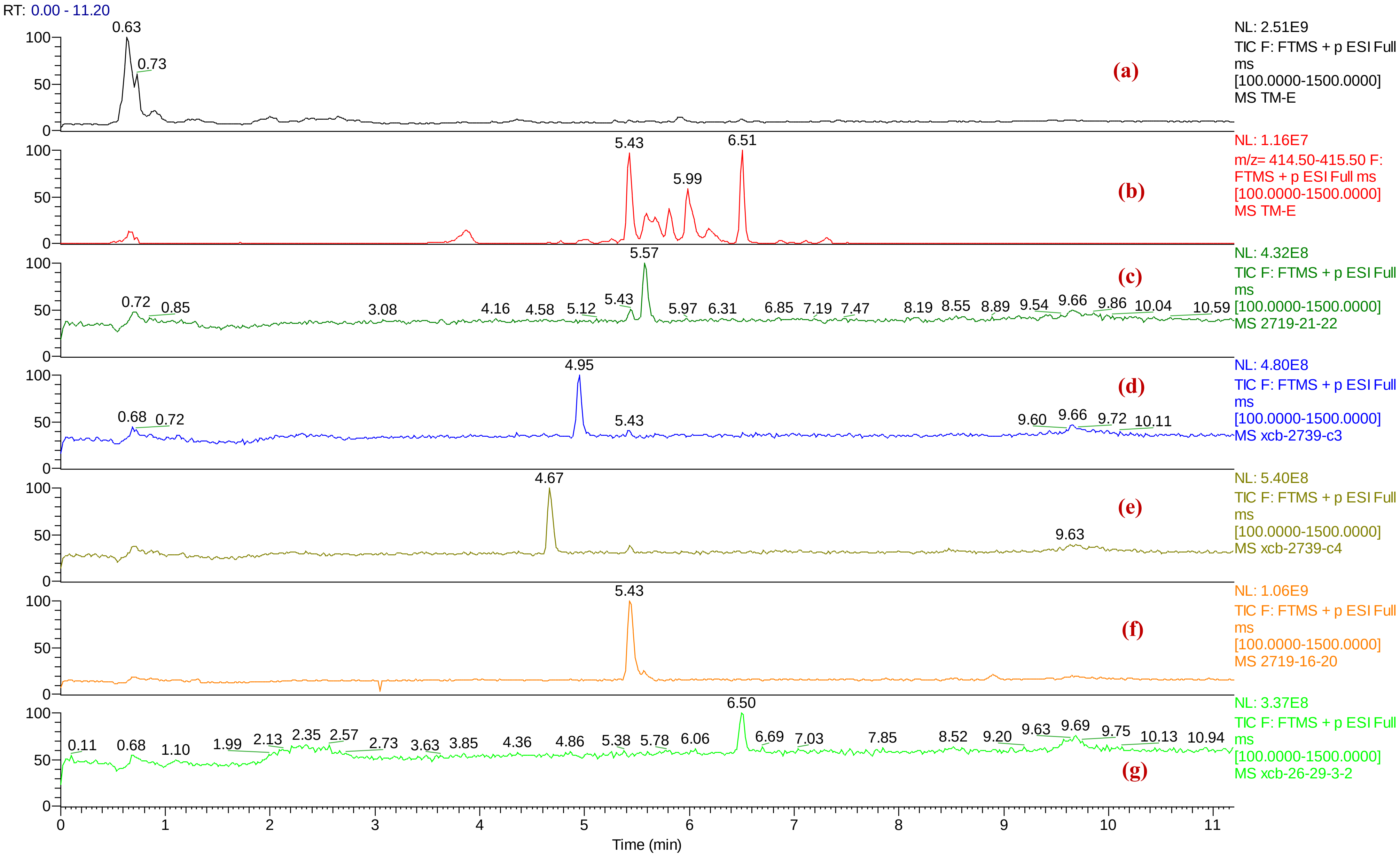

Fig. S114 Overlaid (a) (+)-TIC of an ethanol extract prepared by refluxing of the commercially available "tian ma" sample for 1 h; (b) the chromatogram of the extracted positive ion at *m/z* 415 $[M + Na]^+$ from (a); (c)–(g) (+)-TIC of aqueous solutions of compounds **1–4** and **10**, respectively

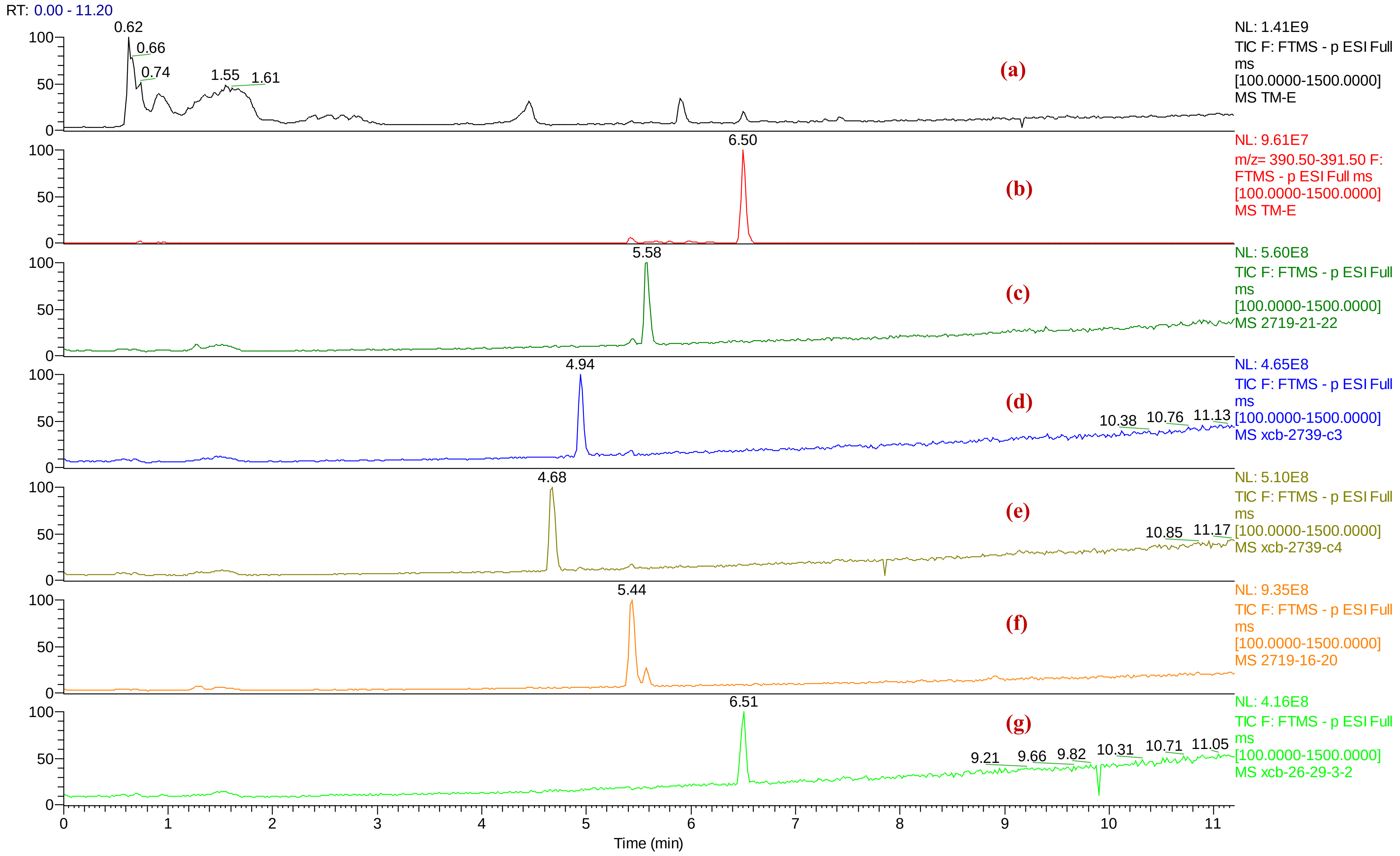

Fig. S115 Overlaid (a) (–)-TIC of an ethanol extract prepared by refluxing of the commercially available "tian ma" sample for 1 h; (b) the chromatogram of the extracted negative ion at *m/z* 391 $[M-H]^-$ from (a); (c)–(g) (–)-TIC of aqueous solutions of compounds **1**–**4** and **10**, respectively

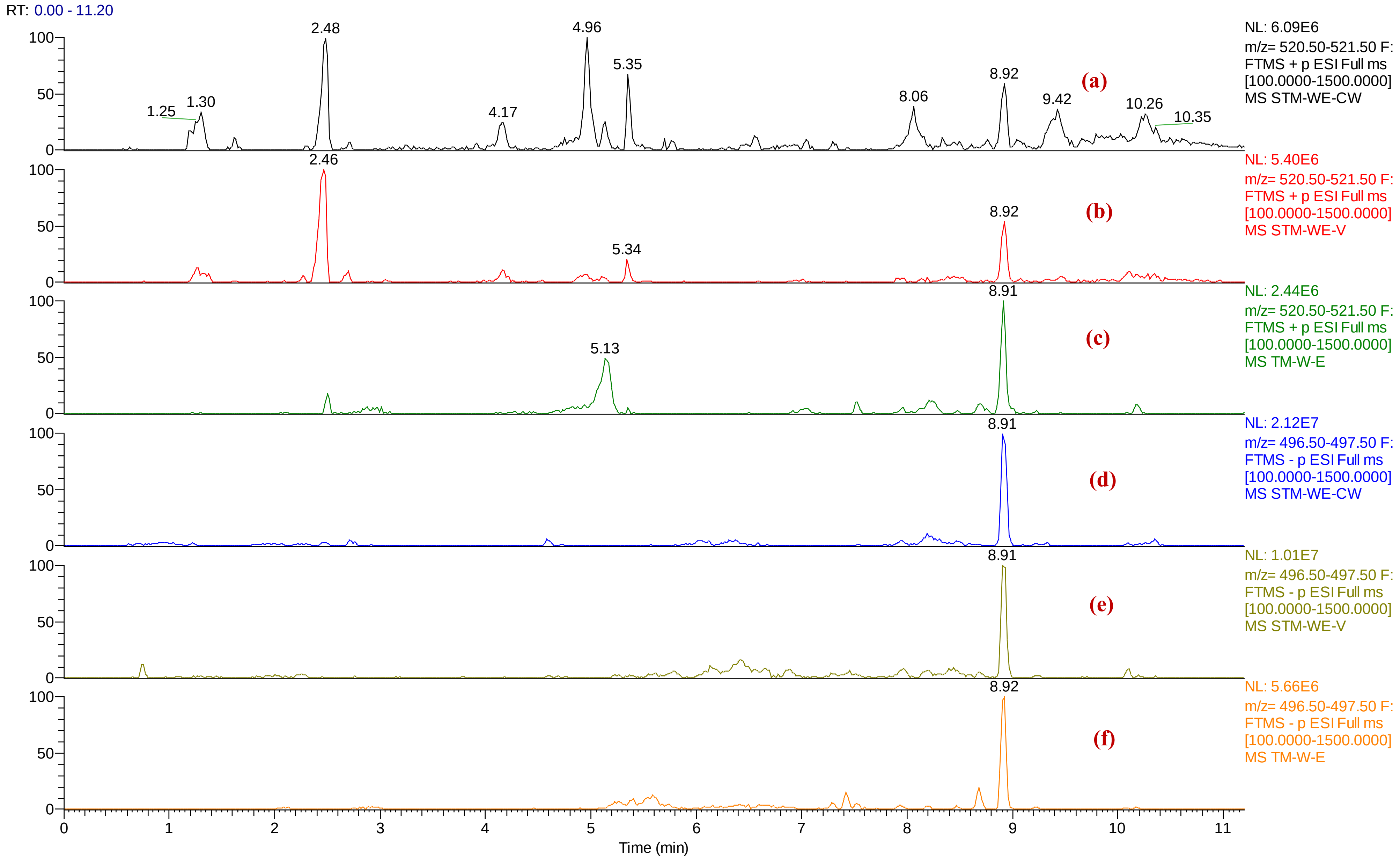

Fig. S116 Overlaid UPLC-HRESIMS chromatograms of the extracted positive ion at *m/z* 521 $[M + Na]^+$ from (+)-TIC of: (a) an aqueous extract prepared by soaking of the freeze-dried sample of the freshly collected *G. elata* rhizomes at room temperature for 24 h; (b) an aqueous extract prepared by refluxing of the freeze-dried sample of the freshly collected *G. elata* rhizomes for 1 h; (c) an aqueous extract prepared by refluxing of the commercially available "tian ma" sample for 1 h; and (d)–(f) for the extracted negative ion at *m/z* 497 $[M - H]^-$ from (–)-TIC of (a)–(c), respectively

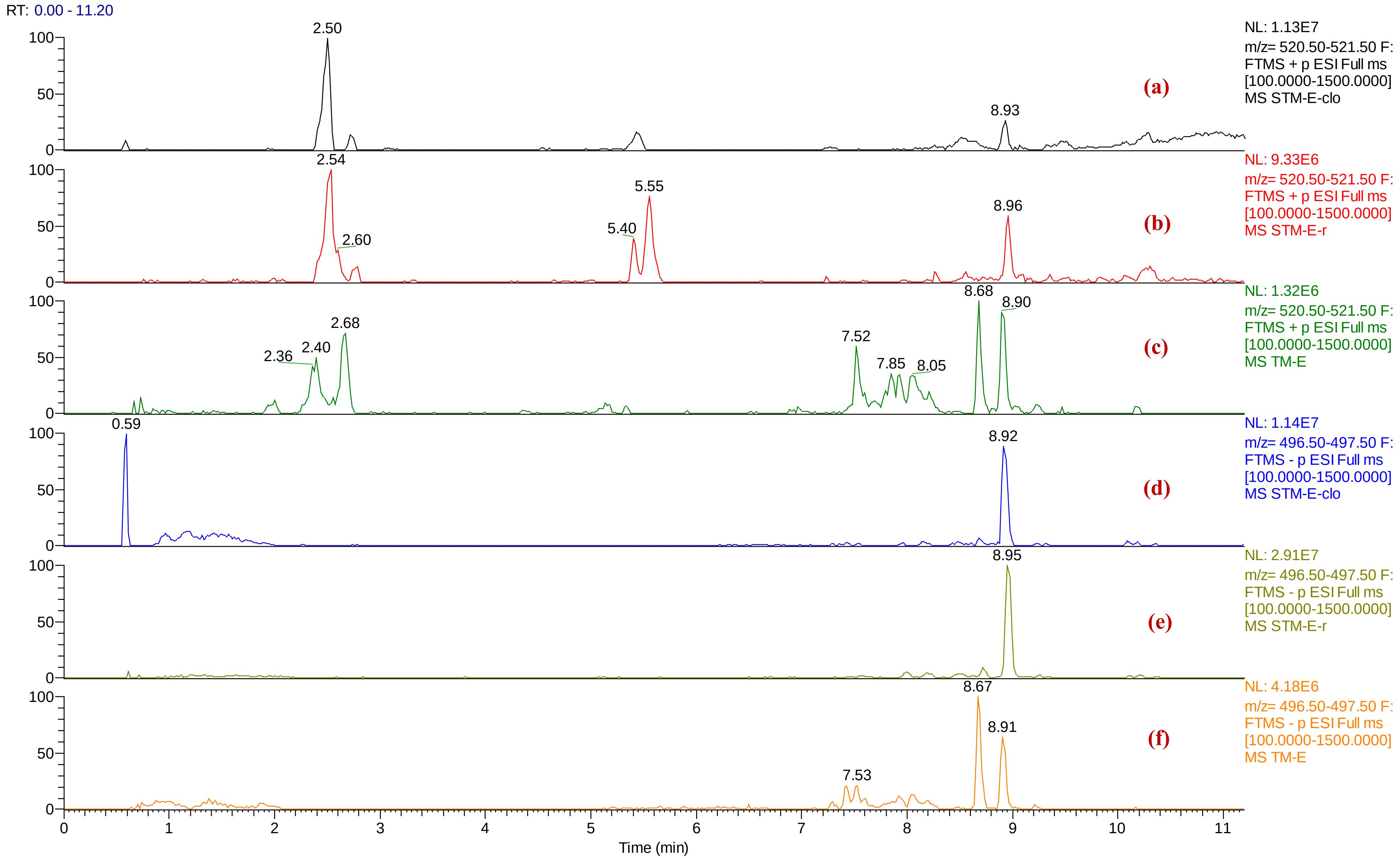

Fig. S117 Overlaid UPLC-HRESIMS chromatograms of the extracted positive ion at *m/z* 521 $[M + Na]^+$ from (+)-TIC of: (a) an ethanol extract prepared by soaking of the freeze-dried sample of the freshly collected *G. elata* rhizomes at room temperature for 24 h; (b) an ethanol extract prepared by refluxing of the freeze-dried sample of the freshly collected *G. elata* rhizomes for 1 h; (c) an ethanol extract prepared by refluxing of the commercially available "tian ma" sample for 1 h; and (d)–(f) for the extracted negative ion at *m/z* 497 $[M - H]^-$ from (–)-TIC of (a)–(c), respectively

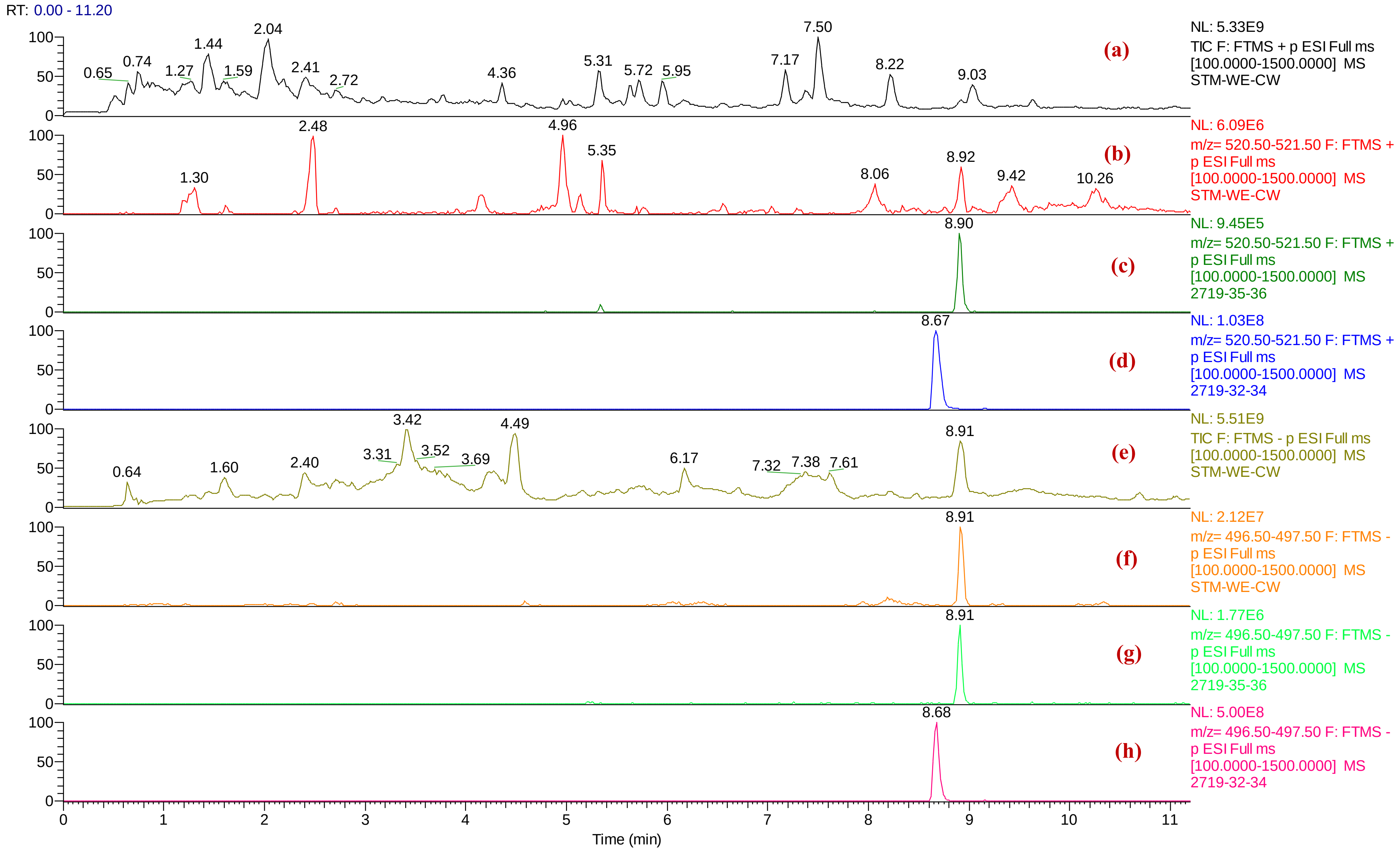

Fig. S118 Overlaid (a) (+)-TIC of an aqueous extract prepared by soaking of the freeze-dried sample of the freshly collected *G. elata* rhizomes at room temperature for 24 h; (b) the chromatogram of the extracted positive ion at *m/z* 521 $[M + Na]^+$ from (a); (c) and (d) (+)-TIC of aqueous solutions of compounds **7** and **10**; (e) (–)-TIC of an aqueous extract prepared by soaking of the freeze-dried sample of the freshly collected *G. elata* rhizomes at room temperature for 24 h; (f) the chromatogram of the extracted negative ion at *m/z* 497 $[M - H]^-$ from (e); (g) and (h) (–)-TIC of aqueous solutions of compounds **7** and **10**

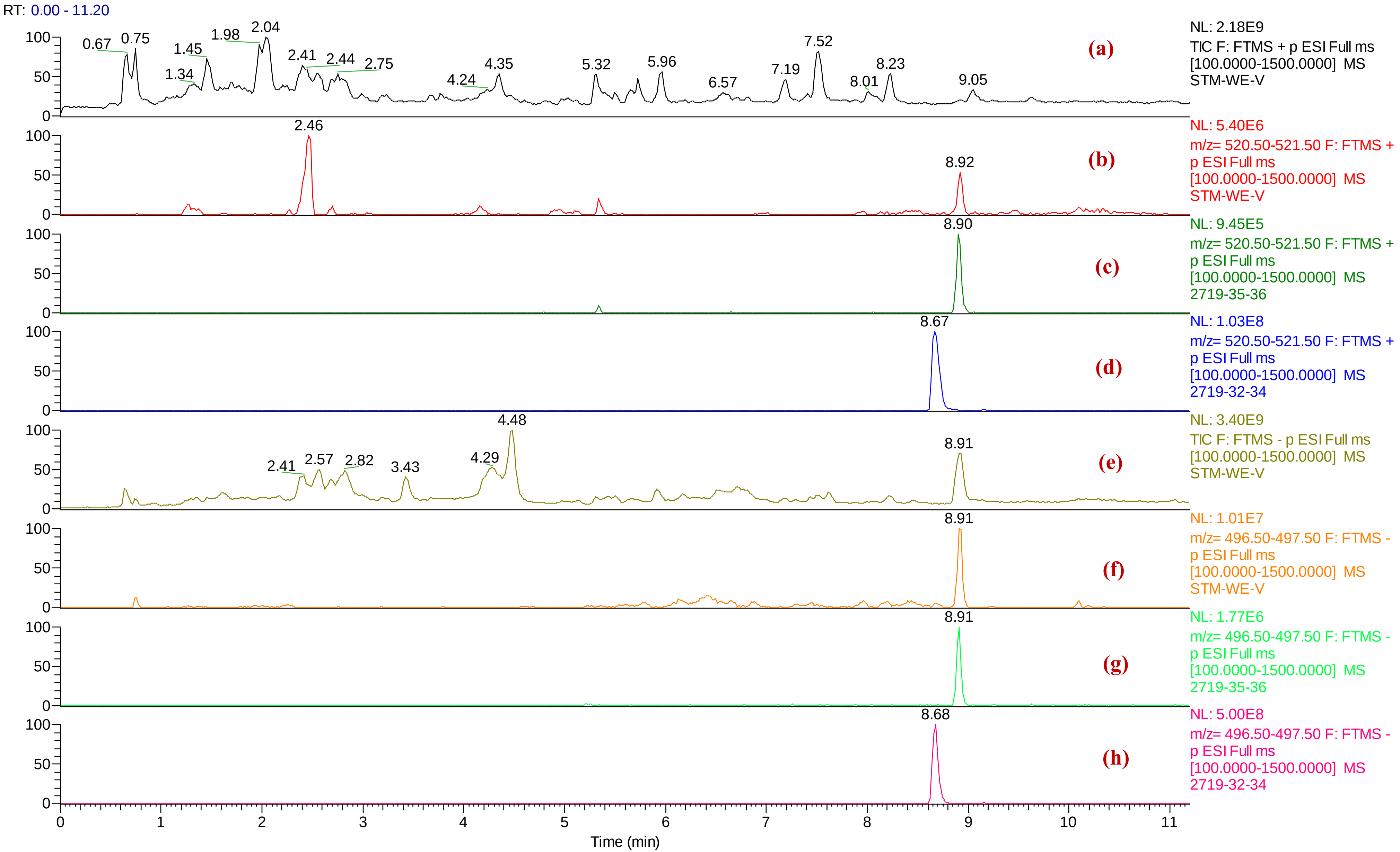

Fig. S119 Overlaid (a) (+)-TIC of an aqueous extract prepared by refluxing of the freeze-dried sample of the freshly collected *G. elata* rhizomes for 1 h; (b) the chromatogram of the extracted positive ion at *m/z* 521 $[M + Na]^+$ from (a); (c) and (d) (+)-TIC of aqueous solutions of compounds **7** and **10**; (e) (−)-TIC of an aqueous extract prepared by refluxing of the freeze-dried sample of the freshly collected *G. elata* rhizomes for 1 h; (f) the chromatogram of the extracted negative ion at *m/z* 497 $[M - H]^-$ from (e); (g) and (h) (−)-TIC of aqueous solutions of compounds **7** and **10**

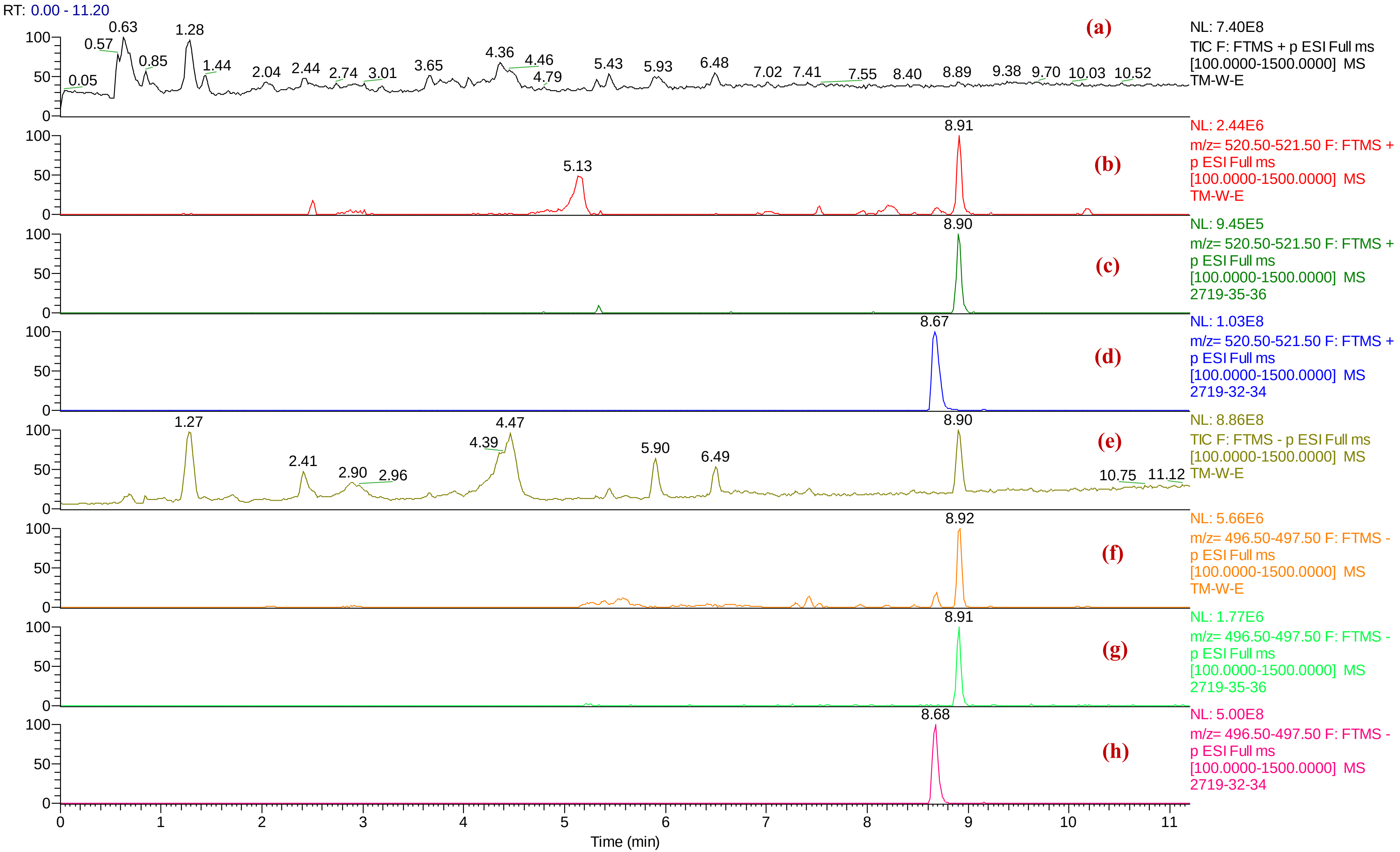

Fig. S120 Overlaid (a) (+)-TIC of an aqueous extract prepared by refluxing of the commercially available "tian ma" sample for 1 h; (b) the chromatogram of the extracted positive ion at *m/z* 521 $[M + Na]^+$ from (a); (c) and (d) (+)-TIC of aqueous solutions of compounds **7** and **10**; (e) (–)-TIC of an aqueous extract prepared by refluxing of the commercially available "tian ma" sample for 1 h; (f) the chromatogram of the extracted negative ion at *m/z* 497 $[M - H]^-$ from (e); (g) and (h) (–)-TIC of aqueous solutions of compounds **7** and **10**

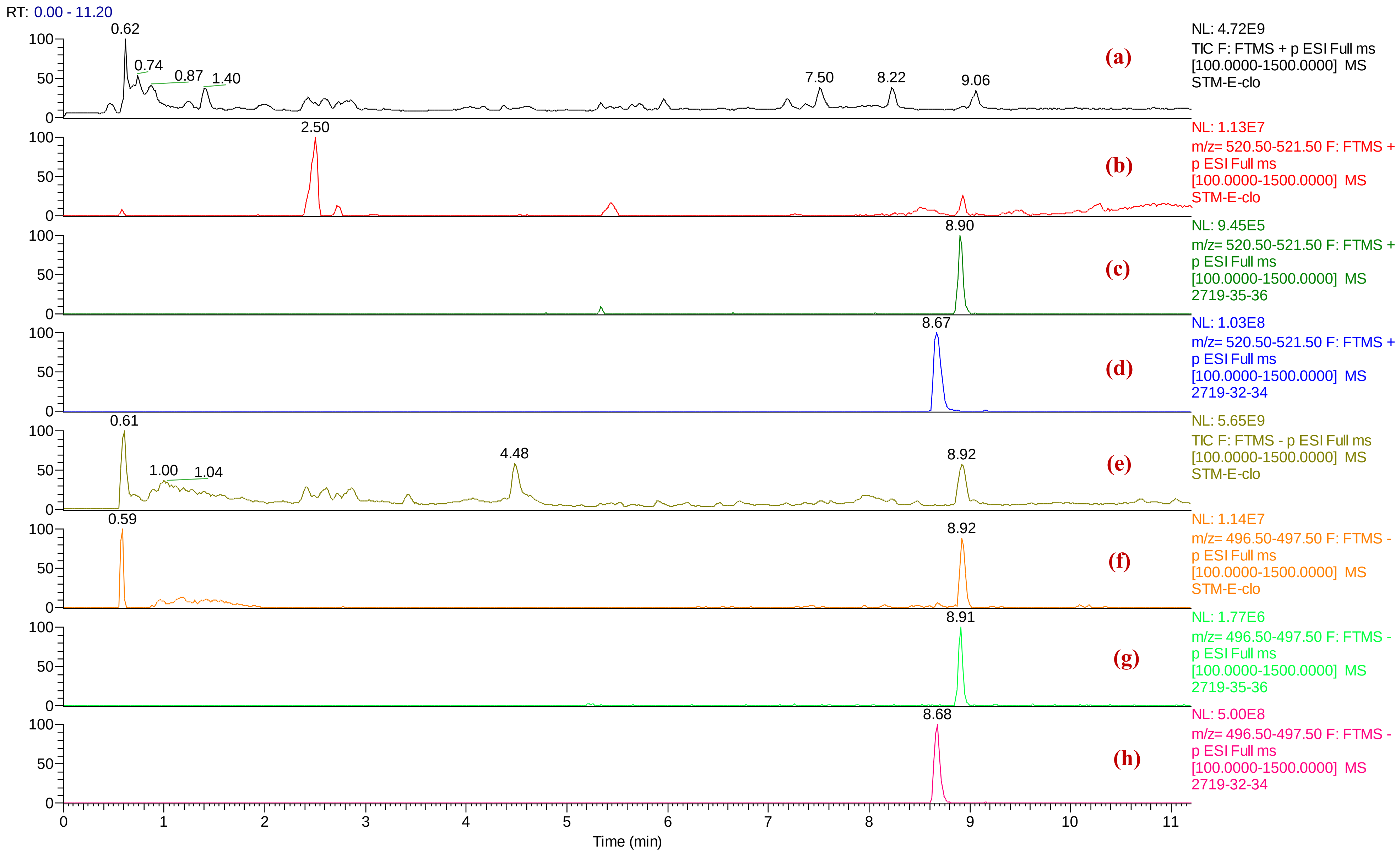

Fig. S121 Overlaid (a) (+)-TIC of an ethanol extract prepared by soaking of the freeze-dried sample of the freshly collected *G. elata* rhizomes at room temperature for 24 h; (b) the chromatogram of the extracted positive ion at *m/z* 521 $[M + Na]^+$ from (a); (c) and (d) (+)-TIC of aqueous solutions of compounds **7** and **10**; (e) (–)-TIC of an ethanol extract prepared by soaking of the freeze-dried sample of the freshly collected *G. elata* rhizomes at room temperature for 24 h; (f) the chromatogram of the extracted negative ion at *m/z* 497 $[M - H]^-$ from (e); (g) and (h) (–)-TIC of aqueous solutions of compounds **7** and **10**

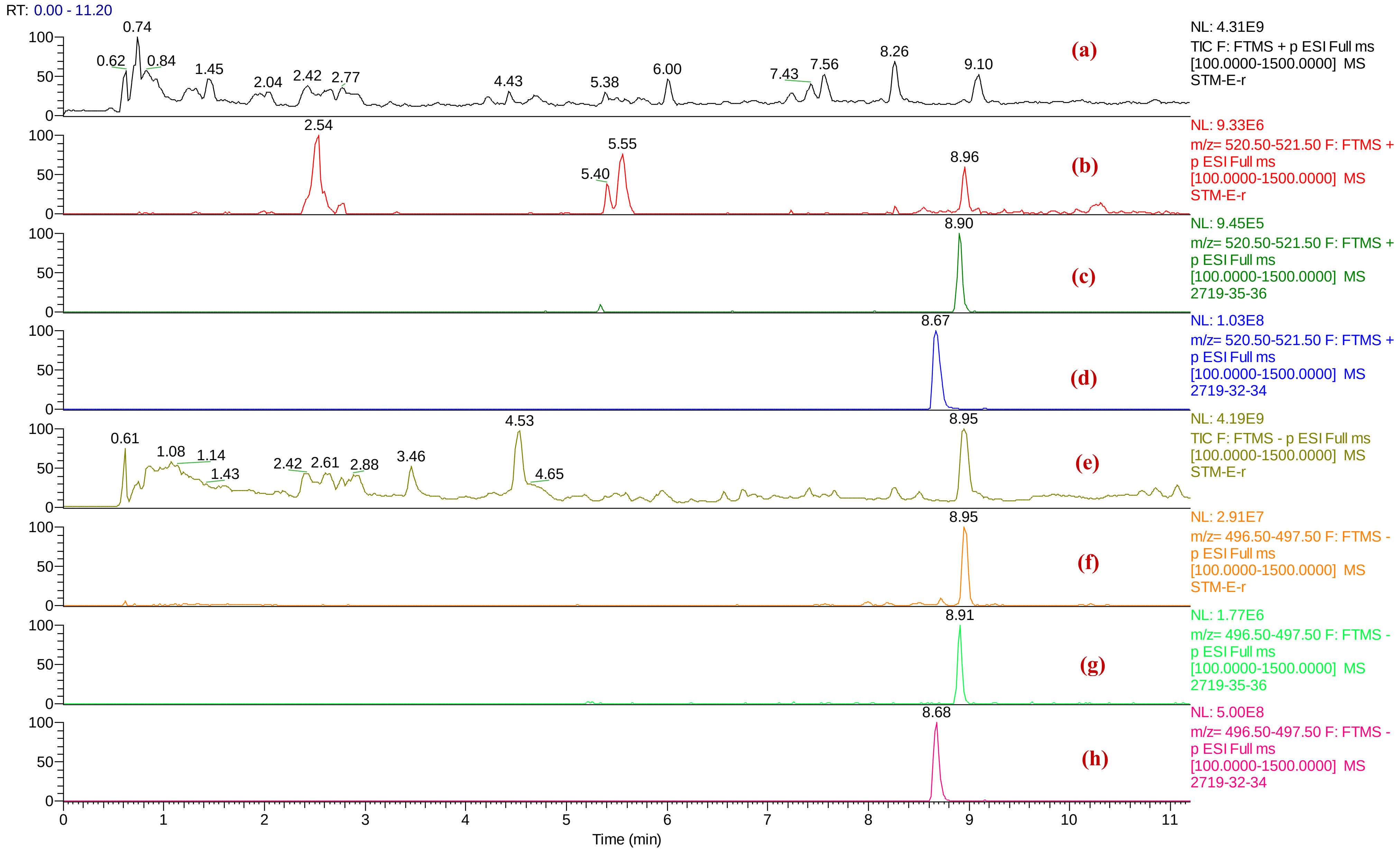

Fig. S122 Overlaid (a) (+)-TIC of an ethanol extract prepared by refluxing of the freeze-dried sample of the freshly collected *G. elata* rhizomes for 1 h; (b) the chromatogram of the extracted positive ion at *m/z* 521 $[M + Na]^+$ from (a); (c) and (d) (+)-TIC of aqueous solutions of compounds **7** and **10**; (e) (−)-TIC of an ethanol extract prepared by refluxing of the freeze-dried sample of the freshly collected *G. elata* rhizomes for 1 h; (f) the chromatogram of the extracted negative ion at *m/z* 497 $[M - H]^-$ from (e); (g) and (h) (−)-TIC of aqueous solutions of compounds **7** and **10**

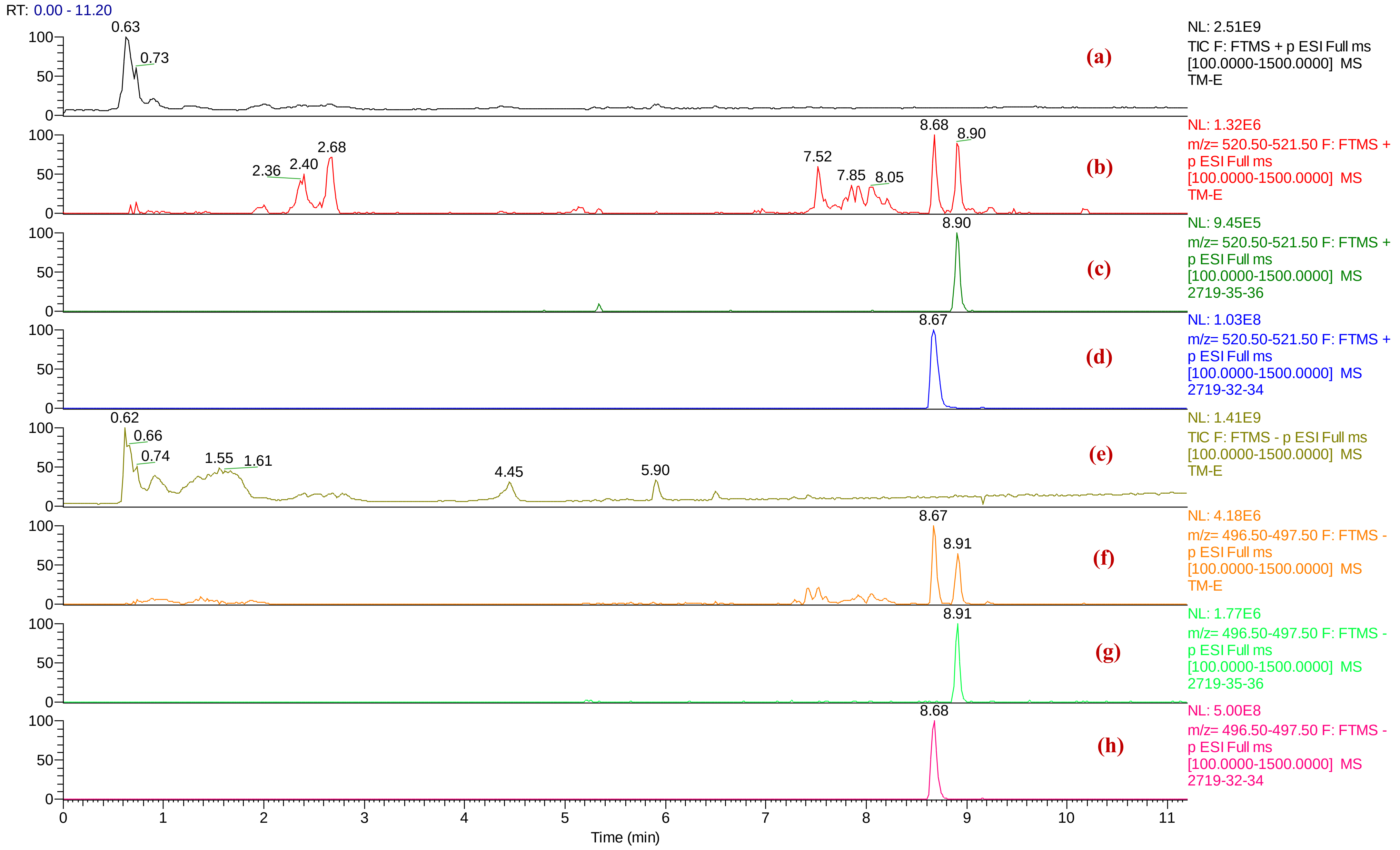

Fig. S123 Overlaid (a) (+)-TIC of an ethanol extract prepared by refluxing of the commercially available "tian ma" sample for 1 h; (b) the chromatogram of the extracted positive ion at *m/z* 521 $[M + Na]^+$ from (a); (c) and (d) (+)-TIC of aqueous solutions of compounds **7** and **10**; (e) (–)-TIC of an ethanol extract prepared by refluxing of the commercially available "tian ma" sample for 1 h; (f) the chromatogram of the extracted negative ion at *m/z* 497 $[M - H]^-$ from (e); (g) and (h) (–)-TIC of aqueous solutions of compounds **7** and **10**

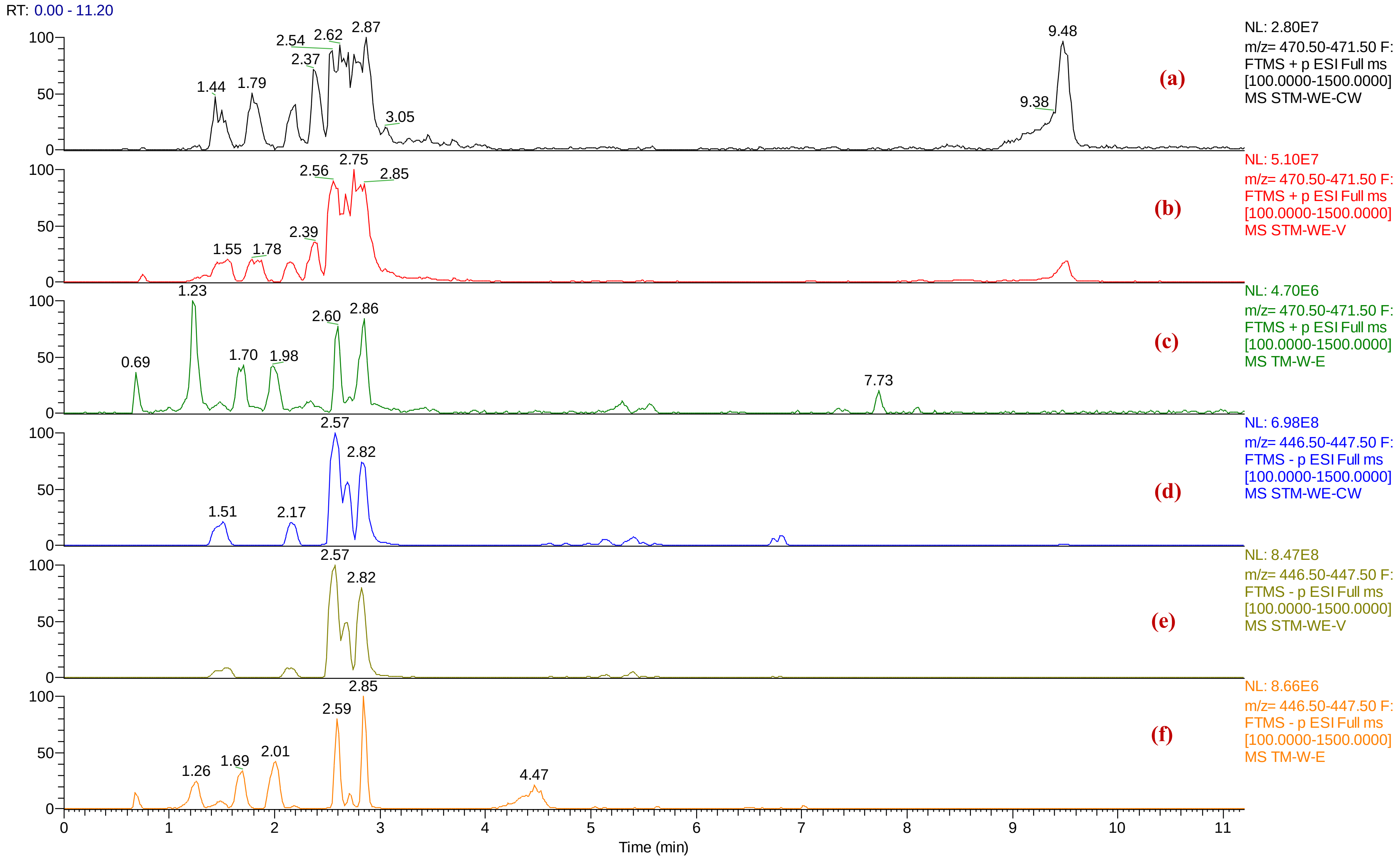

Fig. S124 Overlaid UPLC-HRESIMS chromatograms of the extracted positive ion at *m/z* 471 $[M + Na]^+$ from (+)-TIC of: (a) an aqueous extract prepared by soaking of the freeze-dried sample of the freshly collected *G. elata* rhizomes at room temperature for 24 h; (b) an aqueous extract prepared by refluxing of the freeze-dried sample of the freshly collected *G. elata* rhizomes for 1 h; (c) an aqueous extract prepared by refluxing of the commercially available "tian ma" sample for 1 h; and (d)–(f) for the extracted negative ion at *m/z* 447 $[M - H]^-$ from (–)-TIC of (a)–(c), respectively

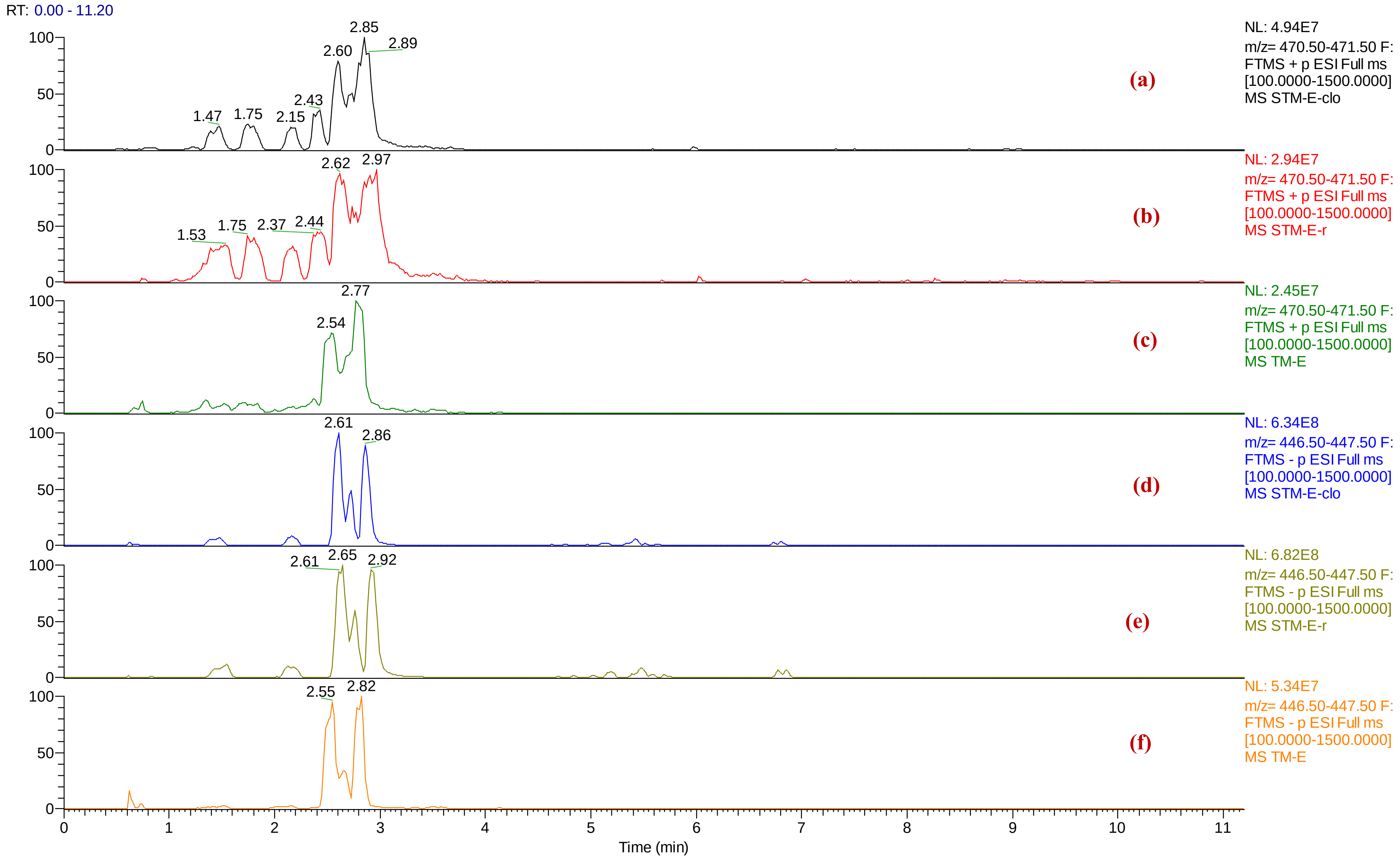

Fig. S125 Overlaid UPLC-HRESIMS chromatograms of the extracted positive ion at *m/z* 471 $[M + Na]^+$ from (+)-TIC of: (a) an ethanol extract prepared by soaking of the freeze-dried sample of the freshly collected *G. elata* rhizomes at room temperature for 24 h; (b) an ethanol extract prepared by refluxing of the freeze-dried sample of the freshly collected *G. elata* rhizomes for 1 h; (c) an ethanol extract prepared by refluxing of the commercially available “tian ma” sample for 1 h; and (d)–(f) for the extracted negative ion at *m/z* 447 $[M - H]^-$ from (–)-TIC of (a)–(c), respectively

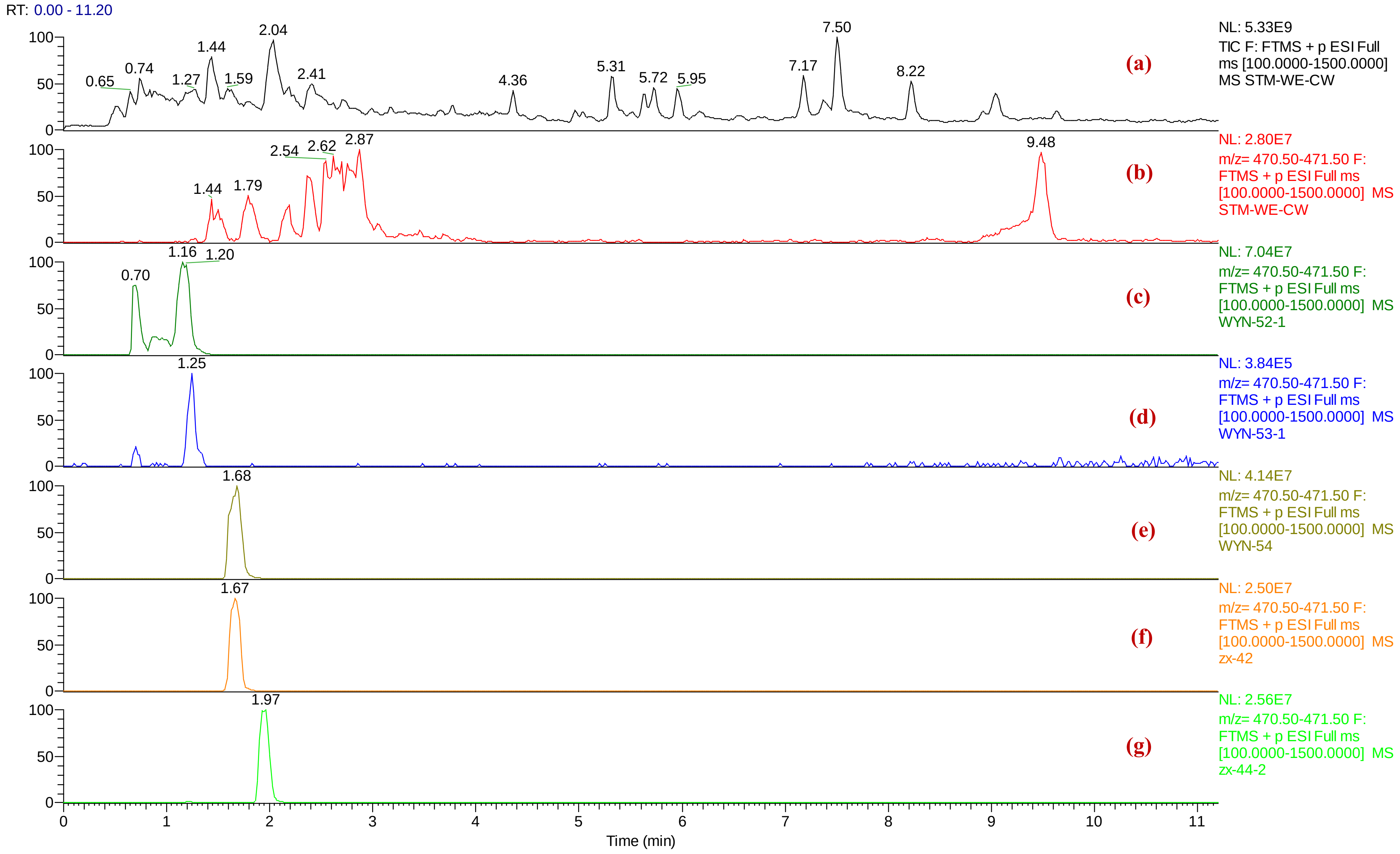

Fig. S126 Overlaid (a) (+)-TIC of an aqueous extract prepared by soaking of the freeze-dried sample of the freshly collected *G. elata* rhizomes at room temperature for 24 h; (b) the chromatogram of the extracted positive ion at *m/z* 471 $[M + Na]^+$ from (a); (c)–(g) (+)-TIC of aqueous solutions of compounds **9**, 4-[*β*-D-glucopyranosyl-(1→6)-*β*-D-glucopyranosyloxy]benzyl alcohol, 4-[*a*-D-glucopyranosyl-(1→4)-*β*-D-glucopyranosyloxy]benzyl alcohol, 4-[*β*-D-glucopyranosyl-(1→3)-*β*-D-glucopyranosyloxy]benzyl alcohol, and 4-[*β*-D-glucopyranosyl-(1→4)-*β*-D-glucopyranosyloxy]benzyl alcohol

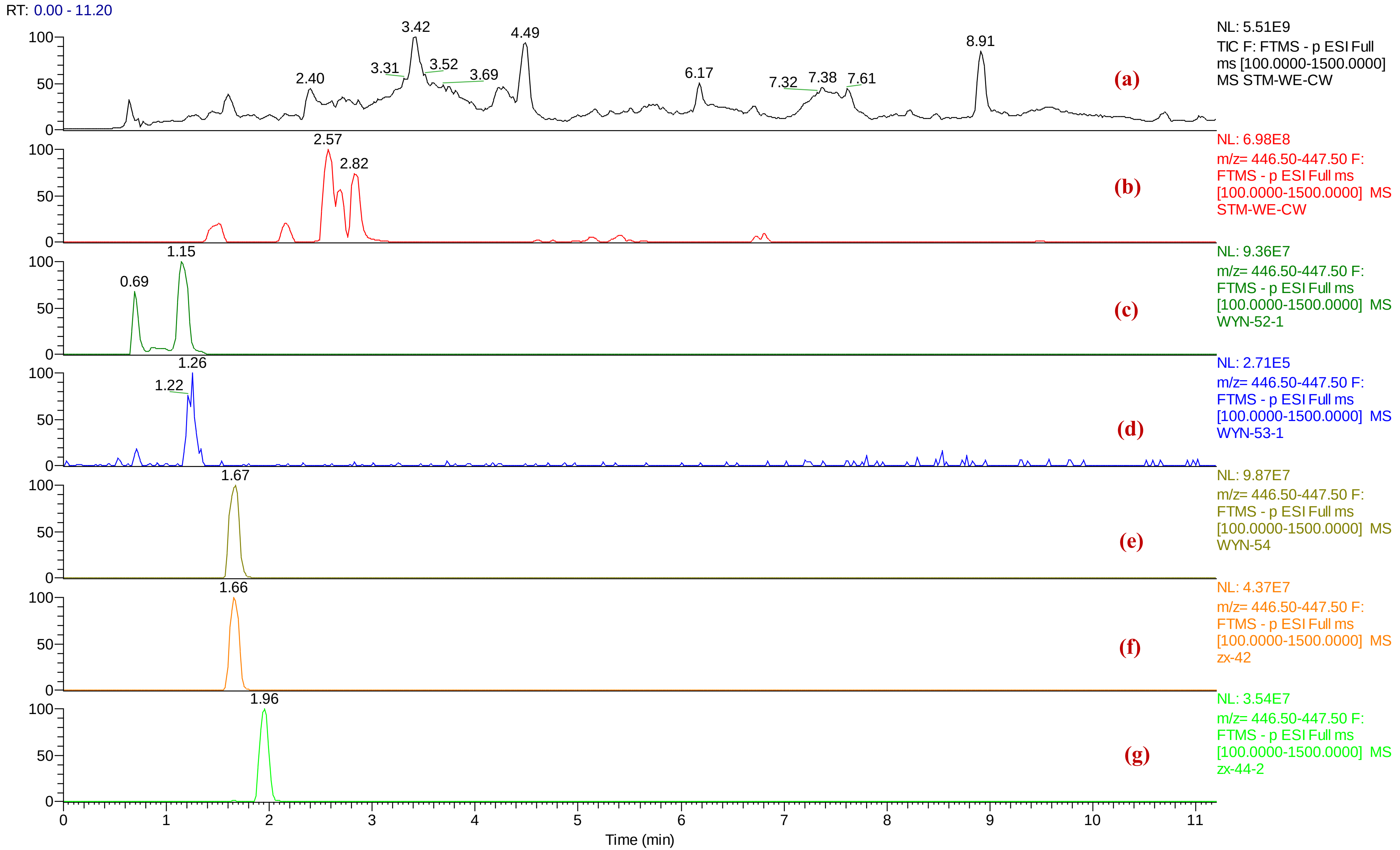

Fig. S127 Overlaid (a) (–)-TIC of an aqueous extract prepared by soaking of the freeze-dried sample of the freshly collected *G. elata* rhizomes at room temperature for 24 h; (b) the chromatogram of the extracted negative ion at *m/z* 447 [M – H]⁻ from (a); (c)–(g) (–)-TIC of aqueous solutions of compounds **9**, 4-[*β*-D-glucopyranosyl-(1→6)-*β*-D-glucopyranosyloxy]benzyl alcohol, 4-[*a*-D-glucopyranosyl-(1→4)-*β*-D-glucopyranosyloxy]benzyl alcohol, 4-[*β*-D-glucopyranosyl-(1→3)-*β*-D-glucopyranosyloxy]benzyl alcohol, and 4-[*β*-D-glucopyranosyl-(1→4)-*β*-D-glucopyranosyloxy]benzyl alcohol

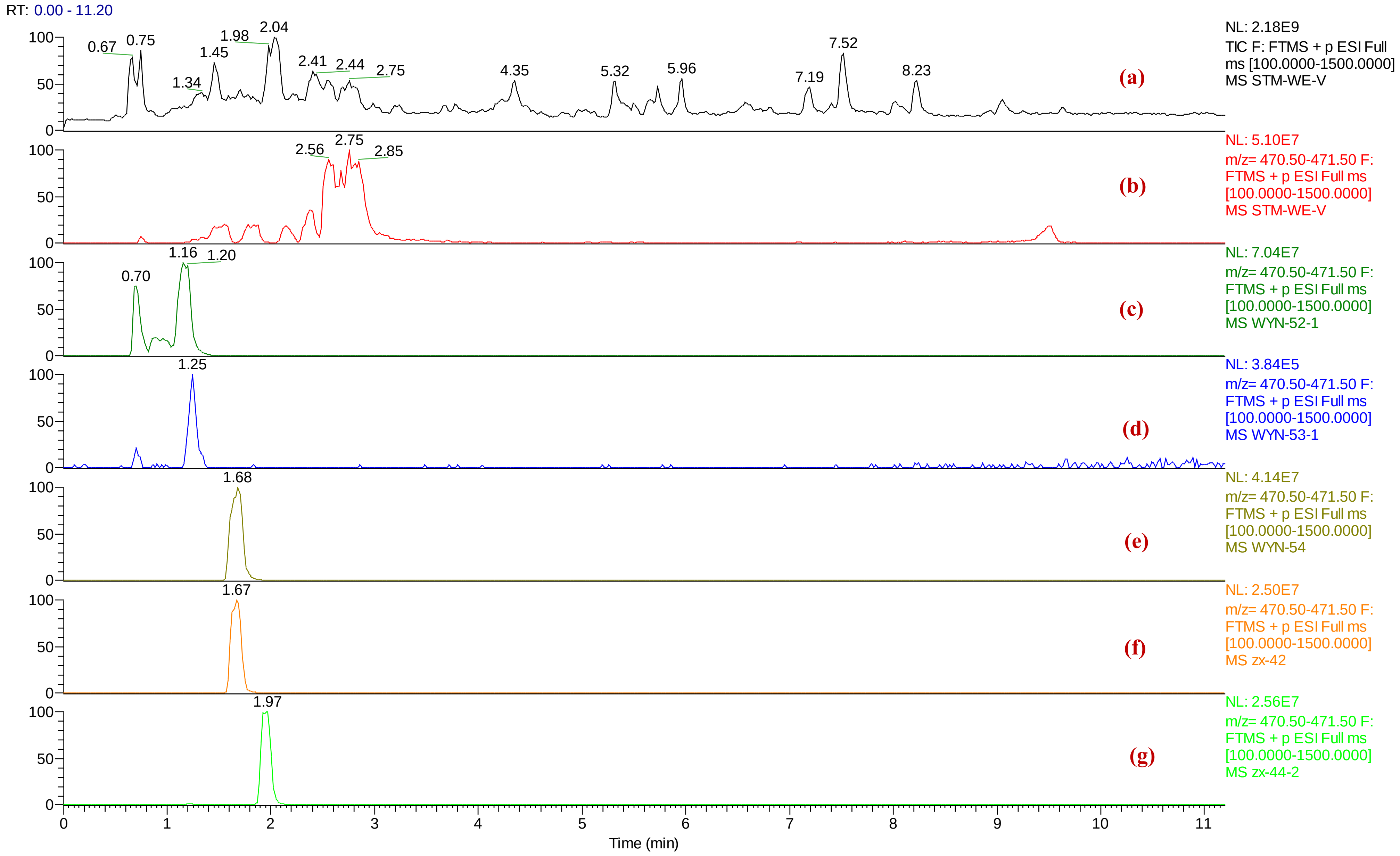

Fig. S128 Overlaid (a) (+)-TIC of an aqueous extract prepared by refluxing of the freeze-dried sample of the freshly collected *G. elata* rhizomes for 1 h; (b) the chromatogram of the extracted positive ion at *m/z* 471 $[M + Na]^+$ from (a); (c)–(g) (+)-TIC of aqueous solutions of compounds **9**, 4-[*β*-D-glucopyranosyl-(1→6)-*β*-D-glucopyranosyloxy]benzyl alcohol, 4-[*a*-D-glucopyranosyl-(1→4)-*β*-D-glucopyranosyloxy]benzyl alcohol, 4-[*β*-D-glucopyranosyl-(1→3)-*β*-D-glucopyranosyloxy]benzyl alcohol, and 4-[*β*-D-glucopyranosyl-(1→4)-*β*-D-glucopyranosyloxy]benzyl alcohol

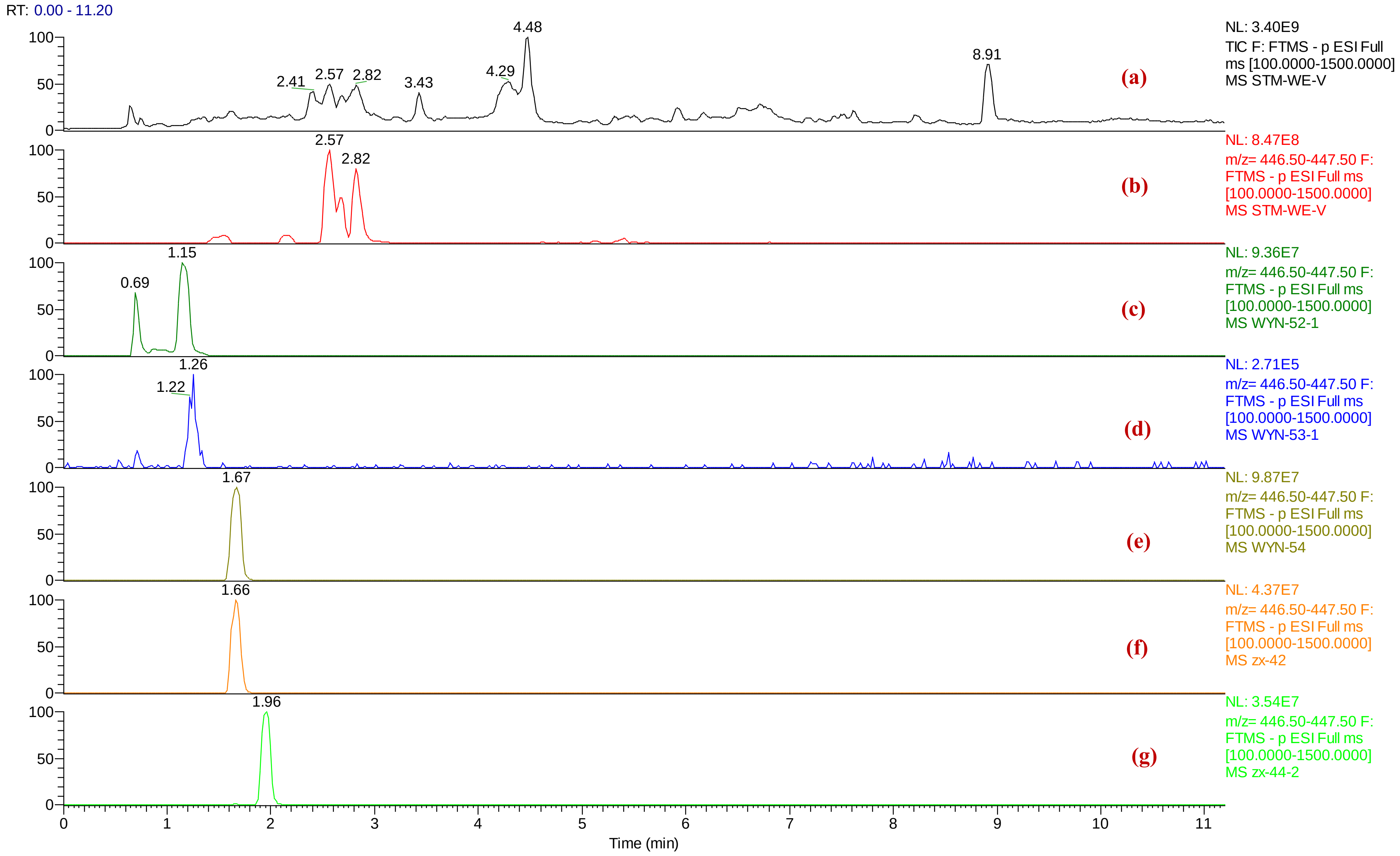

Fig. S129 Overlaid (a) (–)-TIC of an aqueous extract prepared by refluxing of the freeze-dried sample of the freshly collected *G. elata* rhizomes for 1 h; (b) the chromatogram of the extracted negative ion at *m/z* 447 $[M - H]^-$ from (a); (c)–(g) (–)-TIC of aqueous solutions of compounds **9**, 4-[*β*-D-glucopyranosyl-(1→6)-*β*-D-glucopyranosyloxy]benzyl alcohol, 4-[*a*-D-glucopyranosyl-(1→4)-*β*-D-glucopyranosyloxy]benzyl alcohol, 4-[*β*-D-glucopyranosyl-(1→3)-*β*-D-glucopyranosyloxy]benzyl alcohol, and 4-[*β*-D-glucopyranosyl-(1→4)-*β*-D-glucopyranosyloxy]benzyl alcohol

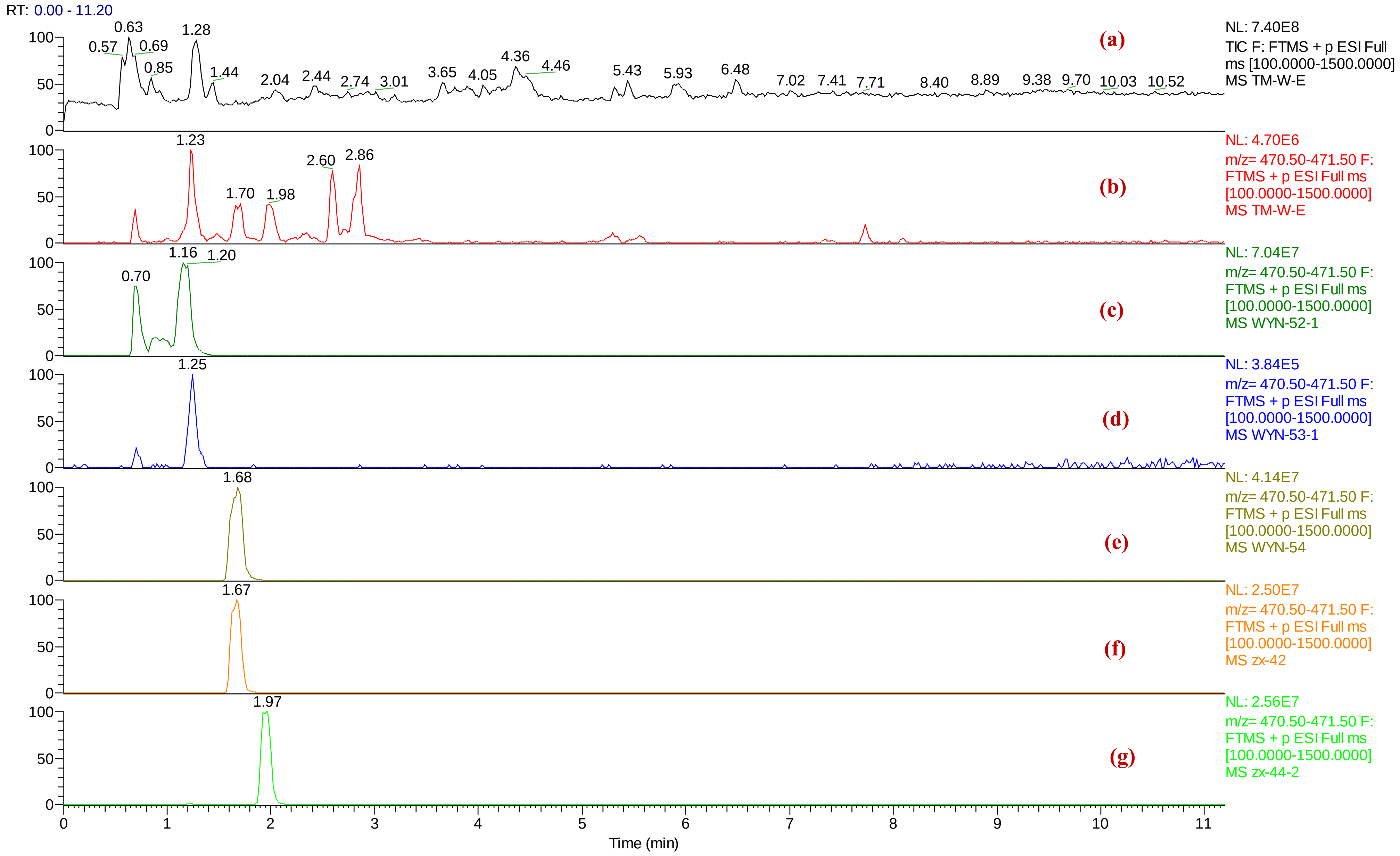

Fig. S130 Overlaid (a) (+)-TIC of an aqueous extract prepared by refluxing of the commercially available "tian ma" sample for 1 h; (b) the chromatogram of the extracted positive ion at *m/z* 471 $[M + Na]^+$ from (a); (c)–(g) (+)-TIC of aqueous solutions of compounds **9**, 4-[*β*-D-glucopyranosyl-(1→6)-*β*-D-glucopyranosyloxy]benzyl alcohol, 4-[*a*-D-glucopyranosyl-(1→4)-*β*-D-glucopyranosyloxy]benzyl alcohol, 4-[*β*-D-glucopyranosyl-(1→3)-*β*-D-glucopyranosyloxy]benzyl alcohol, and 4-[*β*-D-glucopyranosyl-(1→4)-*β*-D-glucopyranosyloxy]benzyl alcohol

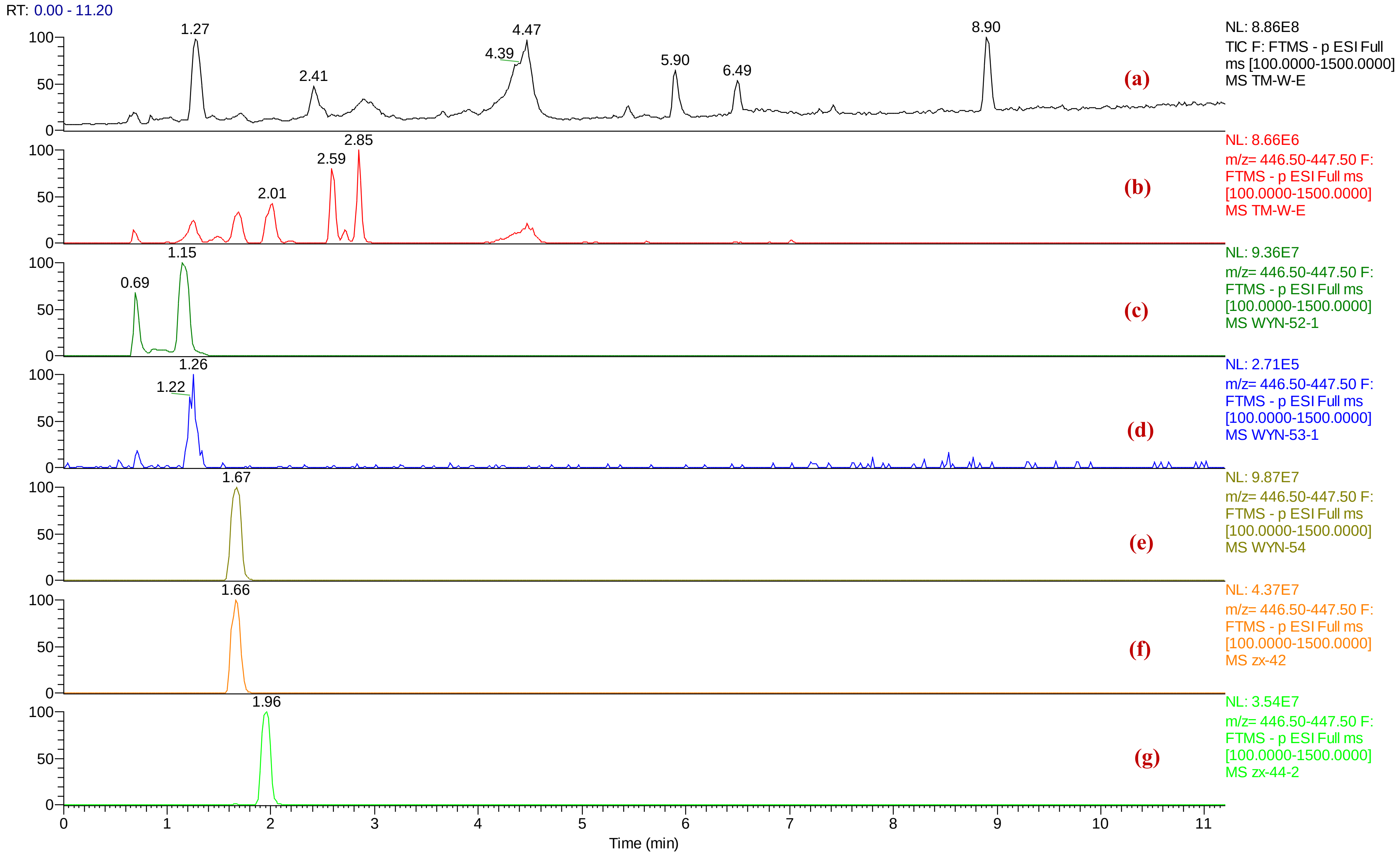

Fig. S131 Overlaid (a) (–)-TIC of an aqueous extract prepared by refluxing of the commercially available "tian ma" sample for 1 h; (b) the chromatogram of the extracted negative ion at *m/z* 447 $[M - H]^-$ from (a); (c)–(g) (–)-TIC of aqueous solutions of compounds **9**, 4-[*β*-D-glucopyranosyl-(1→6)-*β*-D-glucopyranosyloxy]benzyl alcohol, 4-[*a*-D-glucopyranosyl-(1→4)-*β*-D-glucopyranosyloxy]benzyl alcohol, 4-[*β*-D-glucopyranosyl-(1→3)-*β*-D-glucopyranosyloxy]benzyl alcohol, and 4-[*β*-D-glucopyranosyl-(1→4)-*β*-D-glucopyranosyloxy]benzyl alcohol

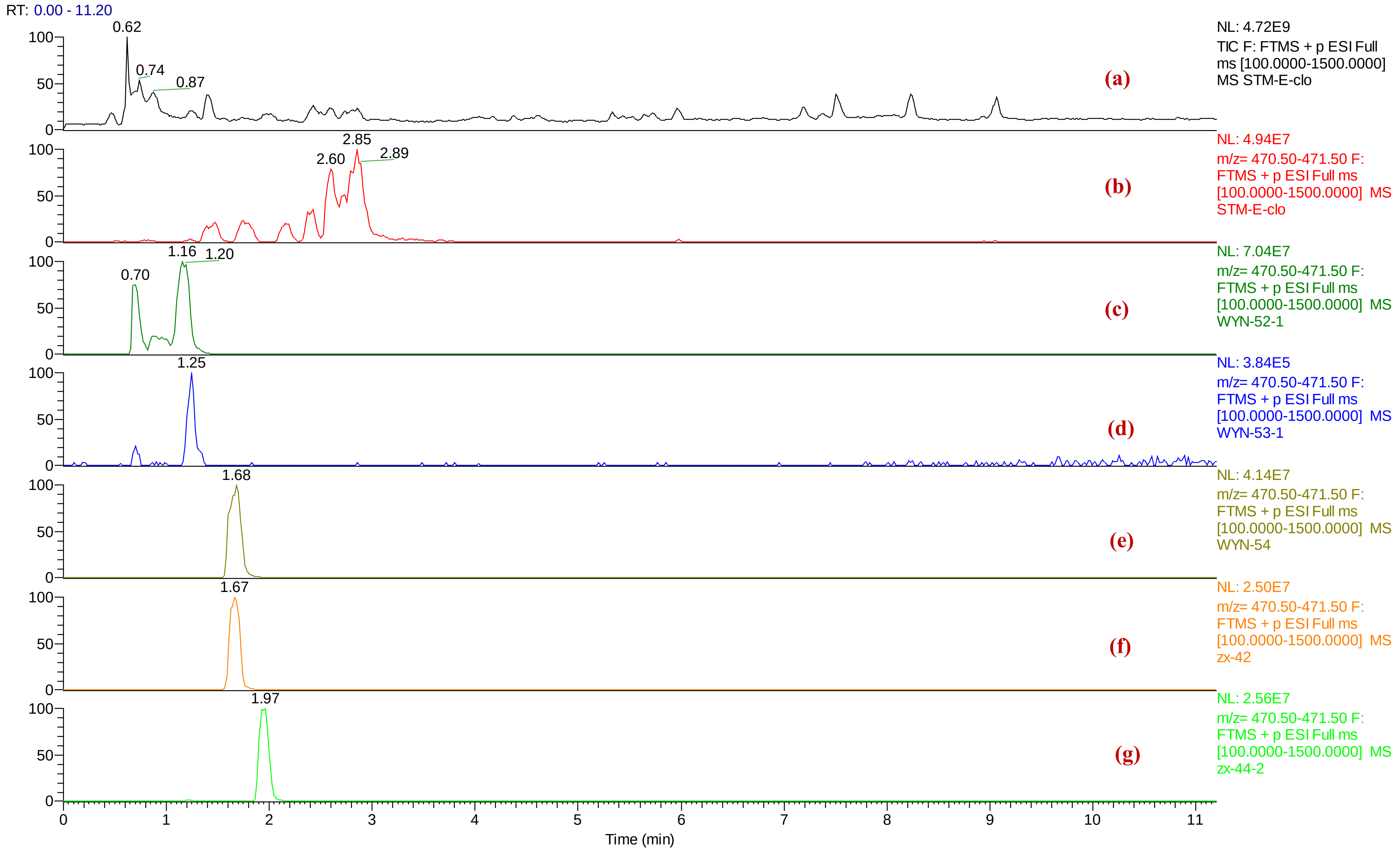

Fig. S132 Overlaid (a) (+)-TIC of an ethanol extract prepared by soaking of the freeze-dried sample of the freshly collected *G. elata* rhizomes at room temperature for 24 h; (b) the chromatogram of the extracted positive ion at *m/z* 471 $[M + Na]^+$ from (a); (c)–(g) (+)-TIC of aqueous solutions of compounds **9**, 4-[*β*-D-glucopyranosyl-(1→6)-*β*-D-glucopyranosyloxy]benzyl alcohol, 4-[*a*-D-glucopyranosyl-(1→4)-*β*-D-glucopyranosyloxy]benzyl alcohol, 4-[*β*-D-glucopyranosyl-(1→3)-*β*-D-glucopyranosyloxy]benzyl alcohol, and 4-[*β*-D-glucopyranosyl-(1→4)-*β*-D-glucopyranosyloxy]benzyl alcohol

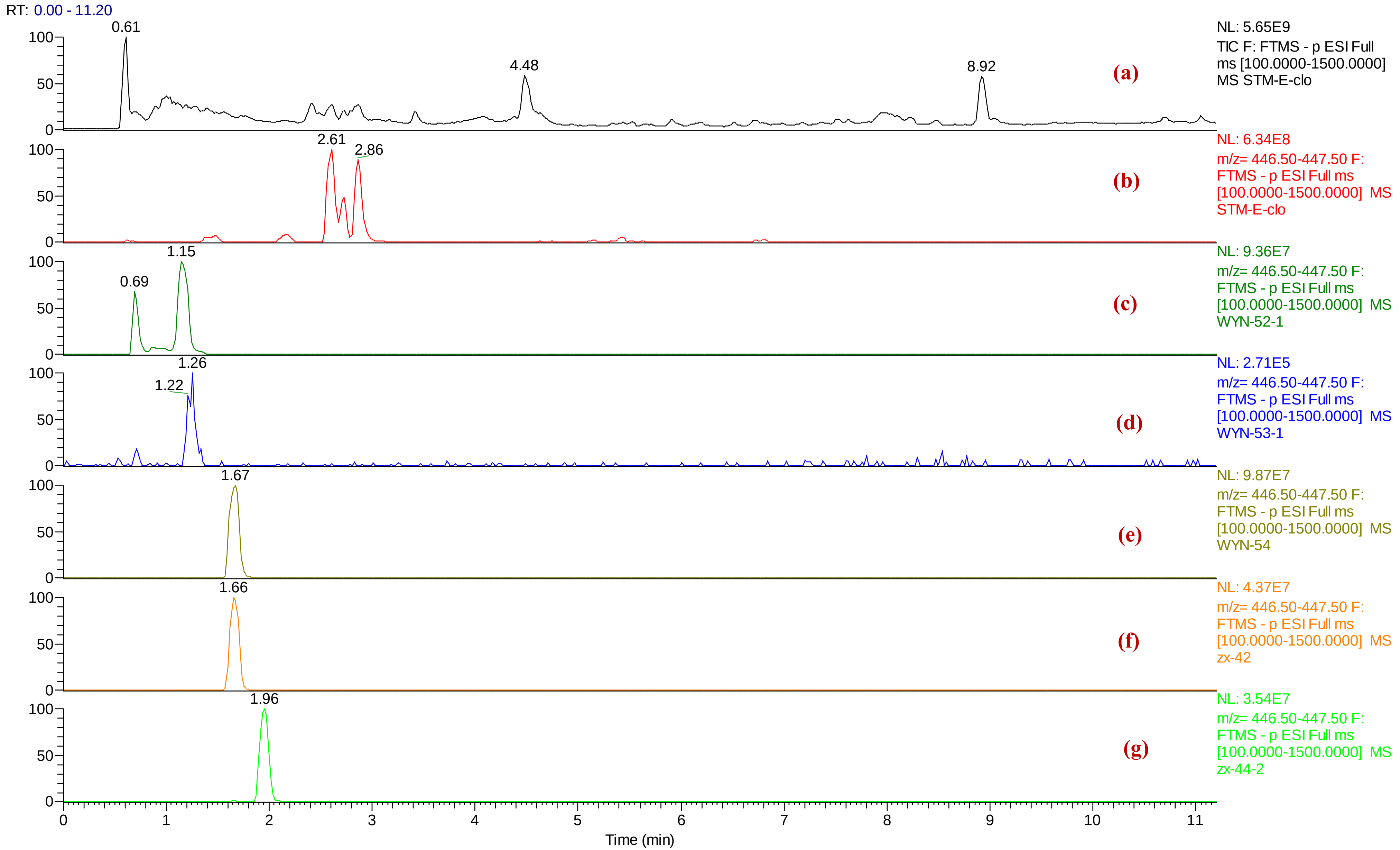

Fig. S133 Overlaid (a) (–)-TIC of an ethanol extract prepared by soaking of the freeze-dried sample of the freshly collected *G. elata* rhizomes at room temperature for 24 h; (b) the chromatogram of the extracted negative ion at *m/z* 447 $[M - H]^-$ from (a); (c)–(g) (–)-TIC of aqueous solutions of compounds **9**, 4-[*β*-D-glucopyranosyl-(1→6)-*β*-D-glucopyranosyloxy]benzyl alcohol, 4-[*a*-D-glucopyranosyl-(1→4)-*β*-D-glucopyranosyloxy]benzyl alcohol, 4-[*β*-D-glucopyranosyl-(1→3)-*β*-D-glucopyranosyloxy]benzyl alcohol, and 4-[*β*-D-glucopyranosyl-(1→4)-*β*-D-glucopyranosyloxy]benzyl alcohol

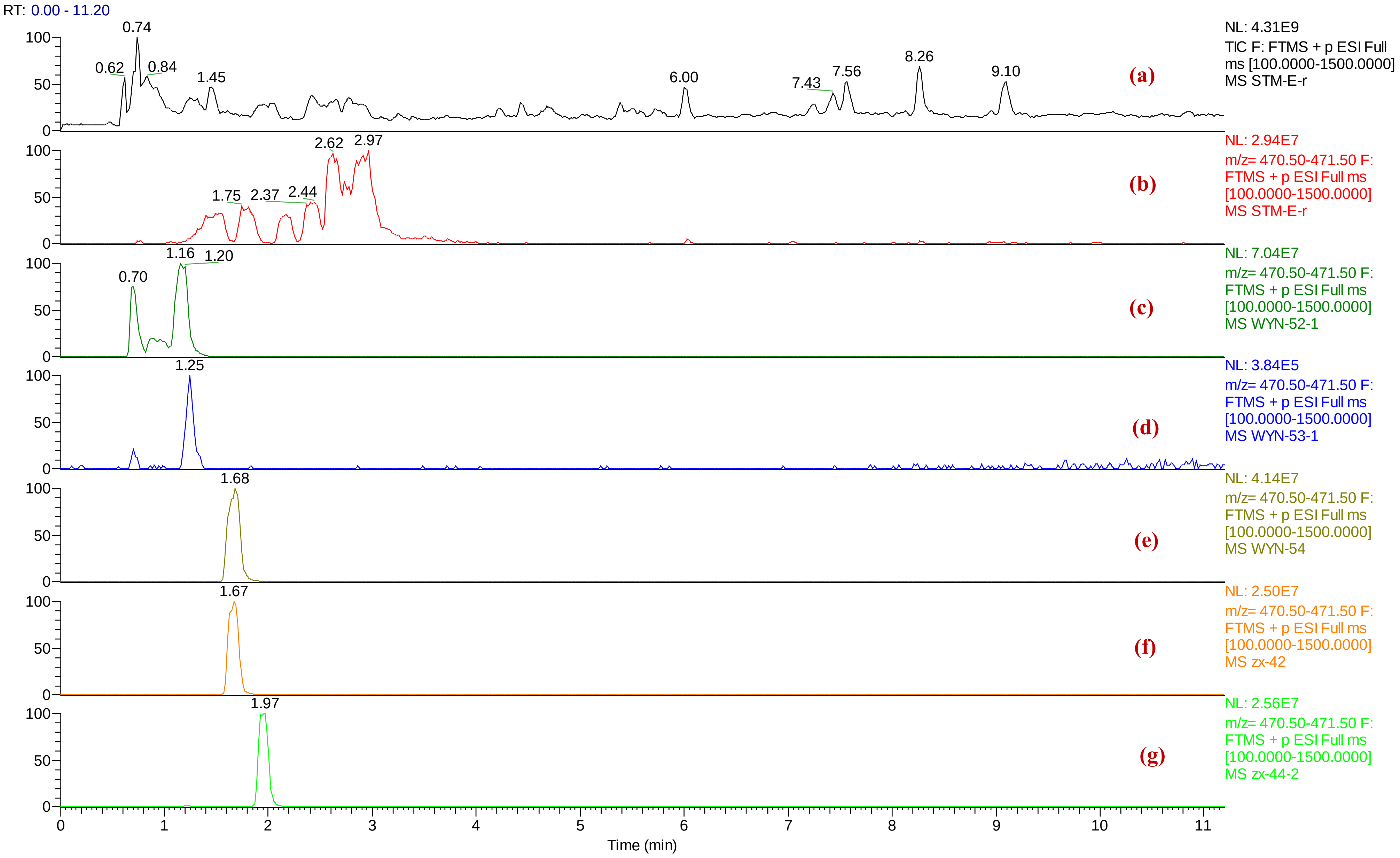

Fig. S134 Overlaid (a) (+)-TIC of an ethanol extract prepared by refluxing of the freeze-dried sample of the freshly collected *G. elata* rhizomes for 1 h; (b) the chromatogram of the extracted positive ion at *m/z* 471 $[M + Na]^+$ from (a); (c)–(g) (+)-TIC of aqueous solutions of compounds **9**, 4-[*β*-D-glucopyranosyl-(1→6)-*β*-D-glucopyranosyloxy]benzyl alcohol, 4-[*a*-D-glucopyranosyl-(1→4)-*β*-D-glucopyranosyloxy]benzyl alcohol, 4-[*β*-D-glucopyranosyl-(1→3)-*β*-D-glucopyranosyloxy]benzyl alcohol, and 4-[*β*-D-glucopyranosyl-(1→4)-*β*-D-glucopyranosyloxy]benzyl alcohol

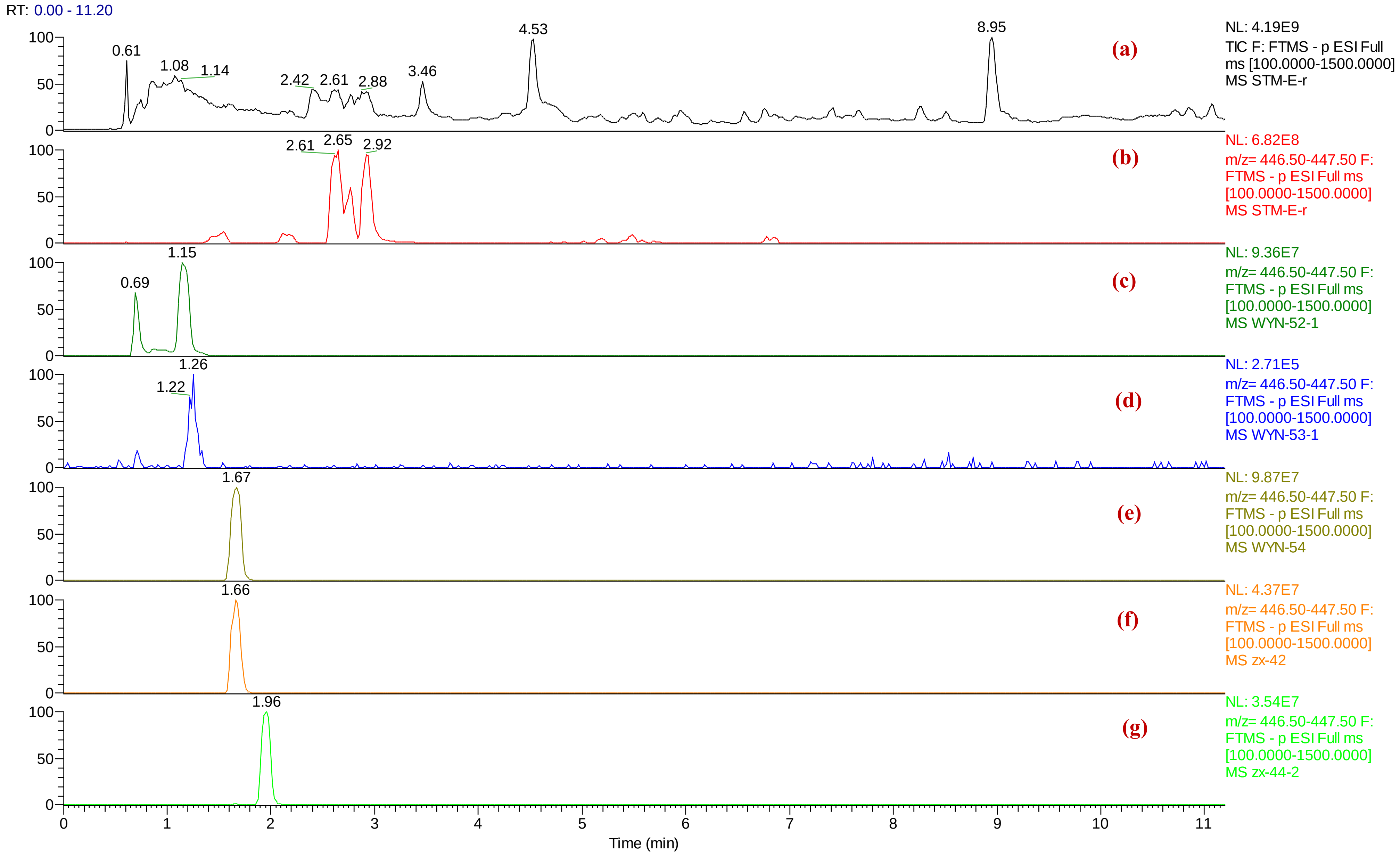

Fig. S135 Overlaid (a) (–)-TIC of an ethanol extract prepared by refluxing of the freeze-dried sample of the freshly collected *G. elata* rhizomes for 1 h; (b) the chromatogram of the extracted negative ion at *m/z* 447 [M – H]$^-$ from (a); (c)–(g) (–)-TIC of aqueous solutions of compounds **9**, 4-[*β*-D-glucopyranosyl-(1→6)-*β*-D-glucopyranosyloxy]benzyl alcohol, 4-[*a*-D-glucopyranosyl-(1→4)-*β*-D-glucopyranosyloxy]benzyl alcohol, 4-[*β*-D-glucopyranosyl-(1→3)-*β*-D-glucopyranosyloxy]benzyl alcohol, and 4-[*β*-D-glucopyranosyl-(1→4)-*β*-D-glucopyranosyloxy]benzyl alcohol

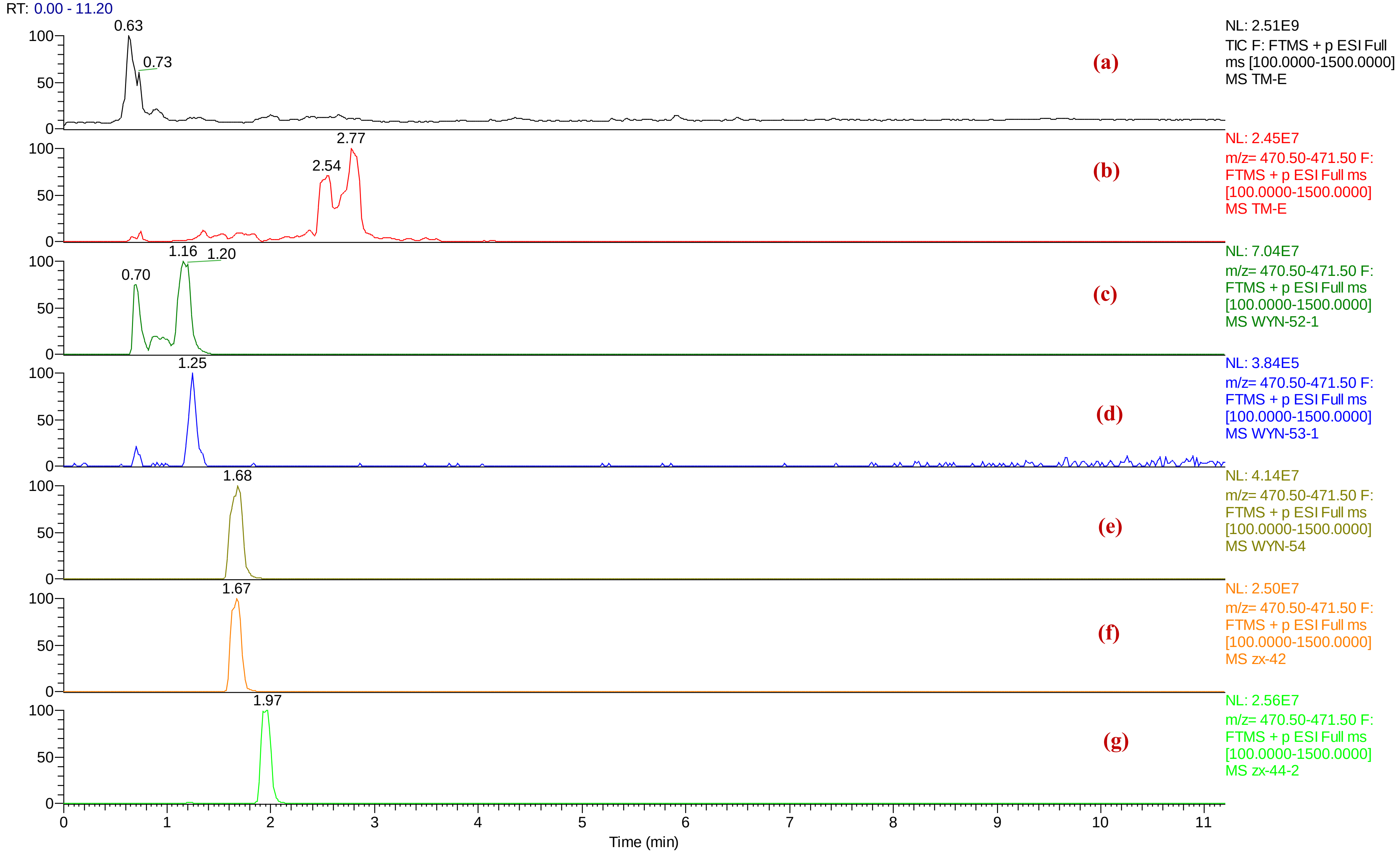

Fig. S136 Overlaid (a) (+)-TIC of an ethanol extract prepared by refluxing of the commercially available "tian ma" sample for 1 h; (b) the chromatogram of the extracted positive ion at *m/z* 471 [M + Na]$^+$ from (a); (c)–(g) (+)-TIC of aqueous solutions of compounds **9**, 4-[*β*-D-glucopyranosyl-(1→6)-*β*-D-glucopyranosyloxy]benzyl alcohol, 4-[*a*-D-glucopyranosyl-(1→4)-*β*-D-glucopyranosyloxy]benzyl alcohol, 4-[*β*-D-glucopyranosyl-(1→3)-*β*-D-glucopyranosyloxy]benzyl alcohol, and 4-[*β*-D-glucopyranosyl-(1→4)-*β*-D-glucopyranosyloxy]benzyl alcohol

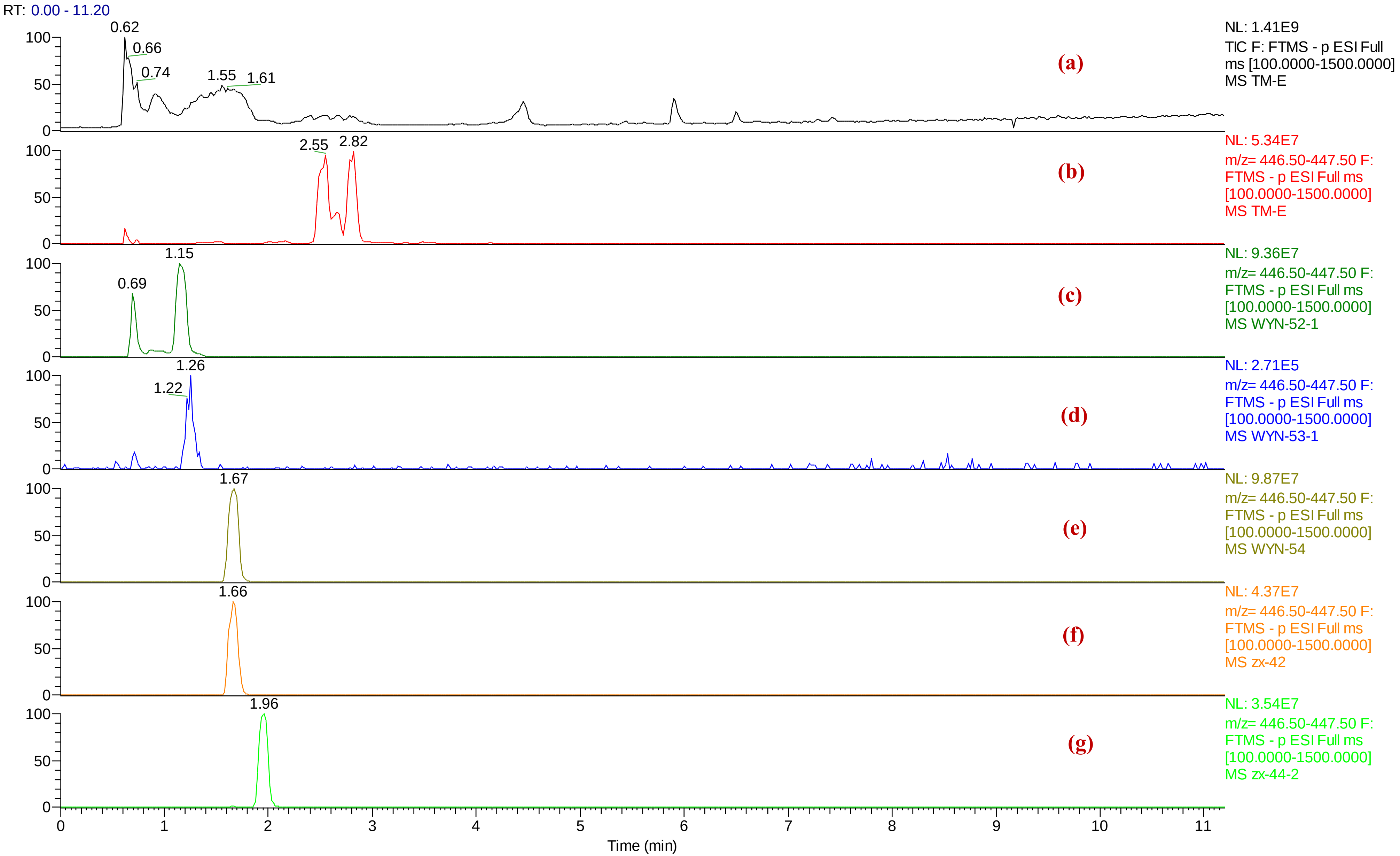

Fig. S137 Overlaid (a) (–)-TIC of an ethanol extract prepared by refluxing of the commercially available "tian ma" sample for 1 h; (b) the chromatogram of the extracted negative ion at *m/z* 447 $[M - H]^-$ from (a); (c)–(g) (–)-TIC of aqueous solutions of compounds **9**, 4-[*β*-D-glucopyranosyl-(1→6)-*β*-D-glucopyranosyloxy]benzyl alcohol, 4-[*a*-D-glucopyranosyl-(1→4)-*β*-D-glucopyranosyloxy]benzyl alcohol, 4-[*β*-D-glucopyranosyl-(1→3)-*β*-D-glucopyranosyloxy]benzyl alcohol, and 4-[*β*-D-glucopyranosyl-(1→4)-*β*-D-glucopyranosyloxy]benzyl alcohol
